# An automated protocol to construct flexibility parameters for classical forcefields: applications to metal–organic frameworks†

**DOI:** 10.1039/d4ra01859a

**Published:** 2024-07-19

**Authors:** Reza Ghanavati, Alma C. Escobosa, Thomas A. Manz

**Affiliations:** a Chemical & Materials Engineering, New Mexico State University Las Cruces NM 88001 USA tmanz@nmsu.edu

## Abstract

In this work, forcefield flexibility parameters were constructed and validated for more than 100 metal–organic frameworks (MOFs). We used atom typing to identify bond types, angle types, and dihedral types associated with bond stretches, angle bends, dihedral torsions, and other flexibility interactions. Our work used Manz's angle-bending and dihedral-torsion model potentials. For a crystal structure containing *N*_atoms_ in its unit cell, the number of independent flexibility interactions is 3(*N*_atoms_ − 1). Because the number of bonds, angles, and dihedrals is normally much larger than 3(*N*_atoms_ − 1), these internal coordinates are redundant. To reduce (but not eliminate) this redundancy, our protocol prunes dihedral types in a way that preserves symmetry equivalency. Next, each dihedral type is classified as non-rotatable, hindered, rotatable, or linear. We introduce a smart selection method that identifies which particular torsion modes are important for each rotatable dihedral type. Then, we computed the force constants for all flexibility interactions together *via* LASSO regression (*i.e.*, regularized linear least-squares fitting) of the training dataset. LASSO automatically identifies and removes unimportant forcefield interactions. For each MOF, the reference dataset was quantum-mechanically-computed in VASP *via* DFT with dispersion and included: (i) finite-displacement calculations along every independent atom translation mode, (ii) geometries randomly sampled *via ab initio* molecular dynamics (AIMD), (iii) the optimized ground-state geometry using experimental lattice parameters, and (iv) rigid torsion scans for each rotatable dihedral type. After training, the flexibility model was validated across geometries that were not part of the training dataset. For each MOF, we computed the goodness of fit (*R*-squared value) and the root-mean-squared error (RMSE) separately for the training and validation datasets. We compared flexibility models with and without bond–bond cross terms. Even without cross terms, the model yielded *R*-squared values of 0.910 (avg across all MOFs) ± 0.018 (st. dev.) for atom-in-material forces in the validation datasets. Our SAVESTEPS protocol should find widespread applications to parameterize flexible forcefields for material datasets. We performed molecular dynamics simulations using these flexibility parameters to compute heat capacities and thermal expansion coefficients for two MOFs.

## Abstract 2

This article introduces an automated protocol for optimizing the flexibility parameters (to be used in classical forcefields) through regularized linear regression and applies this new method to 116 metal–organic frameworks.

## Graphical abstract

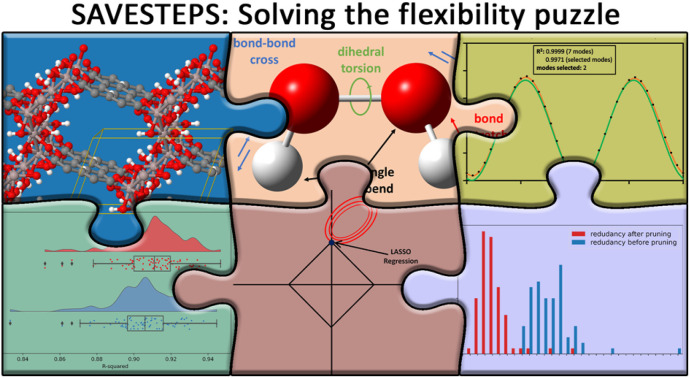

## 1. Introduction

Optimizing forcefields for classical molecular dynamics and Monte Carlo simulations of materials is a pragmatic task focusing on practical aspects of usability and accuracy. In this work, we focus on applications to porous solids such as metal–organic frameworks (MOFs). Several different approaches have been developed to optimize the flexibility parameters (*e.g.*, bond stretches, angle bends, dihedral torsions, *etc.*) used to construct such forcefields *via* fitting to quantum-mechanically-computed reference data. Classical forcefields whose parameters have been fitted to quantum-mechanically-computed reference data are often referred to as first-principles-derived forcefields or quantum-mechanically-derived forcefields (QMDFFs).^1–6^ Dubbeldam *et al.* recently reviewed parameterization schemes for constructing flexible forcefields for MOFs.^7,8^ In pioneering works, several authors introduced first-principles-derived flexible forcefields for specific MOFs.^9,10^

In ‘adoption-plus-tweaking’ approaches, the flexibility parameter values for a MOF's organic linkers are adopted from a prior forcefield (such as an organic or biomolecular or generic/universal forcefield), then combined with a few new parameters (*e.g.*, to describe the metal–ligand coupling or other interactions), and then tweaked to reproduce a handful of desired experimental or computed properties. Such ‘adoption-plus-tweaking’ approaches have been effective and pragmatic strategies to quickly assemble functional flexible forcefields for MOFs.^11–19^ However, they are only partial re-optimizations and not full optimizations of the flexibility parameters' values. This article's focus is on approaches that fully optimize the flexibility parameters' values rather than ‘adoption-plus-tweaking’ approaches that partially re-optimize them.

Partial Hessian-fitting strategies (such as the Seminario method^20,21^) that attempt to optimize the flexibility force constants sequentially one-at-a-time rather than simultaneously are generally ill-advised. When the active internal coordinates used are redundant, the corresponding flexibility terms are coupled together and do not vary independently of each other. For this reason, the corresponding force constants must be optimized simultaneously rather than sequentially one-at-a-time. A previously published attempt to optimize the flexibility parameters for a MOF using the Seminario method failed.^22^ Specifically, the Seminario method often gives angle-bending force constants that are too stiff, sometimes being as much as a factor of two too large.^20,22^

Strategies that only fit the full Hessian^23^ are not generally robust, because they only sample geometries on the potential energy landscape that are differentially close to the optimized ground-state geometry. This problem can only be fixed by also including in the training dataset some (non-Hessian) geometries that are far away from the optimized ground-state geometry.

Several authors used genetic or evolutionary optimization algorithms to optimize forcefield flexibility parameters for specific MOFs.^1,24^ For example, recent generations of the MOF-FF approach use a genetic algorithm or a covariance matrix adaptive evolutionary strategy (CMA-ES) to optimize the force constants.^25–27^ In the MOF-FF approach, terms including preset non-bonded parameter values (*e.g.*, atomic charges and van der Waals (VDW) parameters) are included in the Hessian and energy expressions when the flexibility parameter values are optimized.^25,26^ The MOF-FF approach uses the quantum-mechanically-computed optimized geometry and Hessian as target reference data to fit the forcefield's flexibility parameters.^25,26^ Since the Hessian corresponds to small displacements about the equilibrium geometry, it appears that the large displacements associated with rotational barriers are insufficiently sampled in the MOF-FF parameterization protocol. For this reason, the dihedral torsion terms may not be accurately (or sufficiently) sampled in the MOF-FF parameterization protocol.

Gabrieli *et al.* used force matching to optimize flexibility parameters for the ZIF-8 MOF, the silicalite zeolite, and the molecules methane and carbon dioxide.^28^ Their protocol involved the following steps. First, they performed *ab initio* molecular dynamics (AIMD) calculations using density functional theory (DFT). Then, they used a constrained search optimization algorithm (specifically, L-BFGS-B) to calculate the flexibility parameter values that minimized the sum of squared differences between the DFT-computed and flexibility-model-computed atom-in-material forces across their training set of AIMD geometries.

The QuickFF approach first determines dihedral multiplicities and dihedral resting values, then it performs a series of quantum-mechanical calculations for perturbation trajectories along the corresponding internal coordinate (*i.e.*, bond length or bond angle) for each bond stretch and angle-bending term in the forcefield to compute the corresponding ‘resting value’ of the bond length or bond angle, and finally it uses least-squares fitting between the *ab initio* Hessian and the forcefield's Hessian to optimize all force constant values.^29^ While the original QuickFF protocol used non-periodic cluster models to represent periodic crystals, an updated QuickFF protocol was subsequently published that can use fully periodic models.^30^ The updated QuickFF protocol fits the mass-weighted Hessian instead of the non-mass-weighted Hessian, and it can include cross terms and/or anharmonic terms in the forcefield.^30^ The updated QuickFF protocol is a sequence of six major steps that involve optimizations and re-optimizations (aka tune-ups).^30^ A key feature of the QuickFF protocol is that terms including preset non-bonded parameter values (*e.g.*, atomic charges and VDW parameters) are included in the Hessian and energy expressions when the flexibility parameter values are optimized.^29,30^ The QuickFF approach was used in several studies to generate flexible forcefields for MOFs.^31–48^ According to the published descriptions, the QuickFF protocol does not currently treat dihedral torsions rigorously but instead uses a lone cosine mode potential for each dihedral, where each ABCD dihedral is assigned a multiplicity *m*_ABCD_.^29,30^ Obviously, many dihedrals cannot be described by such a restricted potential form. Those dihedrals that could not be described by such a simplified potential were neglected, and this may cause the parameterized forcefield to be inaccurate.^29,30^

Dubbeldam and coworkers developed flexible forcefields that were optimized to reproduce the elastic response properties or volume-*versus*-temperature curve of MOFs.^7,11,12,49^ These can be referred as ‘top-down’ approaches that focus on bulk response properties as opposed to ‘bottom-up’ approaches that focus on forces and motions of individual atoms and chemical groups within the material.

In the present article, we develop a different flexibility parameterization strategy that is based on Force Field Functional Theory (FFFT). As described in a companion article, FFFT studies “topics related to the functional representation of nonreactive forcefields to achieve various desirable properties”.^50^ Specific theoretical advances of FFFT that are directly relevant to the present article include:

(1) A new ansatz for separating the bonded potential energy from the nonbonded potential energy within a bonded cluster that does not introduce any new approximations and enables bonded parameters to be optimized using linear regression instead of requiring nonlinear regression.^50^ (Examples of a bonded cluster include a molecule or a MOF.) Manz's ansatz separates the bonded potential energy from the nonbonded potential energy in such a way that the ‘resting values’ of internal coordinates appearing in the forcefield's flexibility terms are identically equal to the equilibrium values of those internal coordinates in the isolated bonded cluster's optimized ground-state geometry.^50^ The forcefield's total potential energy is represented as^50^1
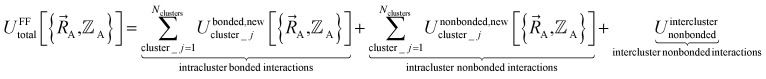
where 
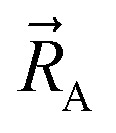
 is the position of atom A and 
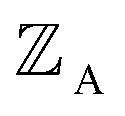
 is its atomic number (aka element number).

(2) Most importantly, Manz’ ansatz defines the intracluster bonded interactions in such a way that the atom-in-material forces for extremely small (*i.e.*, infinitesimal) displacements relative to the isolated bonded cluster's optimized ground-state geometry do not depend on any intracluster nonbonded interactions.^50^ This allows the intracluster bonded interactions to be rigorously parameterized up to second order (*i.e.*, within a harmonic approximation) without having to include the intracluster nonbonded interactions.^50^ (Manz's ansatz can be used to optimize the flexibility parameter terms so that the forcefield rigorously describes the anharmonicities (*i.e.*, third-order and higher-order derivatives of the energy), but this requires including intracluster nonbonded interactions when the bonded parameter values are optimized^50^). The present article focuses exclusively on parameterizing the intracluster bonded interactions (*i.e.*, parameterizing the flexibility terms) up to second-order derivatives in the energy. The intracluster nonbonded interactions and intercluster nonbonded interactions have been partly studied in several previous publications (co)authored by one of us^51–64^ and will be further studied in some of our upcoming publications.

(3) New angle-bending and dihedral torsion model potentials that are nearly universal, improve accuracy, improve numerical stability, and have a small number of adjustable parameters.^50^ Most importantly, these model potentials avoid derivative discontinuities (*i.e.*, force discontinuities) associated with linear bond angles.^50^

(4) “Forcefield design that guarantees the reference ground-state geometry is exactly reproduced as an equilibrium structure on the forcefield's potential energy landscape”.^50^ In this work, the reference ground state geometry consisted of the experimental lattice parameters defining the unit cell's size and shape plus DFT_with_dispersion optimized atom-in-material positions.

(5) “Well-designed methods to parameterize the forcefield from quantum-mechanically-computed and (optionally) experimental reference data”.^50^ The SAVESTEPS protocol introduced in the present article accomplishes this.

(6) “Computationally efficient embedded feature selection that identifies and removes unimportant forcefield terms”.^50^ Within the present article, we developed three important embedded feature selection techniques: (a) dihedral pruning as described in Sections 5.4.3 and 8.6.1, (b) smart selection of rotatable dihedral modes as described in Sections 7.1 and 8.5, and (c) least absolute shrinkage and selection operator (LASSO^65,66^) regression as described in Sections 7.4 and 8.6.

A key goal of this article is to create an automated workflow that allows a large number of materials to be processed efficiently. To the best of our knowledge, this is the first time first-principles-derived flexibility parameters have been optimized in a system-specific manner for more than one hundred MOFs in a single study. To date, ‘generic/universal’ forcefields (*e.g.*, UFF^67^ and UFF4MOF^68,69^) that attempt a common parameterization across multiple material types have not been accurate for describing dihedral torsions in MOFs, even though they do a reasonably good job of predicting equilibrium bond lengths, bond angles, and bulk moduli in many materials^68–70^ (however, some modifications to UFF4MOF are needed to treat rare earth elements^71^). We attribute this limitation of ‘generic/universal’ forcefields to the algebraic dependencies that mathematically couple dihedrals to each other and to other flexibility parameters due to the redundancy of flexibility parameters (especially dihedral angles). In contrast to non-bonded parameters that exhibit a high degree of transferability across similar chemical environments for a given second-neighbor-based atom type,^57^ the redundancy of flexibility parameters (especially dihedrals) impairs transferability of the flexibility parameter values (especially torsion potentials) between two different chemical building blocks. Because this redundancy is difficult to remove or avoid, and because torsion potentials are exquisitely sensitive to the chemical environment, we believe it is generally preferable to optimize flexibility parameter values specifically for each chemical building block rather than trying to transfer their values across different chemical building blocks. Here, the term ‘chemical building block’ could mean either a specific bonded cluster (such as a molecule or a MOF) or a specific monomer in a polymer (*e.g.*, a specific amino acid in a protein sequence, a specific base pair in DNA, or a specific RNA base, *etc.*). Thus, our strategy is to create an automated workflow that optimizes flexibility parameters specifically for each material.

Our protocol develops new best practices for the typing of bonds, angles, and dihedrals. We use Chen and Manz's second-neighbor-based atom typing scheme to define the atom types.^57^ To minimize (but not eliminate) internal coordinate redundancy, angles in 3- and 4-membered rings are flagged in the internal coordinate list and not used in the angle-bending potential, while diagonals in 4-membered rings are added to the list of Urey–Bradley^72^ stretches. A key strength of our parameterization protocol is the more accurate and more automated treatment of dihedral torsion modes than prior literature approaches. Key improvements of our approach include:

(a) Automated pruning of dihedral types to reduce (but not completely eliminate) internal coordinate redundancy; our protocol does this in a way that preserves symmetry equivalency.

(b) Automated classification of each dihedral type as (a) rotatable, (b) hindered, (c) non-rotatable, or (d) linear.

(c) Our protocol specifically performs a series of quantum-mechanical calculations for scans along each rotatable dihedral type. Our protocol automatically analyzes this data to determine which specific subset among the first seven possible torsion modes contribute to each rotatable dihedral torsion energy curve. This ensures each rotatable dihedral term has optimal form.

(d) Our protocol samples the rotatable dihedral barriers thoroughly by including an energy scan for each rotatable dihedral type when optimizing all of the force constants.

(e) As described above, our protocol uses Manz's^50^ new angle-bending and dihedral-torsion model potentials that avoid derivative discontinuities (*i.e.*, force discontinuities).

Previous forcefields included some but not all of these aspects for modeling dihedral torsions. The AMBER forcefield uses a truncated Fourier series expansion of the torsion potential for which particular modes were manually selected for different dihedral types based on dihedral scans (using quantum-chemistry calculations) to generate potential energy curves.^73,74^ Barone *et al.*'s forcefield parameterization protocol for molecules included (i) the classification of each dihedral as soft or stiff, (ii) dihedral scans (using quantum-chemistry calculations) to generate potential energy curves for soft dihedrals, and (iii) a truncated Fourier series expansion of the torsion potential for soft dihedrals.^5^ Grimme's QMDFF parameterization protocol included (i) the classification of each dihedral as rotatable or non-rotatable, (ii) dihedral scans (using tight-binding calculations) to generate potential energy curves for rotatable dihedrals, and (iii) a four-term distance-damped modified Fourier series expansion of the torsion potential for rotatable dihedrals.^6^

Our protocol includes physically-motivated non-negative bounds for some of the force constants. Specifically, we constrained force constants for the bond stretches, Urey–Bradley stretches, angle bends, non-rotatable/hindered dihedral torsions, and linear-dihedral torsions to be non-negative. We did not apply bounds to the bond–bond cross terms. If a rotatable dihedral torsion type had more than one active mode, no bounds were applied to the force constants associated with this torsion. If a rotatable dihedral torsion type had only one active mode, the force constant associated with this lone torsion mode was constrained to be non-negative. These choices are physically motivated as described in Section 7.4.

The remainder of this article is organized as follows. Section 2 describes the specific model potentials we used for bond stretches, angle bends, dihedral torsions, and other flexibility terms (*e.g.*, Urey–Bradley interactions, bond–bond cross terms). Section 3 gives an overview of the major features of our SAVESTEPS approach. Section 4 describes the crystal geometry verification steps we performed to ensure the crystal structures chosen were reliable. Section 5 describes the identification of bond types, angle types, and dihedral types. Section 5 also describes the pruning of redundant dihedral types and the classification of each dihedral type as rotatable, hindered, non-rotatable, or linear. Section 6 describes the quantum chemistry methods. Section 7 describes the rotatable dihedral mode selection and the regularized linear least-squares fitting that we performed to optimize all force constants. Section 7 also contains formulas for computing *R*-squared and root-mean-squared error (RMSE) that quantify how well the model performed. Section 8 presents and analyzes the computed flexibility parameterization results. Section 9 investigates whether the force constant values are transferable for matched types occurring in two different chemical structures. In Section 10, the heat capacity and coefficient of thermal expansion computed using molecular dynamics simulations for IRMOF-1 are compared to experimental measurements and to values computed using other forcefields. Section 10 also presents these computed bulk properties for MIL-53(Ga) using our flexibility model. Section 11 concludes. Note: in this article, function arguments are enclosed in square brackets; for example, *h*[*q*] would denote a function *h* that depends on *q*, while *h*(*q*) would denote *h* multiplied by *q*.

## 2. Model potentials for flexibility terms

### 2.1 Types of flexibility terms to include

As reviewed in the literature, the bonded interaction potential in non-reactive flexible forcefields is typically constructed by combining bond stretch, angle bend, dihedral torsion, (optionally) Urey–Bradley, (optionally) cross terms, and (optionally) concurrence terms:^8,74–79^

2 *U*^FF^_bonded_ = *U*_bond_stretch_ + *U*_angle_bend_ + *U*_dihedral_torsion_ + (*U*_Urey–Bradley_) + (*U*_cross_terms_) + (*U*_concurrence_)

**Fig. 1:**
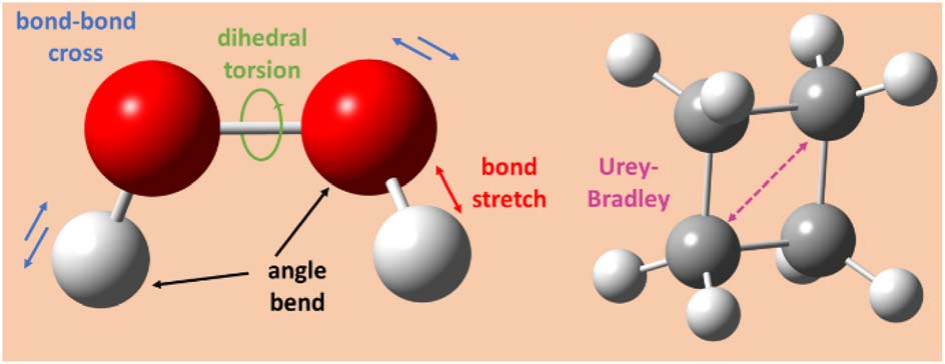
Types of bonded interactions studied in this work.

Fig. 1 illustrates types of bonded interactions studied in this work. Without loss of generality, we can write the bonded interaction potential energy for an individual bonded cluster as a linear combination of flexibility terms^50^3
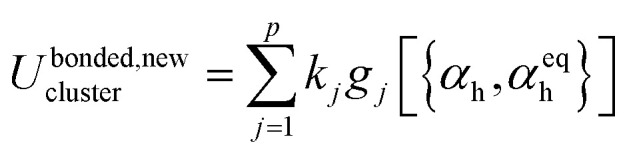
where *k*_*j*_ is the force constant, {*α*_h_} are the corresponding active internal coordinates, and {*α*^eq^_h_} are the equilibrium values of these internal coordinates in the isolated bonded cluster's optimized ground-state geometry. The product *k*_*j*_*g*_*j*_[{*α*_h_,*α*^eq^_h_}] is called a ‘flexibility term’. Since the internal coordinate values are a function of the material's geometry, we can also use the functional form4
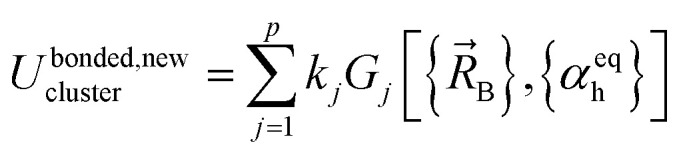
where5
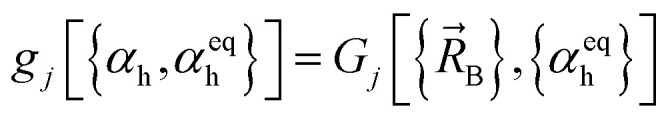


By default, our protocol uses a harmonic bond stretch potential between all first bonded neighbors (*e.g.*, two atoms A and B directly bonded to each other):

6 *U*_harmonic_stretch_[*d*] = *k*_stretch_*G*_harmonic_stretch_[*d*]

7 *G*_harmonic_stretch_[*d*] = ½(*d* − *d*_eq_)^2^

where *k* is the force constant, *d* is the current bond length, and *d*_eq_ is the reference value of the equilibrium bond length in the optimized ground-state geometry. This harmonic bond stretch is simple, popular, and easy to parameterize. If desired, our protocol could be used with other bond stretch potentials including but not limited to Morse,^80^ quartic,^79^ MM3,^8,81^ rigid (*i.e.*, an inflexible bond with fixed length), *etc.* The Morse, quartic, and MM3 bond stretch potentials include some anharmonic terms. The Morse potential approaches a constant value as the two atoms get far apart.

Each 3-membered ring is a triangle whose shape is completely determined by the 3 bond lengths forming the triangle's edges. Since these three bond lengths are included in their corresponding bond stretch potentials, by default our protocol omits angle bends for angles internal to 3-membered rings.

Urey–Bradley (UB) interactions are distance-dependent interactions between second-bonded neighbors.^72^ Each 4-membered ring has 4(3)/2 = 6 internal relative distances, so only 6 internal coordinates are required to describe its shape. These 6 internal coordinates can be constructed by using 4 bond stretches for the ring's 4 edges plus two UB terms for the ring's two diagonals. This is a more compact representation of the internal degrees of freedom than using 4 angle bends plus 4 bond stretches. Accordingly, by default our protocol includes UB terms for the diagonals of 4-membered rings and omits angle bends for angles internal to 4-membered rings. By default, our protocol uses the harmonic stretch potential (eqn (7)) for these UB terms. Although not included by default, our protocol could also include UB terms between additional pairs of second-bonded neighbors.

In a companion article, one of us introduced a new angle-bending potential that has four distinct advantages:^50^

(1) It has a quadratic-like form for small displacements from the equilibrium bond angle over the entire range of possible equilibrium bond angles: 0 < *θ*_eq_ ≤ π.

(2) It has continuous derivatives of all orders for all angle values even at *θ* = π.

(3) As the bond angle approaches zero (*i.e.*, *θ* = 0), the angle-bending potential energy tends towards infinity. This mimics the Pauli repulsion of electrons that energetically prohibits bond angle values from reaching zero.

(4) It has a simple analytic form with only a single adjustable parameter, which is the force constant *k*_angle_.

To the best of our knowledge, no previous angle-bending potential simultaneously has all four of the above features. This new angle-bending potential has the form^50^

8 *U*_Manz_bend_[*θ*] = *k*_angle_*G*_Manz_bend_[*θ*]

9
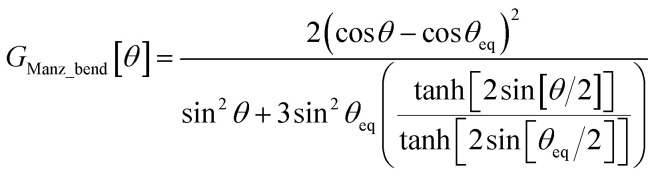


Although it is possible to use other angle-bending model potentials with our SAVESTEPS protocol, the above angle-bending potential is preferable and was used in this work.

One of the key strengths of our SAVESTEPS protocol is a comprehensive yet computationally efficient treatment of dihedral torsions. In a companion article, one of us derived new dihedral-torsion model potentials^50^ that we used in this work. These dihedral-torsion model potentials are described in the next section. Although it is possible to use other dihedral-torsion model potentials with our SAVESTEPS protocol, Manz's new dihedral-torsion model potentials have many compelling advantages.^50^

Our protocol can optionally include various types of cross terms. Some types of cross terms described in the prior literature include bond–bond, bond–bend, bend–bend, bond–torsion, bend–torsion, and others.^8,79,81,82^ In this work, we compared the performance of flexibility models optimized with and without bond–bond cross terms. We used the following model potential for bond–bond cross terms:

10 *U*_bond–bond_[*d*_AB_,*d*_BC_] = *k*_bond–bond_*G*_bond–bond_[*d*_AB_,*d*_BC_]

11 *G*_bond–bond_[*d*_AB_,*d*_BC_] = (*d*_AB_ − *d*^eq^_AB_)(*d*_BC_ − *d*^eq^_BC_)

Cross terms and/or UB terms are sometimes required to match the experimental vibrational spectrum. For example, a carbon dioxide molecule has three elementary vibrational modes: (i) a symmetric stretch at 1333 cm^−1^, (ii) an antisymmetric stretch at 2349 cm^−1^, and (iii) a wag (*i.e.*, angle-bending) mode at 667 cm^−1^ wavenumber.^83^ Here, the symmetric and antisymmetric stretches have frequencies that differ by almost a factor of two. Because a CO_2_ molecule has only three atoms, it does not have any dihedral torsions. Consider a forcefield containing two instances of one type of C–O bond stretch plus one instance of one type of O–C–O angle bend:

12 *U*^model_1^_bonded_ = *U*_bond_[*d*_AB_] + *U*_bond_[*d*_BC_] + *U*_angle_[*θ*_ABC_]

In this case, model_1's Hessian expressed in terms of internal coordinates (*d*_AB_, *d*_BC_, *θ*_ABC_) is13
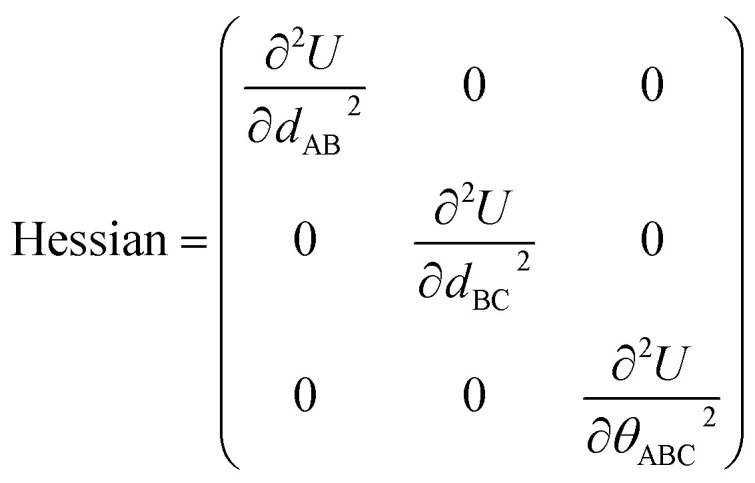
where the off-diagonal terms are zero because both of the following conditions are satisfied: (a) no cross-terms were included in this forcefield and (b) the internal coordinates are independent of each other (*i.e.*, non-redundant). Since this Hessian is diagonal, it immediately follows that these three internal coordinates are the normal vibrational modes. Due to the symmetry of the two C–O bonds in a CO_2_ molecule, we have in this case14
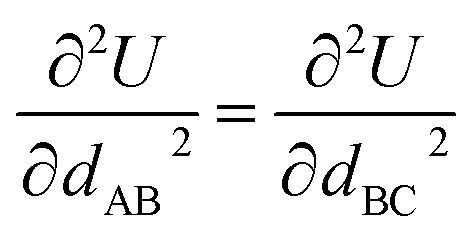


Consequently, two vibrational frequencies are predicted by this forcefield model to be energy degenerate. These two degenerate bond vibrational modes can be linearly combined to yield degenerate symmetric and antisymmetric stretch modes. Because such a forcefield yields symmetric and antisymmetric stretch modes that have the same frequency, it cannot approximate the carbon dioxide molecule's experimental vibrational spectrum. Consequently, a cross term and/or an UB term must be added to this forcefield to resolve this problem. This derived result is general and holds irrespective of the particular functional forms of *U*_bond_[*d*_AB_] and *U*_angle_[*θ*_ABC_].

However, sometimes cross terms and/or UB terms are not required. For example, an isolated water molecule has three elementary vibrational modes: (i) a symmetric stretch at 3657 cm^−1^, (ii) an antisymmetric stretch at 3756 cm^−1^, and (iii) a wag (*i.e.*, angle-bending) mode at 1595 cm^−1^ wavenumber.^83^ The theoretical analysis parallels that for the CO_2_ molecule described above, except that for a H_2_O molecule the symmetric and antisymmetric stretches have frequencies that differ from each other by only ∼3%. Consequently, a forcefield model of the form shown in eqn (12)–(14) above can provide a reasonably good fit to the water molecule's experimental vibrational spectrum.

Many flexible forcefields described in the prior literature include concurrence terms.^6,8,30,82,84,85^ Mathematically, a point of concurrence is where three or more line segments meet at a point. In a material, this corresponds to the situation in which three or more bonds share a common atom. Like cross terms, concurrence terms refine the potential energy expression beyond the basic description provided by bonds, angles, and dihedrals. Consider the ammonia (NH_3_) molecule as an example. At its equilibrium ground-state geometry, the three H–N–H angles in NH_3_ sum to a value smaller than 2π; however, these three angles sum to exactly 2π in the planar transition state for the inversion reaction. Because these angles have a different value in the transition state than in the ground state structure of ammonia, using an angle-bending potential by itself already gives a positive inversion barrier without including a special concurrence term in the forcefield. However, it may be desirable to include a special concurrence term in the forcefield to fine-tune the inversion barrier's value. As another example, consider a planar molecule such as benzene. Suppose that atom C(1) is bound to atoms C(2), C(3) and H. When these four atoms are in the same plane, the three angles C(2)–C(1)–C(3), C(2)–C(1)–H, and C(3)–C(1)–H sum to π. When atom C(1) moves out of the plane defined by atoms C(2), C(3) and H, those three angles sum to less than π. Accordingly, using an angle-bending potential by itself already gives an out-of-plane energy increase for benzene without including a special concurrence term in the forcefield. However, it may be desirable to include a special concurrence term in the forcefield to fine-tune the potential energy. In the prior literature, concurrence terms are typically constructed using one of the following chemical descriptors: out-of-plane distance, out-of-plane angle, and/or improper-dihedral^6,8,30,84,85^ (in this work, the standalone term ‘dihedral’ always refers to a proper dihedral, while ‘improper-dihedral’ will always be explicitly used for improper-dihedrals).

Since adding more terms (*e.g.*, cross terms, concurrence terms, anharmonic terms, *etc.*) increases the forcefield's complexity, a key question is how to identify which particular terms substantially improve the forcefield's accuracy and which are insignificant. Our protocol includes two major innovations to address this question. As described in Section 8.7, our protocol computes statistics (*e.g.*, *R*-squared and RMSE) for individual atoms in a material to identify how well the flexibility model performs for different atoms in the material. This highlights particular atoms (if any) for which the flexibility model needs to be improved. Our protocol also incorporates several embedded feature selection techniques. During least-squares optimization of the force constants, our protocol uses the LASSO method to identify which forcefield terms are necessary and which are unnecessary for constructing the flexibility model. Our protocol automatically generates a concise flexibility model that identifies and includes only those terms that are valuable. In this work, we used this approach to identify and select which particular bond–bond cross interactions are valuable. Our protocol could also use this approach for other types of cross terms, concurrence terms, anharmonic terms, *etc.*

### 2.2 Dihedral torsion potentials

The dihedral torsion potential has five major cases. Case 1: the dihedral type is classified as rotatable, and one or both of the included equilibrium bond angles is ≥130°. In this case, the following angle-damped-dihedral-torsion (ADDT) potential is used which has up to seven modes:^50^

15 *U*^ADDT^_mode_*m*_[*θ*_ABC_,*θ*_BCD_,*ϕ*_ABCD_] = *k*^*m*^_*ϕ*_*G*^ADDT^_mode_m_[*θ*_ABC_,*θ*_BCD_,*ϕ*_ABCD_]

16
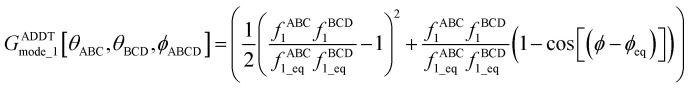
17
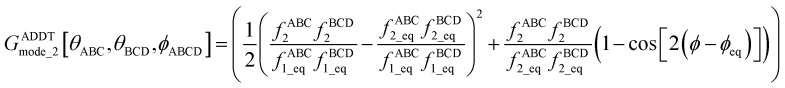
18
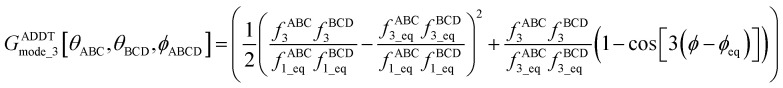
19
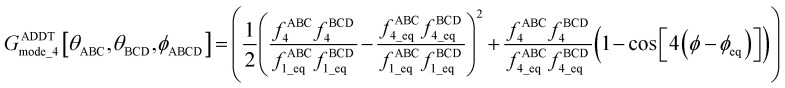
20
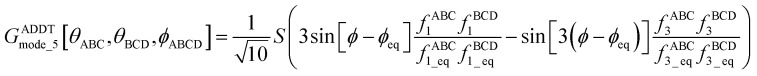
21
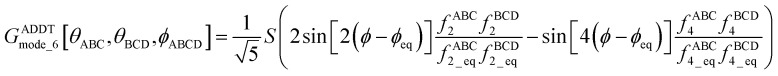
22
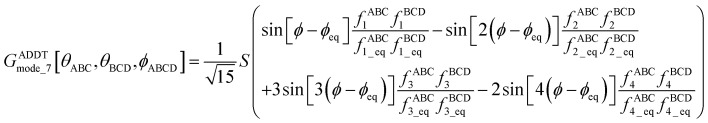


After dihedral mode smart selection (see Section 7.1), this yields23
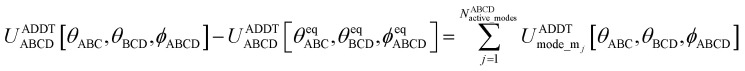
where *N*^ABCD^_active_modes_ is the number of active modes for dihedral ABCD. The angle-damping factors are defined as follows:24
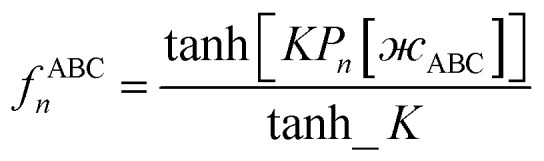
25
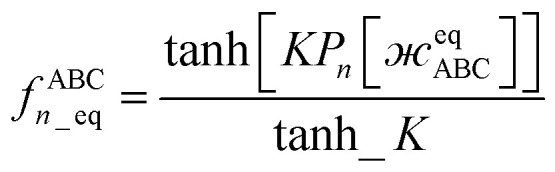
where26
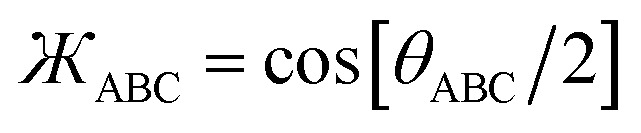
27
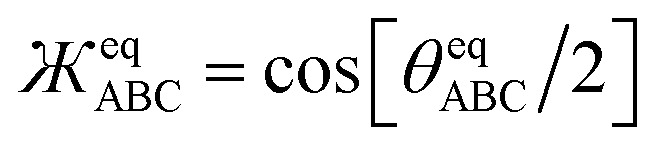
28
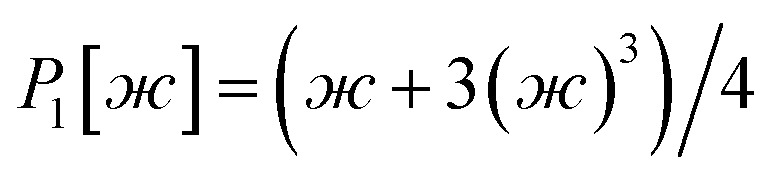
29
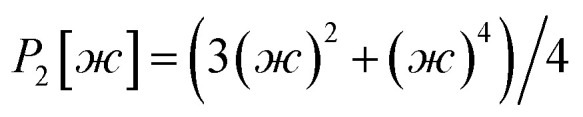
30
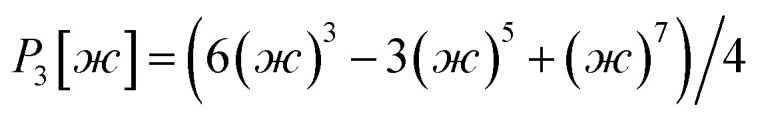
31
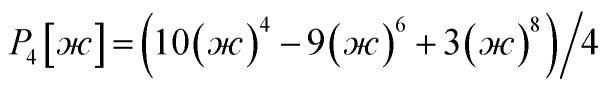

32 *K* = 2.815891616117388…

33 tanh_*K* = tanh[*K*] = 0.992861208914406

Case 2: the dihedral type is classified as rotatable, and both of the included equilibrium bond angles are <130°. In this case, the following constant-amplitude-dihedral-torsion (CADT) potential is used which has up to seven modes:^50^

34 *U*^CADT^_mode_*m*_[*ϕ*_ABCD_] = *k*_*ϕ*_^*m*^*G*^CADT^_mode_*m*_[*ϕ*_ABCD_]

35 *G*^CADT^_mode_*m*≤4_[*ϕ*_ABCD_] = 1 − cos[*m*(*ϕ* − *ϕ*_eq_)]

36
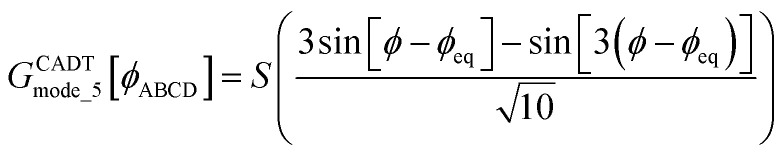
37
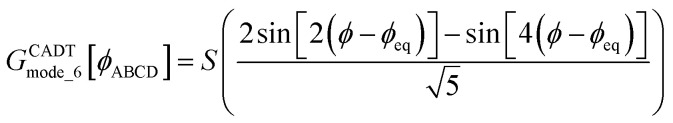
38
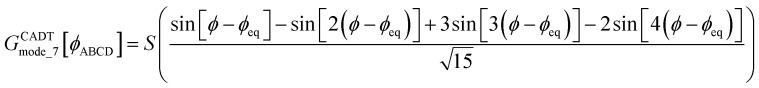


After dihedral mode smart selection (see Section 7.1), this yields39
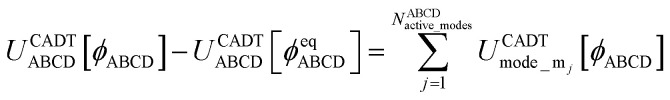
where *N*^ABCD^_active_modes_ is the number of active modes for dihedral ABCD.

Case 3: the dihedral type is classified as non-rotatable or hindered, and one or both of the included equilibrium bond angles is ≥130°. In this case, the dihedral has a restricted range of motion. For small dihedral displacements a harmonic-like potential is sufficient, and this can be approximated by a single torsion mode. Since one of the included equilibrium bond angles is ≥130°, we still need to include the angle-damping factors. Consequently, for Case 3 we used only mode 1 from the ADDT potential:

40 *U*^ADDT_1^_ABCD_[*θ*_ABC_,*θ*_BCD_,*ϕ*_ABCD_] − *U*^ADDT_1^_ABCD_[*θ*^eq^_ABC_,*θ*^eq^_BCD_,*ϕ*^eq^_ABCD_] = *k*_*ϕ*_^1^*G*^ADDT^_mode_1_[*θ*_ABC_,*θ*_BCD_,*ϕ*_ABCD_]

Case 4: the dihedral type is classified as non-rotatable or hindered, and both of the included equilibrium bond angles are <130°. In this case, the dihedral has a restricted range of motion. For small dihedral displacements a harmonic-like potential is sufficient, and this can be approximated by a single torsion mode. Since both of the included equilibrium bond angles are <130°, the constant torsion amplitude approximation can be used. Consequently, for Case 4 we used only mode 1 from the CADT potential:

41 *U*^CADT_1^_ABCD_[*ϕ*_ABCD_] − *U*^CADT_1^_ABCD_[*ϕ*^eq^_ABCD_] = *k*_*ϕ*_^1^*G*^CADT^_mode_1_[*ϕ*_ABCD_]

For Cases 3 and 4, the use of a single torsion mode is an approximation that holds only if the dihedral's displacements are small. If a non-rotatable or hindered dihedral exhibits large displacements (during thermal vibrations) away from the dihedral's equilibrium value, then it could become necessary to add more torsion modes to this dihedral's potential model (we did not perform such an addition for any MOFs studied in this work). Note that in eqn (15), eqn (34), eqn (40) and eqn (41) the raised '*m*' or '1' is a superscript index not an exponent.

Case 5: in this case, one or both of the equilibrium bond angles in the dihedral is linear (or close to linear). ‘Close to linear’ means that π − *θ*^eq^_ABC_ < *ε* or π − *θ*^eq^_BCD_ < *ε*, where ε is a tolerance (*e.g.*, 0.03 radians). We reiterate that this case applies when one or both of the equilibrium bond angle values is linear irrespective of the instantaneous bond angle value. For these ‘linear dihedrals’, Manz's ADDT linear model potential^50^ (which contained two torsion modes) was used as described in ESI Section S1.† After dihedral pruning, only 5 of the 116 MOFs in our study had linear dihedrals. For comparison purposes, we also completely reoptimized the flexibility parameterization for these 5 MOFs using an analogous flexibility model except the ADDT linear model potential was omitted. We found that the validation dataset *R*-squared and RMSE (eV Å^−1^) values for these five MOFs changed little (*e.g.*, in third or fourth significant digits) when the linear dihedrals were omitted from the flexibility model. However, for completeness in the remainder of this article all of the results for these five MOFs included our ADDT linear model potential. The ADDT linear model potential was not used for the other 111 MOFs that had no after-pruning linear dihedrals.

## 3. Overview of the SAVESTEPS approach

**Fig. 2:**
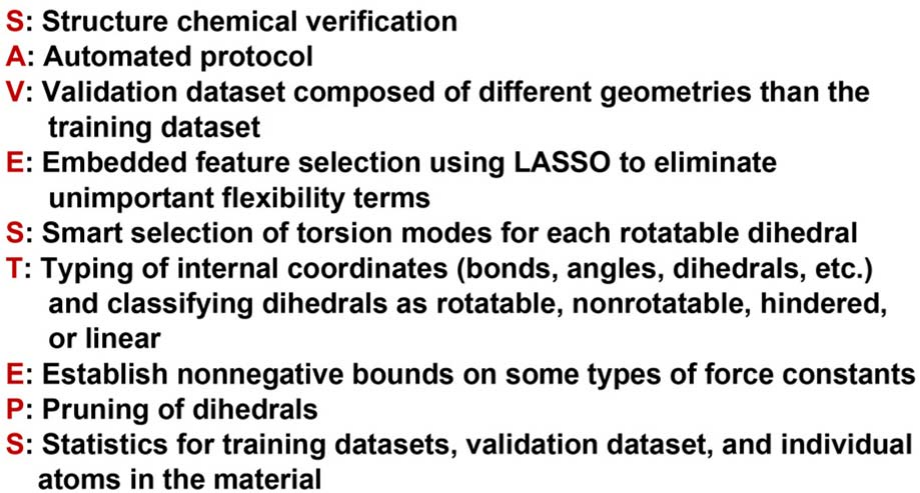
The SAVESTEPS acronym.

As shown in Fig. 2, SAVESTEPS is an acronym constructed from some of the major features of our approach. Our approach excels particularly at: chemical structure verification; extensive automation; state-of-the-art typing of atoms, bonds, angles, and dihedrals; dihedral pruning that preserves symmetry equivalency; classification of each dihedral type as rotatable, nonrotatable, hindered, or linear; smart selection of torsion modes for each rotatable dihedral type; state-of-the-art angle-bending and dihedral-torsion model potentials; model potentials having improved numeric stability even for linear bond angles; the ability to use linear regression instead of requiring nonlinear regression to optimize values of the flexibility parameters; embedded feature selection using the LASSO method to identify and zero out unimportant forcefield terms; thorough training and validation sets; non-negative bounds on force constants for bond stretches, Urey–Bradley stretches, angle bends, single-mode torsions, and ADDT linear torsion modes; insightful statistics both for the whole material and for individual atoms in the material; and excellent computational efficiency. No previously published approach to optimize flexibility parameters for classical forcefields has the complete set of these features.

**Fig. 3:**
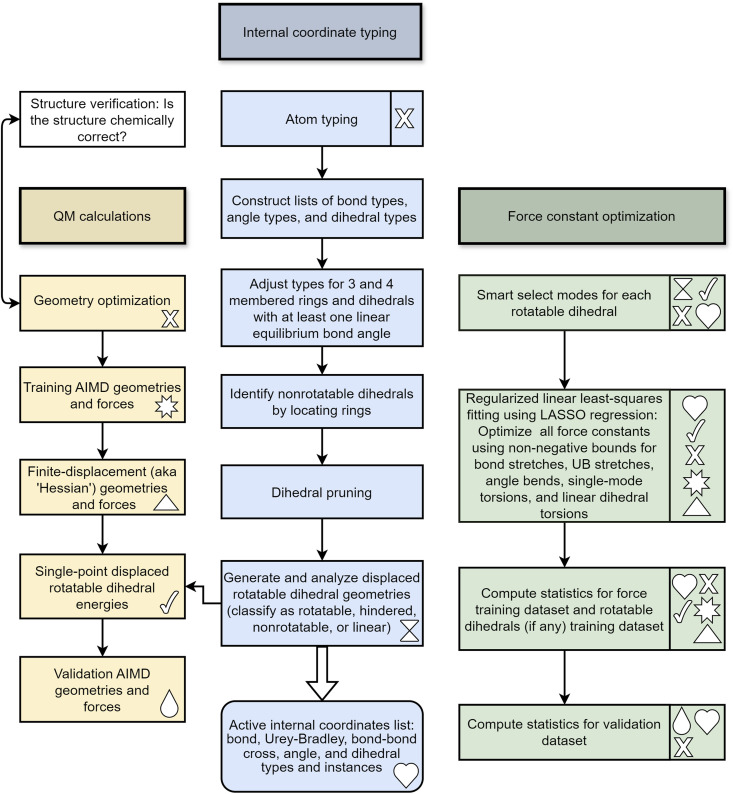
Flowchart summarizing our automated protocol to construct flexibility parameters for classical forcefields. Steps performing quantum chemistry calculations are shaded tan. Steps involving the typing of atoms, bonds, angles, or dihedrals are shaded blue. Steps involving linear regression and statistical performance are shaded green. Icons are used to represent particular data that is generated (no separating line) or used (separating line) in a particular step: optimized geometry (*X*), training AIMD geometries and forces (eight-pointed star), finite-displacement (aka ‘Hessian’) geometries and forces (triangle), classification of each dihedral as rotatable, hindered, nonrotatable, or linear (hourglass), displaced rotatable dihedral single-point energies (checkmark), active internal coordinate list (heart), and validation AIMD geometries and forces (raindrop).

Fig. 3 is a flowchart summarizing our automated protocol to construct flexibility parameters for classical forcefields. The key steps in this protocol are:

Step # 1: the starting chemical structure and geometry are checked for misbonded atoms and other chemical errors. Structures with chemical errors are not accepted.

Step # 2: a quantum chemistry calculation is performed to find the material's optimized ground-state geometry. For periodic materials, the lattice constants defining the unit cell's size and shape are preferably held fixed at the experimental values (if known) while the atom-in-material positions are quantum-mechanically relaxed (if experimentally-measured lattice vectors are not available or not reliable, then quantum-mechanically-computed lattice vectors can be used to determine the unit cell's size and shape). If the material's experimental geometry is available, the quantum-mechanically-computed geometry is compared to the experimental geometry to ensure they match within a reasonable tolerance. The optimized structure is rechecked for misbonded atoms and other chemical errors. Structures with chemical errors are not accepted.

Step # 3: for the quantum-mechanically-computed optimized ground-state geometry, typing is performed to generate lists of atom types and internal coordinate types (*e.g.*, stretch types, angle types, and dihedral types). To be classified as the same type, two specific occurrences of an internal coordinate must satisfy all three conditions: (i) They must have the same combination and order of atom types. (ii) The internal coordinate's absolute value for the second occurrence must match that of the first occurrence within a chosen tolerance. (iii) Two angles of the same type must contain the same combination and order of bond types. Two dihedrals of the same type must contain the same combination and order of angle types.

Step # 4: some adjustments are made to the active internal coordinate types list: (1) Urey–Bradley stretches are added for the two diagonals of each 4-membered ring. (2) Angles in 3- and 4-membered rings are flagged in the internal coordinates list and not used in the angle-bending potential. (3) Dihedrals containing angles from 3- and 4-membered rings are removed from the active internal coordinates list. (4) Dihedral ABCD is classified as ‘linear’ if either π − *θ*^eq^_ABC_ < *ε* or π − *θ*^eq^_BCD_ < *ε*, where ε is a tolerance (*e.g.*, 0.03 radians).

Step # 5: a dihedral instance is classified as non-rotatable if its middle bond is part of a bonded ring; otherwise, it is classified as rotatable. A dihedral type is classified as non-rotatable iff any one or more of its instances is non-rotatable.

Step # 6: if two or more different dihedral types pass through the same middle bond type, the lists of middle bond instances for these two dihedral types are compared to see if they are identical. Repetitions and ordering do not matter in this comparison. For example, the list of middle bond instances {a,b,c,a,b} is considered equivalent to {a,c,b} in this comparison. If two or more different dihedral types have equivalent middle bond instances (where repetitions and ordering do not matter in this comparison), only one of these dihedral types is retained in the active internal coordinates list. Since symmetry-equivalent dihedral instances are grouped into the same dihedral type, this dihedral pruning preserves the symmetry equivalency while reducing redundancy.

Step # 7: a set of quantum chemistry calculations is performed corresponding to finite displacements along each independent atom translation mode (aka finite-displacement ‘Hessian’ geometries) plus a set of AIMD-generated geometries that together with the optimized geometry comprise the force training set (as shown by the computational tests in ESI Section S5,† including AIMD-generated geometries in the force training dataset improves the flexibility model's accuracy). A completely independent set of AIMD geometries is generated to make the validation set.

Step # 8: for a rotatable dihedral type, one dihedral instance is randomly selected and uniformly displaced (*e.g.*, in 10° increments) over its full range to generate a set of dihedral-displaced geometries. Each such dihedral-displaced geometry is then analyzed to identify its atom types. If the atom type for each atom in each dihedral-displaced geometry matches that for the corresponding atom in the optimized ground-state geometry, the dihedral type retains its rotatable classification; otherwise, it is reclassified as a ‘hindered dihedral type’. A hindered dihedral corresponds to the situation in which its rotation over some values changes the material's bond connectivity and hence changes the atom type of one or more atoms. This process of generating and analyzing dihedral-displaced geometries is performed sequentially one rotatable dihedral type at a time (always starting from the optimized ground-state geometry) until all rotatable dihedral types in the active internal coordinate list have been analyzed and classified as ‘rotatable’ or ‘hindered’.

Step # 9: for all dihedral types that retained ‘rotatable’ classification, single-point quantum chemistry calculations are performed for their dihedral-displaced geometries that were generated in Step # 8 above. This yields a quantum-mechanically-computed total energy for each such dihedral-displaced geometry.

Step # 10: for each rotatable dihedral type, the energy curve for its dihedral-displaced geometries is projected onto a set of seven orthonormal torsion modes (as described in Sections 2, 7.1, and 8.5) to identify and smart select the particular torsion modes that contribute significantly to this energy curve.

Step # 11: although there is some leeway in how to construct the potential model, the following describes a preferred choice. Manz's^50^ angle-bending potential is preferably used for each of the active bond angles. For non-rotatable, hindered, and rotatable dihedrals, a CADT model is preferably used iff both contained bond angles are less than 130°; otherwise, an ADDT model is preferably used. Each non-rotatable or hindered dihedral type is normally described by a torsion potential containing a single mode (*e.g.*, mode 1); however, if desired another torsion mode could be added to describe anharmonicity. Each rotatable dihedral type is described by a torsion potential containing the smart selected modes. Dihedrals for which at least one of the contained equilibrium bond angles is linear are preferably modeled using the ADDT linear torsion modes. Either a simple harmonic potential or a more sophisticated potential could be used for the bond and Urey–Bradley stretches. Where desired, cross terms, concurrence terms, and/or other terms can be optionally included.

Step # 12: the force-constant values are optimized *via* regularized linear least-squares fitting. Non-negative bounds are placed on the force constants for bond stretches, Urey–Bradley stretches, angle bends, lone-mode torsions, and ADDT linear torsion modes. No bounds are placed on force constants for bond–bond cross terms and multi-mode rotatable torsions. LASSO regression is used to automatically identify and zero out the force constants for unnecessary flexibility terms. The training dataset includes:

(a) A full dihedral scan energy curve for each rotatable dihedral type (if any are present in the material).

(b) Quantum-mechanically-computed atom-in-material forces for the material's optimized ground-state geometry. These forces are zero within a convergence tolerance.

(c) Quantum-mechanically-computed atom-in-material forces for finite-displacement ‘Hessian’ geometries that sample *x*, *y*, *z* displacements for each atom in the material.

(d) Quantum-mechanically-computed atom-in-material forces for AIMD-generated geometries. Geometries are included for at least ten independent AIMD runs.

Step # 13: using the optimized force-constant values from Step # 12 above, the *R*-squared and RMSE values are computed for the validation set of geometries. This tests how well the flexible forcefield model reproduces atom-in-material forces across a new set of AIMD-generated geometries that were not used in training the model, as well as in the optimized ground-state geometry. *R*-Squared and RMSE values are also computed and printed for each individual atom in the material to identify particular atoms (if any) for which the forcefield needs to be improved.

## 4. Crystal geometry verification

In 2014, Chung *et al.*^86^ published a Computation Ready Experimental (CoRE) MOF database that was created by first screening the Cambridge Structural Database (CSD^87^) to find MOFs and then partially cleaning these structures. Their cleaning process aimed to remove solvent molecules and other small adsorbates from the MOF's pores, keep charge-balancing ions, and fix or eliminate any structures with disordered atoms and partial occupancies. Also, some of the structures had missing hydrogen atoms added to them. This cleaning procedure was not perfect, therefore some structures still had problems.^57,88–90^

CoRE MOF 2019 is an updated version of the database containing thousands more structures than the 2014 version.^91^ Structures with only free solvent molecules removed are found in the free solvent removed (FSR) set. Structures in the all solvent removed (ASR) set have both bound and free solvent molecules eliminated. These modified structures are designated as the FSR-public and ASR-public datasets.^91^ Chung *et al.* reported the original CSD refcode as the relevant structure in instances when the FSR or ASR processes did not result in any molecules being removed or any other modifications to the structure; these are designated as the FSR_CSD and ASR_CSD datasets.^91^

In 2017, Moghadam *et al.*^92^ constructed a CSD MOF subset using seven “look-for-MOF” criteria to locate and extract MOF materials from the CSD database. Moreover, a variety of computational techniques were developed and employed to first exclude the solvent molecules from the CSD MOF subset and create a CSD non-disordered MOF subset.^92^

To identify structures with isolated or mis-bonded atoms, Chen and Manz^93^ screened the 2019 CoRE MOF database for the following: (i) atoms not directly bonded to any neighboring atoms (aka, ‘isolated’ atoms), (ii) atoms that are too close together (aka, overlapping atoms), (iii) misplaced hydrogen atoms, (iv) under-bonded carbon atoms (this could result from missing hydrogen atoms) and (v) over-bonded carbon atoms. MOFs that passed this screening procedure were placed into accepted_FSR (for free solvent removed) and/or accepted_ASR (for all solvent removed) subsets of the CoRE MOF database.

In 2021, Daglar *et al.*^94^ showed that a considerable number of MOFs are reported with the same refcode but different reduced chemical compositions in the 2019 CoRE MOF database *versus* the CSD non-disordered MOF subset. They claimed that 2434 MOFs had the same reduced chemical formula in both databases; these are known as chemical formula matched-MOFs (CFM-MOFs).^94^ 1109 MOFs had different reduced chemical formulas in one database compared to the other database; these are known as chemical formula unmatched-MOFs (CFU-MOFs).^94^ They demonstrated how the database used affects the simulation results of 1109 CFU-MOFs by yielding significantly different gas uptakes.^94^

In 2021, Rosen *et al.*^95,96^ used a high-throughput periodic DFT methodology using the PBE-D3(BJ) data initially to create the QMOF database of quantum-chemical characteristics for MOFs. They accounted for the list of materials classified as MOFs from the 2019 CoRE MOF database as well as the CSD non-disordered MOF subset. They first filtered problematic MOFs that had missing H atoms, fractional occupancies, missing framework atoms, lone (*i.e.*, unbonded) atoms, overlapping atoms, an insufficient number of charge-balancing ions, and other structural problems.^95,96^ Afterwards, they performed DFT calculations on MOFs that passed this screening process. The QMOF database currently includes more than 20 000 experimentally synthesized MOFs with publicly available parameters determined by DFT such as optimized geometries, density of states, and DDEC6 population analysis results (*e.g.*, net atomic charges,^58–60^ atomic spin moments,^58,97^ and bond orders^58,98^).^95,96^

Taken together, the above studies cast some doubts on the quality of available databases containing partly cleaned experimentally-derived MOF structures. What happens if a particular MOF has different chemical structures in different partly cleaned experimentally-derived MOF databases? In such a case, how does one decide which (if any) of the reported structures for the MOF is chemically reasonable? An obvious way to mitigate this issue is to use a subset of MOFs that have the same reported chemical structure in several partly cleaned experimentally-derived MOF databases. Because these various databases used different cleaning procedures, a MOF that has exactly the same ‘cleaned’ chemical structure in several of these databases is more likely to be trustworthy. For example, a MOF missing hydrogen atom(s) might pass through one database's cleaning procedures but be rejected by a different database's cleaning procedures. If one or more of the databases added in missing hydrogen atoms, their placement is suspect if two databases do not agree on the hydrogen atom positions. As another example, a particular adsorbed solvent molecule in a particular MOF might be removed by one database's cleaning procedures but not removed by a different database's cleaning procedures. These disagreeing structures will be rejected if we select a subset of MOFs that have the same chemical structure in several partly cleaned experimentally-derived MOF databases.

**Fig. 4:**
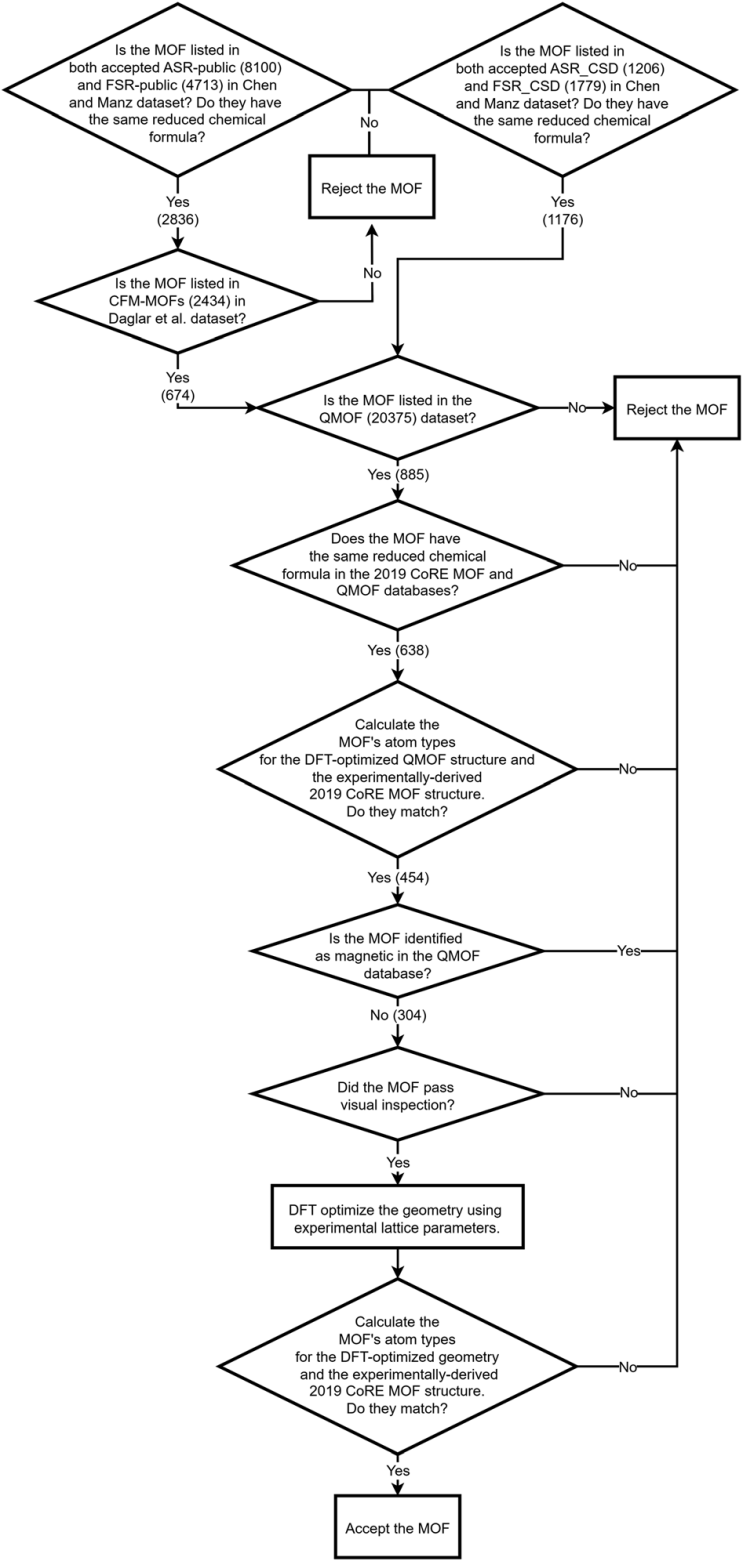
Crystal geometry verification procedure workflow.

As shown in Fig. 4, we applied a crystal geometry verification procedure to select MOFs with reliable chemical structures. In Fig. 4, each number in parentheses is the number of MOF structures satisfying that criterion. First, we checked whether a MOF was listed in and had the same reduced chemical formula in Chen and Manz's accepted_FSR and accepted_ASR datasets. Selecting MOFs that have the same chemical structure after free solvent removal as after all solvent removal reduces ambiguity in the solvent removal process. Moreover, these accepted structures had passed through Chen and Manz's screening process to identify misbonded and lone atoms.^93^ For those MOFs in the ASR and FSR public datasets, we then checked to see if they were in Daglar *et al.*'s^94^ CFM-MOF dataset which ensures the MOF's reduced chemical formula is the same in the CoRE MOF and CSD non-disordered MOF databases.

For each MOF (whether in the public or CSD portion of the ASR and FSR databases) that passed the above screening criteria, we next checked whether it was listed in the QMOF database and had the same chemical formula in the QMOF and 2019 CoRE MOF databases. Because the QMOF database applied some different cleaning procedures than the 2019 CoRE MOF database, this screening criterion selects MOFs whose chemical structure is more robust because it passed through different cleaning procedures. Then we performed atom typing on the DFT-optimized QMOF structure and the experimentally-derived 2019 CoRE MOF structure using Chen and Manz's^57^ atom-typing procedure. This criterion ensured that the MOF's structure did not drastically change during DFT geometry optimization. For example, this screening criterion rejects a MOF is that is unstable after adsorbed solvent is removed from its pores and consequently changes bond connectivity during DFT geometry optimization.

Because magnetic MOFs present greater computational challenges to converge each DFT calculation to the correct magnetic ordering, we decided for simplicity to restrict the current study to non-magnetic MOFs. We emphasize that the SAVESTEPS protocol introduced in this manuscript applies also to magnetic materials, but it is more work since care must be exercised to ensure each quantum-chemistry calculation converges to its low-energy magnetic ordering.

We then performed a visual inspection of each MOF using a chemical visualization program. This step serves as a sanity check by ensuring the MOF's structure has been viewed by a human expert. The purpose of this step is to remove any MOFs that appear to have chemically unstable structures and/or undesirable chemical linkages. Rejection or acceptance at this visual inspection stage is subjective according to the human expert's judgement and experience. Reasons for rejecting MOFs at this step included (but was not limited to) the following. Structures containing rings of 5 to 8 atoms containing four or more nitrogen atoms within the same ring (*e.g.*, tetrazole rings) were rejected, because these may potentially thermally decompose releasing N_2_ gas. Structures containing high concentrations of N–N linkages were also rejected, because these may potentially thermally decompose releasing N_2_ gas. Structures that contained free or weakly bound ions that may potentially dissociate were also rejected. Examples included carbonate ions ([CO_3_]^2−^), weakly bound OH^−^ ions, weakly bound Cl^−^ ions, bicarbonate ions ([HCO_3_]^−^), nitrate ions ([NO_3_]^−^), and sulfate ions ([SO_4_]^2−^). Some structures containing high concentrations of Cu–C–N–Cu linkages were rejected. This visual inspection also checked for misbonded atoms (*e.g.*, overlapping atoms, misplaced hydrogen atoms, missing hydrogen atoms, *etc.*), but we did not find any misbonded atoms at this point. This indicates that the earlier screening for misbonded atoms was reliable.

After visual inspection, we performed DFT geometry optimization on each MOF holding the unit cell's size and shape rigid at the experimental values. Afterwards, we recalculated the MOF's atom types (using Chen and Manz's^57^ procedure) and checked that these were the same as atom types extracted from the experimentally-derived 2019 CoRE MOF structure. This step rejected any MOF whose bond connectivity changed during DFT geometry optimization.

Our goal was to select at least 100 MOFs for flexibility parameters optimization, so after identifying 116 MOFs that passed all of the above criteria, we stopped searching. Likely, there are additional MOFs that would have passed all of the above criteria, but we did not continue looking for them, because our goal was already reached.

## 5. Bond, angle, and dihedral typing

### 5.1 Overview and flow diagram

Previously, Chen and Manz^57^ worked on the large-scale computation of atom types and forcefield precursors. In contrast to atom types based on only first neighbors, they demonstrated that atom types based on first and second neighbors can accurately capture the chemical environment.^57^ Specifically, they showed that the standard deviation of calculated forcefield precursors was significantly high for atoms with similar first-neighbor environments but comparatively small for atoms with similar first-and-second-neighbor environments.^57^ For instance, the atom type 6[1-(0),1-(0),1-(0),6-(1,1,8)] designates a central carbon atom with four first neighbors (H, H, H, and C), where each of the first-neighbor H atoms is not directly linked to any second neighbors and the first-neighbor C atom is directly bonded to H, H, and O in addition to the central atom.^57^ We used this method to compute each atom's type in a MOF's geometry.

**Fig. 5:**
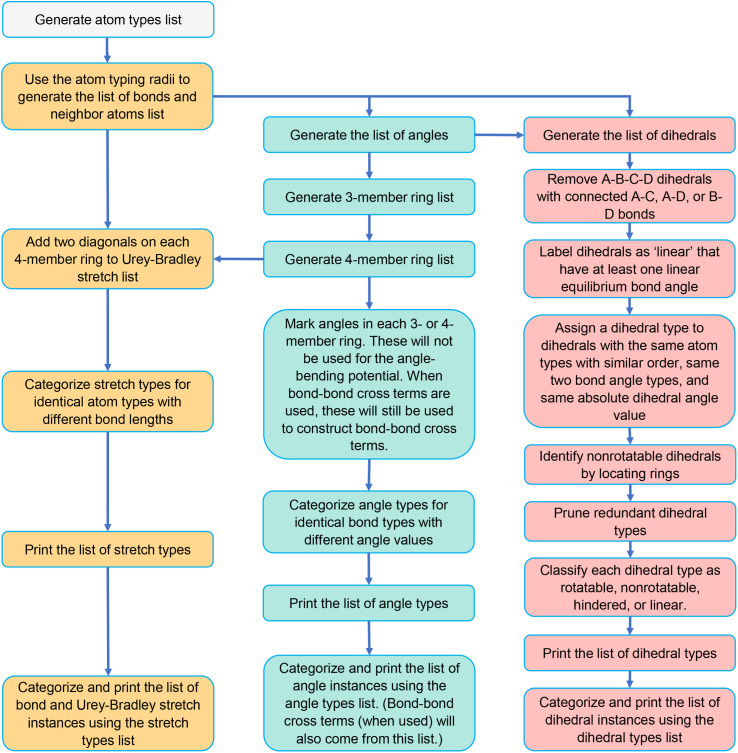
Flowchart for generating lists of stretch types and instances, angle types and instances, and dihedral types and instances.

In this study, we wrote Python codes to first identify all the existing bonds, angles, and dihedrals in any given MOF geometry. Some of these will be placed on an ‘active list’ that will be used to construct the flexibility model. The lists of active bond, angle, and dihedral types and instances are generated using the protocols described in Sections 5.2, 5.3, and 5.4, respectively. Fig. 5 summarizes the workflow to generate these lists of active internal coordinate types and instances. This information is essential to building a potential energy model describing a particular MOF's flexibility; that is, to construct 
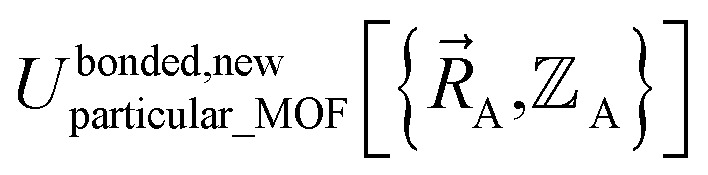
 that can be used in a flexible forcefield (see eqn (1)).

Our SAVESTEPS protocol requires that the unit cell used is large enough that each atom A is directly bonded to only one image of a particular first-neighbor atom B. This is a feature not a bug. Consider a material such as NaCl crystal that has a small primitive unit cell. If we define the unit cell to contain only one Na and one Cl atom, then during AIMD simulations all Cl atoms will move in unison, because they are periodic images of the same reference Cl atom. Because there is no such thing as a Cl–Cl vibrational stretch when using such a small unit cell, it follows that using such a small unit cell overly restricts the atom-in-material motions. To resolve this problem, a larger unit cell must be used such that each atom A is directly bonded to only one image of a particular first-neighbor atom B. For NaCl crystal, we could accomplish this by creating a supercell that contains many Na and Cl atoms, and then use this supercell as our periodic unit cell during all of the quantum chemistry calculations and subsequent flexibility model development. This enables Cl–Cl vibrational stretches to exist and be included in the parameterized flexibility model for NaCl crystal.

### 5.2 Generating the list of stretches to use in the forcefield

Iff the distance between two atoms was less than or equal to the sum of their atom typing radii, we classified them as directly bonded to each other.^57^ For each atom A in the reference unit cell, we checked for its bonds to any other atom images {(B,0,0,0)} in the reference unit cell and also for its bonds to any atom images {(B,L_1_,L_2_,L_3_)} in the 26 unit cells surrounding the reference unit cell.

**Fig. 6:**
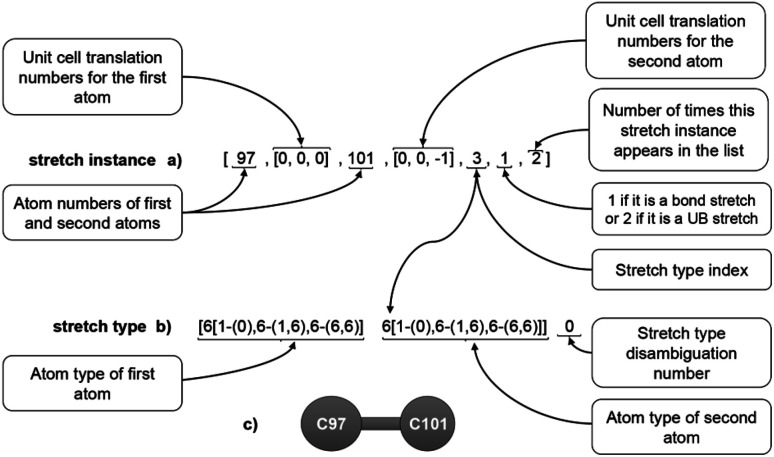
Example format for a stretch instance (a), stretch type (b), and ball-and-stick illustration (c).

All of these bond instances were added to the list of stretch instances. To the list of stretch instances, we also added Urey–Bradley (UB) second-neighbor stretch instances for diagonals of 4-membered rings. Fig. 6 illustrates the information stored in each stretch instance. Each stretch instance stored the two atom numbers, the unit cell translation indices for each atom, the stretch type index, whether the stretch is a bond stretch or UB stretch, and the number of times this stretch instance appears in the list. Within a stretch instance, the two atoms are ordered such that their atom types are in alphabetical order; this makes it easier for the code to lookup stretch instances of the same stretch type.

The last number (*i.e.*, the number of times this stretch instance appears in the list) is important to avoid double-counting when computing the potential energy (this number will be either 1 or 2). For example, a stretch instance of the form [A, (0,0,0), B, (−1,0,0), …, 2] will appear again in the list as [A, (1,0,0), B, (0,0,0), …, 2]. Specifically, if there are N_duplicates_ duplicate instances of the same bond stretch instance in the list, then the factor of (1/*N*_duplicates_) will be applied to each duplicate when computing the potential energy, so that the potential energy for this instance is counted exactly *N*_duplicates_(1/*N*_duplicates_) = 1 time.

Whether or not to include some translationally displaced duplicate instances in the list is a software design choice. It is possible to remove the duplicate instances from the list, and this would avoid having to use the *N*_duplicates_ variable. Whether it is easier to include or exclude the translationally displaced duplicate instances has to do with how the files are read, searched, and processed; however, the end results are not changed in any way as long as the software code is correctly written to avoid double-counting. We found it easier to include those translationally displaced duplicate instances and then introduce a weighting factor to avoid double-counting. This applies not only to the stretch instances described in this section, but also to the dihedral instances described in Section 5.4 below.

Two stretch instances were classified into the same stretch type iff they had the same combination of atom types and their equilibrium lengths differed by less than a chosen tolerance. In this work, the first instance of each stretch type was chosen as a reference and another instance containing the same combination of atom types was added to this same stretch type iff its equilibrium length differed by less than 1% of the equilibrium length of the first instance (the reference) in this stretch type. We found this typing criterion that includes equilibrium value similarity is necessary to achieve good performance of the parameterized forcefield. If this criterion did not pass, the new instance was placed into a new stretch type instead of being added to the existing stretch type. As shown in Fig. 6, multiple stretch types that contain the same combination of atom types are distinguished by the ‘stretch type disambiguation number’.

**Fig. 7:**
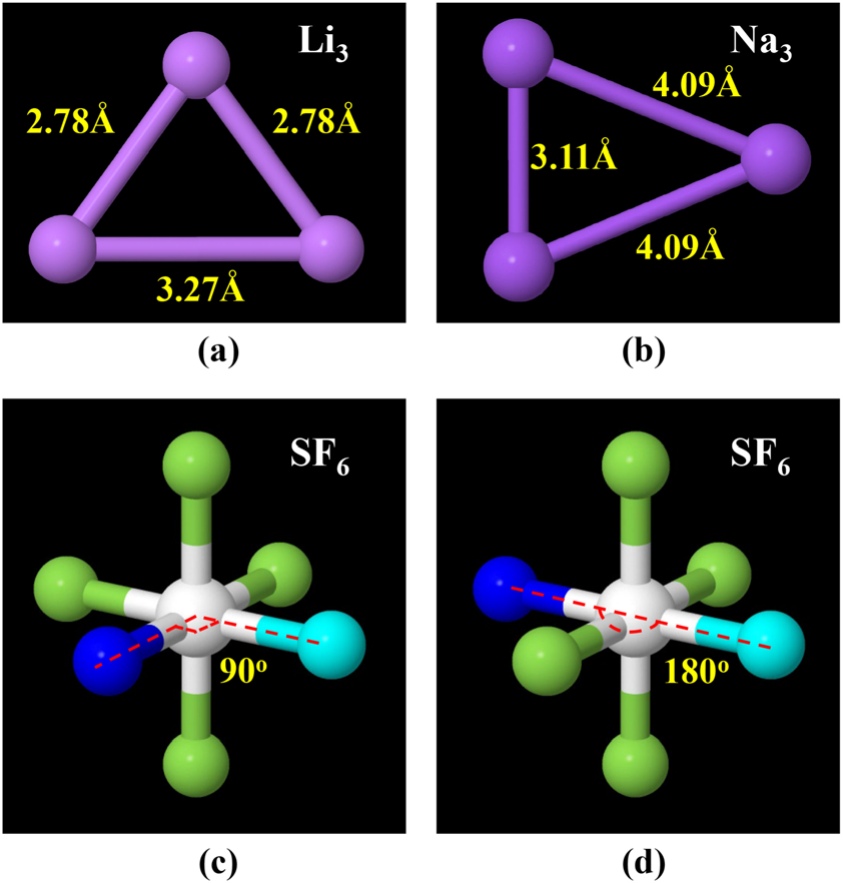
Panels (a) and (b): illustration of bonds comprised of the same order of atom types but having dramatically different equilibrium bond lengths. Shown here are the PBE (ref. 99) + D3 (ref. 100)/aug-cc-pvtz optimized geometries (which we computed using Gaussian software^101^) of Li_3_ and Na_3_ clusters that exhibit Jahn–Teller distortion. Panels (c) and (d): illustration of angles comprised of the same order of atom types, and comprised of the same order of bond types, but having dramatically different equilibrium angle values. This proves that defining unique angle types cannot be based solely on the underlying atom types and bond types but also must consider the angle's equilibrium value. Shown here is a ball and stick model of sulfur hexafluoride (SF_6_). Selected angles are highlighted using navy as the color of the first atom, white as the color of the center atom, and cyan as the color of the last atom.

Fig. 7 shows examples of bonds comprised of the same order of atom types but having dramatically different equilibrium bond lengths. Both the Li_3_ and Na_3_ molecules exhibit Jahn–Teller distortion in which one of the three bonds has a substantially different equilibrium length than the other two bonds. Because this bond has a substantially different equilibrium length, its stretch force constant has a value different from that of the other two bonds. For this reason, bonds of substantially different equilibrium lengths should be classified into different stretch types even if they are comprised of the same atom types.

### 5.3 Generating the list of angles to use in the forcefield

**Fig. 8:**
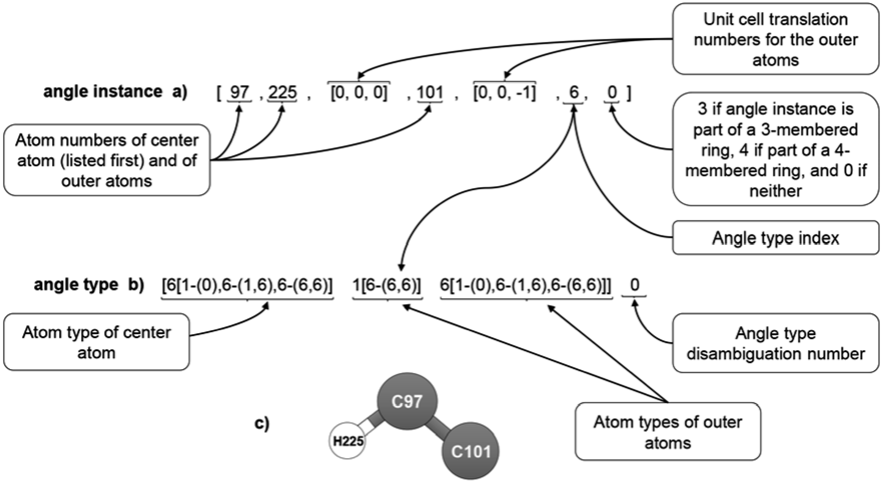
Example format for an angle instance (a), angle type (b), and ball-and-stick illustration (c).

We first constructed a list of all angle instances for which the center atom in the bond angle resides within the reference unit cell (and thus has translation indices equal to (0,0,0)). Each of the two outer atoms may reside in either the reference unit cell or one of its neighboring unit cells. Fig. 8 illustrates the information stored in each angle instance. The atom number of the center atom is listed first. The atom numbers and translation indices of the outer atoms is then listed. Just as for the stretch instances, these two atoms are ordered such that their atom types are in alphabetical order; this makes it easier for the code to lookup angle instances of the same angle type. This is followed by the angle type index. The last number indicates whether the angle is inside a 3-membered ring, a 4-membered ring, or neither (an angle is considered to be part of a 3-membered or 4-membered ring if both bonds comprising the angle are part of the ring. If only one bond is part of such a ring, the angle is not considered to be part of the ring.). Each angle instance appears exactly once in the list with no duplicates, so there is no need to store the number of times each angle instance appears within the list.

In this work, angle instances that are part of 3-membered or 4-membered rings were not used in the angle-bending potential, because those degrees of freedom were already described by the bond stretches (for 3-membered rings) or UB stretches (for 4-membered rings). However, all angle instances (including those which are part of 3-membered or 4-membered rings) were used to construct bond–bond cross terms, when the potential model included bond–bond cross terms.

Two angle instances were classified into the same angle type iff they had the same combination of bond types and their equilibrium angle values differed by less than a chosen tolerance (as explained in the previous section, two instances of the same bond type necessarily have the same combination of atom types and similar equilibrium bond lengths). In this work, the equilibrium value for each angle instance was rounded to the nearest 0.01 radians. If two angle instances had the same combination of bond types and their equilibrium angle values matched (when rounded to two decimal places), then they were placed into the same angle type; otherwise, they were placed into different angle types. As shown in Fig. 8, multiple angle types that contain the same combination and order of atom types (but have different bond types or different equilibrium angle values) are distinguished by the ‘angle type disambiguation number’ which labels them as 0, 1, 2, ….

Fig. 7 illustrates the critical importance of including the equilibrium angle value in the angle-typing scheme. For example, all bond angles in the sulfur hexafluoride (SF_6_) molecule have the same combination and order of atom types and bond types. However, there are two dramatically different types of bond angles in this molecule: (i) 90° F–S–F angles and (ii) 180° F–S–F angles. Because these two angle types can have different force constant values, they need to be included as separate angle types in the flexibility model.

### 5.4 Generating the list of proper dihedrals to use in the forcefield

#### 5.4.1 Constructing the dihedral types and instances

The list of dihedral instances was generated as follows. We start with the complete list of angle instances for which the center atom in the bond angle resides within the reference unit cell, as explained in Section 5.3 above. Now, a dihedral instance can be generated by adding a bond to either end of a bond angle. For example, starting with bond angle ABC, if atom C is directly bonded to atom D, then we can generate the dihedral instance ABCD. In this example, if atom A is directly bonded to atoms E and F, then we can also generate the dihedral instances EABC and FABC.

During this process, we have to keep track of the unit cell translation indices for each atom in the dihedral instance. For example, dihedral instance A(0,0,−1)B(0,0,0)C(0,1,0)D(0,1,0) means that atom A resides inside the (0,0,−1) unit cell, atom B resides within the reference (*i.e.*, (0,0,0)) unit cell, atom C resides within the (0,1,0) unit cell, and atom D resides within the (0,1,0) unit cell. As explained in Section 5.3 above, the center atom in each angle instance resides within the reference unit cell. By examining all bonds connecting to either end of each angle instance, we can generate the full list of dihedral instances for which at least one of the two middle atoms resides within the reference unit cell.

During this process, a dihedral instance containing exactly the same set of unit cell translation indices is added only one time to the dihedral instances list. For example, dihedral instance A(0,0,−1)B(0,0,0)F(0,0,0)G(0,1,0) will be generated both by adding atom image G(0,1,0) to the F end of the A(0,0,−1)B(0,0,0)F(0,0,0) bond angle and also by adding atom image A(0,0,−1) to the B end of the B(0,0,0)F(0,0,0)G(0,1,0) bond angle (in this notation, B(0,0,0) is the center atom of the ‘A(0,0,−1)B(0,0,0)F(0,0,0)’ bond angle). Before adding a specific dihedral instance to the list, our code checks to see if it is already included in the list for the same unit cell translation indices; therefore, A(0,0,−1)B(0,0,0)F(0,0,0)G(0,1,0) is contained exactly once not twice within the list of dihedral instances.

**Fig. 9:**
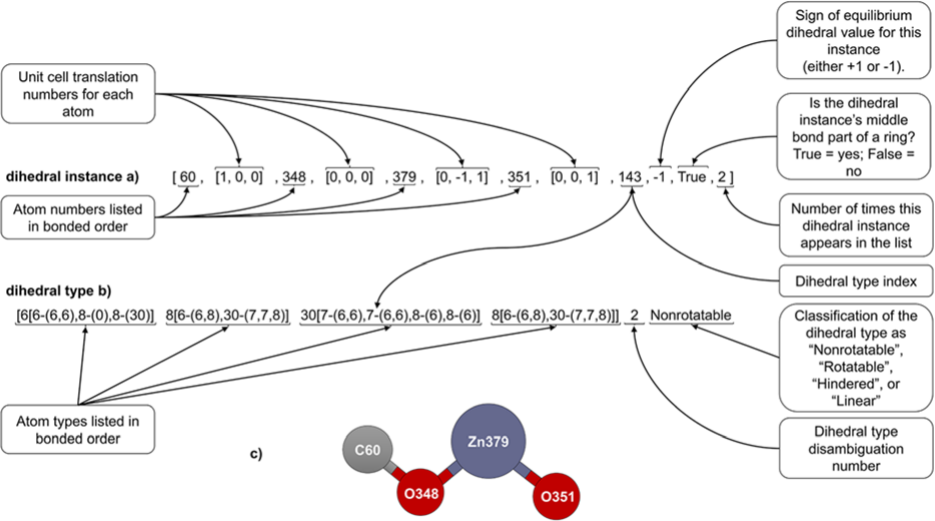
Example format for a dihedral instance (a), dihedral type (b), and ball-and-stick illustration (c).

However, a single instance containing different translation indices can be included twice within the list of dihedral instances. For example, both A(0,0,−1)B(0,0,0)C(0,1,0)D(0,1,0) and A(0,−1,−1)B(0,−1,0)C(0,0,0)D(0,0,0) will appear within the dihedral instances list, even though they are translations of the same dihedral instance. Fig. 9 illustrates the data stored for each dihedral instance. The last number is the number of times each dihedral instance appears within the list (this number will be either 1 or 2). This number is important to avoid double-counting when computing the potential energy. Specifically, if there are *N*_duplicates_ duplicate instances of the same dihedral instance in the list, then the factor of (1/*N*_duplicates_) will be applied to each duplicate when computing the potential energy, so that the potential energy for this instance is counted exactly *N*_duplicates_(1/*N*_duplicates_) = 1 time.

When computing the number of ‘stretch instances in a stretch type’ and the number of ‘dihedral instances in a dihedral type’, the duplicates are not double-counted. For example, a bond stretch type containing the bonds A(0,0,0)B(1,0,0), A(−1,0,0)B(0,0,0), and C(0,0,0)D(0,0,0) is said to contain two bond instances rather than three, because A(0,0,0)B(1,0,0) and A(−1,0,0)B(0,0,0) are translated images of the same bond.

**Fig. 10:**
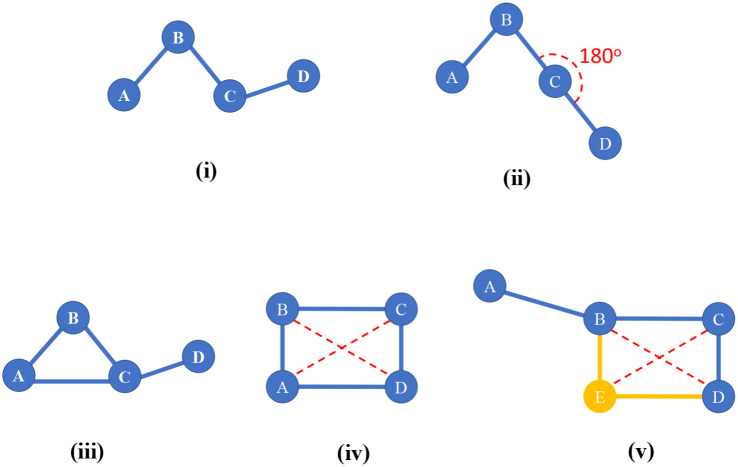
Illustration of proper dihedrals involving atoms A, B, C, and D. Panels (i) and (ii) show examples of dihedrals that were used in our flexibility model. Panel (i) shows a dihedral in which neither contained equilibrium bond angle is linear. Panel (ii) shows a dihedral in which one of the contained equilibrium bond angles is linear. Panels (iii) to (v) show examples of dihedrals that were not used in our flexibility model. The 3-member ring in panel (iii) is already described by the bond lengths, so no corresponding dihedral term in the flexibility model is required. The 4-member ring in panel (iv) is already described by its six stretches: AB, BC, CD, AD, AC, and BD. In panel (v), both the ABCD and the EBCD dihedrals were excluded, because the BCD angle is inside a 4-membered ring.

As shown in Fig. 10, certain kinds of dihedrals are deleted from the list of dihedral instances. A dihedral instance is deleted if it contains a 3-member ring. A dihedral instance is deleted if at least one of its contained bond angles is inside a 4-member ring. These dihedral instances are deleted, because one of the underlying angles is part of a 3-member or 4-member ring and does not appear in the active list of angles that are treated by the angle-bending potential. As explained in a previous section, the internal coordinate degrees of freedom of the 3-member and 4-member rings are covered by the bond stretches and UB stretches.

The remaining entries in the dihedral instance data are described as follows. The sign of the equilibrium dihedral value was set to +1 if *ϕ*_eq_ ≥ 0 and to −1 if *ϕ*_eq_ < 0. Each dihedral type was assigned an index number. For each dihedral instance, the index number of the dihedral type that it belongs to was stored. Also, an entry was stored that indicated whether the dihedral instance's middle bond belonged to ring: “True” = belonged to a ring, “False” = did not belong to a ring. The algorithm we used to detect rings is described in Section 5.4.2.

Two dihedral instances were classified into the same dihedral type iff they had the same combination of angle types and the absolute values of their equilibrium dihedrals differed by less than a chosen tolerance (as explained in the previous section, two instances of the same angle type necessarily have the same combination of bond types, same combination of atom types, and similar equilibrium angle values). In this work, the equilibrium value for each dihedral instance was rounded to the nearest 0.01 radians. If two dihedral instances had the same combination of angle types and the absolute values of their equilibrium dihedrals matched (when rounded to two decimal places), then they were placed into the same dihedral type; otherwise, they were placed into different dihedral types. As shown in Fig. 9, multiple dihedral types that contain the same combination and order of atom types (but have different angle types or different absolute values of their equilibrium dihedrals) are distinguished by the ‘dihedral type disambiguation number’ which labels them as 0, 1, 2, …. The final entry in the dihedral type indicates whether it is classified as ‘nonrotatable’, ‘rotatable’, ‘hindered’, or ‘linear’ according to the criteria explained in Section 5.4.4.

#### 5.4.2 Identifying whether the middle bond (of a dihedral instance) is part of a ring

**Fig. 11:**
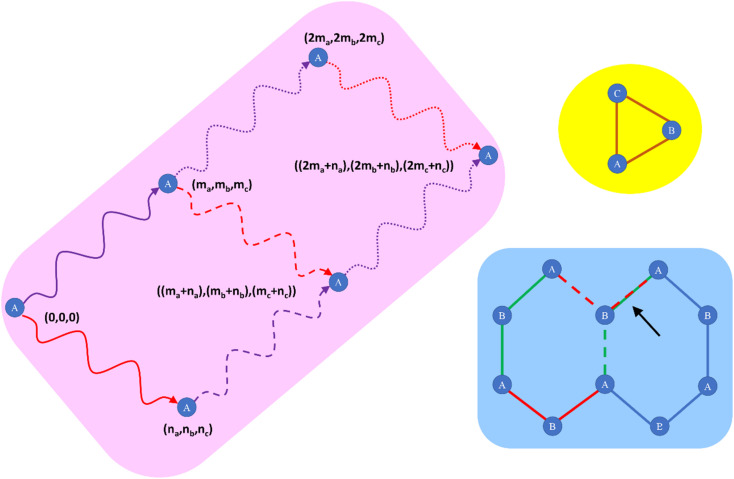
Illustration showing why the smallest bond path cycle passing through a particular bond cannot contain more than four translated images of the same atom. Please see the text for a detailed description.

If a bond is part of a ring (*i.e.*, a bond path cycle) in a periodic crystal, then at least one ring passing through the bond contains fewer than (4*N*_atoms_ + 1) atoms, where *N*_atoms_ is the number of atoms in the material's periodic unit cell. This can be shown by proving the smallest (*i.e.*, shortest) bond path cycle passing through a bond contains no more than four periodic images of the same parent atom. Fig. 11 illustrates the underlying reasons for this. The region shaded pink in Fig. 11 illustrates any case in which a bond path exists from a first image of atom A in the reference (*i.e.*, (0,0,0)) unit cell to a second image of atom A denoted by the unit cell translation indices (*m*_a_, *m*_b_, *m*_c_) and a bond path also exists from the first image of atom A to a third image of atom A denoted by the unit cell translation indices (*n*_a_, *n*_b_, *n*_c_) which does not lie along the infinite line passing through the first and second images. The latter condition is equivalent to saying that (*n*_a_, *n*_b_, *n*_c_) does not equal (c(*m*_a_), c(*m*_b_), c(*m*_c_)) for any value of c. Moreover, we choose (*m*_a_, *m*_b_, *m*_c_) and (*n*_a_, *n*_b_, *n*_c_) such that they are the closest images to the first image along each of these bond paths. This is equivalent to choosing (*m*_a_, *m*_b_, *m*_c_) such that the greatest common factor of *m*_a_, *m*_b_, and *m*_c_ is one, and also choosing (*n*_a_, *n*_b_, *n*_c_) such that the greatest common factor of *n*_a_, *n*_b_, and *n*_c_ is one. For example, starting with the proposed second image (2*m*_a_, 2*m*_b_, 2*m*_c_) we divide by the greatest common factor (2 in this case) to arrive at (*m*_a_, *m*_b_, *m*_c_) as the actual location of the second image. Because of the periodic boundary conditions, it immediately follows that a fourth image of atom A characterized the unit cell translation indices ((*m*_a_ + *n*_a_), (*m*_b_ + *n*_b_), (*m*_c_ + *n*_c_)) has: (i) a bonded path to the second image of atom A and (ii) a bonded path to the third image of atom A. Thus, it follows that a bonded path exists from the first image of atom A to the second image of atom A to the fourth image of atom A to the third image of atom A and back to the first image of atom A. Thus, it necessarily follows that when any bond path cycle exists that passes through a particular bond, we can find a smallest (*i.e.*, shortest) bond path cycle passing through that bond that such that the number of translated images of the same parent atom is no more than four. Since there are *N*_atoms_ in the reference unit cell, it means the shortest bond path cycle (if any exists) passing through a particular bond contains no more than 4*N*_atoms_ atoms.

As shown in blue-shaded region of Fig. 11, the connected path described above from image 1 to image 2 to image 4 to image 3 and back to image 1 of atom A is not necessarily itself a bond path cycle. Specifically, the blue-shaded region shows a graphene segment. Taking the lower left image of atom A to be the (0,0,0) image, the solid red path shows a bond path to image 2 of atom A, and the solid green path shows a bond path to image 3 of atom A. The dashed red path shows a bond path connecting image 3 to image 4. The dashed green path shows a bond path connecting image 2 to image 4. Interestingly, in this case the dashed green and dashed red paths overlap; consequently, the shortest bond path cycle (which happens to be a 6-membered ring) contains 3 images of atom A rather than 4.

The yellow-shaded region in Fig. 11 illustrates a simple case (*e.g.*, a triangle connecting atoms A, B, and C) for which the shortest bond path cycle contains a single image of atom A.

ESI Section S2† contains a rigorously correct and complete Python function we wrote that determines which middle bonds (of the dihedral instances) are parts of rings (*i.e.*, bond path cycles) and which are not.

#### 5.4.3 Pruning redundant dihedral types

**Fig. 12:**
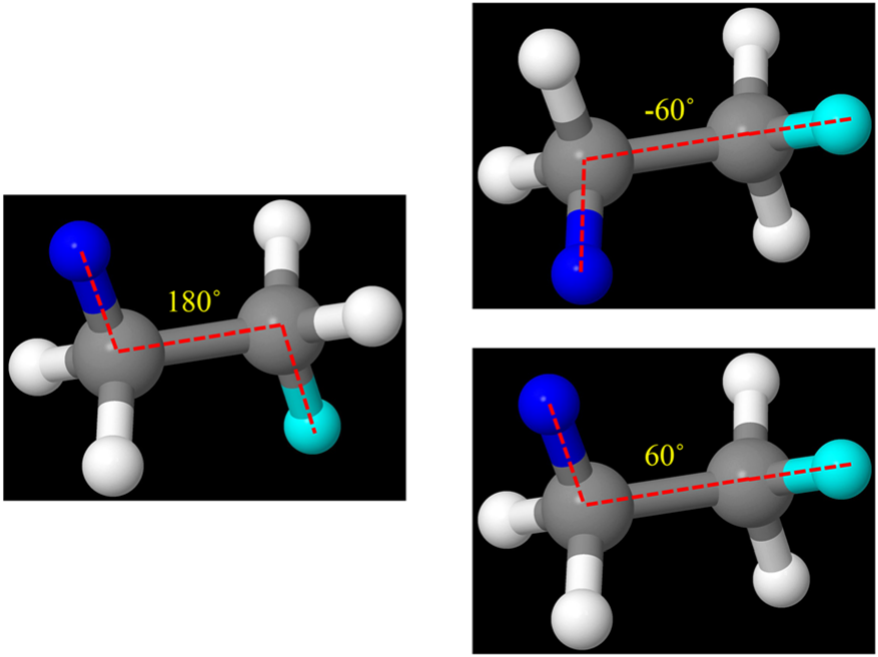
Illustration of coupled dihedral types for ball and stick model of ethane (C_2_H_6_). Here, selected dihedrals are highlighted using blue as the color of the first atom and cyan as the color of the last atom. We can define two dihedral types for ethane with different absolute values of dihedral angles: 180° (left panel) and 60° (right panels). These dihedrals have the same C–C as their middle bond. To construct a concise forcefield, we can use either dihedral type and discard the other.

As an example, consider the ethane molecule shown in Fig. 12. Because this molecule has a total of 9 dihedral instances but only one middle bond, this means rigid rotation of any one of these dihedral instances causes all of the other 8 dihedral instances to also rigidly rotate. If we discard 8 of these dihedral instances and keep the remaining dihedral instance to construct the flexibility model, then this breaks the molecule's symmetry. To preserve the symmetry equivalency, we must therefore keep and discard the dihedral types rather than individual dihedral instances. In ethane, there are two dihedral types, and these have absolute values of dihedrals of 180° (containing 3 instances) and 60° (containing 6 instances). To construct a concise flexibility model that preserves the symmetry equivalency, we can keep the dihedral type with 3 instances and discard the dihedral type with 6 instances.

We use the term ‘coupled dihedral types’ to mean dihedrals that share the same set of middle bond instances. The process of discarding some of the coupled dihedral types is called ‘dihedral pruning’. Because all dihedral instances of the same dihedral type are either all kept or all discarded, this dihedral pruning preserves the symmetry equivalency.

Our SAVESTEPS program performs dihedral pruning using the following procedure. First, it identifies which dihedral types share the same set of middle bond instances. When making this comparison, repeated values are not important. For example, the set {a,b,c,d} is considered equivalent to the set {b,c,a,d,c,a,d,b} but not to the sets {a,b,c}, {a,b,c,e}, or {a,b,c,d,e}, where ‘a’, ‘b’, ‘c’, ‘d’, and ‘e’ label particular middle bond instances. For each dihedral type, the following metric is computed:42
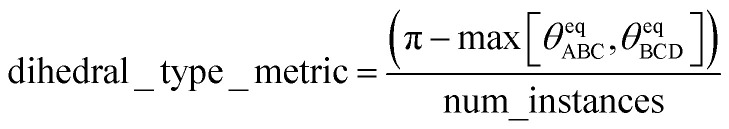
where max[*θ*^eq^_ABC_,*θ*^eq^_BCD_] is the maximum value of the two equilibrium bond angles for that dihedral type, and num_instances is the number of dihedral instances in that dihedral type. Among two or more coupled dihedral types (*i.e.*, those sharing the same set of middle bond instances), the dihedral type having the largest value of dihedral_type_metric is retained while the others are discarded. If two or more dihedral types tie for the largest value of dihedral_type_metric, then the software program uses a random number generator to randomly select which dihedral type (among those that tied for the largest value of dihedral_type_metric) to keep and discards the others.

Among coupled dihedral types, why is a dihedral type having the largest value of dihedral_type_metric retained while the others are discarded? This has the following simple explanation. Since the CADT potential is simpler and more computationally efficient than the ADDT potential, it would be preferable to retain a dihedral type having contained bond angles far away from linear (*i.e.*, maximizing (180° − max[*θ*^eq^_ABC_,*θ*^eq^_BCD_])). To maximize the computational efficiency, it would also be preferable to keep the coupled dihedral type that has the smallest number of dihedral instances. The dihedral_type_metric (see eqn (42)) combines these two criteria into a single descriptor whose value is to be maximized.

#### 5.4.4 Classifying each dihedral type as ‘nonrotatable’, ‘rotatable’, ‘hindered’, or ‘linear’

Each dihedral type was classified as ‘nonrotatable’, ‘rotatable’, ‘hindered’, or ‘linear’. A dihedral type was classified as ‘linear’ iff one of its equilibrium bond angles was close to linear; that is, if either π − *θ*^eq^_ABC_ < *ε* or π − *θ*^eq^_BCD_ < *ε*, where *ε* is a tolerance (*e.g.*, 0.03 radians).

What exactly does it mean for a dihedral type to be ‘non-rotatable’? Grimme classified a bond as ‘nonrotatable’ if it was part of a ring or had a bond order greater than 1.3.^6^ According to Grimme's definition, the C=C bond in ethene (*i.e.*, C_2_H_4_ molecule) would be classified as ‘nonrotatable’, because its bond order equals ∼2. Our dihedral typing protocol does not use the bond orders as inputs and instead classifies a dihedral type as ‘non-rotatable’ iff at least one dihedral instance belonging to this dihedral type has a middle bond that is part of a ring (*i.e.*, bond path cycle). Using our definition, the C=C bond in ethene would be classified as ‘rotatable’ even though it has a high rotational energy barrier.

Are there situations in which the rotational barrier is small even though a dihedral instance's middle bond is part of a bond path cycle? Consider a polymer made of benzene rings where the 1,4-position carbons of each benzene ring are bonded to adjacent benzene rings to form the structure (C_6_H_4_)_*n*_. Connecting the two ends of this polymer together forms a loop (aka ‘necklace’). Even though each C–C bond in this ‘necklace’ belongs to at least one bond path cycle, it still seems possible for each benzene ring to rotate about an axis running through its 1,4-position carbon atoms. Thus, we must offer the caveat that being part of a bond path cycle ‘normally’ but ‘not universally’ hinders rotations about a middle bond.

For simplicity, our SAVESTEPS algorithm (at least in its current form) classifies a dihedral type as nonrotatable iff at least one of its dihedral instances has a middle bond that is part of a ring. If a dihedral type had some dihedral instances whose middle bond was part of a ring and other dihedral instances whose middle bond was not part of a ring, then this dihedral type was still classified as ‘nonrotatable’.

Consider a dihedral type for which none of its dihedral instances had a middle bond that was part of a ring. Using a random number generator, the SAVESTEPS program randomly chose one of the dihedral instances in this type. Next, we rotated the dihedral for this instance from *ϕ* = −170° to 180° in 10° increments to produce *T* = 36 geometries comprising a rigid torsion scan. Next, we computed the atom types for each atom in each of these geometries and compared them to the atom types in the reference geometry (see Section 6.2 for a description of the reference geometry). If the atom types in each of the rigid torsion scan geometries matched those in the reference geometry, this means no new bonds were formed and no bonds were broken during the rigid torsion scan. In this case, the corresponding dihedral type was classified as ‘rotatable’. On the other hand, if any atom type changed for any atom in any of the rigid torsion scan geometries compared to the reference geometry, then the dihedral type was classified as ‘hindered’. This corresponds to the situation in which the dihedral cannot rigidly rotate through its full range without forming new bonds and/or breaking old bonds. For example, this could correspond to a situation in which one chemical group collides with another chemical group (aka ‘steric collision’) during part of the rigid torsion scan. During the subsequent force constants optimization, hindered and nonrotatable dihedral types are treated on the same footing and use the same forms of torsion model potentials (*i.e.*, CADT_1 or ADDT_1).

The above analysis process was individually applied to each dihedral type for which none of its dihedral instances had a middle bond that was part of a ring. In such a way, each dihedral type having no middle bond instances that were part of a ring was classified as either ‘rotatable’ or ‘hindered’.

Why did we classify an entire dihedral type as non-rotatable even if only some of its instances were part of a ring instead of treating the instances that were not part of a ring as rotatable? This was a pragmatic choice based on two observations. Observation # 1: if a particular dihedral instance that is not part of a ring is classified as non-rotatable, this has negligible effect on small dihedral displacements but severely restricts large dihedral displacements (*e.g.*, Δ*ϕ* ≥ π/4). If this particular dihedral instance should be rotatable, the parameterized flexibility model will still correctly describe small dihedral displacements but will undercount the large dihedral displacements for this particular dihedral instance. Accordingly, the parameterized flexibility model will still be functional even if not exact. Observation # 2: we carefully reviewed the entire set of 116 MOFs and found that the situation of no-ring and ring dihedral instances belonging to the same dihedral type occurred in only three (*i.e.*, AFITEP, AMOYOR, and PORVUO) of these MOFs. We then manually examined each of these MOFs using a visualization program. We found that this situation corresponded to sprawling bonded networks that were a combination of tree-branch-like structures and spider-web-like structures. The local bonded structure of a tree-branch-like structure looked identical to that of a spider-web-like structure; however, their long-range structures differed in that the tree branches were not part of a bonded ring while the spider webs were part of bonded rings. Because the tree branches were long, they would have given rise to ‘hindered’ rotation and thus were not freely rotatable. ‘Non-rotatable’ and ‘hindered’ dihedrals use the same dihedral model potential, so the distinction between the two does not impact the parameterized flexibility model. In summary, these empirical observations support the practice of classifying a dihedral type containing nonzero numbers of both ring and no-ring dihedral instances as ‘non-rotatable’ for pragmatic reasons.

It is critically important to use the same rigid torsion scan geometries for the test to see if the dihedral type is ‘rotatable’ or ‘hindered’ as is subsequently used for the single-point quantum-chemistry calculations to form the rotatable dihedrals training dataset. Specifically, if the dihedral type is classified as ‘rotatable’, then this process has verified that the bond connectivity graph is unchanged during the rigid torsion scan. This is an important pre-requisite for the single-point energy calculations that formed the rotatable dihedrals training dataset, as described in Sections 6.4 and 7.1–7.4.

## 6. Quantum chemistry calculations to compute reference data

### 6.1 Common settings

All periodic quantum chemistry calculations were computed using the PBE^99^ exchange–correlation functional with DFT-D3 Becke–Johnson damping function^100,102,103^ using the Vienna *ab initio* simulation package (VASP).^104–108^ The project or augmented-wave (PAW) method^109,110^ was used. The PAW method is a frozen-core all-electron calculation. An energy convergence criterion of 10^−6^ eV was used for the self-consistent field (SCF) cycles. The number of *k*-points was set so that for each lattice vector the length times the number of *k*-points was greater than 16 Å. The planewave energy cut-off was set to 400 eV. A Prec = Accurate grid with Addgrid = False was used to avoid wrap-around (aka aliasing) errors. These settings were shown in previous work to give accurate results.^58^

### 6.2 Geometry optimization

DFT-with-dispersion geometry optimization was performed allowing the atomic positions to relax with the unit cell volume and shape held fixed at the experimental values taken from the 2019 CoRE MOF^91^ dataset. The convergence criterion was that each force component (*e.g.*, *F*_*x*_, *F*_*y*_, *F*_*z*_) was smaller in magnitude than 0.01 eV Å^−1^ for each atom. This constitutes the ‘reference geometry’ for which all atom-in-material forces in the subsequently parameterized flexibility model will be zero.

We originally applied an earlier variant of this protocol to DFT-optimized structures we computed that fully relaxed both the atom-in-material positions and the unit cell's size and shape. Upon further investigation, we came to believe that the DFT-optimized lattice vectors (which determine the unit cell's size and shape) were less accurate than the experimentally measured lattice vectors for these materials. Technically speaking, quantum chemistry calculations do not generate rigorously correct optimized lattice vectors because of the Pulay stresses due to basis set incompleteness.^111^ While using an extremely large basis set with a fine *k*-point mesh can mitigate this issue,^111–113^ quantum chemistry calculations near the complete basis set limit are computationally expensive. For these reasons, we believe it is usually preferable to construct the reference geometry by allowing the atomic positions to relax during geometry optimization with the unit cell volume and shape held fixed at the experimental values. Accordingly, all computational results presented in this article were obtained using the experimental lattice vectors.

Using the experimental lattice vectors involves three caveats. First, some materials have not been characterized experimentally yet. For these new materials, experimentally-measured lattice vectors are not available, and one should instead use the quantum-mechanically-computed lattice vectors (this case did not arise for any materials in this study). Second, as pointed out by one of the anonymous reviewers of this article: “experimental characterization of MOFs often takes place on solvated structures, so the experimental values do not always pertain to the more relevant empty/activated structures”. Third, experimental characterization often takes place at room temperature while the electronic ground-state structure should technically correspond to a temperature of absolute zero. In spite of these three caveats, it is still true that often the experimentally-measured lattice vectors have smaller uncertainties and smaller errors than their quantum-mechanically-computed counterparts. The important principle is to use whichever lattice vectors are more accurate: the experimental ones or the quantum-mechanically-computed ones.

### 6.3 *Ab initio* molecular dynamics and finite-displacement ‘Hessian’ calculations

To achieve a comprehensive sampling of all internal motion modes, we employed a combination of *ab initio* molecular dynamics (AIMD) and finite-displacement Hessian calculations. AIMD simulations provide information about larger random displacements, while Hessian calculations systematically sample every degree of freedom using small finite displacements. By combining these techniques, we achieved a more rigorous sampling that includes both small finite displacements of every mode as well as some larger displacements of randomly selected modes. Ten AIMD runs were performed for each structure to generate training set data. Another 10 AIMD runs per structure were performed to generate validation set data. The forces and geometries were extracted and assembled into a csv file. Then, these csv files were read into a python program that generates the flexibility parameters through least-squares regression as described in Section 7 below.

For the AIMD calculations, the number of geometry steps per run was set to 100 starting from the optimized geometry. The forces were calculated as a response to the changes in atom positions while keeping the cell shape and volume constant. A time step of 1 femtosecond was used with a starting temperature of 300 K and a microcanonical (NVE) ensemble. This corresponded to the following VASP settings: IBRION = 0 (chooses molecular dynamics), NSW = 100 (chooses 100 geometry steps), ISIF = 0 (chooses fixed cell volume and shape while atoms move), MDALGO = 0 and SMASS = −3 (chooses NVE ensemble), POTIM = 1 (chooses 1 femtosecond time step), TEBEG = 300 (chooses starting temperature).

The Hessian matrix was computed using a finite difference method with a displacement size of 0.07 Å and four displacements per direction. Specifically, the atomic positions were displaced by −0.14 Å, −0.07 Å, 0.07 Å, and 0.14 Å along each of the *x*, *y*, and *z* axes, resulting in a total of 12 displacements per atom. This corresponded to the following VASP settings: NSW = 1, ISIF = 0, IBRION = 5, POTIM = 0.07, NFREE = 4.

### 6.4 Rotatable dihedral single-point calculations

To explore the potential energy associated with the rotation of certain dihedrals, single-point (*i.e.*, rigid geometry) calculations were carried out using the common VASP settings (Section 6.1) as follows. Within each rotatable dihedral type, a single dihedral was randomly selected and rotated in 10° increments from a dihedral value of −170° to 180°. The procedure resulted in 36 different geometries for the selected dihedral. The process was repeated for each rotatable dihedral type in the MOF. This generated energy *versus* dihedral value curves that were analyzed as described in Section 7 below (As shown in ESI Section S6,† we performed a test in which torsion scan curves were generated for every instance of a randomly chosen rotatable dihedral type. All of those torsion scan curves were identical. More generally, if two instances of the same type have different signs for *ϕ*_eq_, then one would have torsion scan curves for these two instances that are mirror images of each other; since this case is automatically handled by Manz's torsion model potentials, it does not require generating separate torsion scan curves for these two instances. This validates the method of generating a torsion scan curve for one instance of each rotatable dihedral type.).

We prepared these rigid torsion scan geometries using Rodrigues' rotation formula:43
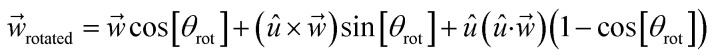
where *û* is the axis of rotation, *θ*_rot_ is the angle of rotation, 
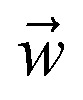
 is the vector before rotation, and 
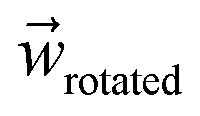
 is the vector after rotation. The rotation angle was set as

44 *θ*_rot_ = *ϕ*_desired_ − *ϕ*_eq_

where *ϕ*_desired_ is the desired value of *ϕ*_ABCD_ and *ϕ*_eq_ is the value of *ϕ*_ABCD_ in the reference geometry (*i.e.*, the quantum-mechanically-optimized ground-state geometry using the experimental lattice vectors without any dihedral constraints). Let 
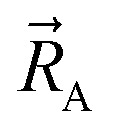
 be the position of atom A in the (0,0,0) unit cell. Let (A,L^A^_1_,L^A^_2_,L^A^_3_) denote a translated atom A image whose position is45
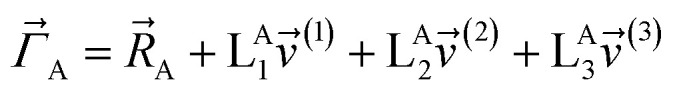
where 
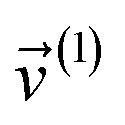
, 
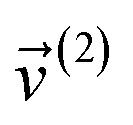
, and 
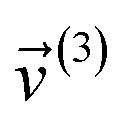
 are the unit cell's lattice vectors. Analogous formulas hold for all other atom images. For example, 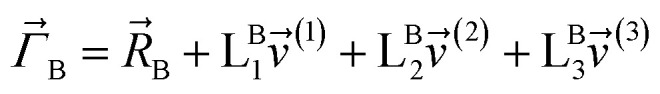 is the position of atom image (B,L^B^_1_,L^B^_2_,L^B^_3_). For a rotatable dihedral ABCD in the extended structure (bonded_group_A)BC(bonded_group_D), we computed whether the bonded_group_A emanating from atom A was smaller than the bonded_group_D emanating from atom D. If bonded_group_A contained fewer atoms than bonded_group_D, then atom image B was chosen as the origin (*i.e.*, 
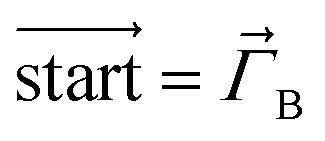
) for the rotation; otherwise, atom image C (*i.e.*, 
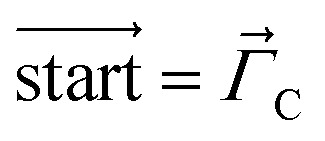
) was chosen as the origin for the rotation. The axis of rotation, *û*, is the unit vector parallel to the middle bond 
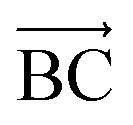
 and pointing towards the chosen origin; that is, pointing along the direction from C to B if bonded_group_A contained fewer atoms than bonded_group_D, otherwise pointing along the direction from B to C. 
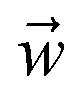
 is the vector from the chosen origin to a particular atom image E in the bonded group being rotated, as computed in the reference geometry:46
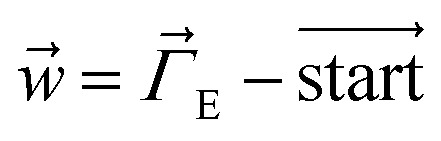


If bonded_group_A contained fewer atoms than bonded_group_D, then bonded_group_A is the group being rotated; otherwise, bonded_group_D is the group being rotated. The position of atom image E after the dihedral rotation is47
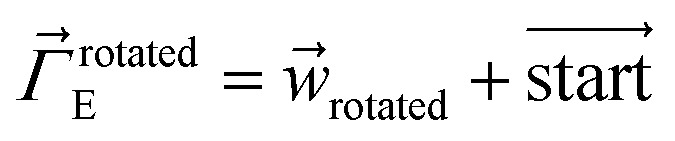
By converting 
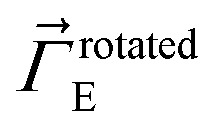
 to fractional coordinates and then converting the decimal part of the fractional coordinates back to real space, the position 
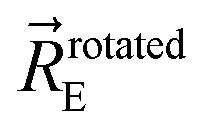
 of the rotated atom E within the (0,0,0) unit cell can be computed from 
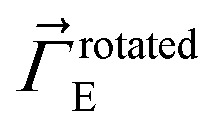
. This process was repeated for each and every atom in the bonded group being rotated to find their new positions; the positions of all other atoms were the same as in the reference geometry.

## 7. Least-squares fitting to extract the flexibility parameters

### 7.1 Smart selection of rotatable dihedral potential modes

As described in Section 6.4 above, we quantum-mechanically-computed energies, *E*^QM^_RTS_[*ϕ*], along a rigid torsion scan (RTS) for one rotatable dihedral instance in each rotatable dihedral type. Along this curve for a particular dihedral instance, the geometries must be equally spaced in dihedral value over the range (−π,π]. For example, we used *T* = 36 geometries with equally spaced dihedral values *ϕ*= −170°, −160°, …,170°, 180°. Along this curve for a particular dihedral instance, the average energy is48
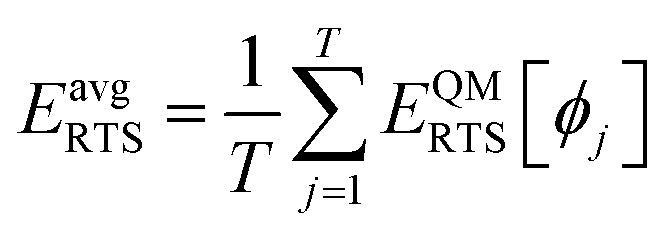
and the self-overlap integral is:^50^49
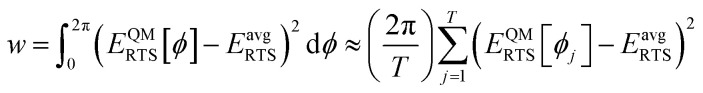


As described in the companion article, the first seven independent torsion modes have the following orthogonal basis functions when the average potential of each torsion mode has been shifted to zero:^50^50
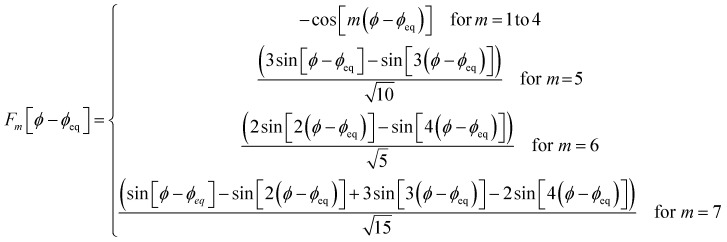


This allows *E*^QM^_RTS_[*ϕ*] be approximated by the following model

51 *E*^QM^_RTS_[*ϕ*] ≈ *E*^model^_RTS_[*ϕ*]

52
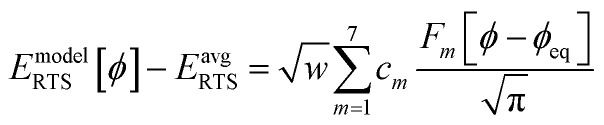
where the coefficients for each mode are defined as^50^53
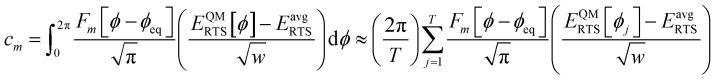


The goodness of fit (aka *R*-squared value) for this RTS model equals the sum of squared coefficients54
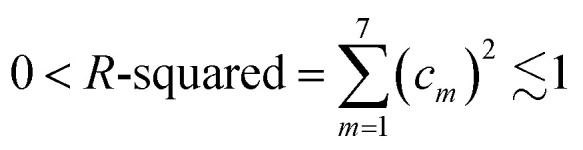
where the sum is performed over all modes included in the model.^50^

In this work, we considered mode *m* to be active iff abs[*c*_*m*_] > 0.1; in this case, we included mode *m* in the subsequent flexible forcefield model. If abs[*c*_*m*_] ≤ 0.1, the mode was considered inactive and not included in the subsequent flexible forcefield model. We call this process ‘smart selection of rotatable dihedral potential modes’. Consequently, the torsion potential for a rotatable dihedral type can be represented as a linear combination of one to seven modes. The goodness of fit (aka *R*-squared value) for this ‘smart-selected’ RTS model equals the sum of squared coefficients:55
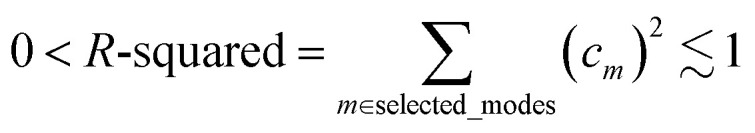
where the sum is performed over the selected modes included in the model.

**Fig. 13:**
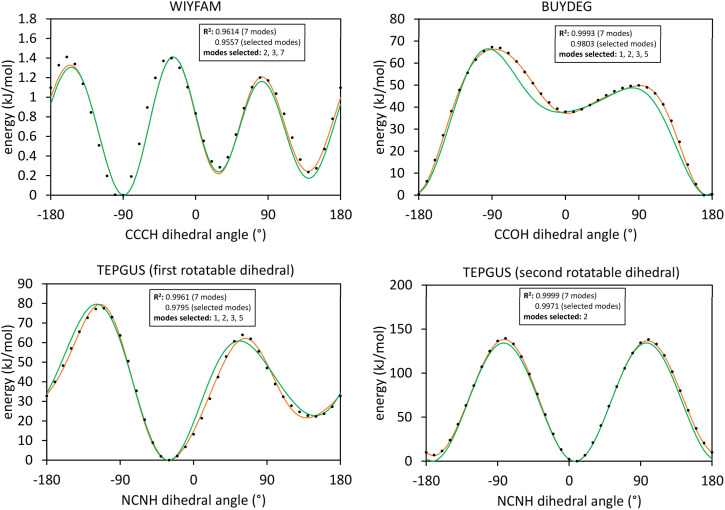
Potential energy curves for rigid torsion scans of rotatable dihedrals. In each panel, the black dots show the quantum mechanical energy obtained from single-point DFT_with_dispersion calculations. The orange curve illustrates the fitted model using all 7 modes, while the green curve shows the fitted model using only the smart-selected modes.

Fig. 13 plots examples of rigid torsion scans for rotatable dihedrals in selected MOFs. The top panels display the results for MOFs with only one rotatable dihedral type, while the bottom panels display the results for one MOF with two rotatable dihedral types. The *R*-squared values close to one show the models performed well.

### 7.2 Linear equations for flexibility parameters

Our linear regression problem contains two kinds of observation variables in the combined training dataset: (a) quantum-mechanically-computed atom-in-material forces and (b) quantum-mechanically-computed total energies. The quantum-mechanically-computed atom-in-material forces are from the QM-optimized geometry, AIMD geometries, and finite-displacement ‘Hessian’ geometries. There are a total of

56 *f*_rows = 3*N*_atoms_*N*_force_geoms_

force components in the forces training dataset. The quantum-mechanically-computed total energies are from the rigid torsion scan (RTS) geometries and comprise the rotatable dihedrals training dataset. This gives the following observation variables for the combined training dataset 
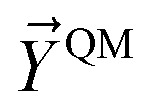
 comprised of the forces training dataset 
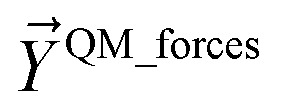
 and the rotatable dihedrals training dataset 
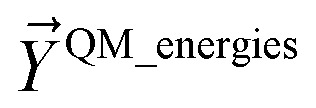
:57
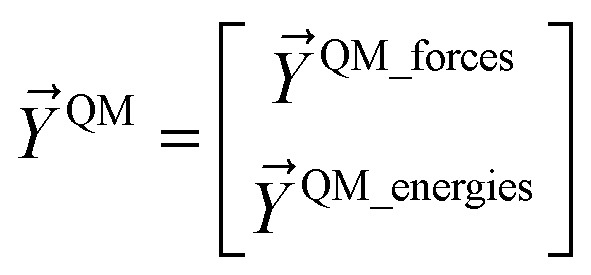


The predictor variables also contain two sets of data: one for forces fitting and the other for the rotatable dihedrals fitting. Let **M** be a matrix containing values of the predictor variables. This leads to the following linear model:58
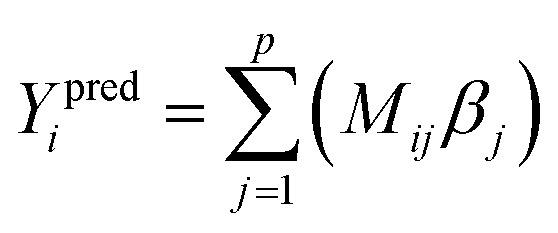
where *i* is the observation index and *j* is the model parameter index. In our case, each model parameter is a force constant for a flexibility term:

59 *β*_*j*_ = *k*_*j*_

The total number of attempted force constants in the model is *p*. Here, the term ‘total number of attempted force constants’ refers to the number of flexibility terms (*i.e.*, number of force constants) that were ‘attempted’ before any of these were zeroed out by the bounds or regularization constraints.

Because the atom-in-material forces for the AIMD-generated geometries depend on all of the force constants while the RTS energies depend only on the rotatable dihedral force constants, it follows that the predictor variables matrix **M** has the form60
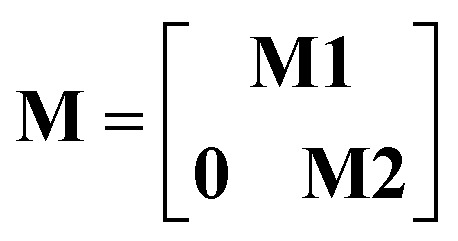
where

61 size[**M1**] = (*f*_rows,*N*_AFCs_)

62 size[**M2**] = (*TN*_rdt_,*N*_rd_AFCs_)

*N*_AFCs_ is the total number of attempted force constants. *N*_rd_AFCs_ is the number of rotatable dihedral attempted force constants.

To define the **M1**, we need to start from the following relation:63
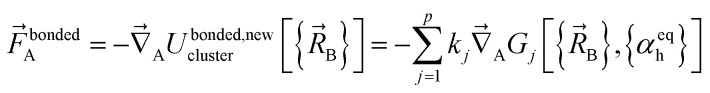


Comparing eqn (58)–(63) reveals that64
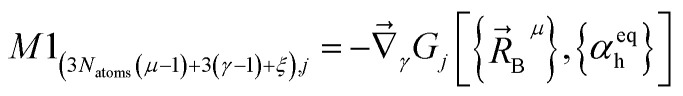
where *μ* is the geometry number. *ξ*= 1, 2, 3 for *x*, *y*, *z* force components of atom *γ*, respectively. {*α*^eq^_h_} are the equilibrium values of the internal coordinates. The derivatives in eqn (64) can be computed either numerically (using finite difference approximation) or analytically.

For the predictor variables in rotatable dihedrals fitting, as discussed in Sections 2.2 and 7.1, we can have up to seven active modes for each rotatable dihedral. We first determine which dihedral modes are active for each rotatable dihedral type using the method described in Section 7.1. Since the forces are zero at the equilibrium geometry, the no-intercept linear regression model is used. Therefore, to be able to use a no-intercept model, we centered the observation variable (*i.e.*, the QM-computed energy) for rotatable dihedral torsions by subtracting the average value as described in ESI Section S3† to construct the matrix **M2**. Please see ESI Section S3† for a more detailed description of linear equations for flexibility parameters.

### 7.3 Defining SSE, SST, *R*-squared, and RMSE

Optimizing the flexibility model (*i.e.*, force constant values) to the combined training dataset yields the matrix *β* containing optimized force constants values. As explained in the following section, some of the force constants may have values equal to zero (aka ‘eliminated’).

To assess the predictive power of our flexibility model, we employed two metrics: the *R*-squared (aka ‘goodness of fit’) and the root-mean-squared error (RMSE). *R*-Squared is calculated using the formula:65
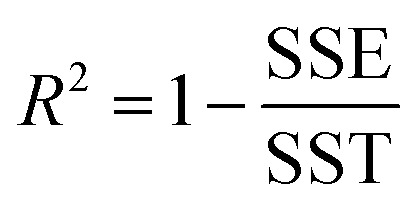


The sum of squared errors (SSE) and sum of squares total (SST) for rotatable dihedrals fitting and forces fitting are defined as follows:66
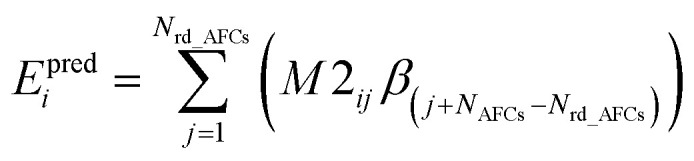
67
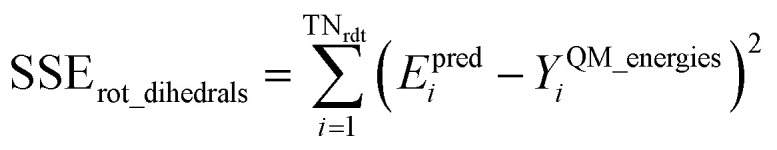
68
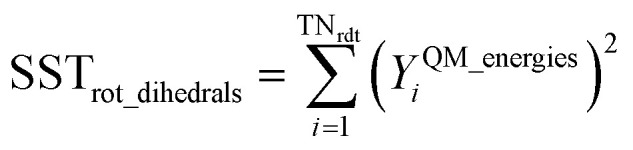
69
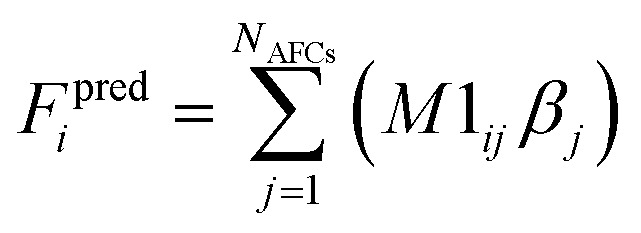
70
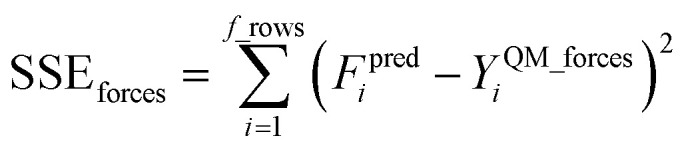
71
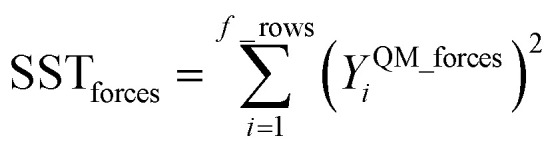
*F*^pred^_*i*_ is the *i*th predicted force component. RMSE_rot_dihedrals_and RMSE_forces_ are computed as follows:72
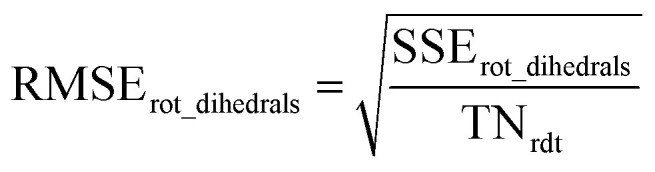
73
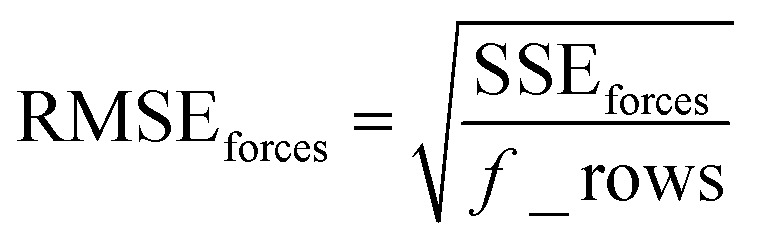


Applying eqn (65), (70), (71), and (73) to the forces training dataset yields SSE_forces_training_, SST_forces_training_, *R*_forces_training_^2^, and RMSE_forces_training_. Applying eqn (65) to the rotatable dihedrals dataset yields *R*_rot_dihedrals_^2^. If the MOF has rotatable dihedrals, both *R*_rot_dihedrals_^2^ and *R*_forces_training_^2^ are computed. If the MOF has no rotatable dihedrals, then **M2**, SSE_rot_dihedrals_, SST_rot_dihedrals_, and *R*_rot_dihedrals_^2^ are not computed.

Whether or not the MOF has rotatable dihedrals, the validation dataset contains quantum-mechanically-computed forces for the optimized ground-state geometry plus new AIMD-generated geometries (we included the material's optimized ground-state reference geometry in both the training and validation datasets in order to validate that the trained forcefield yields zero-valued atom-in-material forces for this optimized geometry). The AIMD-generated geometries for the validation dataset are taken from separate AIMD runs than those used to prepare the forces training dataset. When computing statistics for the validation dataset, the *β* matrix (*i.e.*, set of force constants values) applied is the one that was previously optimized to the combined training dataset. For the validation dataset, the model-predicted forces follow eqn (69), where **M1** is constructed by applying eqn (64) to the validation dataset geometries. Applying eqn (65), (70), (71), and (73) to the forces validation dataset yields SSE_validation_, SST_validation_, *R*_validation_^2^, and RMSE_validation_. When analyzing the validation dataset, *f*_rows = 3*N*_atoms_*N*_validation_geoms_ is the number of force components in the validation dataset, where *N*_validation_geoms_ is the number of geometries in the validation dataset.

### 7.4 Embedded feature selection using the LASSO method with bounds on some force constants

A common issue in fitting parameters using ordinary least squares regression is multicollinearity. When two or more predictors in the linear model are highly correlated to each other or not linearly independent, this is known as multicollinearity.^114^ In this case, the Gram matrix **M**^**T**^**M** contains one or more singular values that are zero or close to zero.^114^ In this case, there are more predictors than needed to build the model. Embedded feature selection is needed to select an appropriate subset of predictors for model building. The LASSO method solves these two problems (*i.e.*, multicollinearity and embedded feature selection) by adding a L_1_-norm penalty term to the least-squares loss function.^65,66^ Specifically, the LASSO method zeroes out coefficients of unnecessary predictors; this reduces the number of predictors required to build a useful model.^65,66,114^ Alternatively, ridge regression^115^ solves the multicollinearity problem by adding a L_2_-norm penalty term to the least-squares loss function; however, ridge regression does not solve the embedded feature selection problem. Accordingly, we decided to use the LASSO method rather than ridge regression to optimize the force constants for flexibility interactions.

We used the Python version of the glmnet package^116^ which minimizes the following loss function:74
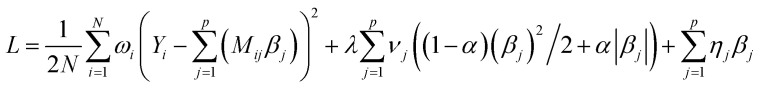
Here, *i* is the observation index and *j* is the predictor variable index. For LASSO regression, *α* = 1. In contrast, *α* = 0 for ridge regression. We used LASSO regression. *N* is the number of observations (*i.e.*, the number of rows in matrix **M**) and *p* is the number of predictor variables (*i.e.*, the number of columns in matrix **M**). *λ* ≥ 0 is the regularization parameter. *ω*_*i*_ is the observation weight. *ν*_*j*_ is the *j*th variable's penalty factor. *η*_*j*_ is the Lagrange multiplier to enforce bounds on the model parameter *β*_*j*_. The optimized model parameter values, {*β*_*j*_}, minimize the loss function's value:75
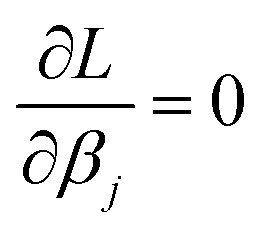


If the MOF contains any rotatable dihedrals, we defined the training dataset observation weights as follows.76
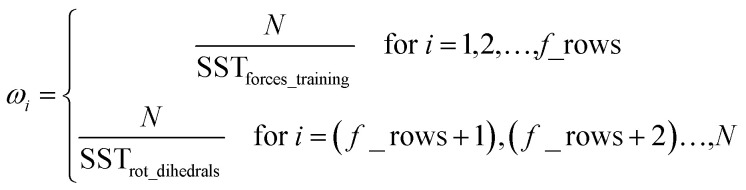
(By convention, Glmnet_Python rescales the observation weights so that they sum to *N*.^117^ This does not change their ratios, the optimized {*β*_*j*_}, the *R*-squared values, or the RMSE (as defined in eqn (72) and (73)).) SSE_forces_ (eqn (70)) and SSE_rot_dihedrals_ (eqn (67)) can be rewritten as follows:77
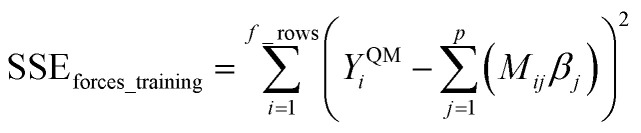
78
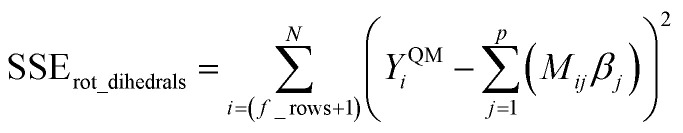
By defining *ω*_*i*_ as described in eqn (76), setting *α* = 1, and by substituting eqn (77) and (78) together with the *R*-squared definition (eqn (65)) into (74), the loss function can be rewritten as follows:79
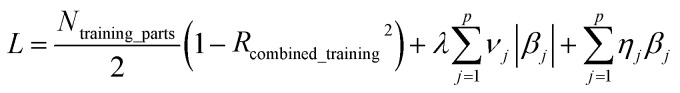
where80
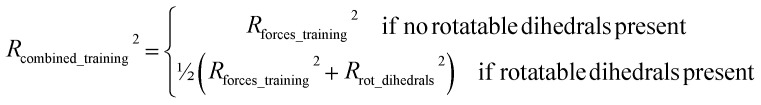
*N*_training_parts_ is the number of separate parts in the training dataset for which *R*-squared values are computed. Specifically, *N*_training_parts_ = 1 if no rotatable dihedrals are present, and *N*_training_parts_ = 2 if any rotatable dihedrals are present. Note that *R*_combined_training_^2^ is the average of *R*-squared values for the training parts. Examining eqn (79) and (80), this choice for *ω*_*i*_ maximizes the sum of *R*-squared values for the forces training and rotatable dihedrals training datasets subject to the applied constraints.

We defined the *j*th predictor variable's penalty factor as follows:81
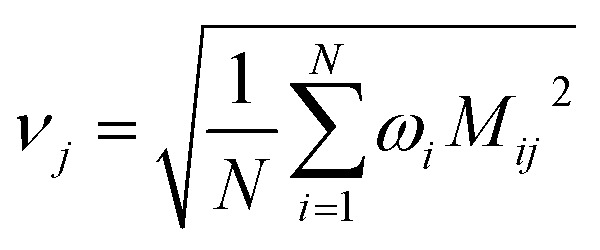
(By convention, Glmnet_Python rescales the penalty factors so that they sum to *p*.^117^ This does not change their ratios, the optimized {*β_j_*}, the *R*-squared values, or the RMSE (as defined in eqn (72) and (73)).) We chose this definition, because it makes the model invariant to the choice of measurement units. For example, whether the distance between two atoms is measured in either Angstroms (Å) or bohrs, the optimized model still gives the same optimized {*β_j_*}, *R*-squared values, and RMSE (as defined in eqn (72) and (73)). Proof: (1) *M*_*ij*_*β*_*j*_ has the same units as *Y*^QM^_*i*_. (2) Examining eqn (76)*ω*_*i*_ has the same units as 1/(*Y*^QM^_*i*_)^2^. (3) Thus it follows that82
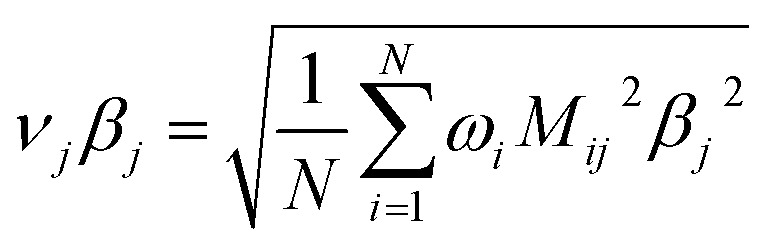
is dimensionless. (4) Since |*β*_*j*_| has the same units as *β*_*j*_, it follows that the penalty factors defined by eqn (81) give optimized *R*-squared values (*i.e.*, *R*_forces_training_^2^, *R*_rot_dihedrals_^2^, and *R*_combined_training_^2^) that are independent of the measurement units.

lb_*j*_ and ub_*j*_ provide a lower bound and an upper bound on the model parameter *β*_*j*_:

83 lb_*j*_ ≤ *β*_*j*_ ≤ ub_*j*_

We used no upper bound (*i.e.*, ub_*j*_ → infinity). We used the lower bound of zero to constrain all bond stretches, UB stretches, angle bends, non-rotatable/hindered dihedral torsions, and ADDT linear torsion modes to be non-negative. This guarantees that displacements along those internal coordinates away from the equilibrium (aka optimized) structure leads to energy increases in the model forcefield. For rotatable dihedrals, the lower bound was set to zero iff only one mode was smart selected, because a single rotatable dihedral mode cannot exhibit competing signs. When a rotatable dihedral has more than one mode that is smart selected, no lower bound on the corresponding force constants was imposed (*i.e.*, lb_*j*_ was set to minus infinity) because some modes having a negative force constant could be compensated by other modes having a positive force constant to still produce an energy increase when the geometry is displaced away from the equilibrium structure. The bond–bond cross terms determine the relative energy of symmetric stretches compared to asymmetric stretches. The lb_*j*_ was set to minus infinity for the bond–bond cross terms, because depending on the situation symmetric stretches may be either higher or lower in energy than asymmetric stretches.

By default, the glmnet package assigns and uses a series of 100 distinct *λ* values in a geometric progression from *λ*_min_ to *λ*_max_.^116^ As an input to the glmnet function, the user specifies the desired ratio of *λ*_min_/*λ*_max_.^116^ We used the ratio 10^−5^. Glmnet automatically determines the *λ*_max_ value, which is the smallest value of *λ* that sets all force constants to zero.^116^ If the smallest value of *λ* is too close to zero, then it will not sufficiently regularize the multicollinearity problem.^65,66^ Therefore, we must use a *λ*_min_ that is small but not too close to zero.^65,66^

Next, we tried to find the best *λ* among the generated *λ* sequence to have our final linear model parameters. Each *λ* will give us a set of model parameters and by increasing the *λ* value we will have lower number of non-zero parameters and smaller *R*-squared. To generate a selection criterion, we first need to estimate the number of degrees of freedom in the physical system. For a material containing *N*_atoms_ in the reference unit cell in the presence of optional externally applied fields, there are 3*N*_atoms_ degrees of freedom in the atomic positions. In the absence of externally applied fields, this may be reduced by 0 to 5 degrees of freedom due to center-of-mass translational symmetry and/or rotational symmetry about the center of mass. However, the precise reduction in degrees of freedom depends on whether the system is periodic or nonperiodic, whether the unit cell parameters and symmetry can be changed, and whether a molecule is linear or nonlinear or monoatomic. For simplicity, we neglect this degrees of freedom reduction (due to the absence of externally applied fields) and simply use the 3*N*_atoms_ degrees of freedom.

A force constant should be kept in the flexible forcefield iff excluding it would increase the SSE by more than half the formal amount per degree of freedom. Therefore, we used the following test to identify *λ*_best_:84
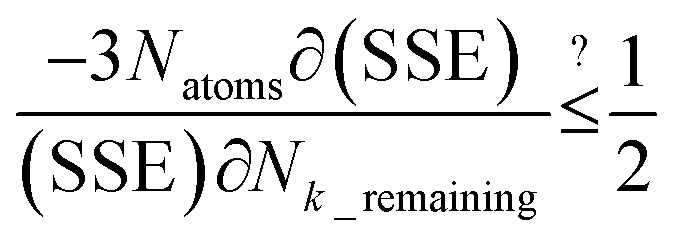
*N*_*k*_remaining_ is the number of non-zero parameters in the model (the change in SSE formally corresponding to one degree of freedom corresponds to the left-hand side of eqn (84) being equal to one). Substituting eqn (65) into (84) gives:85
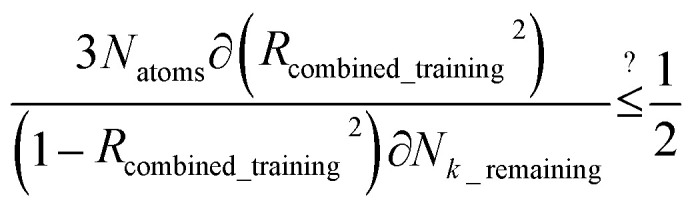
Note that SST does not depend on *N*_*k*_remaining_. Eqn (85) was evaluated using finite difference approximation:86
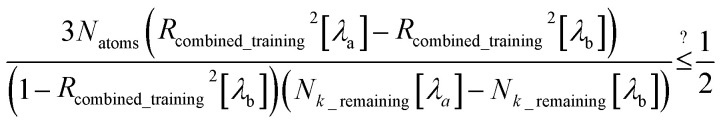


Therefore, we started with the smallest *λ* in the *λ* sequence, which also corresponds to the largest *R*-squared with highest number of non-zero parameters. As mentioned earlier, as *λ* increases, the *R*-squared value tends to decrease, while the number of non-zero parameters may remain the same. If we have the same number of non-zero parameters for different *λ* values, we choose the smallest *λ* among these that yields the highest *R*-squared. Next, we compare the results obtained with our selected *λ* value with the next higher *λ* value, which has a different (lower) number of non-zero parameters. We also ensure that the second chosen *λ* value generates the highest *R*-squared among *λ* values having the same number of non-zero parameters. Therefore, we proceed with our test until we reach a step where the condition defined in eqn (86) is no longer satisfied. If *λ*_a_ represents the smaller *λ* and *λ*_b_ is the *λ* value that violates the test condition, we identify *λ*_a_ as our *λ*_best_. Then, using *λ*_best_, we generate our linear model parameters (*i.e.*, the optimized force constant values). ESI Section S4† contains the python function we employed to identify *λ*_best_.

In the glmnet package,^116^ we also specified the following options: family = ‘Gaussian’ (this corresponds to linear least-squares fitting), thresh = 10^−10^ (convergence threshold for updating model parameters in each optimization iteration), standardize = False (logical flag for independent variables standardization), standardize_resp = False (logical flag for response variables standardization), intr = False (logical flag to add or remove intercept from linear model; assigning “False” to this parameter means we are using a no-intercept linear model).

## 8. Results

### 8.1 Classifying the MOFs into four quadrants

As explained in Sections 5.4.2 and 5.4.4, we classified each dihedral as non-rotatable, rotatable, hindered, or linear. Since all MOFs contain at least one dihedral, each MOF can be classified into a quadrant depending on whether it contains any rotatable dihedrals and/or any hindered dihedrals. Quadrant 1 includes MOFs having no rotatable dihedrals and no hindered dihedrals; each MOF in quadrant 1 contains only non-rotatable and/or linear dihedrals. Each MOF in quadrant 2 contains at least one hindered dihedral, no rotatable dihedrals, and any number of non-rotatable and/or linear dihedrals. Each MOF in quadrant 3 contains at least one rotatable dihedral, no hindered dihedrals, and any number of non-rotatable and/or linear dihedrals. Each MOF in quadrant 4 contains at least one hindered dihedral and at least one rotatable dihedral and any number of non-rotatable and/or linear dihedrals.

A dataset comprising 116 MOFs successfully passed the crystal geometry verification procedure outlined in Section 4. As described in Section 6, we performed quantum chemistry calculations on these MOFs. No MOFs were excluded from the dataset during or after flexibility parameters optimization. For this dataset, Table 1 lists the number of MOFs in each quadrant.

**Table 1:** Quadrant table classifying MOFs based on whether they contained any rotatable dihedrals or hindered dihedrals

	No rotatable dihedrals	At least one rotatable dihedral
No hindered dihedrals	Quadrant 1: 78 MOFs	Quadrant 3: 35 MOFs
At least one hindered dihedral	Quadrant 2: 1 MOFs	Quadrant 4: 2 MOFs

### 8.2 MOF sizes and chemical element compositions

**Fig. 14:**
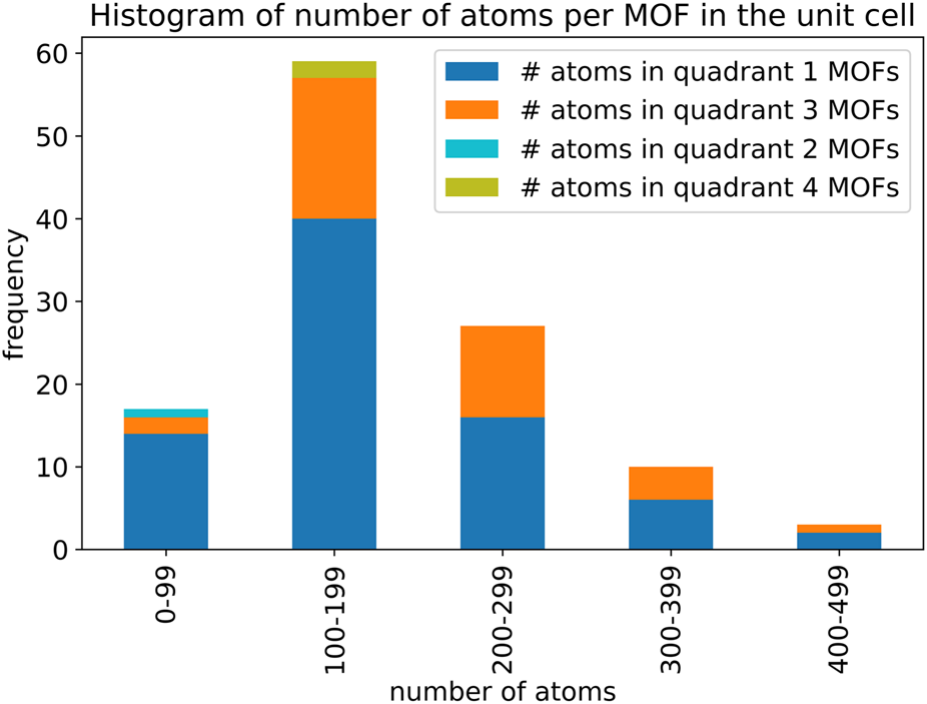
A stacked histogram showing the number of MOFs with different unit cell sizes as quantified by the number of atoms per unit cell. The total number of MOFs was 116, and we optimized flexibility parameters for all these.

Fig. 14 shows the distribution of unit cell sizes as quantified by the number of atoms per unit cell. The most prevalent range was 100–199 atoms per unit cell. The largest and smallest MOFs in our investigation contained 496 and 38 atoms per unit cell, respectively.

**Fig. 15:**
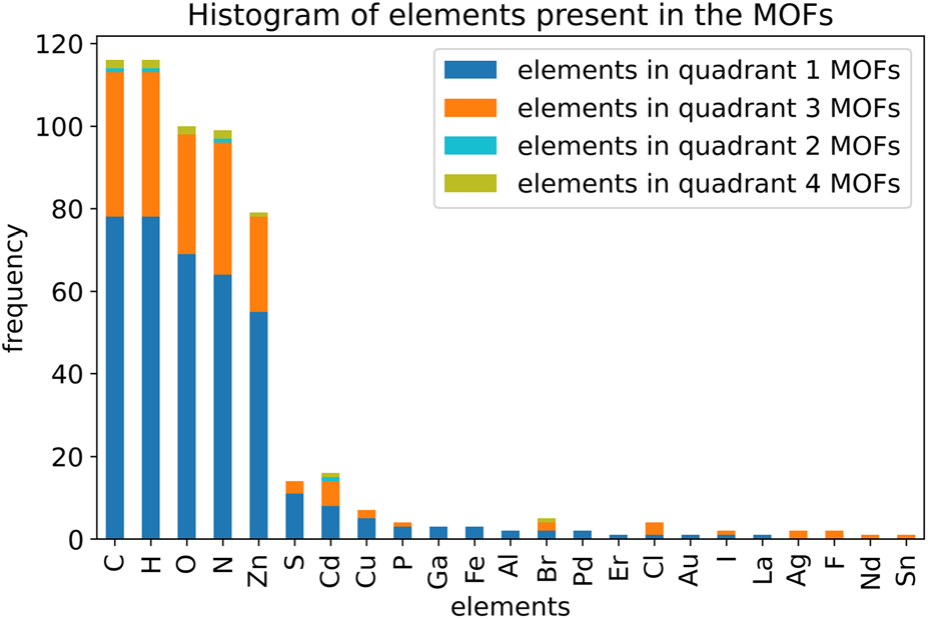
A stacked histogram showing the number of MOFs containing various chemical elements (If a particular MOF had more than one atom of a particular chemical element, this counted only once. For example, a MOF with six Zn atoms counts as 1 towards the Zn bin.). Elements not shown in this graph were not contained in any of these 116 MOFs.

We identified a total of 23 distinct chemical elements present within these structures. Fig. 15 shows the frequency of occurrence for each of these 23 elements across the 116 MOFs. Every MOF within the dataset had both carbon (C) and hydrogen (H) atoms.

### 8.3 Bond, angle, and dihedral types

In this section, all of the plotted data corresponds to the final internal coordinates list that follows all adjustments, such as removal of angles in 3- and 4-membered rings and dihedral pruning. Moreover, all MOFs in the relevant quadrants were included in these plots.

**Fig. 16:**
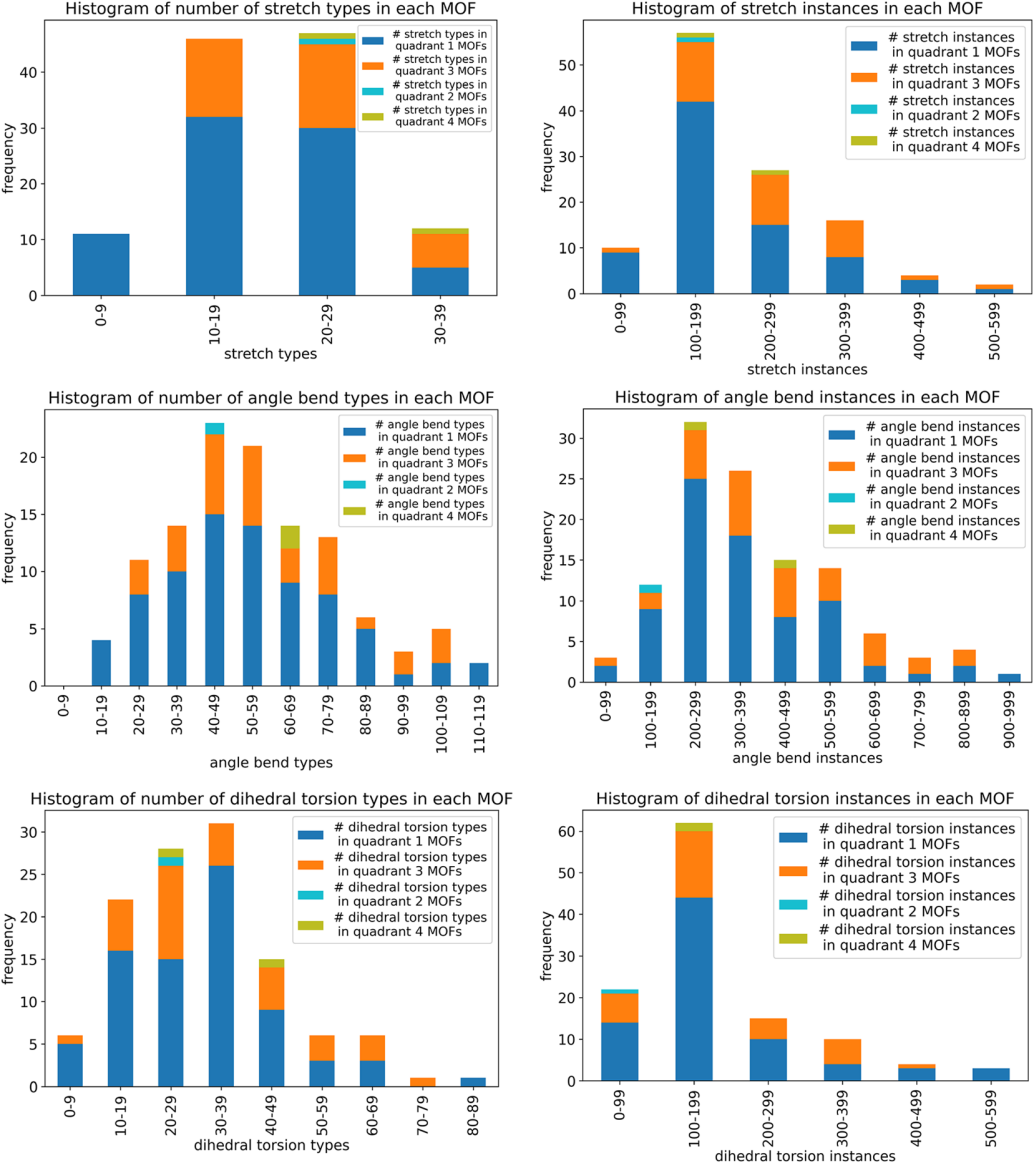
Stacked histograms showing the number of MOFs containing various numbers of active internal coordinate types (left panels) and active internal coordinate instances (right panels). The top panels are for bond and UB stretches. The middle panels are for angle bends. The bottom panels are for dihedral torsions after dihedral pruning.

Fig. 16 shows stacked histograms of the number of MOFs containing various numbers of active internal coordinate types (left panels) and active internal coordinate instances (right panels). The number of active angle bend types was usually larger than the number of active bond plus UB stretch types. After dihedral pruning, the number of active angle bend types was usually larger than the number of active dihedral torsion types. Overall, the numbers of internal coordinate instances per MOF were much larger than the numbers of internal coordinate types per MOF. This clearly demonstrates the effectiveness of grouping similar instances into the same type to reduce the number of force constant values that have to be optimized.

Section S7 of the ESI† contains histograms showing the distribution of number of internal coordinate instances per internal coordinate type. The total number of stretch, angle, and dihedral types was 2327, 6358, and 3740, respectively. These stretches included both the bond stretches and the UB stretches. These distributions peaked at 6–8 instances per stretch type, 4–5 instances per angle type, and 4–5 instances per dihedral type.

**Fig. 17:**
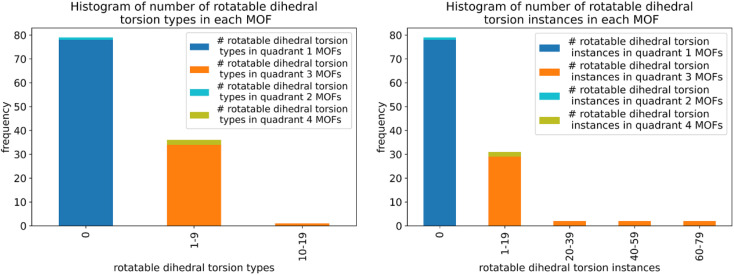
Histograms showing the distribution of the number of rotatable dihedral types per MOF (left panel) and the distribution of the number of rotatable dihedral instances per MOF (right panel). These results are after dihedral pruning.

The histogram in the left panel of Fig. 17 shows the distribution of rotatable dihedral types per MOF after dihedral pruning. Of the 116 MOFs we studied, 79 had no rotatable dihedrals corresponding to MOFs in quadrants 1 and 2. The other 37 MOFs had at least one rotatable dihedral and represent quadrants 3 and 4. Each of the 37 MOFs had fewer than 20 rotatable dihedral types after pruning, with most MOFs in these quadrants having between 1 and 9 rotatable dihedral types. The histogram in the right panel of Fig. 17 shows the distribution of rotatable dihedral instances per MOF after dihedral pruning. The 37 MOFs in quadrants 3 and 4 had fewer than 80 rotatable dihedral instances after pruning, with most MOFs in these quadrants having between 1 and 19 rotatable dihedral instances.

Table 2 lists the number of dihedral instances, number of dihedral types, and the number of MOFs that used five different kinds of dihedral torsion model potentials: CADT non-rotatable/hindered, ADDT non-rotatable/hindered, CADT rotatable, ADDT rotatable, and ADDT linear. These model potentials are explicitly defined in Section 2.2 above. Examining Table 2, the vast majority of active dihedrals used the CADT non-rotatable/hindered model potential. This is the simplest and computationally cheapest among the five model potentials. The ADDT rotatable model potential is the most computationally expensive among the five model potentials, and it was used the least often. Moreover, the CADT rotatable and ADDT rotatable model potentials are used along with smart selection to ensure the computational cost is minimized by including only important torsion modes. Overall, this strategy provides an extremely general, concise, and cost-effective approach. The following sections show this strategy is also extremely accurate.

**Table 2:** The frequency of occurrence of CADT non-rotatable/hindered, ADDT non-rotatable/hindered, CADT rotatable, ADDT rotatable, and ADDT linear dihedral torsion model potentials. These results are for all 116 MOFs after dihedral pruning

Dihedral torsion model potential	# Dihedral instances	# Dihedral types	# MOFs
CADT non-rotatable/hindered	22 010	3287	116
ADDT non-rotatable/hindered	2599	343	78
CADT rotatable	623	95	37
ADDT rotatable	12	3	2
ADDT linear	124	12	5

### 8.4 Internal coordinate redundancy

The number of degrees of freedom for atom-in-material motions in a crystal having fixed unit cell size and shape can be derived as follows. Each atom can be moved along *x*, *y*, and *z* directions; this gives 3*N*_atoms_ motions. In the absence of externally applied fields, the total potential energy is unchanged by uniform translation so this means 3 motions do not affect the material's potential energy function. Hence there are (3*N*_atoms_ − 3) independent degrees of freedom in the material's internal coordinates when keeping the unit cell's size and shape rigid.

The internal coordinate redundancy (ICR) is therefore defined as87
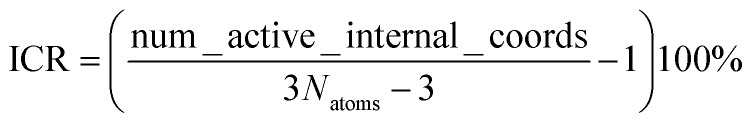
In eqn (87), num_active_internal_coords is the number of active internal coordinates instances; that is, the number of internal coordinates instances that are used to construct any interactions in the flexibility model. If the ICR is negative, this means the active internal coordinates list contains fewer instances than there are degrees of motion freedom. If the ICR is positive, this means the active internal coordinates list contains more instances than there are degrees of motion freedom.

What is the ‘best’ ICR value? At first, we may think that zero ICR is the ‘best’ value, because it means there are exactly the same number of instances in the active internal coordinates list as there are degrees of motion freedom; however, this means the flexibility model does not self-correct for any oversimplifications in the model potentials. If ICR is greater than zero, then this provides some malleability for the model to partly self-correct for any oversimplifications in the model potentials. However, too much redundancy is also a disadvantage because it means the flexibility model contains a relatively large number of interaction terms, and this leads to relatively high computational costs when using the model. Therefore, the ‘best’ ICR value is a modest positive percentage (*e.g.*, ∼20–60%) that provides some malleability for the flexibility model to partly self-correct for any oversimplifications in the model potentials while still keeping the computational costs relatively low.

**Fig. 18:**
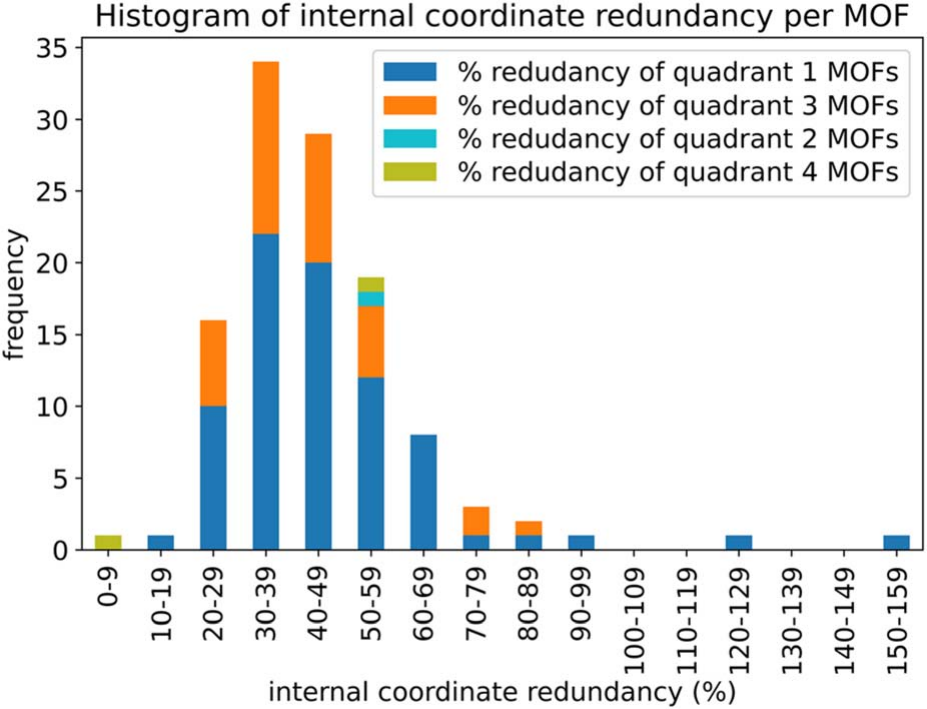
Histogram showing the internal coordinate redundancy after applying our protocol to 116 MOFs. The plotted data corresponds to the final internal coordinates list that follows all adjustments, such as removal of angles in 3- and 4-membered rings and dihedral pruning.

Fig. 18 shows a stacked histogram of ICR for all 116 MOFs after dihedral pruning. When computing these values, the active list of internal coordinates instances included the bond stretches, the angles not in 3-membered or 4-membered rings, UB stretches composed from the diagonals of 4-membered rings, and the dihedrals active after pruning. Examining Fig. 18, 30–39% redundancy was the most popular interval. When applying our protocol, the ICR was less than zero for none of these 116 MOFs. Fig. 18 shows that our protocol yielded 20–69% redundancy for the vast majority of systems investigated. Our protocol yielded ICR larger than 100% for only 2 of the 116 MOFs, and ICR values of 0–19% for only 2 of the 116 MOFs. Overall, this shows our protocol worked well.

### 8.5 Smart mode selection for rotatable dihedrals

**Fig. 19:**
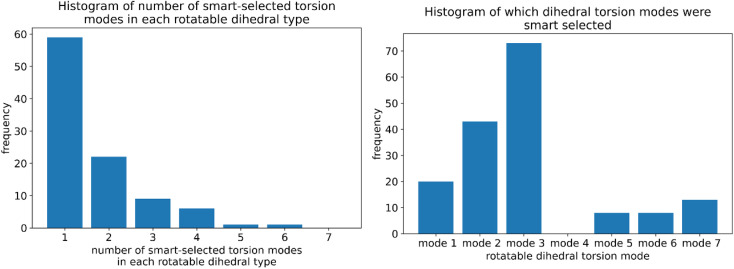
Left panel: Histogram showing the number of smart-selected torsion modes in each rotatable dihedral type. Right panel: Histogram showing which rotatable dihedral modes were smart selected.

All results in this section are for calculations following dihedral pruning. The left panel of Fig. 19 is a histogram showing the number of smart-selected torsion modes in each rotatable dihedral type. For ∼60% of the rotatable dihedral types, smart selection yielded a model potential containing one torsion mode per rotatable dihedral type. For example, molecular symmetry reveals the torsion potential in ethane is closely described by the single mode *G*^CADT^_mode_3_[*ϕ*_ABCD_], and torsion modal analysis confirms this.^50^ If ethane's rotatable dihedral was a rotatable dihedral type in a MOF, it would be plotted in the bar labeled ‘1’ in the left panel of Fig. 19, because only a single mode is required to describe this torsion potential. Smaller percentages of rotatable dihedral types required two (∼22%), three (∼9%), four (∼6%), five (∼1%), six (∼1%), or seven (∼0%) torsion modes per rotatable dihedral type.

The right panel of Fig. 19 is a histogram showing which rotatable dihedral modes were smart selected. Mode 3 was the most popular mode, and it appeared in the smart-selected torsion potential for 73 (∼74%) of the rotatable dihedral types. Mode 2 was the second-most popular followed by mode 1 as the third-most popular torsion mode for rotatable dihedral types. Modes 5, 6, and 7 were less popular but appeared in the smart-selected torsion potential for significant numbers of rotatable dihedral types. Mode 4 was the least popular and was not significant in any of the 116 MOFs analyzed in this work.

Notably, the torsion sine modes (*i.e.*, modes 5, 6, and/or 7) cannot be the only smart-selected modes for a rotatable dihedral type. This follows directly from the observation that the torsion sine modes are odd functions of (*ϕ* − *ϕ*_eq_); these modes increase the potential energy for a small displacement of *ϕ* in one direction away from *ϕ*_eq_ while decreasing the potential energy for a small displacement in the opposite direction. In contrast, the torsion cosine modes (*i.e.*, modes 1 to 4) are even functions of (*ϕ − ϕ*_eq_); these modes increase the potential energy for small displacements of *ϕ* in either direction away from *ϕ*_eq_. Since *ϕ* = *ϕ*_eq_ is a local energy minimum, it directly follows that the smart-selected torsion modes for a rotatable dihedral type must include at least one torsion cosine mode.

### 8.6 Regularized linear least squares fitting results

#### 8.6.1 Comparing results before to after dihedral pruning

**Fig. 20:**
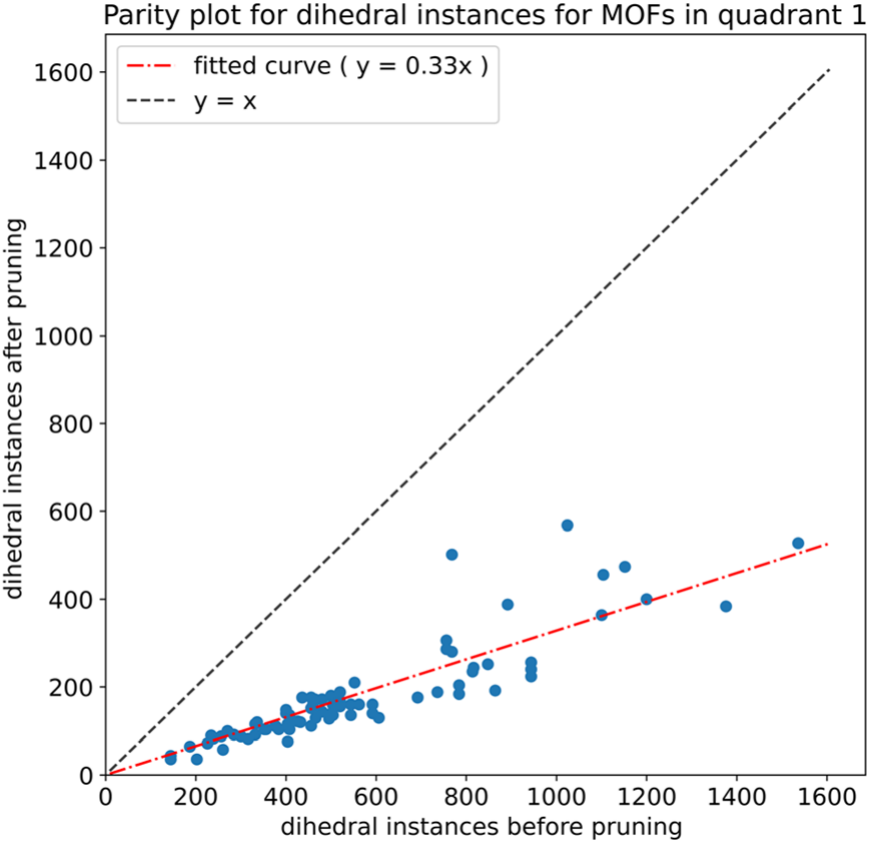
Parity plot showing the number of dihedral instances after pruning compared to before pruning in quadrant 1 MOFs.

This section contains several plots comparing performance before dihedral pruning to performance after dihedral pruning for all of the MOFs in quadrant 1. As shown in Fig. 20, dihedral pruning eliminated approximately two-thirds of the dihedral instances leaving the other one-third after pruning. Except for a couple of outliers, more than half of the dihedral instances for each MOF were consistently eliminated *via* dihedral pruning.

**Fig. 21:**
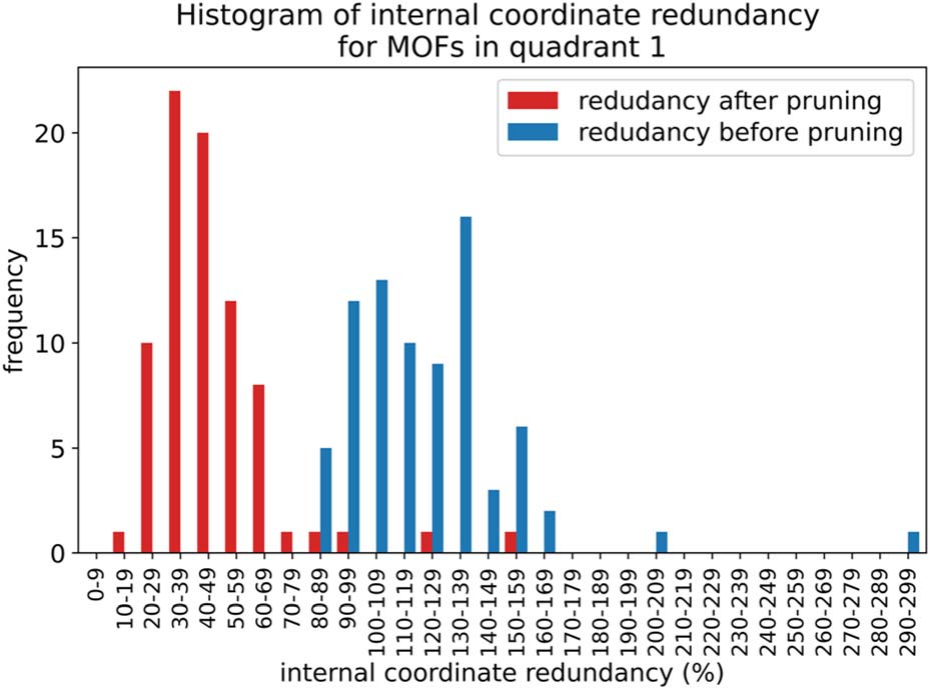
The histograms for internal coordinate redundancy (%) after pruning (red) and before pruning (blue) for quadrant 1 MOFs.

As shown in Fig. 21, dihedral pruning consistently reduced the internal coordinate redundancy percentage. Before dihedral pruning, the internal coordinate redundancy was >100% for most (but not all) MOFs. After dihedral pruning, the internal coordinate redundancy was 20–69% for most (but not all) MOFs.

In this manuscript, we report separate least-squares regression results using individual equilibrium values and average equilibrium values. Using ‘individual equilibrium values’ means that each instance of each active internal coordinate uses flexibility terms employing the specific equilibrium value for that particular instance as taken from the material's quantum-mechanically-computed ground-state geometry (as stated previously, we held the unit cell's size and shape fixed at the experimental values). Using ‘average equilibrium values’ means the bond lengths, angle values, or dihedral absolute values were averaged over all instances belonging to each active internal coordinate type. This yielded an ‘average equilibrium value’ for each internal coordinate type that was subsequently used as the equilibrium value in all flexibility terms involving that internal coordinate type.

Why do we report separate results using the ‘individual equilibrium values’ and the ‘average equilibrium values’ instead of choosing only one? In our experience, computing both is extremely valuable for diagnostic purposes. Consider a scenario in which *R*-squared values for regression using individual equilibrium values are much higher than *R*-squared values for regression using average equilibrium values. This scenario could indicate a situation in which an internal coordinate type was defined too loosely and included instances that differ too much from each other.

**Fig. 22:**
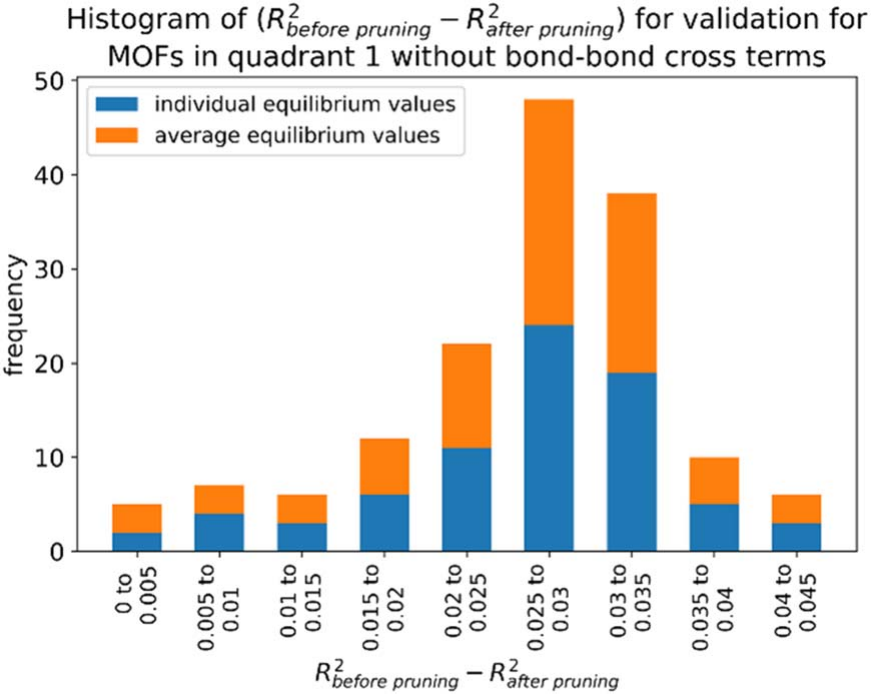
Histogram of difference between *R*-squared before dihedral pruning and *R*-squared after dihedral pruning for MOFs in quadrant 1.

Fig. 22 shows a stacked histogram of the difference between the validation dataset *R*-squared for force constants regression performed using internal coordinate lists without (aka ‘before’) or with (aka ‘after’) dihedral pruning (all *R*-squared and RMSE statistics for the validation datasets in this article used force constants optimized using *λ*_best_ values). Both distributions (*i.e.*, using average and individual equilibrium values) peaked at a *R*-squared difference of 0.025–0.03. Fig. 22 shows dihedral pruning always reduced the *R*-squared values by a small (*i.e.*, 0–0.045) amount.

**Fig. 23:**
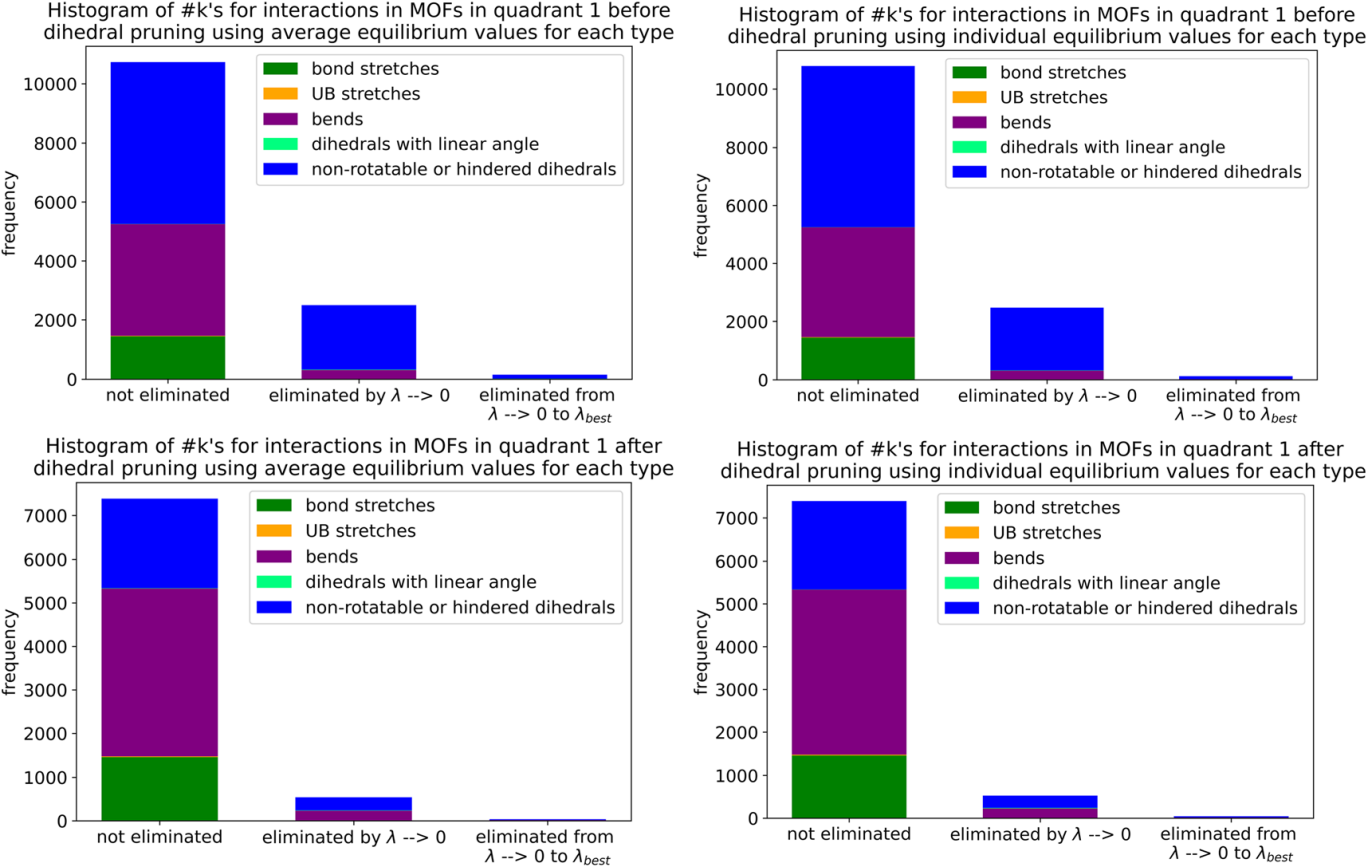
Histograms of force constants eliminated by the bounds or regularization constraints in the LASSO method applied to MOFs in quadrant 1. These histograms show results before dihedral pruning (top panels) and after dihedral pruning (bottom panels) using average (left panels) and individual (right panels) equilibrium values without bond–bond cross terms.

In Fig. 23, histograms present the distribution of number of *k*'s before (top panels) and after (bottom panels) dihedral pruning using average (left panels) and individual (right panels) equilibrium values for each internal coordinate type for MOFs in quadrant 1. Notably, a significant portion (∼30%) of the *k*'s representing non-rotatable or hindered dihedrals were eliminated by the LASSO method in the before dihedral pruning case. In the after dihedral pruning case, the LASSO method removed a smaller portion (∼10%) of non-rotatable or hindered dihedral *k*'s.

**Fig. 24:**
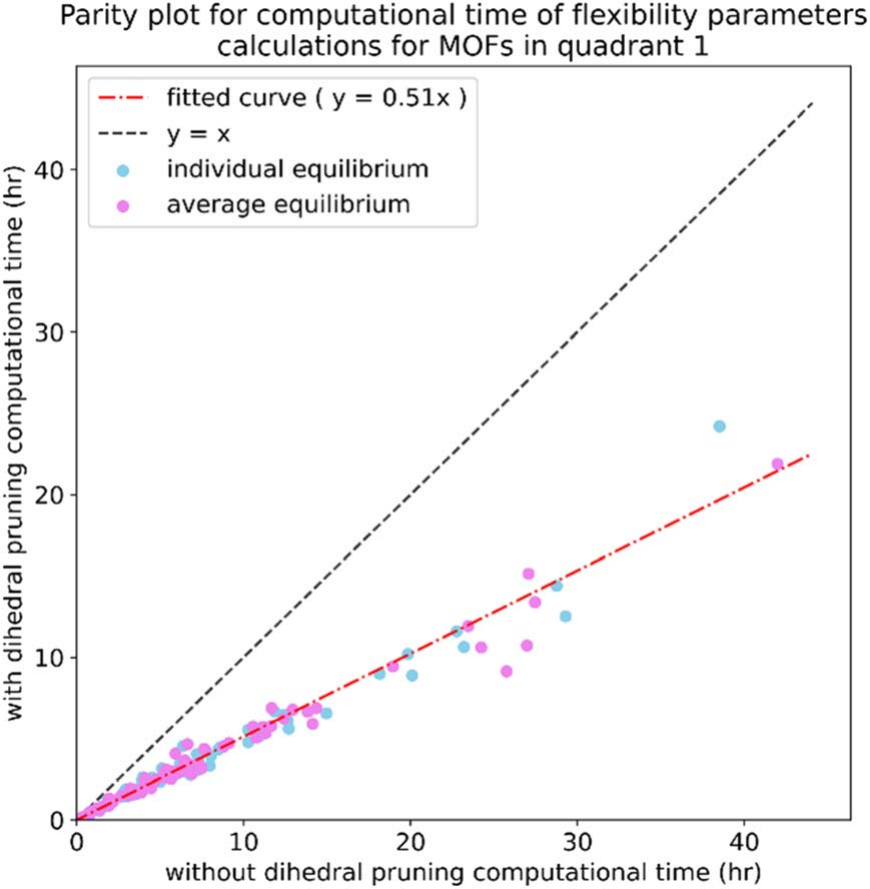
Parity plot showing computational time for flexibility parameters calculation when the flexibility model was constructed with dihedral pruning compared to without dihedral pruning. These computational times include optimizing force constant values, computing statistics for the training datasets, and computing statistics for the validation datasets. These computational times do not include the times for quantum chemistry calculations to prepare the training and validation datasets.

As shown in Fig. 24, the computational time for flexibility parameters calculation (using our SAVESTEPS software) was approximately cut in half by dihedral pruning. Overall, the results in this section showed dihedral pruning consistently and substantially reduces the number of active dihedral instances, internal coordinate redundancy, number of force constants that need to be optimized, and the computational time, while causing only a small (*i.e.*, 0–0.045) reduction in *R*-squared values. These results clearly show our dihedral pruning protocol was highly effective.

Additional after-pruning LASSO results for MOFs without rotatable dihedrals are shown in Section S8 of the ESI.† Except for the absence of rotatable dihedrals, these results are similar to those presented in the next section for MOFs with rotatable dihedrals.

#### 8.6.2 LASSO results for MOFs with rotatable dihedrals

**Fig. 25:**
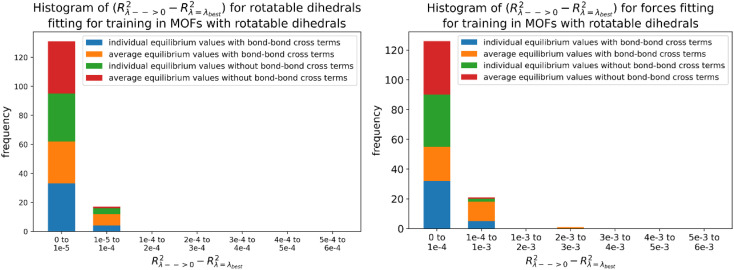
Histogram of difference between *R*-squared for *λ* → 0 and *R*-squared for *λ* = *λ*_best_ for rotatable dihedrals training dataset (left panel) and forces training dataset (right panel) in MOFs belonging to quadrants 3 and 4.

All results in this section are for calculations following dihedral pruning and rotatable torsion mode smart selection. Histograms showing the difference between the *R*-squared value for *λ* → 0 compared to *λ* = *λ*_best_ are shown in Fig. 25. This *R*-squared difference was almost negligible for both the rotatable dihedrals training dataset and the forces training dataset.

**Fig. 26:**
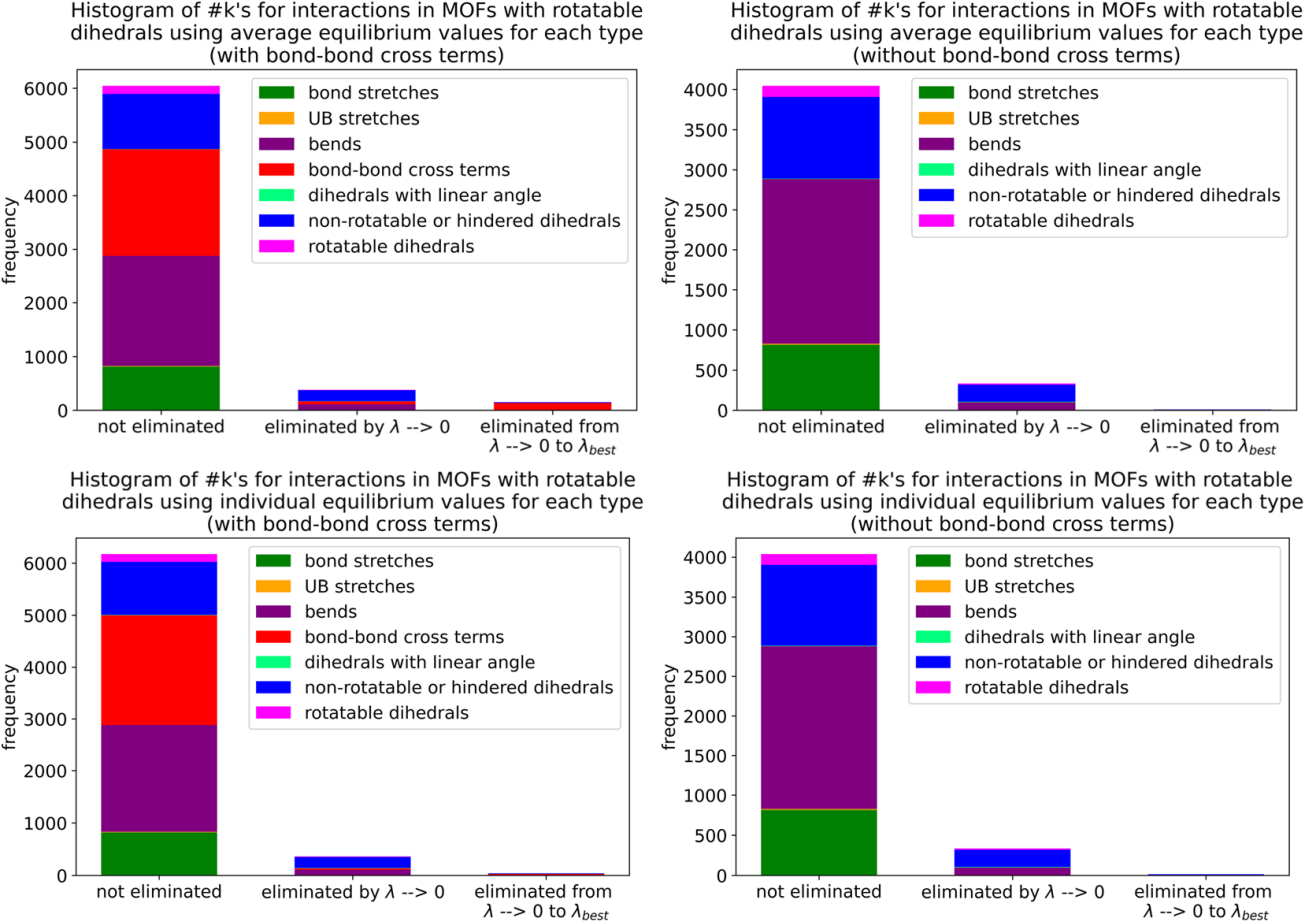
Histograms of force constants eliminated by the bounds or regularization constraints in the LASSO method applied to MOFs in quadrants 3 and 4. These histograms show results after dihedral pruning using average (top panels) and individual (bottom panels) equilibrium values with bond–bond cross terms (left panels) and without bond–bond cross terms (right panels).

Fig. 26 shows the number of force constants eliminated by the LASSO method for *λ* → 0 and the additional number that were eliminated when increasing *λ* to *λ*_best_. Some of the k's eliminated for *λ* → 0 may have been eliminated by the non-negative bounds placed on stretches, bends, and single-mode torsions, while others may have been eliminated due to linear dependencies between the flexibility terms. The *k*'s eliminated when increasing *λ* to *λ*_best_ also correspond to unimportant flexibility terms that contribute negligibly to the model's accuracy. Results are presented for calculations with and without bond–bond cross terms.

Examining Fig. 26, the flexibility models containing bond–bond cross terms had approximately 50% more force constants on average than the flexibility models without bond–bond cross terms. A small percentage of the bond–bond cross terms were eliminated by the LASSO method. Accordingly, including bond–bond cross terms increases the computational costs to use the flexibility model.

**Fig. 27:**
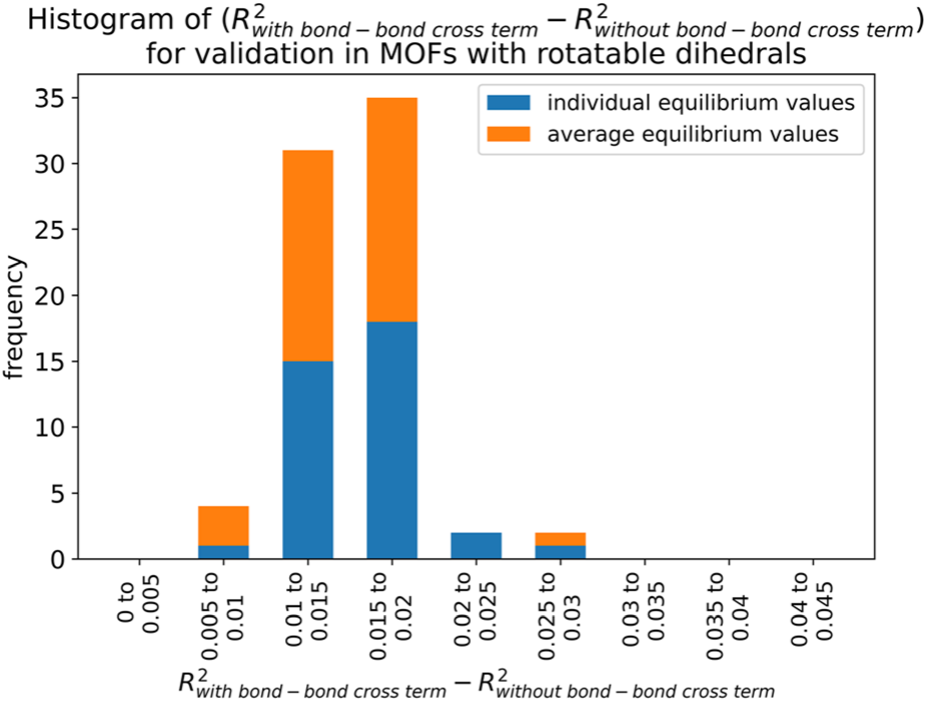
Histogram of difference between *R*-squared with bond–bond cross terms and *R*-squared without bond–bond cross terms for the validation dataset in MOFs from quadrants 3 and 4.

All subsequent results in this section used *λ*_best_. Because including bond–bond cross terms resulted in only a small improvement in *R*-squared value (see Fig. 27), this suggests including bond–bond cross terms is not essential to obtain useful flexibility models for these MOFs.

In this article, each raincloud plot contains a box plot below the kernel density plot (‘cloud’). All box plots in this article have the following features. The middle line is the median. The box encloses the 2nd and 3rd quartiles. The whiskers mark the 5th and 95th percentiles. The diamonds show individual outliers. These raincloud plots also show all of the individual data points as jittered points (‘raindrops’).

**Fig. 28:**
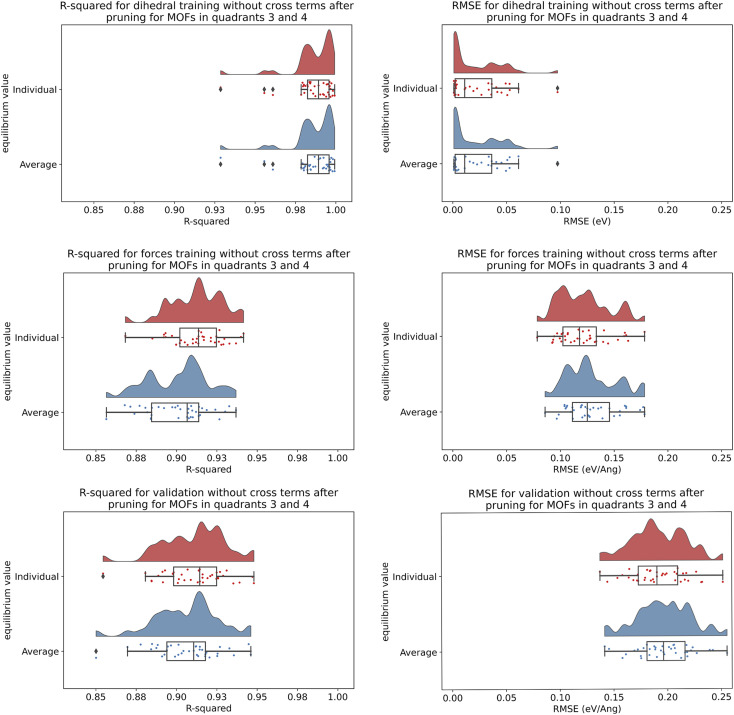
Raincloud plots of *R*-squared (left panels) and RMSE (right panels) for rotatable dihedrals training (top panels), forces training (central panels), and validation (bottom panels) for MOFs in quadrants 3 and 4 without cross terms and after pruning. The red distributions represent the values for individual equilibrium values, while the blue distributions represent the values for average equilibrium values.

Fig. 28 contains raincloud plots showing the distribution of *R*-squared and RMSE values for rotatable dihedrals training, forces training, and validation datasets for MOFs in quadrants 3 and 4 without bond–bond cross terms. All of these *R*-squared values were greater than 0.85, and all of the median *R*-squared values were well above 0.90. The median *R*-squared value was ∼0.99 for the rotatable dihedrals training dataset, and this attests to an outstanding protocol. *R*-Squared values for the forces training and validation datasets were similar, and this demonstrates the flexibility models did not have overfitting. The RMSE values for rotatable dihedrals training, forces training, and validation datasets were reasonable and did not have many outliers.

#### 8.6.3 Performance overview

Table 3 summarizes training and validation statistics for MOFs in quadrants 1 and 3. All of the average *R*-squared values were >0.90, and all of the *R*-squared standard deviations were ≤0.02. This clearly shows our method worked extremely well and performed consistently across different materials.

**Table 3:** Summary of training and validation statistics for MOFs in quadrants 1 and 3. The fourth column indicates whether bond–bond cross (bbc) terms were included. Each numeric entry is the average ± standard deviation

Quadrant	Equilibrium values type	Dihedral pruned?	bbc?	*R*-Squared training rotatable dihedrals	RMSE (eV) training rotatable dihedrals	*R*-Squared training forces	RMSE (eV Å^−1^) training forces	*R*-Squared validation	RMSE (eV Å^−1^) validation
1	Individual	N	N	—	—	0.932 ± 0.013	0.116 ± 0.025	0.936 ± 0.014	0.165 ± 0.017
1	Average	N	N	—	—	0.922 ± 0.016	0.125 ± 0.025	0.931 ± 0.015	0.171 ± 0.017
1	Individual	Y	N	—	—	0.912 ± 0.015	0.133 ± 0.026	0.910 ± 0.017	0.196 ± 0.019
1	Average	Y	N	—	—	0.902 ± 0.018	0.140 ± 0.027	0.905 ± 0.018	0.201 ± 0.019
1	Individual	Y	Y	—	—	0.929 ± 0.011	0.120 ± 0.024	0.928 ± 0.014	0.175 ± 0.017
1	Average	Y	Y	—	—	0.917 ± 0.017	0.129 ± 0.025	0.922 ± 0.016	0.182 ± 0.018
3	Individual	Y	N	0.988 ± 0.010	0.022 ± 0.024	0.913 ± 0.016	0.121 ± 0.024	0.911 ± 0.020	0.192 ± 0.026
3	Average	Y	N	0.988 ± 0.010	0.022 ± 0.024	0.901 ± 0.020	0.128 ± 0.024	0.907 ± 0.020	0.197 ± 0.026
3	Individual	Y	Y	0.988 ± 0.010	0.022 ± 0.024	0.927 ± 0.014	0.111 ± 0.022	0.927 ± 0.018	0.174 ± 0.025
3	Average	Y	Y	0.988 ± 0.010	0.022 ± 0.024	0.915 ± 0.019	0.119 ± 0.023	0.922 ± 0.019	0.180 ± 0.025

The average *R*-squared values for forces training and validation were slightly higher for three conditions:

• When using the individual equilibrium values compared to using average equilibrium values.

• Without dihedral pruning compared to with dihedral pruning.

• With bond–bond cross terms compared to without bond–bond cross terms. However, in all three comparisons the differences in average *R*-squared values were small. This means either individual or average equilibrium values can be used in the flexible forcefield according to the user's preference with little change in accuracy. We also conclude that dihedral pruning effectively reduced the forcefield's computational cost while causing only a small reduction in its accuracy. Finally, bond–bond cross terms do not appear to be essential in most cases.

For each calculation type, the average *R*-squared value for the validation dataset was approximately the same as (but not strictly equal to) the average *R*-squared value for the forces training dataset. This clearly shows our method does not have any over-fitting problems.

The slightly higher average RMSE values for the validation dataset compared to the forces training dataset is due to the inclusion of finite-displacement ‘Hessian’ geometries in the forces training dataset. In each finite-displacement ‘Hessian’ geometry, only one atom is displaced away from its position in the optimized ground-state geometry. In contrast, all atoms are moved in AIMD-generated geometries. Consequently, the average root-mean-squared value of each force component in a finite-displacement ‘Hessian’ geometry is much smaller than for an AIMD-generated geometry. Finite-displacement ‘Hessian’ geometries are included along with AIMD-generated geometries and the optimized ground-state geometry in the forces training dataset, while the validation dataset includes new AIMD-generated geometries and the optimized ground-state geometry. Consequently, the average root-mean-squared value of each force component is smaller in the forces training dataset compared to the validation dataset. Since the *R*-squared values are similar for the forces training and validation datasets, it directly follows that the average RMSE must therefore be slightly higher for the validation dataset compared to the forces training dataset.

The *R*-squared value for rotatable dihedrals training was 0.988 (average) ± 0.010 (standard deviation) irrespective of whether bond–bond cross terms were included and irrespective of whether average or individual equilibrium values were used. This high average *R*-squared value and small standard deviation clearly prove the flexibility model consistently described the rigid torsion scan energies with extremely high accuracy. The RMSE values were small: 0.022 (average) ± 0.024 (standard deviation) eV. In this case, the standard deviation was larger than the average RMSE, because the average RMSE was relatively small in magnitude.

### 8.7 Performance statistics for individual atoms in a material

For further analysis, we selected two MOFs that had the lowest validation *R*-squared values. Among MOFs which had no rotatable dihedrals (*i.e.*, quadrants 1 and 2), OGIBUD had the lowest validation *R*-squared value. Among MOFs with at least one rotatable dihedral (*i.e.*, quadrants 3 and 4), HEBZEV had the lowest validation *R*-squared value. Table 4 summarizes performance statistics for these two MOFs.

**Table 4:** Summary of performance statistics for OGIBUD and HEBZEV. The results displayed outside (inside) parentheses represent outcomes from models optimized with (without) bond–bond cross terms

MOF name	Equilibrium values type	*R*-Squared training rotatable dihedrals	RMSE (eV) training rotatable dihedrals	*R*-Squared training forces	RMSE (eV Å^−1^) training forces	*R*-Squared validation	RMSE (eV Å^−1^) validation
OGIBUD	Individual	—	—	0.8789 (0.8516)	0.1827 (0.2023)	0.8767 (0.8520)	0.2300 (0.2520)
	Average	—	—	0.8330 (0.8160)	0.2146 (0.2253)	0.8504 (0.8332)	0.2533 (0.2675)
HEBZEV	Individual	0.9945 (0.9944)	0.0489 (0.0492)	0.8859 (0.8683)	0.1229 (0.1321)	0.8739 (0.8545)	0.2342 (0.2517)
	Average	0.9944 (0.9943)	0.0492 (0.0495)	0.8714 (0.8565)	0.1305 (0.1379)	0.8675 (0.8501)	0.2401 (0.2554)

To gain further insights, our SAVESTEPS Python code automatically computed and printed the atom-wise *R*-squared and atom-wise RMSE statistics for each atom in the material. These were computed and printed for both the forces training and validation datasets. This helps to identify whether the flexibility model performed poorly for specific atoms in the material.

Raincloud plots help visualize this data. There are four scenarios:

• Scenario # 1: there are no small *R*-squared values and no large RMSE values in these raincloud plots. This means the flexibility model gave small relative errors and small absolute errors when predicting atom-in-material force components for individual atoms in the material.

• Scenario # 2: there are some small *R*-squared values but no large RMSE values in these raincloud plots. This means the flexibility model gave large relative errors but small absolute errors when predicting atom-in-material force components for some of the atoms having small root-mean-squared forces.

• Scenario # 3: there are no small *R*-squared values but there are some large RMSE values in these raincloud plots. This means the flexibility model gave small relative errors but large absolute errors when predicting atom-in-material force components for some of the atoms having large root-mean-squared forces.

• Scenario # 4: there are both small *R*-squared values and large RMSE values for some of the atoms in these raincloud plots. This is only a problem if a small atom-wise *R*-squared value and a large atom-wise RMSE value occurred for the same atom. In this case, the flexibility model gave large relative errors and large absolute errors when predicting this atom's forces.

Scenarios # 1, 2, and 3 suggest the flexibility model performed acceptably, because either small relative errors or small absolute errors are acceptable. On the other hand, scenario # 4 may indicate the flexibility model performed poorly and needs to be improved.

What constitutes ‘small’ and ‘large’ values is a judgement call. An atom-wise *R*-squared value less than 0.5 could be considered ‘small’. An atom-wise RMSE value could be considered relatively large if it is larger than five times the median value.

**Fig. 29:**
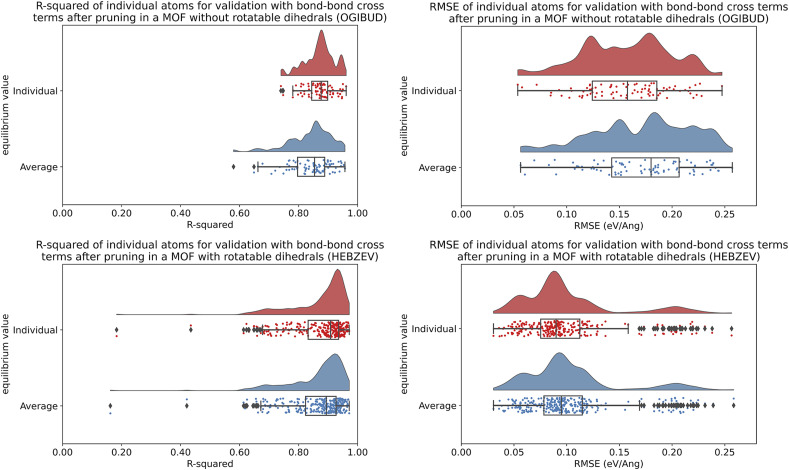
Raincloud plots showing the distribution of atom-wise *R*-squared and atom-wise RMSE (eV Å^−1^) values for atom-in-material forces in the validation datasets for OGIBUD (top panels) and HEBZEV (bottom panels). Results are plotted for individual (red) and average (blue) equilibrium values.

Fig. 29 shows raincloud plots for the atom-wise *R*-squared and atom-wise RMSE values for the validation datasets of OGIBUD and HEBZEV. Close examination of this figure indicates Scenario # 1 when using individual and average equilibrium values for OGIBUD, and Scenario # 2 when using individual and average equilibrium values for HEBZEV. Accordingly, the flexibility models for these two MOFs performed acceptably.

### 8.8 Computational time and memory

Computational time and memory can vary somewhat depending on the computing architecture and setup conditions. Even with this caveat, we believe it is useful to include this type of data here, because it provides some guidance for planning purposes. Potential users of our new method will likely want to know how much computing resources it could potentially require to optimize flexibility parameters for large material databases containing tens of thousands of materials.

**Fig. 30:**
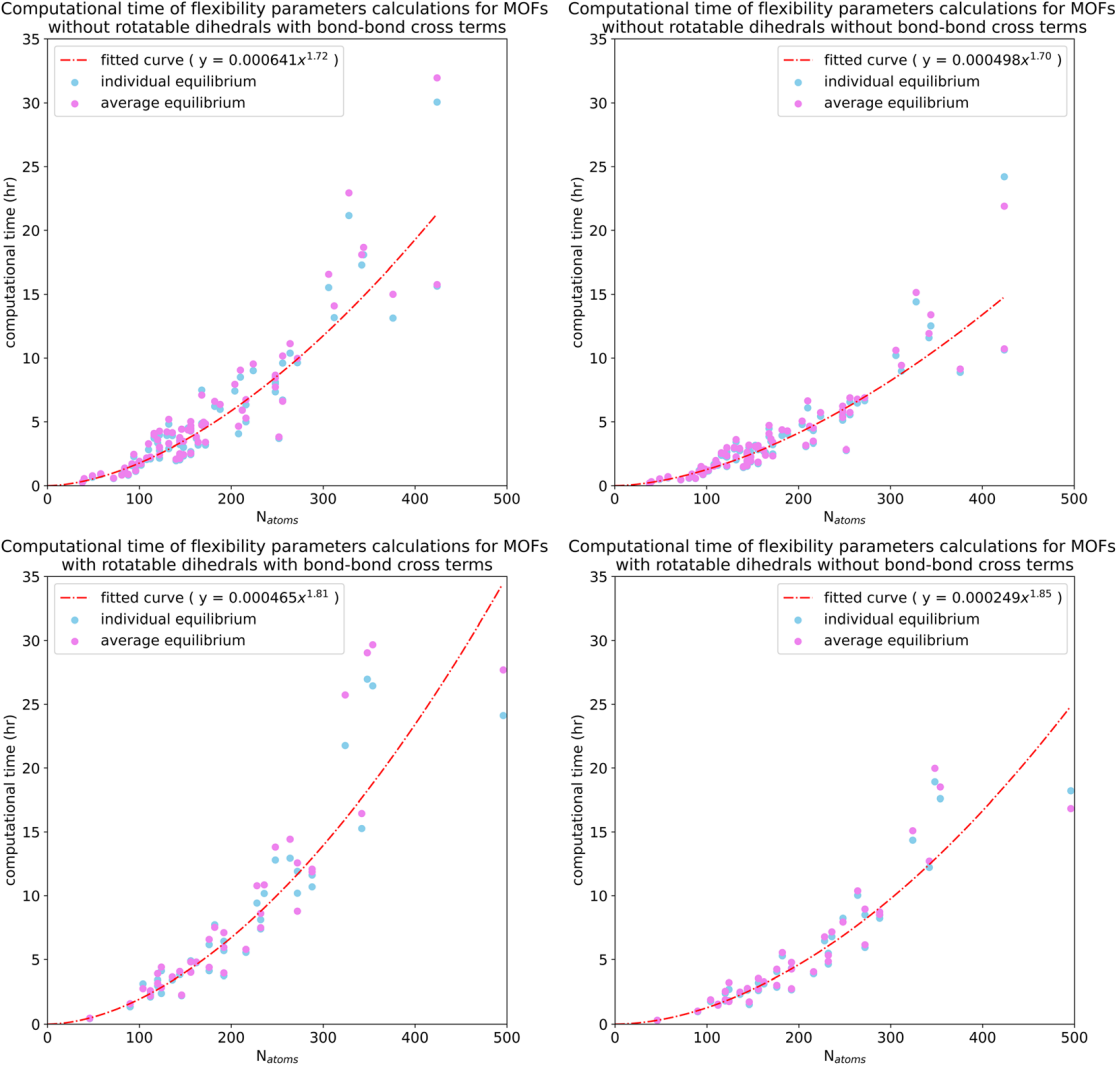
Plots of computational time for flexibility parameters calculation *versus* number of atoms in the MOF's unit cell. These computational times include optimizing force constant values, computing statistics for the training datasets, and computing statistics for the validation datasets. These computational times do not include the times for quantum chemistry calculations to prepare the training and validation datasets. The top panels are for 79 MOFs in quadrants 1 and 2. The bottom panels are for 37 MOFs in quadrants 3 and 4. The left (right) panels are for computations with (without) bond–bond cross terms.

The computational times plotted in Fig. 30 include optimizing force constant values, computing statistics for the training datasets, and computing statistics for the validation datasets. These computational times do not include the times for quantum chemistry calculations to prepare the training and validation datasets. The plotted computational times are for running our SAVESTEPS Python code on a single computing core (*i.e.*, serial computation) on the Expanse cluster at the San Diego Supercomputing Center (SDSC) (The Expanse cluster has AMD EPYC 7742 “Rome” processors.) As shown in Fig. 30, these computational times ranged from 0.17 to 32 hours. The required computational time scaled approximately quadratically (*i.e.*, observed effective exponent between 1.70 and 1.85) as the number of atoms in the MOF's unit cell increased.

Table 5 summarizes overall computational costs for: (i) quantum chemistry calculations (using VASP) to compute the training and validation datasets and (ii) flexibility parameters calculation using our SAVESTEPS Python code. For testing, a MOF with rotatable dihedrals and a MOF without any rotatable dihedrals were chosen in each of five different *N*_atoms_ intervals: [1,99], [100,199], [200,299], [300,399], [400,499].

**Table 5:** Total computational time and memory for: (i) quantum chemistry calculations (using VASP) to compute the training and validation datasets and (ii) flexibility parameters calculation using our SAVESTEPS Python code. Please see the main text for how the peak memory and required memory were defined

MOF	Number of atoms	Quadrant	Quantum chemistry calculations (VASP)		Flexibility parameters calculation (python)	
			Total core hours	Peak memory (GB)	Total core hours	Required memory (GB)
DONNIE	72	1	1650	10	0.6	1
LIWXIZ	132	1	4164	51	5.5	8
OPOBIF	210	1	4091	55	10.5	13
BOMCOX	328	1	31 296	46	21.8	31
ATOBIW	424	1	34 113	53	33.5	42
KACZUM	90	3	1301	39	1.6	2
BEPMEQ	156	3	5880	37	4.4	6
EWUGEK	248	3	7909	62	12.8	18
KATDAM	354	3	18 831	91	28.4	40
SARBUK	496	3	54 408	134	27.1	22

The quantum chemistry computational costs included: (a) optimizing the MOF's geometry (atom-in-material positions) while holding the lattice vectors constant at their experimental values, (b) AIMD calculations for training dataset, (c) finite-displacement ‘Hessian’ geometries for training dataset, (d) single-point energy calculations for a rigid torsion scan for one instance of each rotatable dihedral type (if any were present in the MOF), (e) AIMD calculations for validation dataset. For each MOF, the total core hours for quantum chemistry calculations was computed as follows88
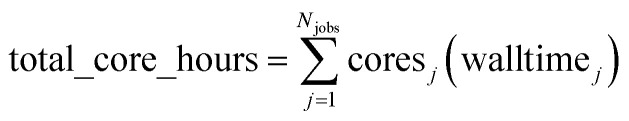
where cores_*j*_ is the number of computing cores for job *j* and walltime_*j*_ is the elapsed wall time from the start of job *j* to its completion. In eqn (88), the sum is over all jobs required to complete items (a) through (e) listed above. The ‘peak memory’ for these quantum chemistry calculations was defined as89
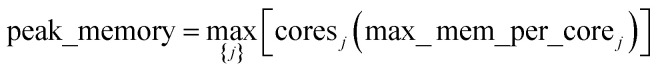
VASP printed the maximum memory used per core (*i.e.*, max_mem_per_core_*j*_) in the output file for each job *j*. In eqn (89), peak memory represents the largest memory that was used for any job to complete items (a) through (e) listed above.

These VASP quantum chemistry calculations were performed on the Expanse cluster at SDSC, the Stampede2 cluster at the Texas Advanced Computing Center (TACC), and/or the Frontera cluster at TACC. We used 48 cores (a partial node) for each VASP job ran on Expanse. The Stampede2 cluster had Intel Xeon Platinum 8160 “Skylake” processors with 48 cores per node. The Frontera cluster has Intel 8280 “Cascade Lake” processors with 56 cores per node. We used one full node for each VASP job ran on Stampede2 and Frontera. For these calculations, we used the following parallelization settings in VASP: LPLANE = TRUE, NCORE = 12 (for Expanse and Stampede2) or NCORE = 14 (for Frontera), LSCALU = FALSE, and NSIM = 4. NCORE specifies the number of cores in a group that work on the same orbital.^118^ For a job running on 48 cores, specifying NCORE = 12 yields 4 groups with 12 cores per group.

Peak memory is not the same as required memory. Required memory is defined as the minimum amount of memory a software program needs to successfully complete a job. Required memory is obviously less than or equal to peak memory. However, the required memory could be significantly smaller than the peak memory, because a software program may use more memory when it is available but not necessarily require this optional memory to successfully complete a job.

In Table 5, the listed time and memory for the flexibility parameters calculation used average equilibrium values and included bond–bond cross terms. Since the flexibility parameters calculation (using our SAVESTEPS Python code) ran on a single computing core, its total core hours was simply the elapsed wall time for that job. For these jobs, we computed the required memory as follows. In the batch script that was submitted to the job scheduler for the Expanse cluster at SDSC, we requested that a specific amount of memory be set aside for running the batch job. We submitted multiple such batch jobs that were identical except they requested different amounts of memory. Jobs that requested too little memory failed due to an out-of-memory error. If a job failed due to an out-of-memory error, we submitted a new job that requested more memory. If a job completed successfully, we submitted a new job that requested less memory. In this way, we found the minimum amount of memory (*i.e.*, the required memory) that had to be requested in order for the job to complete successfully.

As expected, Table 5 shows an average trend of increasing computational time and memory as the number of atoms in the MOF's unit cell increased. However, there are some fluctuations about this average trend in which a specific MOF may require more computational time or memory than a slightly larger MOF. As expected, the quantum chemistry calculations required orders of magnitude more core hours than the flexibility parameters calculation. The required memory for the flexibility parameters calculation was never higher than the peak memory for the quantum chemistry calculations. In other words, the flexibility parameters calculations were less resource intensive than the quantum chemistry calculations.

## 9. How transferable are the force constant values?

**Fig. 31:**
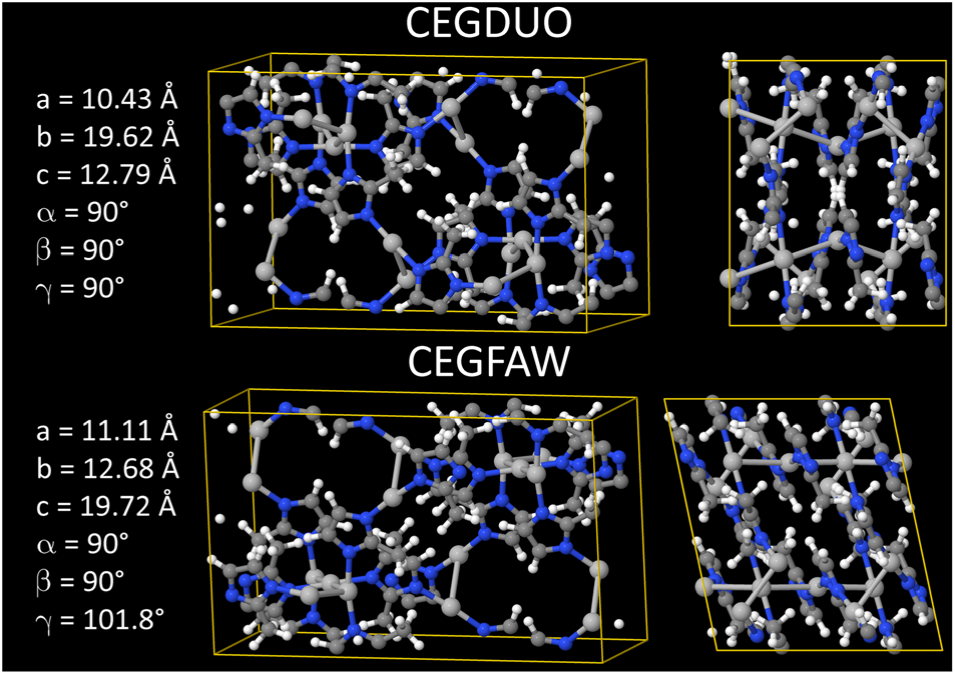
The pair of MOFs CEGDUO and CEGFAW having different crystal structure phases but the same reduced chemical formulas.

**Fig. 32:**
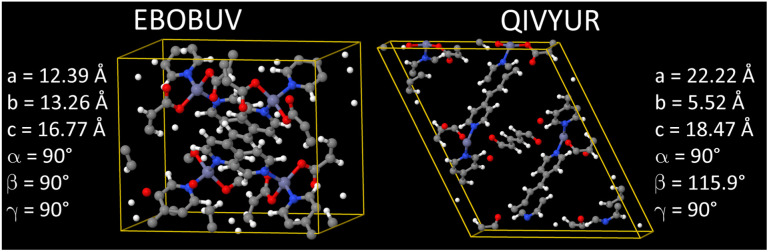
The pair of MOFs EBOBUV and QIVYUR having different crystal structure phases but the same reduced chemical formulas.

To investigate the question of how transferable the optimized force constant values are between different structures, we compared results for a pair of MOFs having different crystal structure phases but the same reduced chemical formulas. Fig. 31 shows the crystal structures of the first pair (CEGDUO and CEGFAW) which are from Quadrant 3 and have the reduced chemical formula AgC_4_H_5_N_2_. Fig. 32 shows the crystal structures of the second pair (EBOBUV and QIVYUR) which are from Quadrant 1 and have the reduced chemical formula ZnC_18_H_14_N_2_O_4_.

Table 6 summarizes the numbers of total and matched types for each of these two pairs. A stretch, bend, or dihedral type was considered ‘matched’ if it was comprised of the same atoms in the same order in both crystal structure phases. The number of ‘matched types (within 3%)’ satisfied the additional criterion that the equilibrium value of the corresponding internal coordinate differed by ≤3% between the MOF pair. For this comparison, the average equilibrium values (*i.e.*, averaged over all instances of the same type within a particular MOF) were compared, and for dihedrals the absolute values of the dihedral instances for each type were averaged and compared (this conforms to exactly the same convention as used for all ‘average equilibrium value’ results presented in this article). This type matching does not have to be one-to-one. For example, symmetry breaking can produce the situation in which one type in the first crystal structure matches two different types in the second crystal structure.

**Table 6:** Number of total and matched types for pairs of MOFs before pruning (BP) and after pruning (AP) of dihedrals. For ‘matched types (within 3%)’, the equilibrium value of the corresponding internal coordinate differed by ≤3% between the MOF pair. For ‘matched types of different value’, the equilibrium value of the corresponding internal coordinate differed by >3% between the MOF pair

		Pair 1 AP		Pair 2 BP		Pair 2 AP	
		CEGDUO	CEGFAW	EBOBUV	QIVYUR	EBOBUV	QIVYUR
Total types	Stretch	11	12	17	17	17	17
	Bend	33	45	30	33	30	33
	Torsion	17	44	51	53	16	14
Matched types (within 3%)	Stretch	8		17		17	
	Bend	18		26		26	
	Torsion	1		13		1	
Matched types of different value	Stretch	0		0		0	
	Bend	0		4		4	
	Torsion	4		17		2	

A stretch, bend, or dihedral type was considered ‘unmatched’ if it appeared in only one MOF of the pair but there was no corresponding type comprised of the same atoms in the same order in the other MOF. This situation could arise if the bond connectivity of atoms differed between the two crystal structures and/or different dihedrals were kept during dihedral pruning. Obviously, there is no notion of ‘transferability’ for types that are ‘unmatched’.

**Fig. 33:**
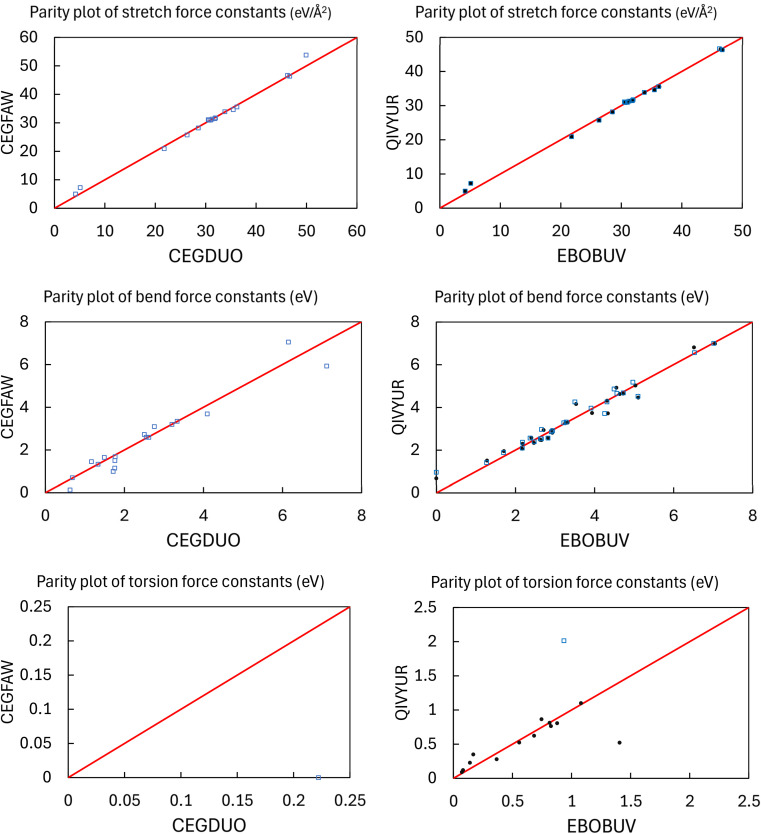
Parity plots of stretch (top panels), bend (middle panels), and torsion (bottom panels) force constants between pairs of MOFs having the same reduced chemical formulas but different crystal structure phases. Data is only shown for the matched types that had average equilibrium values differing by ≤3% between the two MOFs. The left panels show results for CEGDUO/CEGFAW. The right panels show results for EBOBUV/QIVYUR. For both pairs, the after-pruning results are plotted as blue squares. For EBOBUV/QIVYUR, the before-pruning results are plotted as solid black circles.

Fig. 33 shows parity plots of stretch, bend, and torsion force constants for the matched types that had average equilibrium values differing by ≤3% between the two MOFs. From these results, the following conclusions can be drawn. First, the stretch force constant values were highly transferable and almost unchanged by dihedral pruning. Second, the bend force constant values were moderately transferable and almost unchanged by dihedral pruning. Before dihedral pruning, there was good but not great transferability of the torsion force constant values for matched types of the EBOBUV/QIVYUR pair. The torsion force constant values were highly impacted by dihedral pruning. After dihedral pruning, the torsion force constant values had poor transferability.

## 10. Molecular dynamics simulations to compute heat capacity and thermal expansion coefficient

**Fig. 34:**
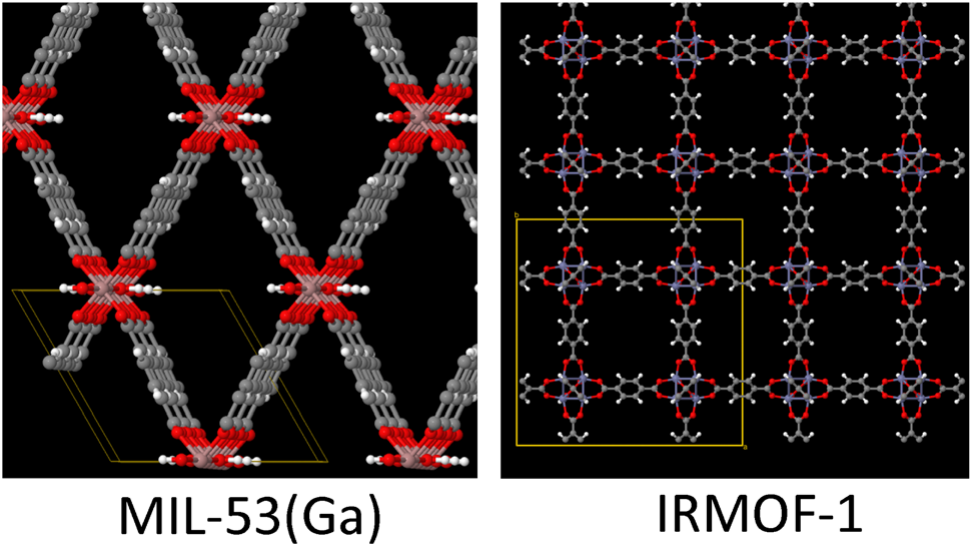
The crystal structures of MIL-53(Ga) (refcode COMDOY) and IRMOF-1 (refcode MIBQAR01).

To calculate the heat capacity (*C*_p_) and volumetric thermal expansion coefficient (*α*) of IRMOF-1 and MIL-53(Ga), we performed molecular dynamics (MD) simulations using the RASPA software package.^119^Fig. 34 illustrates the crystal structures of these two MOFs. The MIL-53(Ga) system (refcode COMDOY) was modeled with a 2 × 3 × 3 supercell with periodic boundary conditions, while the IRMOF-1 system (refcode MIBQAR01) was modeled with a 2 × 2 × 2 supercell with periodic boundary conditions. The MD simulations used a 0.5 femtosecond time step, the Nose–Hoover thermostat with default settings,^120^ and the barostat (with default settings) available in RASPA v2. The simulations were conducted in the NPT ensemble under a range of external temperatures (200, 300, and 400 K) and 1 atm pressure. We performed 100 000 equilibration cycles followed by 500 000 production cycles for MIL-53(Ga). We performed 50 000 equilibration cycles followed by 250 000 production cycles for IRMOF-1. Three different runs were performed at each condition and the results averaged.

We performed these simulations using two different forcefields. Forcefield # 1: we programmed Manz's new angle-bending and dihedral-torsion model potentials into RASPA version 2. We used this modified RASPA version with our flexibility models for the simulations. This forcefield did not include any Lennard-Jones parameters or atomic charges. Table 7 summarizes the types and instances of stretches, angles, and dihedrals in our flexibility models for these two MOFs. Forcefield # 2: additionally, for IRMOF-1 we also used the flexible forcefield developed by Dubbeldam *et al.* (DWES).^11^ The DWES forcefield included Lennard-Jones interactions and atomic charge interactions.

**Table 7:** Summary of types and instances of stretches, angles, and dihedrals in our flexibility models for MIL-53(Ga) and IRMOF-1. The letters after the dot represent a different interaction type involving the same elements. The numbers in parentheses are the number of instances of that particular type

	Stretches	Angles	Dihedrals
MIL-53(Ga) (refcode COMDOY)	8 types: GaO.a(12), GaO.b(5), CH(8), OH(2), CC.a(6), CC.b(10), CC.c(4), CO(9)	16 types: OGaO.a(4), OGaO.b(4), OGaO.c(4), OGaO.d(8), OGaO.e(8), OGaO.f(2), HCC.a(8), HCC.b(8), CCC.a(8), CCC.b(4), CCC.c(8), CCO(8), OCO(4), GaOC(8), GaOGa(2), HOGa(4)	Before pruning: 19 types (186 instances)
			After pruning: 6 types: OGaOC(12), OGaOH(10), CCCC.a(6), CCCC.b(10), CCCO(8), CCCGa(9)
IRMOF-1 (refcode MIBQAR01)	7 types: ZnO.a(32), ZnO.b(96), CO(96), CC.a(48), CC.b(96), CC.c(72), CH(96)	11 types: OZnO.a(96), OZnO.b(96), ZnOZn(48), ZnOC(96), OCO(48), CCO(96), CCC.a(96), CCC.b(48), CCC.c(96), HCC.a(96), HCC.b(96)	Before pruning: 15 types (1632 instances)
			After pruning: 6 types: OZnOZn(96), OZnOC(96), ZnOCC(96), OCCC(96), CCCH(96), CCCC(72)

Table 8 summarizes the computed heat capacities for these materials. For IRMOF-1, both our flexibility model and the DWES forcefield gave *C*_p_ values in excellent agreement with the experimentally-measured value. For MIL-53(Ga), no experimentally-measured *C*_p_ value was available. According to our calculations, the *C*_p_ value for MIL-53(Ga) is predicted to be similar to but slightly higher than the *C*_p_ value for IRMOF-1.

**Table 8:** Comparison of heat capacities at 1 atm and 300 K of different MOFs. BP = before dihedral pruning; AP = after dihedral pruning

MOF/forcefield used	*C*_p_ (J g^−1^ K^−1^)
IRMOF-1 experimental	0.813 (ref. 121)
IRMOF-1/DWES (this work)	0.884
IRMOF-1/our forcefield BP	0.885
IRMOF-1/our forcefield AP	0.847
MIL-53(Ga) COMDOY/our forcefield	0.901

Table 9 compares different values for the volumetric thermal expansion coefficient *α* of IRMOF-1. For this material, the negative value of *α* is caused by ligand flopping which increases with temperature and shortens the ligand end-to-end distance and lattice vector length.^11,122^ When using our flexibility model for this material with the thermostat/barostat (with default settings) in RASPA v2, the volumetric thermal expansion coefficient *α* of IRMOF-1 was substantially over-estimated in magnitude compared to the experimentally-measured value. This is probably due to one of two possible reasons related to excessive floppiness of the ligands. It is possible (although not yet proved) that excessive floppiness of the ligands was caused by the omission of out-of-plane-distance (improper-dihedral) terms in our flexibility model for this material. This issue will need to be studied in more detail in future work. Alternatively, it is possible (although not yet proved) that excessive floppiness of the ligands was caused by excessively large pressure and/or temperature fluctuations introduced by the particular thermostat/barostat employed in these MD simulations. The choice of thermostat/barostat impacts the size of temperature/pressure fluctuations during MD simulations.^123^ At this time, different kinds of thermostats/barostats were not available to us for testing within the simulation code we used; consequently, this issue will need to be studied in more detail in future work.

**Table 9:** Comparison of volumetric thermal expansion coefficient *α* for IRMOF-1 in the range 200–400 K. BP = before dihedral pruning; AP = after dihedral pruning

Method	*α* (10^−6^ K^−1^)
Experimental	−39 to −48 (ref. 122 and 124)
BTW-FF	−16, −9 (ref. 10 and 70)
UFF4MOF^68^	−79 (ref. 70)
DWES (literature)	−57 (ref. 70)
DWES (this work)	−48
QuickFF	−42 to −65 (ref. 30)
UFF^67^	−39 (ref. 70)
DREIDING^125^	−31.8 (ref. 70)
Our forcefield BP	−120
Our forcefield AP	−181

For MIL-53(Ga), we computed a volumetric thermal expansion coefficient *α* of −8.8 × 10^−5^ K^−1^. Several prior studies investigated some mechanical and thermal properties of this MOF, including its breathing motion and a temperature-induced transition between narrow-pore and large-pore phases.^112,113,126–129^

## 11. Conclusions

In this work, we developed a new protocol (see Fig. 3) for fitting the flexibility parameters of a classical forcefield to quantum-mechanically-computed reference data. Our protocol uses the following functional form to describe bonded interactions:

90 *U*_flexibility_ = *U*_bonds_ + *U*_UB_ + *U*_angles_ + *U*_dihedrals_ + (*U*_optional_)

*U*_bonds_ includes bond stretches between bonded neighbors. *U*_UB_ includes Urey–Bradley interactions between a selected subset of second neighbors. In this work, we included Urey–Bradley interactions between diagonal corners of four-membered rings but not between other second neighbors. *U*_angles_ includes angle bends for all bond angles except those contained in 3-membered and 4-membered rings (our protocol discards angles in 3-membered rings, because their degrees of freedom are already covered by the bond stretches. Our protocol discards angles in 4-membered rings, because their degrees of freedom are already covered by the bond stretches and diagonal Urey–Bradley terms.). *U*_dihedrals_ includes the after-pruning dihedrals. If desired, bond–bond cross terms and/or other optional terms (*U*_optional_) can be included in our protocol.

Some key benefits of our SAVESTEPS protocol include the following:

(1) It uses Manz's^50^ ansatz for separating intracluster bonded interactions from intracluster nonbonded interactions. This separation ansatz allows the bonded interactions to be optimized up to and including second-order derivatives in the energy (*i.e.*, harmonic approximation) without requiring any prior parameterization of the intracluster nonbonded interactions.

(2) When using Manz's separation ansatz, the ‘resting value’ of bond length, angle, or dihedral in each flexibility term does not require special fitting, because it equals the corresponding equilibrium value in the quantum-mechanically-computed optimized ground-state geometry.^50^ This allows the forcefield's bonded parameters to be optimized using linear regression instead of requiring nonlinear regression.

(3) The protocol is automated to facilitate its deployment across many materials.

(4) Using an automated procedure, symmetry-equivalent and near-symmetry-equivalent bonds, angles, or dihedrals are classified together into the same type. All instances of the same type share the same force constant value.

(5) The selection of which internal coordinates and which flexibility terms to include in the forcefield is performed in a way that preserves symmetry equivalency while reducing (but not eliminating) redundancy. Dihedral pruning is an important step in this selection process to reduce internal coordinate redundancy.

(6) Our protocol automatically classifies dihedrals as: (a) non-rotatable if they are part of a ring, (b) hindered if they are not part of a ring but have limited range of motion, (c) rotatable if they are not part of a ring and have full range of motion, and (d) linear if they contain at least one linear equilibrium bond angle.

(7) Our protocol uses Manz's^50^ potential energy models for angle bends, rotatable dihedral torsions, and linear-dihedral torsions. The potential energy for each rotatable dihedral type is modeled using a series expansion containing up to seven orthonormal modes, and only those modes making a significant contribution are selected for inclusion in the forcefield. These angle-bending and ADDT potential models provide continuous energy derivatives (*i.e.*, forces) even as the bond angle approaches linearity.

(8) Our protocol optimizes force constant values using a training dataset. This optimization is performed using a regularized linear least squares fitting based on the LASSO method with bounds on some force constants. This resolves the multicollinearity problem and also zeros out unnecessary force constants.

(9) Our protocol ensures that every independent degree of freedom of atom-in-material motion is sampled in the training dataset by including both finite-displacement (aka ‘Hessian’) geometries and AIMD geometries in the force training dataset. This was done while holding the unit cell's size and shape fixed at the experimental values (as pointed out in Section 6.2, it is also possible to apply our protocol to reference geometries that use quantum-mechanically-computed lattice vectors instead of experimentally-measured lattice vectors).

(10) Our protocol ensures each rotatable dihedral type is adequately sampled by performing a series of quantum chemistry calculations across the full range of this dihedral's values. These rotatable dihedral energy scans are included in the training dataset.

(11) Our protocol includes a validation step that verifies the optimized flexibility parameter values accurately reproduce atom-in-material forces across brand new geometries (generated *via* AIMD) that were not used in the training set. Key statistical parameters including *R*-squared and RMSE are computed for the validation dataset. *R*-Squared and RMSE values are also computed and reported for individual atoms in the material to help identify if and where the forcefield needs to be improved.

(12) When the equilibrium values are set individually for each instance of a type, each flexibility term we used is defined such that *U*_term_ = 0 and ∂*U*_term_/∂(IC) = 0 at the optimized ground-state reference geometry, where IC is a corresponding internal coordinate of that flexibility term. For this optimized ground-state geometry, all atom-in-material forces are identically zero for both the quantum-chemistry level of theory used in the training dataset and also for the classical forcefield produced by our optimization protocol. Moreover, *U*_flexibility_ = 0 at this optimized ground-state geometry.

Using this protocol, we constructed and optimized flexibility parameters for 116 MOFs. For each MOF, this method's accuracy was assessed by computing the *R*-squared and RMSE values for a set of 991 geometries in each validation set: 990 new AIMD-generated geometries that were not used in the training set, plus the optimized geometry. Even without cross terms, the flexibility model yielded *R*-squared values of 0.910 (avg across all MOFs) ± 0.018 (st. dev.) for atom-in-material forces in the validation datasets. This is excellent performance. When bond–bond cross terms were included, the flexibility model yielded *R*-squared values of 0.928 (avg across all MOFs) ± 0.015 (st. dev.) for atom-in-material forces in the validation datasets.

Finally, we note some choices in the types of flexibility terms included in our protocol. In this work, we used Urey–Bradley^72^ stretches only for the diagonals of 4-membered rings. It is possible to incorporate additional Urey–Bradley terms in our protocol to augment or replace some of the angle-bending interactions. In this work, we compared flexibility models with and without bond–bond cross-terms. As evident from the statistics listed in the prior paragraph, including bond–bond cross terms produced only a small overall improvement in accuracy. Other types of cross terms (*e.g.*, bond–angle, angle–angle, *etc.*) could be explored.^5,79,130^ Such cross terms could be included in our protocol on an as-needed basis to further improve accuracy. In this work, our protocol used a harmonic bond stretch potential. If desired, anharmonic bond stretching terms could be included to improve accuracy.^6,8,80,131,132^ Our general philosophy is that improper-dihedrals and out-of-plane-distances are not required to construct an accurate flexible forcefield, because these degrees of freedom are already covered by linear combinations of bonds, angles, and proper dihedrals already used in the force field. Though not required, cross terms,^5,79,130^ anharmonic terms,^6,8,80,131,132^ improper-dihedrals, out-of-plane distances, and other refinements could be included in our protocol. Such tweaks to the flexibility terms could further improve accuracy at the expense of slightly increased computational cost and complexity.

We believe this protocol should find widespread applications for developing classical non-reactive flexible forcefields for nanoporous solids, small molecules, and other materials. The automated nature of this protocol facilitates deployment across large numbers of materials. The protocol is concise and computationally efficient without gratuitous oversimplification.

In Section 9, we investigated the question of force constant transferability for similar internal coordinate types appearing in two different chemical structures. For matched types with equilibrium values within 3%, the stretch and bend force constant values exhibited good transferability between different chemical structures. For matched types with equilibrium values within 3%, the torsion force constants exhibited medium transferability before dihedral pruning but poor transferability after dihedral pruning.

In Section 10, we presented molecular dynamics calculations of the heat capacity and volumetric thermal expansion coefficient for IRMOF-1 and MIL-53(Ga). This demonstrates utility of our framework flexibility models for computing some bulk thermodynamic properties of MOFs. We recommend that future work explore the calculation of bulk thermodynamic and mechanical properties in more detail. We recommend that future work compare results using different thermostats, barostats, and ensembles to better understand the effects of computational settings on the computed bulk property values. Specifically, future work should try to resolve the question of whether the overestimation of volumetric thermal expansion coefficient magnitude for IRMOF-1 was due to an inaccuracy of our flexibility model for this material (*e.g.*, neglect of out-of-plane/improper torsion terms in our flexibility model for this material) or due to excessive fluctuations introduced by the particular thermostat/barostat that was used in the molecular dynamics simulations.

We also recommend that future work explore the computation of bulk modulus and elastic constants for MOFs using our flexibility models. This will require a detailed analysis of approximations and convergence analysis for computing bulk modulus and elastic constants.^111–113^ Bulk modulus values are sometimes theoretically estimated by fitting an equation of state to simulated energy *versus* volume curves at absolute zero temperature neglecting the zero-point vibrational energy.^15,111^ However, due to the ligand floppiness that increases with temperature, that approach may not be accurate for estimating the bulk modulus of IRMOF-1 (and other MOFs with floppy ligands) near ambient temperatures. On the other hand, computing the bulk modulus using MD simulations in the NPT ensemble introduces challenges because the magnitude of volume fluctuations is strongly impacted by the choice of barostat.^112,133^ To date, the amount of experimentally-measured and theoretically-computed bulk modulus values for MOFs is limited and close agreement between the two has been reached in only a handful of cases.^134–136^ This issue is beyond the scope of the present work, and we recommend that it be explored in future studies.

## Data availability

Data supporting this article have been included as part of the ESI.† The Python code of our SAVESTEPS program is available at https://bitbucket.org/manzgroup/SAVESTEPS/. We used the May 20, 2024 version of this code for results presented in this article.

## Author contributions

All authors planned calculations, performed calculations, wrote computer codes, analyzed data, interpreted results, and wrote the manuscript. T. A. M. and A. C. E. obtained financial support for the work. T. A. M. supervised the work.

## Conflicts of interest

There are no conflicts of interest to declare.

## Supplementary Material

RA-014-D4RA01859A-s001

RA-014-D4RA01859A-s002

RA-014-D4RA01859A-s003
