# Correction: An automated protocol to construct flexibility parameters for classical forcefields: applications to metal–organic frameworks†

**DOI:** 10.1039/d5ra90084k

**Published:** 2025-07-15

**Authors:** Reza Ghanavati, Alma C. Escobosa, Thomas A. Manz

**Affiliations:** a Chemical & Materials Engineering, New Mexico State University Las Cruces NM 88001 USA tmanz@nmsu.edu

## Abstract

Correction for ‘An automated protocol to construct flexibility parameters for classical forcefields: applications to metal–organic frameworks’ by Reza Ghanavati *et al.*, *RSC Adv.*, 2024, **14**, 22714–22762, https://doi.org/10.1039/D4RA01859A.

## 1. Corrected angle-damped dihedral torsion model potentials

Throughout this correction, the phrase ‘original article’ means the article to which this correction applies: R. Ghanavati, A. C. Escobosa and T. A. Manz, An automated protocol to construct flexibility parameters for classical forcefields: applications to metal–organic frameworks, *RSC Adv.*, 2024, **14**, 22714–22762, DOI: 10.1039/D4RA01859A.

In our original article, eqn (18) for the angle-damped dihedral torsion (ADDT) potential mode three was incorrect, because it violates the combined angle-dihedral coordinate branch equivalency condition described in ref. 50:

a potential[*θ*_ABC_, *θ*_BCD_, *ϕ*_ABCD_] = potential[(2π − *θ*_ABC_),*θ*_BCD_,(*ϕ*_ABCD_ ± π)]

This condition must be satisfied, because both sides of this equation refer to the same physical geometry (*i.e.*, the same positions of atoms in the material).^50^ ADDT mode 3 was represented in our original article asb
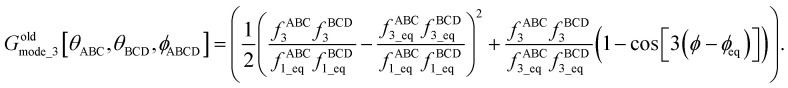
Expanding the square in eqn (b) and regrouping the terms gives

c *G*^old^_mode_3_[*θ*_ABC_,*θ*_BCD_,*ϕ*_ABCD_] = term_1 + term_2 + term_3,

whered
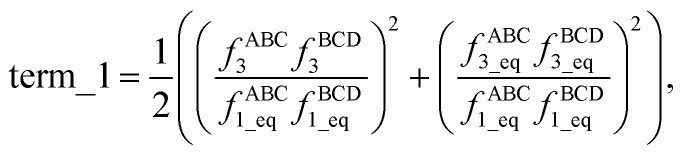
e
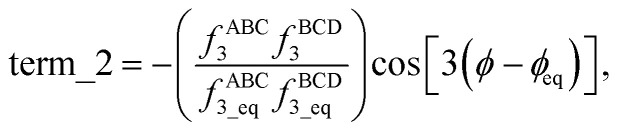
f
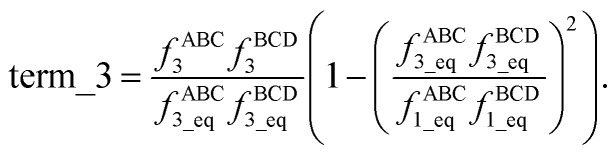


Term_1 and term_2 each satisfy the combined angle-dihedral coordinate branch equivalency condition; however, term_3 does not. Since *f*^ABC^_3_ is an odd function of kangal ABC and has no *ϕ* dependence,

g term_3[*θ*_ABC_,*θ*_BCD_,*ϕ*_ABCD_] = −term_3[(2π − *θ*_ABC_),*θ*_BCD_,(*ϕ*_ABCD_ ± π)],

which violates eqn (a) whenever term_3 is not equal to zero.

In our original article, the model potentials for ADDT modes 1, 2, and 4 already satisfied the combined angle-dihedral coordinate branch equivalency condition in eqn (a); however, a slightly better form was subsequently derived that improves the handling of correlations between *f*^ABC^_*n*_ and *f*^BCD^_*n*_. The updated potentials for ADDT modes 1–4 were derived in ref. 50 and are reproduced below in the corrected eqn (16)–(19):16
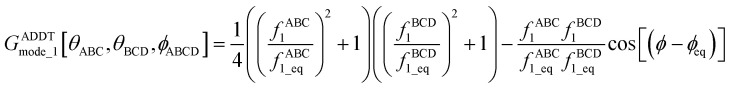
17
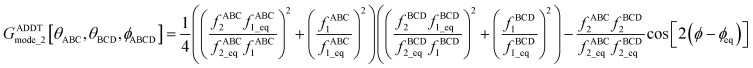
18
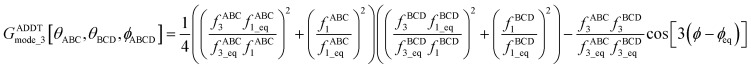
19
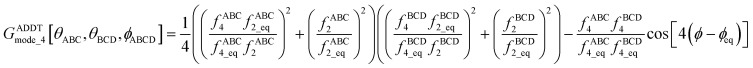
Each of these potentials satisfy the combined angle-dihedral coordinate branch equivalency condition shown in eqn (a). These correct eqn (16)–(19) of our original article.

The potential for ADDT modes 5 through 7 is unchanged. However, we corrected the definition used to define *S*_instance_ to the following:^50^h
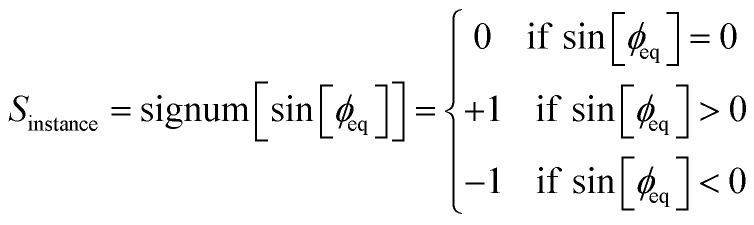
This definition is the same as before, except *S*_instance_ becomes zero for *ϕ*_eq_ = 0 and for *ϕ*_eq_ = π. This requires Fig. 9 to be corrected to include a scaled sign that ranges between −1 and +1.

**Fig. 1:**
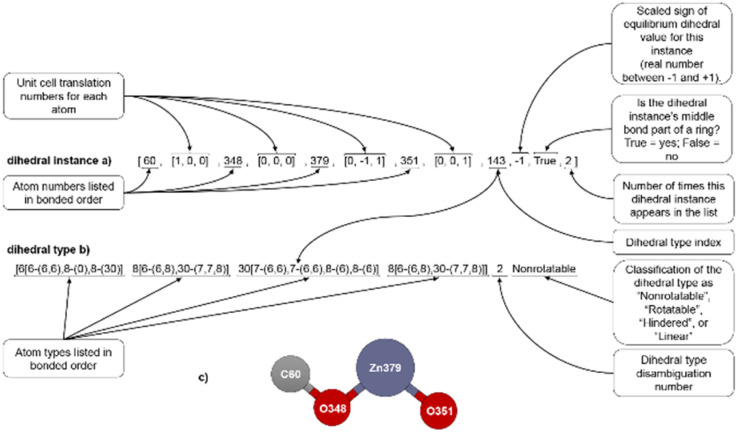
(corrected): Example format for a dihedral instance (a), dihedral type (b), and ball-and-stick illustration (c).

## 2 Flexibility parameters computed using the SAVESTEPS protocol incorporating the corrected dihedral torsion model potentials

For the same 116 MOFs studied in our original article, we repeated the calculations using the five dihedral torsion model potentials and associated selection criteria introduced in ref. 50. These are the ADDT, constant amplitude dihedral torsion (CADT), angle-damped cosine-only (ADCO), constant amplitude cosine-only (CACO), and angle-damped linear dihedral (ADLD) model potentials. This represents minor but important changes compared to the selection protocol used in our original article. Specifically, in cases for which *U*^torsion^_ABCD_[*ϕ*] = *U*^torsion^_ABCD_[−*ϕ*], the updated protocol uses the ADCO (if (*θ*^eq^_ABC_ or *θ*^eq^_BCD_) ≥ 130°) or CACO (if (*θ*^eq^_ABC_ and *θ*^eq^_BCD_) < 130°) model potentials. The ADLD model potential was used for single-linear dihedrals (*i.e.*, when (*θ*^eq^_ABC_ xor *θ*^eq^_BCD_) = 180°); in this case, we included the two ADLD modes^50^ corresponding to the *k*^1^_LD1_ and *k*^1^_LD2_ force constants.

We used the improved smart selection criteria described in ref. 50. For each torsion scan, we first computed the following symmetry descriptori
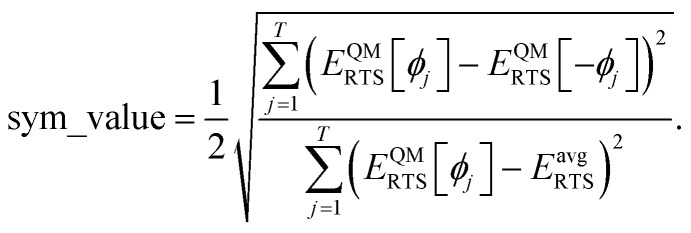


“If sym_value ≤ 0.01, this means *U*^torsion^_ABCD_[*ϕ*] = *U*^torsion^_ABCD_[−*ϕ*] within the tolerance, so the ADCO or CACO model potential was used. In this case, an ADCO or CACO mode was kept if abs[*c*_*m*_] > 0.001. Keeping the ADCO or CACO coefficients greater than this ‘very tight’ cutoff helps *ϕ*^FF^_eq_ more closely approach *ϕ*^training^_eq_. If 0.01 < sym_value ≤ 0.1, the ADDT or CADT model potential was used, and an ADDT or CADT mode was kept if abs[*c*_*m*_] > 0.01. This case corresponds to the situation in which *U*^torsion^_ABCD_[*ϕ*] is approximately, but not strictly equal to, *U*^torsion^_ABCD_[−*ϕ*], so it is beneficial to use a ‘tight’ cutoff (*i.e.*, abs[*c*_*m*_] > 0.01) for retaining torsion modes to achieve a balance between accuracy and conciseness. This ‘tight’ cutoff helps the ADDT or CADT model potential to more accurately reproduce the position of the alternate local energy minimum *ϕ*^alternate^_eq._ ≈ −*ϕ*_eq_. If 0.1 < sym_value, the ADDT or CADT model potential was used, and an ADDT or CADT mode was kept if abs[*c*_*m*_] > 0.1. This case corresponds to the situation in which *U*^torsion^_ABCD_[*ϕ*] is not approximately equal to *U*^torsion^_ABCD_[−*ϕ*], so conciseness of the torsion modes is preferred.”^50^ This ‘normal’ cutoff (*i.e.*, abs[*c*_*m*_] > 0.1) neglects a torsion mode if it affects the SumCSq value by <0.01, where SumCSq is defined as:^50^j
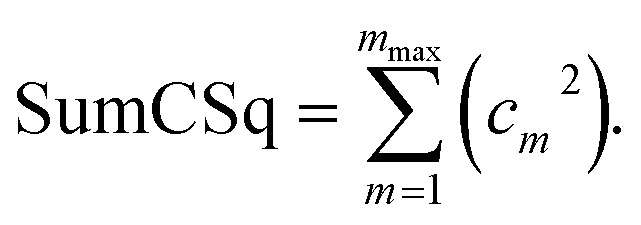
Herein, the ADDT and CADT rotatable types used *m*_max_ = 7, while the ADCO and CACO rotatable types used *m*_max_ = 4.^50^

The corrected Fig. 13 illustrates results of this smart selection procedure for six rotatable dihedral types. When the average of the model potential torsion scan curve differs from the average of the QM-computed torsion scan curve, then *R*-squared is less than SumCSq.^50^ This distinction between *R*-squared and SumCSq for torsion scan curves was missed in our original article, and it is explained in ref. 50. *R*-Squared equals SumCSq when the averages of the model potential and QM-computed torsion scan curves coincide.^50^

**Fig. 2:**
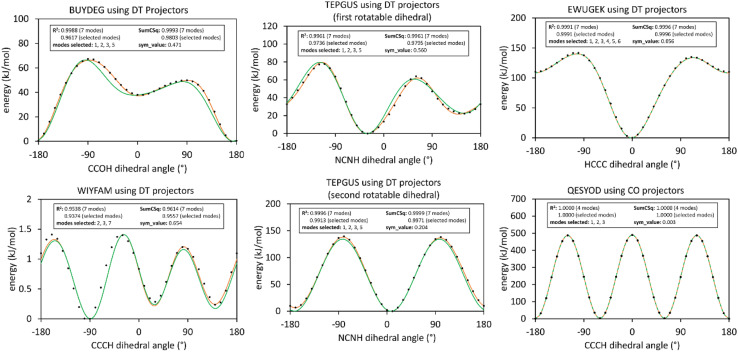
(corrected): Potential energy curves for rigid torsion scans of rotatable dihedrals. In each panel, the black dots show the quantum mechanical energy obtained from single-point DFT_with_dispersion calculations. The orange curve illustrates the fitted model using all modes up to and including *m*_max_, while the green curve shows the fitted model using only the smart-selected modes. Left and middle panels are for rotatable dihedrals having sym_value > 0.1 using DT projectors. The top right panel is for a rotatable dihedral having 0.01 < sym_value ≤ 0.1 using DT projectors. The bottom right panel is for a rotatable dihedral having sym_value ≤0.01 using CO projectors.

Using these smart selection criteria with the corrected dihedral torsion model potentials, the corrected Fig. 19 displays histograms of the smart selected modes for rotatable dihedral types, which includes ADDT rotatable, CADT rotatable, ADCO rotatable, and CACO rotatable dihedral types. The left panel is a histogram of how many torsion modes were smart-selected per rotatable dihedral type. Selecting one torsion mode was the most popular, followed by selecting two torsion modes, and so on in a monotonically decreasing distribution. The right panel is a histogram of the particular modes that were smart-selected. Torsion mode 3 was the most popular followed by torsion mode 2, which was in turn followed by torsion mode 1. Torsion mode 4 was the least popular. Compared to the histograms published in our original article, these histograms have similar overall behavior but occasionally include more modes owing to the slightly tighter smart selection criteria used herein.

**Fig. 3:**
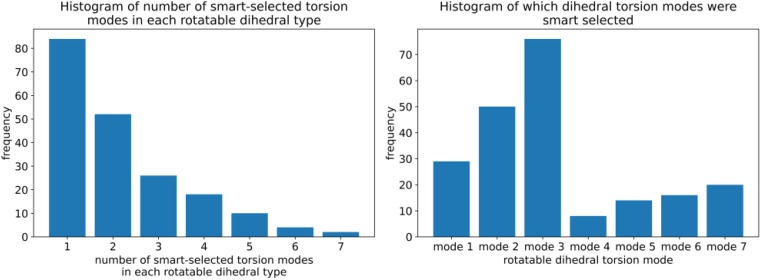
(corrected): (Left panel) Histogram showing how many torsion modes were smart-selected in each rotatable dihedral type. (Right panel) Histogram showing which rotatable dihedral modes were smart selected.

The corrected Table 2 lists the number of dihedral instances, types, and MOFs for each kind of dihedral torsion model potential. When reporting instances in the corrected Table 2, ‘duplicate instances’, which are simply different periodic images of the same underlying dihedral, are counted together as only one dihedral instance; these would be dihedrals that are translated by whole lattice vector(s) relative to each other. Analogous to our original article, the CADT non-rotatable/hindered and ADDT non-rotatable/hindered types were modeled using mode_1 only, except here we used the corrected mode_1 potential from the corrected eqn (16) for the ADDT non-rotatable/hindered dihedral types. The CADT non-rotatable/hindered mode_1 potential is the same as used in our original article. For the CADT rotatable, ADDT rotatable, CACO, and ADCO dihedral types, we used torsion mode smart selection as described above. For the ADLD dihedral types, we used the following potential model:k
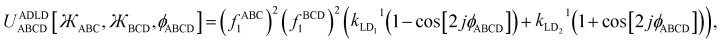
which includes two modes from Manz’s ADLD model potential derived in ref. 50. These are directly proportional to the two modes that were used in our original article.

**Table 2** (corrected): The frequency of occurrence of CADT non-rotatable/hindered, ADDT non-rotatable/hindered, CADT rotatable, ADDT rotatable, ADLD, ADCO, and CACO dihedral torsion model potentials. These results are for all 116 MOFs after dihedral pruning. Results for the corrected dihedral torsion model potentials are shown outside parentheses, while results corresponding to the uncorrected dihedral torsion model potentials are shown inside parentheses

**Table:** 

Dihedral torsion model potential	#Dihedral instances	#Dihedral types	#MOFs
hin/hindered	19 031 (19 031^*a*^)	3287 (3287)	116 (116)
ADDT non-rotatable/hindered	2140 (2140^*a*^)	343 (343)	78 (78)
CADT rotatable	554 (590^*a*^)	90 (95)	37 (37)
ADDT rotatable	10 (10^*a*^)	3 (3)	2 (2)
ADLD	126 (126^*b*^)	12 (12)	5 (5)
CACO	36 (0)	5 (0)	2 (0)
ADCO	0 (0)	0 (0)	0 (0)
^*a*^This is the number with duplicate instances counted together as only one dihedral instance. The slightly different numbers reported in our original article are due to separately counting each duplicate instance, which results in over-counting. ^*b*^This was mistakenly reported as 124 instead of 126 in our original article.			

Table A lists dihedral type classification for MOFs containing one or more rotatable dihedral types with sym_value ≤0.1, which may also contain other rotatable dihedral types with larger sym_values. The last column shows the maximum *R*-squared change when using the updated smart selection criteria with corrected dihedral torsion model potentials compared to the smart selection criteria and dihedral torsion model potentials from our original article. In all cases, the *R*-squared values changed by small amounts. As shown in Table A, the maximum *R*-squared change was 0.0167 which is for EWUGEK.

The classification of dihedrals in Table 2 (corrected) and Table A are after dihedral pruning and before LASSO regression to optimize the values of the force constants. In some cases, LASSO regression optimizes a force constant’s value to zero, which effectively removes that flexibility term from the parameterized flexibility model.

**Table A**: Dihedral type classification for MOFs containing one or more rotatable dihedral types with sym_value ≤0.1, which may also contain other rotatable dihedral types with larger sym_values. Each cell lists the number of dihedral types of that kind in each MOF. Results using our updated smart selection criteria with corrected dihedral torsion model potentials are displayed outside parentheses. Results using the smart selection criteria and uncorrected dihedral model potentials from our original article are shown inside parentheses

**Table:** 

MOF name	hin/hindered	CADT rotatable	ADDT non-rotatable/hindered	ADDT rotatable	CACO	ADCO	Maximum *R*-squared change
ESIFIX	15 (15)	4 (6)	6 (6)	0 (0)	2 (—)	0 (—)	0.0002
EWUGEK	40 (40)	1 (1)	4 (4)	0 (0)	0 (—)	0 (—)	0.0167
HEBZAR	22 (22)	5 (5)	7 (7)	0 (0)	0 (—)	0 (—)	0.0029
IBICED	43 (43)	2 (2)	0 (0)	0 (0)	0 (—)	0 (—)	0.0019
JIVFUQ	6 (6)	2 (2)	2 (2)	0 (0)	0 (—)	0 (—)	0.0034
QESYOD	68 (68)	6 (9)	6 (6)	0 (0)	3 (—)	0 (—)	0.0036

Using the corrected dihedral torsion model potentials and the updated smart selection criteria, we recomputed the flexibility parameters for all 116 MOFs in our dataset (results do not change if a MOF contained no linear dihedrals, no ADDT dihedrals, and no rotatable dihedrals). Using the corrected results, we regenerated all of the figures that are impacted by correcting the torsion model potentials or updating the smart selection criteria: Fig. 13, 19, 22, 23, 25–29. After being replotted using the corrected data, Fig. 23 and 26 did not exhibit any visual changes (compared to the original figures) and therefore do not require a correction. The corrected Fig. 13, 19, 22, 25 and 27–29 are presented here. All changes in these figures were minor and did not impact any of the article’s Conclusions.

**Fig. 4:**
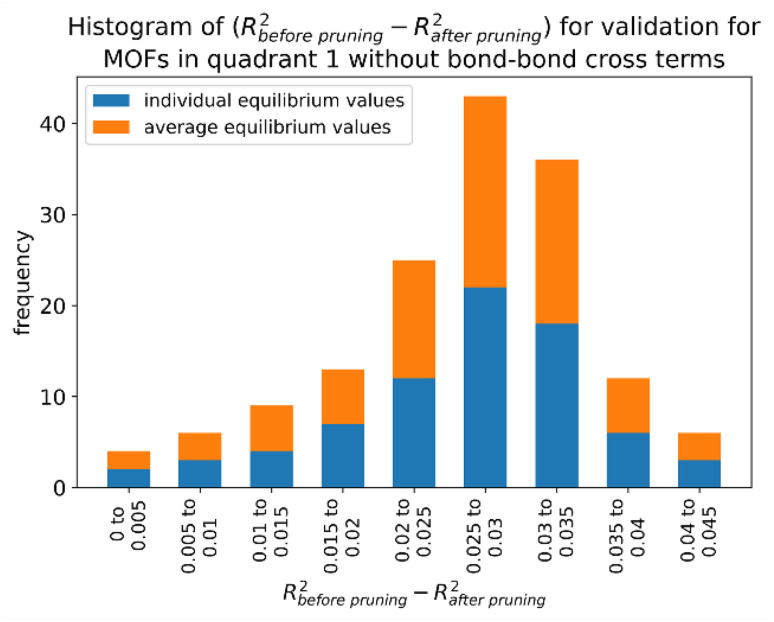
(corrected): Histogram of difference between *R*-squared before dihedral pruning and *R*-squared after dihedral pruning for MOFs in quadrant 1.

**Fig. 5:**
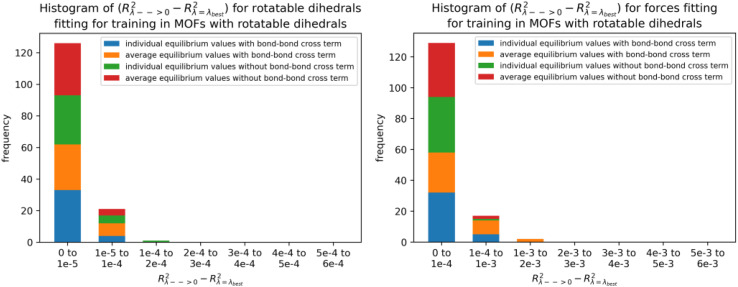
(corrected): Histogram of difference between *R*-squared for *λ* → 0 and *R*-squared for *λ* = *λ*_best_ for rotatable dihedrals training dataset (left panel) and forces training dataset (right panel) in MOFs belonging to quadrants 3 and 4.

**Fig. 6:**
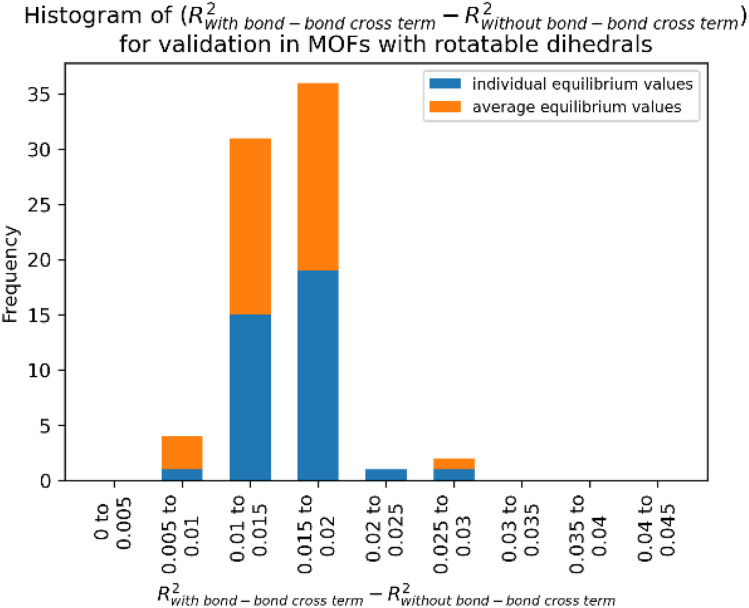
(corrected): Histogram of difference between *R*-squared with bond–bond cross terms and *R*-squared without bond–bond cross terms for the validation dataset in MOFs from quadrants 3 and 4.

**Fig. 7:**
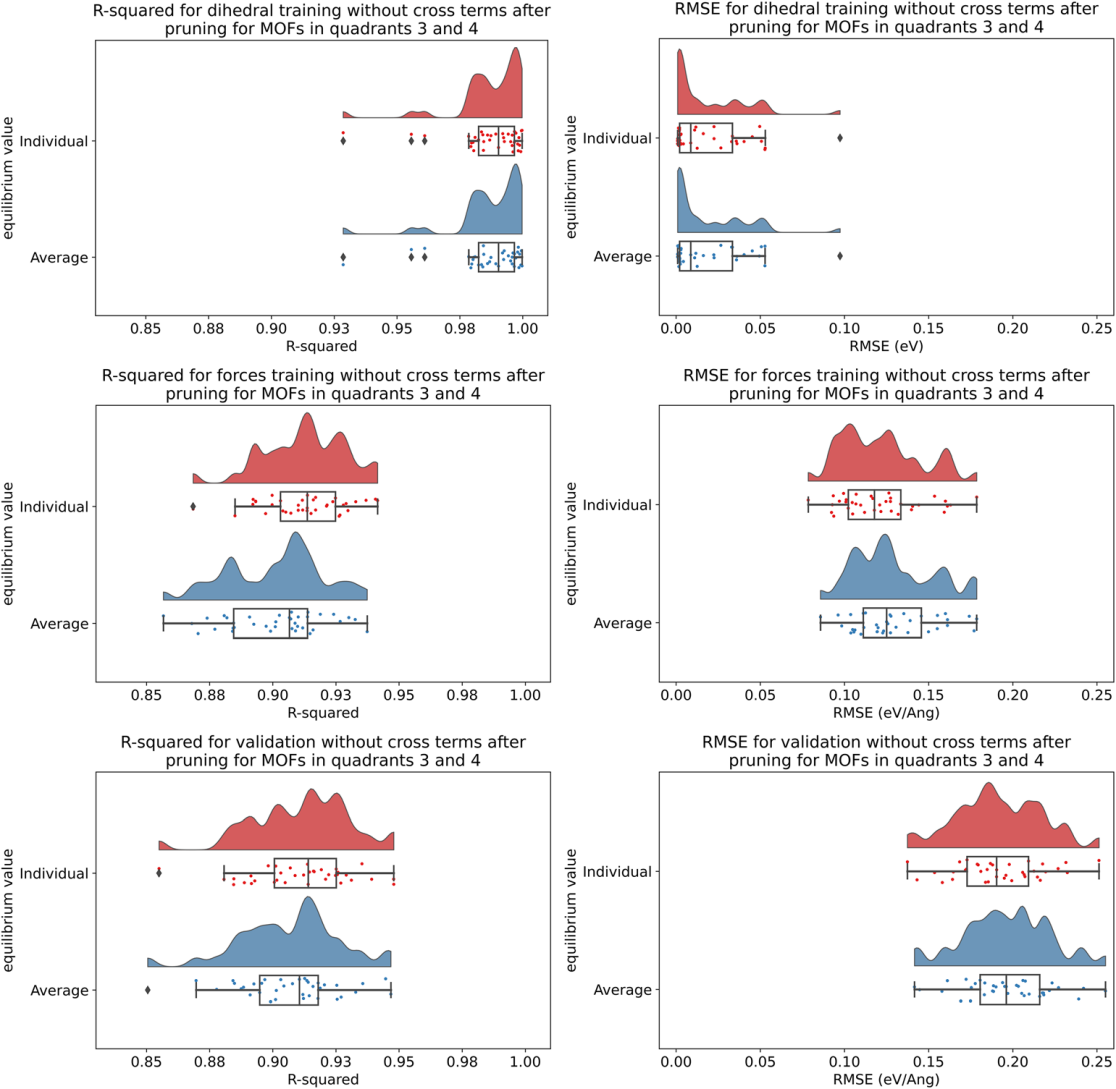
(corrected): Raincloud plots of *R*-squared (left panels) and RMSE (right panels) for rotatable dihedrals training (top panels), forces training (central panels), and validation (bottom panels) for MOFs in quadrants 3 and 4 without cross terms and after pruning. The red distributions represent the values for individual equilibrium values, while the blue distributions represent the values for average equilibrium values.

**Fig. 8:**
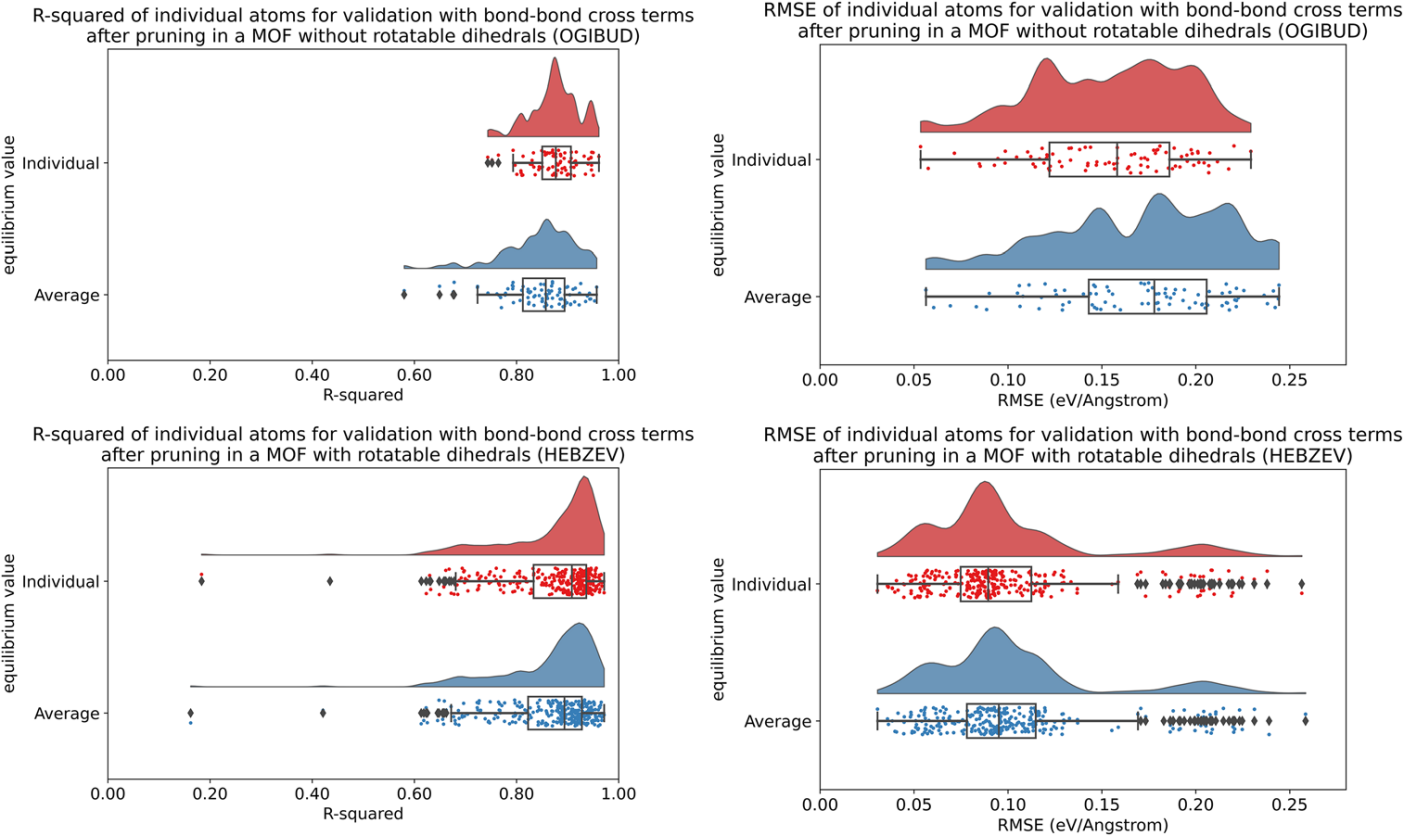
(corrected): Raincloud plots showing the distribution of atom-wise *R*-squared and atom-wise RMSE (eV Å^−1^) values for atom-in-material forces in the validation datasets for OGIBUD (top panels) and HEBZEV (bottom panels). Results are plotted for individual (red) and average (blue) equilibrium values.

In addition to the corrected Table 2 discussed above, corrected Tables 3, 4, 8 and 9 are presented using the data computed using the corrected dihedral torsion model potentials and updated smart selection criteria. All changes in these tables were minor and did not impact any of the article’s Conclusions.

We performed molecular dynamics (MD) simulations using our corrected dihedral torsion model potentials to compute the results shown in the corrected Tables 8 and 9. To perform these simulations, we first programmed and tested our corrected dihedral torsion model potentials into a modified RASPA version 2. RASPA provides a feature (‘Simulation Type Numerical’) that compares analytically-computed to numerically-computed atom-in-material forces. We used this feature to verify that our potential energy and analytic force formulas programmed into RASPA were internally consistent with each other. We also printed out the computed atom-in-material forces of the dihedral torsion potential for selected test cases and compared them to Manz’s reference implementation in Matlab that was published in ref. 50. Agreement was reached between these. These MD simulations used the same settings as described in our original article. For MIL-53(Ga), we computed a volumetric thermal expansion coefficient *α* of −7.7 × 10^−5^ K^−1^ using the forcefield with corrected dihedral torsion model potentials compared to the value of −8.8 × 10^−5^ K^−1^ reported in our original article.

**Table 3** (corrected): Summary of training and validation statistics for MOFs in quadrants 1 and 3. The fourth column indicates whether bond–bond cross (bbc) terms were included. Each numeric entry is the average ± standard deviation

**Table:** 

Quadrant	Equilibrium values type	Dihedral pruned?	bbc?	*R*-Squared training rotatable dihedrals	RMSE (eV) training rotatable dihedrals	*R*-Squared training forces	RMSE (eV Å^−1^) training forces	*R*-Squared validation	RMSE (eV Å^−1^) validation
1	Individual	N	N	—	—	0.933 ± 0.012	0.116 ± 0.024	0.936 ± 0.014	0.165 ± 0.017
1	Average	N	N	—	—	0.922 ± 0.015	0.124 ± 0.025	0.932 ± 0.015	0.171 ± 0.017
1	Individual	Y	N	—	—	0.912 ± 0.014	0.133 ± 0.026	0.910 ± 0.017	0.196 ± 0.018
1	Average	Y	N	—	—	0.902 ± 0.017	0.140 ± 0.027	0.905 ± 0.018	0.201 ± 0.019
1	Individual	Y	Y	—	—	0.929 ± 0.011	0.120 ± 0.024	0.928 ± 0.013	0.175 ± 0.016
1	Average	Y	Y	—	—	0.917 ± 0.016	0.128 ± 0.025	0.922 ± 0.016	0.182 ± 0.018
3	Individual	Y	N	0.988 ± 0.010	0.020 ± 0.023	0.913 ± 0.016	0.121 ± 0.024	0.911 ± 0.020	0.192 ± 0.026
3	Average	Y	N	0.988 ± 0.010	0.020 ± 0.023	0.902 ± 0.020	0.128 ± 0.024	0.907 ± 0.020	0.197 ± 0.026
3	Individual	Y	Y	0.989 ± 0.010	0.020 ± 0.023	0.927 ± 0.015	0.111 ± 0.022	0.927 ± 0.018	0.174 ± 0.025
3	Average	Y	Y	0.988 ± 0.010	0.020 ± 0.023	0.915 ± 0.019	0.119 ± 0.023	0.922 ± 0.019	0.180 ± 0.026

**Table 4** (corrected): Summary of performance statistics for OGIBUD and HEBZEV. The results displayed outside (inside) parentheses represent outcomes from models optimized with (without) bond–bond cross terms

**Table:** 

MOF name	Equilibrium values type	*R*-Squared training rotatable dihedrals	RMSE (eV) training rotatable dihedrals	*R*-Squared training forces	RMSE (eV Å^−1^) training forces	*R*-Squared validation	RMSE (eV Å^−1^) validation
OGIBUD	Individual	—	—	0.8827 (0.8550)	0.1799 (0.1999)	0.8810 (0.8561)	0.2260 (0.2485)
	Average	—	—	0.8363 (0.8196)	0.2124 (0.2230)	0.8543 (0.8372)	0.2500 (0.2643)
HEBZEV	Individual	0.9945 (0.9943)	0.0489 (0.0497)	0.8860 (0.8685)	0.1229 (0.1320)	0.8742 (0.8550)	0.2340 (0.2512)
	Average	0.9944 (0.9943)	0.0492 (0.0495)	0.8715 (0.8567)	0.1304 (0.1377)	0.8678 (0.8505)	0.2398 (0.2551)

**Table 8** (corrected): Comparison of heat capacities at 1 atm and 300 K of different MOFs. BP = before dihedral pruning; AP = after dihedral pruning

**Table:** 

MOF/forcefield used	*C*_p_ (J g^−1^ K^−1^)
IRMOF-1 experimental	0.813 (ref. 121)
IRMOF-1/DWES (this work)	0.884
IRMOF-1/our forcefield BP	0.866
IRMOF-1/our forcefield AP	0.850
MIL-53(Ga) COMDOY/our forcefield	0.888

**Table 9** (corrected): Comparison of volumetric thermal expansion coefficient *α* for IRMOF-1 in the range 200–400 K. BP = before dihedral pruning; AP = after dihedral pruning

**Table:** 

Method	*α* (10^−6^ K^−1^)
Experimental	−39 to −48 (ref. 122 and 124)
BTW-FF	−16, −9 (ref. 10 and 70)
UFF4MOF^68^	−79 (ref. 70)
DWES (literature)	−57 (ref. 70)
DWES (this work)	−48
QuickFF	−42 to −65 (ref. 30)
UFF^67^	−39 (ref. 70)
DREIDING^125^	−31.8 (ref. 70)
Our forcefield BP	−125
Our forcefield AP	−144

Finally, we believe that it is not required to recompute the following data. The computational times displayed in Fig. 24 and 30 and Table 5 and the required memory displayed in Table 5 would not be appreciably impacted by our correction to the dihedral torsion model potentials. Also, the overall trends in the transferability of the force constant values (as shown in Table 6 and Fig. 33) would not be appreciably impacted by our correction to the dihedral torsion model potentials. Table S1 of the ESI† showed modestly better *R*-squared validation and modestly better RMSE validation when *ab initio* molecular dynamics (AIMD) data was included in the training dataset compared to when it was omitted from the training dataset. This trend would not be appreciably impacted by our correction to the dihedral torsion model potentials.

## 3. Is it necessary to include linear dihedrals in the flexibility model?

For the five MOFs containing linear dihedrals, we correctly stated in Section 2.2 of our original article: “For comparison purposes, we also completely reoptimized the flexibility parameterization for these 5 MOFs using an analogous flexibility model except the ADDT linear model potential [*i.e.*, ADLD model potential] was omitted. We found that the validation dataset *R*-squared and RMSE (eV Å^−1^) values for these five MOFs changed little (*e.g.*, in third or fourth significant digits) when the linear dihedrals were omitted from the flexibility model.” However, we neglected to include the underlying data. Please see Table B for the associated data computed using the corrected dihedral torsion model potentials. This does not change any of our article’s Conclusions.

Two of these MOFs (BEPVID and GIWMOP) were from quadrant 3 having at least one rotatable dihedral in their structure. Three of these MOFs (HECQUB, KEWZOD, and XAHROQ) were from quadrant 1 having no rotatable dihedrals in their structure.

Why are the *R*-squared and RMSE values almost unchanged whether linear dihedrals are included or excluded from the flexibility model? The straightforward reason is the dihedral rotation barrier at constant bond angles goes to zero as a bond angle becomes linear. Consequently, the dihedral rotation barrier is almost negligible for linear dihedrals (*i.e.*, dihedrals having at least one linear equilibrium bond angle) for bond angles only slightly displaced away from equilibrium. Thus, the linear dihedrals make comparatively small contributions to the potential energy model in comparison to the other dihedrals in the MOF. For simplicity, it is reasonable to omit the linear dihedrals from the MOF’s flexibility model.

Typically, the linear bond angle would still have an angle-bending model potential that would provide a restoring force whenever the bond angle is displaced away from its equilibrium value (as shown in our original article and ref. 50, the Manz angle-bending model potential is highly recommended due to its accuracy and versatility). Hence, even if the dihedral torsion model potential is omitted for linear dihedrals, the angle-bending model potential would still impose an energy penalty for displacements away from its equilibrium geometry. However, as explained in our original article, there are two cases in which such an angle-bending model potential is omitted in the parameterized flexibility model: (a) The linear bond angle is omitted because it is contained inside a four-membered ring; however, in this case the Urey–Bradley stretches for the diagonals of the four-membered ring help to restore its equilibrium geometry. (b) The angle-bending force constant was optimized to zero during LASSO regression, which indicates it is not needed because some other partly redundant internal coordinates already provide sufficient coverage of the flexibility degrees of freedom.

**Table B**: Comparison of training and validation *R*-squared (unitless) and RMSE (eV Å−1) values for 5 MOFs containing linear dihedrals. The results displayed outside (inside) parentheses represent outcomes from models optimized with (without) considering linear dihedrals in the flexibility model

**Table:** 

MOF name	Equilibrium values type	Dihedral pruned?	bbc?	*R*-Squared training rotatable dihedrals	RMSE (eV) training rotatable dihedrals	*R*-Squared training forces	RMSE (eV Å^−1^) training forces	*R*-Squared validation	RMSE (eV Å^−1^) validation
BEPVID	Individual	Y	N	0.9921 (0.9921)	0.0010 (0.0010)	0.9369 (0.9369)	0.1264 (0.1264)	0.9392 (0.9392)	0.1536 (0.1536)
BEPVID	Average	Y	N	0.9920 (0.9920)	0.0010 (0.0010)	0.9327 (0.9326)	0.1307 (0.1307)	0.9356 (0.9356)	0.1579 (0.1580)
BEPVID	Individual	Y	Y	0.9921 (0.9921)	0.0010 (0.0010)	0.9451 (0.9451)	0.1180 (0.1180)	0.9497 (0.9497)	0.1396 (0.1396)
BEPVID	Average	Y	Y	0.9920 (0.9920)	0.0010 (0.0010)	0.9406 (0.9406)	0.1227 (0.1228)	0.9460 (0.9460)	0.1447 (0.1447)
GIWMOP	Individual	Y	N	0.9785 (0.9786)	0.0507 (0.0507)	0.9152 (0.9154)	0.1274 (0.1273)	0.9209 (0.9210)	0.1867 (0.1867)
GIWMOP	Average	Y	N	0.9786 (0.9786)	0.0507 (0.0507)	0.8740 (0.8740)	0.1553 (0.1553)	0.9044 (0.9044)	0.2053 (0.2053)
GIWMOP	Individual	Y	Y	0.9786 (0.9786)	0.0507 (0.0507)	0.9259 (0.9259)	0.1191 (0.1191)	0.9337 (0.9337)	0.1710 (0.1710)
GIWMOP	Average	Y	Y	0.9785 (0.9785)	0.0507 (0.0507)	0.8842 (0.8842)	0.1488 (0.1489)	0.9136 (0.9136)	0.1952 (0.1952)
HECQUB	Individual	N	N	—	—	0.9193 (0.9192)	0.0973 (0.0974)	0.9242 (0.9241)	0.1905 (0.1906)
HECQUB	Average	N	N	—	—	0.9193 (0.9192)	0.0973 (0.0974)	0.9242 (0.9241)	0.1905 (0.1906)
HECQUB	Individual	Y	N	—	—	0.9011 (0.9011)	0.1077 (0.1078)	0.9002 (0.9002)	0.2186 (0.2187)
HECQUB	Average	Y	N	—	—	0.9011 (0.9011)	0.1077 (0.1078)	0.9002 (0.9002)	0.2186 (0.2187)
HECQUB	Individual	Y	Y	—	—	0.9201 (0.9201)	0.0968 (0.0969)	0.9206 (0.9206)	0.1950 (0.1950)
HECQUB	Average	Y	Y	—	—	0.9201 (0.9201)	0.0968 (0.0969)	0.9206 (0.9206)	0.1950 (0.1950)
KEWZOD	Individual	N	N	—	—	0.9376 (0.9376)	0.1170 (0.1170)	0.9428 (0.9428)	0.1575 (0.1575)
KEWZOD	Average	N	N	—	—	0.9352 (0.9352)	0.1192 (0.1193)	0.9414 (0.9414)	0.1594 (0.1594)
KEWZOD	Individual	Y	N	—	—	0.9223 (0.9223)	0.1305 (0.1305)	0.9208 (0.9208)	0.1852 (0.1853)
KEWZOD	Average	Y	N	—	—	0.9198 (0.9198)	0.1326 (0.1326)	0.9194 (0.9194)	0.1869 (0.1869)
KEWZOD	Individual	Y	Y	—	—	0.9388 (0.9388)	0.1159 (0.1159)	0.9380 (0.9380)	0.1639 (0.1639)
KEWZOD	Average	Y	Y	—	—	0.9358 (0.9358)	0.1187 (0.1187)	0.9362 (0.9362)	0.1663 (0.1663)
XAHROQ	Individual	N	N	—	—	0.9386 (0.9386)	0.1472 (0.1472)	0.9460 (0.9460)	0.1629 (0.1630)
XAHROQ	Average	N	N	—	—	0.9375 (0.9376)	0.1485 (0.1484)	0.9451 (0.9452)	0.1643 (0.1642)
XAHROQ	Individual	Y	N	—	—	0.9333 (0.9334)	0.1535 (0.1534)	0.9385 (0.9386)	0.1738 (0.1738)
XAHROQ	Average	Y	N	—	—	0.9323 (0.9323)	0.1547 (0.1546)	0.9377 (0.9377)	0.1750 (0.1751)
XAHROQ	Individual	Y	Y	—	—	0.9455 (0.9455)	0.1387 (0.1388)	0.9540 (0.9540)	0.1503 (0.1503)
XAHROQ	Average	Y	Y	—	—	0.9444 (0.9444)	0.1402 (0.1402)	0.9531 (0.9531)	0.1519 (0.1519)

## 4. Correction on the origin of energy splitting between symmetric and asymmetric stretches

On pages 22718–22719 of our article, we incorrectly stated that cross terms and/or Urey–Bradley (UB) terms are required to produce an energy splitting between the symmetric stretch vibration and asymmetric stretch vibration in the carbon dioxide molecule. The internal-coordinate Hessian for CO_2_ is diagonal when no cross terms are included in its flexibility model. However, the normal vibrational modes within the harmonic oscillator approximation diagonalize the mass-weighted-Cartesian-coordinate Hessian rather than the internal-coordinate Hessian. As shown in ref. 1 and 2 in the References section of this correction (where ref. 1 denotes ref. 50 of the original article), there is a large energy splitting between the symmetric stretch vibration and asymmetric stretch vibration in the carbon dioxide molecule even when the flexibility model contains no cross terms and no Urey–Bradley terms.

## 5 Corrected ESI†

We updated the ESI† with the results computed using the corrected dihedral torsion model potentials and updated smart selection criteria. Specifically, we corrected the following: (a) Fig. S5–S8† in the PDF file, (b) the spreadsheet containing tables listing detailed information for each MOF, and (c) all of the input and output results in the zip archive. The changes in Fig. S5–S8† were minor and did not impact any of the article’s Conclusions. We also updated the Python code of our SAVESTEPS program available at https://bitbucket.org/manzgroup/SAVESTEPS/.

## Summary

In summary, we used corrected dihedral torsion model potentials and recomputed flexibility parameters for the dataset of 116 MOFs studied in our original publication. This corrected dihedral torsion model potential is required to satisfy the combined angle-dihedral coordinate branch equivalency condition for ADDT mode 3, and it also improves the treatment of correlations between *f*^ABC^_*n*_ and *f*^BCD^_*n*_ for ADDT modes 1 to 4. We also corrected and clarified some other items. All of the Conclusions of our work remain the same. Finally, we note that ref. 50 (the same as ref. S1 in the ESI PDF†) has now been published (as two journal articles) and the updated citation for ref. 50 is listed in the References section below as ref. 1.

The Royal Society of Chemistry apologises for these errors and any consequent inconvenience to authors and readers.

## Supplementary Material

RA-015-D5RA90084K-s001

RA-015-D5RA90084K-s002

RA-015-D5RA90084K-s003
