# Supplementary material for: Correction: An automated protocol to construct flexibility parameters for classical forcefields: applications to metal–organic frameworks
Source: RSC Adv. 2025 Jul 15;15(30):24791–801. doi: 10.1039/d5ra90084k (PMC12261396; doi:10.1039/d5ra90084k)
Supplement: RA-015-D5RA90084K-s002 [file RA-015-D5RA90084K-s002.pdf]

Supplementary Information (SI) for RSC Advances.
This journal is © The Royal Society of Chemistry 2025

Electronic Supplementary Information for

An Automated Protocol to Construct Flexibility Parameters for Classical Forcefields: Applications to Metal-Organic Frameworks

Reza Ghanavati[+], Alma C. Escobosa[+], and Thomas A. Manz*

Chemical & Materials Engineering, New Mexico State University, Las Cruces, NM 88001
*corresponding author email: tmanz@nmsu.edu. [+]These authors contributed equally to this work.

Contents

S1. Torsion model potential for dihedrals with linear equilibrium bond angle

S2. Python function to determine which dihedral middle bonds are contained in rings

S3. Detailed linear equations for flexibility parameters

S4. Python function to compute lambda best

S5. Test of training datasets not including AIMD data

S6. Test of torsion scans for all instances of a rotatable dihedral type

S7. Histograms showing the number of instances per type

S8. Additional LASSO results for MOFs without rotatable dihedrals

S.9 Images of MOF crystal structures

Note: This ESI was corrected in March 2025. The following items were updated to make them consistent with the corrected dihedral torsion model potentials: Figures S5–S8, eqn S19–S21, eqn S24–S25, and ref S1. This does not change any of the Conclusions of our work.

S1. Torsion model potential for dihedrals with linear equilibrium bond angle

A dihedral in which one or both of the equilibrium bond angles is linear (or close to linear) will be referred to here as a 'linear dihedral'. For linear dihedrals, the leading angle-damped dihedral torsion (ADDT_linear, which are more preferably referred to as angle-damped linear dihedral (ADLD)) modes can be derived as follows. First, we start with the allowed angle-damped dihedral torsion potential forms as given in ref [S1]:

$$U_{form\_1}\left[\theta_{ABC},\theta_{BCD},\phi_{ABCD}\right]=\frac{k_{form\_1}}{2}\left(\frac{f_{\theta_{ABC}}\left[\theta_{ABC}\right]f_{\theta_{BCD}}\left[\theta_{BCD}\right]}{f_{\theta_{ABC}}\left[\theta_{ABC}^{eq}\right]f_{\theta_{BCD}}\left[\theta_{BCD}^{eq}\right]}h_{\phi}\left[\phi_{ABCD}\right]-h_{\phi}\left[\phi_{ABCD}^{eq}\right]\right)^{2} \quad (1)$$

$$U_{form\_2}\left[\theta_{ABC},\theta_{BCD},\phi_{ABCD}\right]=k_{form\_2}\frac{f_{\theta_{ABC}}\left[\theta_{ABC}\right]f_{\theta_{BCD}}\left[\theta_{BCD}\right]}{f_{\theta_{ABC}}\left[\theta_{ABC}^{eq}\right]f_{\theta_{BCD}}\left[\theta_{BCD}^{eq}\right]}\left(g_{\phi}\left[\phi_{ABCD}\right]-g_{\phi}\left[\phi_{ABCD}^{eq}\right]\right) \quad (2)$$

(Here, $f_{\theta_{ABC}}\left[\theta_{ABC}\right]$ is a generic placeholder for some angle-damping function(s) that depends on the bond angle's value. Moreover, we could use different specific functions for $f_{\theta_{ABC}}\left[\theta_{ABC}\right]$ in the form_1 and form_2 potentials.) Because the torsion barrier becomes zero when the bond angle is linear, we have

$$\lim_{\theta_{ABC}^{eq}\to\pi} f_{\theta_{ABC}}\left[\theta_{ABC}^{eq}\right]=0 \quad (S3)$$

To avoid division by zero for linear dihedrals, we must modify the two forms above as

$$\tilde{k}_{form\_1}=\frac{k_{form\_1}}{\left(f_{\theta_{ABC}}\left[\theta_{ABC}^{eq}\right]f_{\theta_{BCD}}\left[\theta_{BCD}^{eq}\right]\right)^{2}} \quad (S4)$$

$$U_{form\_1}^{linear\_dihedral}\left[\theta_{ABC},\theta_{BCD},\phi_{ABCD}\right]=\frac{\tilde{k}_{form\_1}}{2}\left(f_{\theta_{ABC}}\left[\theta_{ABC}\right]f_{\theta_{BCD}}\left[\theta_{BCD}\right]h_{\phi}\left[\phi_{ABCD}\right]\right)^{2} \quad (S5)$$

$$\tilde{k}_{form\_2}=\frac{k_{form\_2}}{f_{\theta_{ABC}}\left[\theta_{ABC}^{eq}\right]f_{\theta_{BCD}}\left[\theta_{BCD}^{eq}\right]} \quad (S6)$$

$$U_{form\_2}^{linear\_dihedral}\left[\theta_{ABC},\theta_{BCD},\phi_{ABCD}\right]=\tilde{k}_{form\_2}f_{\theta_{ABC}}\left[\theta_{ABC}\right]f_{\theta_{BCD}}\left[\theta_{BCD}\right]g_{\phi}\left[\phi_{ABCD}\right] \quad (S7)$$

Since ϕ_{ABCD}^{eq} is undefined for linear dihedrals, form_2 for linear dihedrals omits the $g_{\phi}\left[\phi_{ABCD}^{eq}\right]$ term.

In a procedure analogous to that used for the nonlinear dihedrals in ref [S1], for the linear dihedrals we first insert $h_{\phi}\left[\phi_{ABCD}\right]=\sin\left[m\phi_{ABCD}\right]$ and $f_{\theta_{ABC}}\left[\theta_{ABC}\right]=f_{m}^{ABC}\left[Ж_{ABC}\right]$ into eqn (S5) to get

$$U_{sin\_form\_1}^{linear\_dihedral}\left[Ж_{ABC},Ж_{BCD},\phi_{ABCD}\right]=\frac{\tilde{k}_{form\_1}^{m,sin}}{2}\left(f_{m}^{ABC}\left[Ж_{ABC}\right]\right)^{2}\left(f_{m}^{BCD}\left[Ж_{BCD}\right]\right)^{2}\sin^{2}\left[m\phi_{ABCD}\right] \quad (S8)$$

where the kangal is

$$Ж_{ABC}=\cos\left[\theta_{ABC}/2\right] \quad (S9)$$

This procedure is repeated for $h_{\phi}\left[\phi_{ABCD}\right]=\cos\left[m\phi_{ABCD}\right]$ to give:

$$U_{cos\_form\_1}^{linear\_dihedral}\left[Ж_{ABC},Ж_{BCD},\phi_{ABCD}\right]=\frac{\tilde{k}_{form\_1}^{m,cos}}{2}\left(f_{m}^{ABC}\left[Ж_{ABC}\right]\right)^{2}\left(f_{m}^{BCD}\left[Ж_{BCD}\right]\right)^{2}\cos^{2}\left[m\phi_{ABCD}\right] \quad (S10)$$

Clearly,

$$U_{sin\_form\_1}^{linear\_dihedral}\left[Ж_{ABC}^{eq},Ж_{BCD}^{eq},\phi_{ABCD}\right]=U_{cos\_form\_1}^{linear\_dihedral}\left[Ж_{ABC}^{eq},Ж_{BCD}^{eq},\phi_{ABCD}\right]\approx 0 \quad (S11)$$

Since the potential energy increases whenever the system's geometry is moved away from its minimum energy (i.e., the optimized equilibrium ground-state geometry), this requires the constraints

$$\tilde{k}_{form\_1}^{m,sin}\geq 0 \quad (S12)$$

$$\tilde{k}^{m,\cos}_{form\_1} \geq 0 \quad (S13)$$

Substituting

$$\sin^2[m\phi_{ABCD}] = \frac{1-\cos[2m\phi_{ABCD}]}{2} \quad (S14)$$

$$\cos^2[m\phi_{ABCD}] = \frac{1+\cos[2m\phi_{ABCD}]}{2} \quad (S15)$$

into eqn (S8) and (S10) gives

$$U^{linear\_dihedral}_{sin\_form\_1}[Ж_{ABC}, Ж_{BCD}, \phi_{ABCD}] = \frac{\tilde{k}^{m,\sin}_{form\_1}}{2}\left(f^{ABC}_{m}[Ж_{ABC}]\right)^2\left(f^{BCD}_{m}[Ж_{BCD}]\right)^2\left(\frac{1-\cos[2m\phi_{ABCD}]}{2}\right) \quad (S16)$$

$$U^{linear\_dihedral}_{cos\_form\_1}[Ж_{ABC}, Ж_{BCD}, \phi_{ABCD}] = \frac{\tilde{k}^{m,\cos}_{form\_1}}{2}\left(f^{ABC}_{m}[Ж_{ABC}]\right)^2\left(f^{BCD}_{m}[Ж_{BCD}]\right)^2\left(\frac{1+\cos[2m\phi_{ABCD}]}{2}\right) \quad (S17)$$

Since $\sin^2[m\phi_{ABCD}] + \cos^2[m\phi_{ABCD}] = 1$, $U^{linear\_dihedral}_{sin\_form\_1}$ and $U^{linear\_dihedral}_{cos\_form\_1}$ are not fully linearly independent. Consequently, to span the entire possible space of dihedral potential energy functions, it is necessary to also include dihedral potentials of form_2. This is obvious, because $U^{linear\_dihedral}_{sin\_form\_1}[Ж_{ABC}, Ж_{BCD}, \phi_{ABCD}]$ and $U^{linear\_dihedral}_{cos\_form\_1}[Ж_{ABC}, Ж_{BCD}, \phi_{ABCD}]$ are both even functions of ϕ_{ABCD}. The odd functions of ϕ_{ABCD} can be included by choosing

$$U^{linear\_dihedral}_{form\_2}[\theta_{ABC}, \theta_{BCD}, \phi_{ABCD}] = \tilde{k}_{form\_2}\left(f^{ABC}_{m}[Ж_{ABC}]\right)^2\left(f^{BCD}_{m}[Ж_{BCD}]\right)^2 S_{instance}\sin[2m\phi_{ABCD}] \quad (S18)$$

$$S_{instance} \in \{-1, 0, +1\} \quad (S19)$$

The form of eqn (S18) was specifically chosen so that the angle-damping prefactor $\left(f^{ABC}_{m}[Ж_{ABC}]\right)^2\left(f^{BCD}_{m}[Ж_{BCD}]\right)^2$ has the same form as in eqn (S16)–(S17) above, and also so that the $\sin[2m\phi_{ABCD}]$ function in eqn (S18) complements the $\cos[2m\phi_{ABCD}]$ function appearing in eqn (S16)–(S17) above. In eqn (S18), the lowest allowed power of $Ж_{ABC}$ and $Ж_{BCD}$ must be ≥ 2 (i.e., strictly greater than one) to ensure the torsion force goes to zero when the bond angle reaches its equilibrium linear value. To ensure combined angle-dihedral coordinate branch equivalency (see ref [S1]), the sin function must contain an even multiple of ϕ_{ABCD} to match the even power of $Ж_{ABC}$ in $\left(f^{ABC}_{m}[Ж_{ABC}]\right)^2$. These observations show that eqn (S18) has the proper form.

$U^{linear\_dihedral}_{form\_2}[\theta_{ABC}, \theta_{BCD}, \phi_{ABCD}]$ involves the sign $S_{instance}$, because $U^{linear\_dihedral}_{form\_2}[\theta_{ABC}, \theta_{BCD}, \phi_{ABCD}]$ is an odd function of ϕ_{ABCD}. In general, different dihedral instances of the same dihedral type can have different values of $S_{instance}$. Because ϕ_{eq} is undefined for linear dihedrals, the value of $S_{instance}$ cannot be assigned using the sign of ϕ_{eq} for linear dihedrals. For linear dihedrals, the value of $S_{instance}$ must be determined by detecting local mirror-image environments (i.e., local chiral enantiomer environments) for different dihedral instances of the same dihedral type. Owing to this complication, we did not include $U^{linear\_dihedral}_{form\_2}[\theta_{ABC}, \theta_{BCD}, \phi_{ABCD}]$ in the flexibility models parameterized in this article.

In general, the ADDT_linear model potential may contain contributions from m = 1, 2, 3, etc. In practice, this will be truncated at some order. For calculations presented in this article, our ADDT_linear model included the following two modes:

$$G^{ADDT\_linear}_{mode\_8}[\theta_{ABC}, \theta_{BCD}, \phi_{ABCD}] = \left(f^{ABC}_{1}\right)^2\left(f^{BCD}_{1}\right)^2\left(1-\cos[2\phi_{ABCD}]\right) \quad (S20)$$

$$G^{ADDT\_linear}_{mode\_9}\left[\theta_{ABC},\theta_{BCD},\phi_{ABCD}\right]=\left(f_1^{ABC}\right)^2\left(f_1^{BCD}\right)^2\left(1+\cos\left[2\phi_{ABCD}\right]\right) \quad (S21)$$

$$U^{ADDT\_linear}_{mode\_8}\left[\theta_{ABC},\theta_{BCD},\phi_{ABCD}\right]=k_\phi^8 G^{ADDT\_linear}_{mode\_8}\left[\theta_{ABC},\theta_{BCD},\phi_{ABCD}\right] \quad (S22)$$

$$U^{ADDT\_linear}_{mode\_9}\left[\theta_{ABC},\theta_{BCD},\phi_{ABCD}\right]=k_\phi^9 G^{ADDT\_linear}_{mode\_9}\left[\theta_{ABC},\theta_{BCD},\phi_{ABCD}\right] \quad (S23)$$

The ADDT_linear potential modes were numbered as modes ‘8’ and ‘9’, to keep them separate from the seven modes used in the ADDT_rotatable model potential. Comparing eqn (S20)–(S23) to eqn (S16)–(S17) shows that

$$k_\phi^8 = \tilde{k}^{1,sin}_{form\_1}/4 \quad (S24)$$

$$k_\phi^9 = \tilde{k}^{1,cos}_{form\_1}/4 \quad (S25)$$

In this work, we treated MOFs according to two cases. <u>*Case # 1*</u>: When the after-pruning dihedral types for a particular MOF contained no linear dihedral types, then we did not include any ADDT_linear model potential in the parameterized flexibility model for this MOF. In this case, when we generated a before-pruning flexibility model for comparison purposes (to quantify the effects of dihedral pruning on the R-squared and RMSE values) then we also did not include any linear dihedral types (or ADDT_linear model potential) for this MOF in the before-pruning parameterization. <u>*Case # 2*</u>: When the after-pruning dihedral types for a particular MOF contained one or more linear dihedral types, then we included the ADDT_linear model potential in the parameterized flexibility model for this MOF. In this case, when we generated a before-pruning flexibility model for comparison purposes (to quantify the effects of dihedral pruning on the R-squared and RMSE values) then we also included all linear dihedral types (and the ADDT_linear model potential) for this MOF in the before-pruning parameterization.

S2. Python function to determine which dihedral middle bonds are contained in rings

Figure S1 shows a rigorously correct and complete Python function we wrote that determines which middle bonds are parts of rings (i.e., bond path cycles) and which are not. The algorithm we used is briefly described as follows. The function takes three inputs:

(1) The list of bonds that comprise middle bonds in (active) dihedral instances; this list is called ‘middle_bonds_dihedrals’.
(2) The list of first neighbor atom images (aka ‘first_neighbor_atoms’) for every atom in the reference unit cell. For example, suppose that atom 43 in the reference (i.e., (0,0,0)) unit cell is directly bonded to the (1,0,0) image of atom 10; then this would be stored as an entry [10,(1,0,0)] in the list of first neighbors to atom 43.
(3) The number of atoms (N_{atoms}) in the reference unit cell.

Here the term ‘atom image’ refers to an entry such as [10,(1,0,0)] that contains the atom index (in this case = 10) and the unit cell translation indices (in this case = (1,0,0)). The middle bond of a dihedral instance is read and the two corresponding atom images forming this bond are ‘atom_image_A’ and ‘atom_image_B’. Next, we initialized four working lists:

(a) ‘visited_A’ and ‘visited_B’ lists that are each initialized to [atom_image_A, atom_image_B],

(b) A ‘not_searched_A’ list that is initialized to [atom_B], and a ‘not_searched_B’ list that is initialized to [atom_A].

The algorithm contains an outer, middle, and inner layer of nested loops. The outer loop is a FOR loop that runs over the middle bonds in the dihedral instances. The middle loop is a WHILE loop that determines whether a particular middle bond is contained within a ring or not. The two inner loops run over the first neighbor atoms of current_atom_A and current_atom_B, which are the current atoms along the bond paths being searched and the first elements of not_searched_A and not_searched_B, respectively.

Current_atom_image_A is set to the first entry in not_searched_A. The inner loop for current_atom_A works as follows. Using the first_neighbor_atoms list, the algorithm identifies all of the first neighbors of current_atom_image_A. For example, if current_atom_image_A = [43,(0,3,1)] then one of its first neighbors is identified as [10,(1,0,0) + (0,3,1)] = [10,(1,3,1)], where the image indices in current_atom_image_A and first_neighbor_atoms are added together to get 'atom image' of the first neighbor (aka 'adjusted_neighbor_atom_image') of current_atom_image_A. If neither current_atom_image_A nor adjusted_neighbor_atom_image equals atom_image_B, then if adjusted_neighbor_atom_image is in visited_B then a ring has been located and the inner loop iterations break. Otherwise, if adjusted_neighbor_atom_image is not in visited_A, then it is appended to visited_A and also appended to not_searched_A if the edge depth is $\leq 2N_{atoms.}$ After looping over all of the first neighbor atom images of current_atom_A, the first entry in not_searched_A is removed because it has now been searched.

Current_atom_image_B is set to the first entry in not_searched_B. The algorithm then proceeds to the inner loop for current_atom_B. This works analogously to the inner loop iterations for current_atom_A described in the previous paragraph, except the roles of 'A' and 'B' are now swapped.

Here, the 'edge_depth' refers to the number of bonds in the shortest bond path connecting current_atom_image_A to atom_image_A along the search path, or in the shortest bond path connecting current_atom_image_B to atom_image_B along the search path. The algorithm searches paths emanating from each end of the middle bond to an edge_depth of 2Natoms. By adding the edge depths from each end, a total edge depth of $2N_{atoms} + 2N_{atoms} = 4N_{atoms}$ is searched. This guarantees the algorithm searches for all bond path cycles containing up to $4N_{atoms}$, which guarantees that a bond path cycle (aka ring) will be discovered if one passes through the middle bond being investigated. The algorithm finds a ring if any bond path emanating from the A end of the middle bond joins up with any bond path emanating from the B end of the middle bond. The algorithm determines a middle bond is not part of any ring if it exhausts all search paths on either side (i.e., either not_searched_A or not_searched_B becomes empty) or it reaches a searched edge depth of $2N_{atoms}$ on both sides.

```
def finding_rings(middle_bonds_dihedrals, first_neighbor_atoms, Natoms)
    rings_found = []

    for i in range(len(middle_bonds_dihedrals)):
        visited_A = []
        visited_B = []
        rings_found.append(False)
        not_searched_A = []
        not_searched_B = []
        atom_image_A = middle_bonds_dihedrals[i][0]
        atom_image_B = middle_bonds_dihedrals[i][1]
        visited_A.append(atom_image_A)
        visited_A.append(atom_image_B)
        not_searched_A.append([atom_image_B,1])
        visited_B.append(atom_image_B)
        visited_B.append(atom_image_A)
        not_searched_B.append([atom_image_A,1])

        while len(not_searched_A) > 0 and len(not_searched_B) > 0:
            current_atom_image_A = not_searched_A[0][0]
            edge_depth_A = not_searched_A[0][1]
            current_atom_A = current_atom_image_A[0]
            current_image_A = current_atom_image_A[1]
            current_atom_image_B = not_searched_B[0][0]
            edge_depth_B = not_searched_B[0][1]
            current_atom_B = current_atom_image_B[0]
            current_image_B = current_atom_image_B[1]

            for j in range(len(first_neighbor_atoms[current_atom_A-1])):
                neighboring_atom_image = first_neighbor_atoms[current_atom_A-1][j]
                neighboring_atom = neighboring_atom_image[0]
                neighboring_image = neighboring_atom_image[1]
                adjusted_neighboring_image = [neighboring_image[0] + current_image_A[0], neighboring_image[1] + current_image_A[1], neighboring_image[2] + current_image_A[2]]
                adjusted_neighbor_atom_image = [neighboring_atom,adjusted_neighboring_image]
                if current_atom_image_A != atom_image_B:
                    if (adjusted_neighbor_atom_image in visited_B) and (adjusted_neighbor_atom_image != atom_image_B):
                        rings_found[i] = True
                        not_searched_A = []
                        not_searched_B = []
                        break
                if adjusted_neighbor_atom_image not in visited_A:
                    visited_A.append(adjusted_neighbor_atom_image)
                    if edge_depth_A <= 2*Natoms:
                        not_searched_A.append([adjusted_neighbor_atom_image,edge_depth_A+1])

            if len(not_searched_A) > 0:
                not_searched_A.pop(0)
            else:
                break

            for j in range(len(first_neighbor_atoms[current_atom_B-1])):
                neighboring_atom_image = first_neighbor_atoms[current_atom_B-1][j]
                neighboring_atom = neighboring_atom_image[0]
                neighboring_image = neighboring_atom_image[1]
                adjusted_neighboring_image = [neighboring_image[0] + current_image_B[0], neighboring_image[1] + current_image_B[1], neighboring_image[2] + current_image_B[2]]
                adjusted_neighbor_atom_image = [neighboring_atom,adjusted_neighboring_image]
                if current_atom_image_B != atom_image_A:
                    if (adjusted_neighbor_atom_image in visited_A) and (adjusted_neighbor_atom_image != atom_image_A):
                        rings_found[i] = True
                        not_searched_B = []
                        not_searched_A = []
                        break
                if adjusted_neighbor_atom_image not in visited_B:
                    visited_B.append(adjusted_neighbor_atom_image)
                    if edge_depth_B <= 2*Natoms:
                        not_searched_B.append([adjusted_neighbor_atom_image,edge_depth_B+1])

            if len(not_searched_B) > 0:
                not_searched_B.pop(0)

    return rings_found
```

Figure S1: Python function to determine which dihedral middle bonds are contained in rings.

S3. Detailed linear equations for flexibility parameters

First, we need to read the files containing lists of internal coordinate types and instances, which were generated using the procedure described in Section 5 of the main text. Specifically, files containing lists of the following are read: atom types; bond types; bond instances; angle types; angle instances; dihedral types; dihedral instances; classification of each dihedral type as non-rotatable, hindered, rotatable, or linear; training and validation geometries and their quantum-mechanically-computed atom-in-material forces; the (experimental) lattice vectors; rigid torsion scan geometries (and quantum-mechanically-computed single-point energies) for one instance of each rotatable dihedral type. Then, the internal coordinates (i.e., bond lengths, UB lengths, angles, and dihedrals) are parsed into nested arrays containing all the interactions grouped by type. The angles list contains a flag indicating whether the angle is part of a 3-membered or 4-membered ring or not. The bond-bond cross terms use the full list of angles, while the angle-bending potential skips any angles that are part of a 3-membered or 4-membered ring.

Our linear regression problem contains two kinds of observation variables in the combined training dataset: (a) quantum-mechanically-computed atom-in-material forces and (b) quantum-mechanically-computed total energies. The quantum-mechanically-computed atom-in-material forces are from the QM-optimized geometry, AIMD geometries, and finite-displacement 'Hessian' geometries. There are a total of

$$\mathrm{f\_rows} = 3N_{\mathrm{atoms}}N_{\mathrm{force\_geoms}} \tag{S26}$$

force components in the forces training dataset. The quantum-mechanically-computed total energies are from the rigid torsion scan (RTS) geometries and comprise the rotatable dihedrals training dataset.

Since the forces are zero at the equilibrium geometry, the no-intercept linear regression model is used. Therefore, to be able to use a no-intercept model, we centered the observation variable (i.e., the QM-computed energy) for rotatable dihedral torsions by subtracting the average value:

$$E_{\mathrm{avg}} = \frac{1}{TN_{\mathrm{rdt}}}\sum_{i=1}^{N_{\mathrm{rdt}}}\sum_{j=1}^{T} E_{i,j}^{\mathrm{SPG}} \tag{S27}$$

T=36 is the number of equally spaced dihedral values along each rigid torsion scan. N_{rdt} is the number of rotatable dihedral types. Here, $E_{i,j}^{\mathrm{SPG}}$ is the quantum-mechanically-computed total energy of the single-point geometry (SPG) corresponding to the jth displaced geometry of the ith rotatable dihedral type.

This gives the following observation variables for the combined training dataset $\vec{Y}^{\mathrm{QM}}$ comprised of the forces training dataset $\vec{Y}^{\mathrm{QM\_forces}}$ and the rotatable dihedrals training dataset $\vec{Y}^{\mathrm{QM\_energies}}$:

$$\vec{Y}^{\mathrm{QM}} = \begin{bmatrix} \vec{Y}^{\mathrm{QM\_forces}} \\ \vec{Y}^{\mathrm{QM\_energies}} \end{bmatrix} \tag{S28}$$

$$\vec{Y}^{QM\_forces} = \begin{bmatrix} F^{QM}_{atom1,x} \\ F^{QM}_{atom1,y} \\ F^{QM}_{atom1,z} \\ F^{QM}_{atom2,x} \\ \vdots \\ F^{QM}_{N_{atoms},z} \end{bmatrix} \tag{S29}$$

$$\vec{Y}^{QM\_energies} = \begin{bmatrix} \left(E^{SPG}_{1,1} - E_{avg}\right) \\ \left(E^{SPG}_{1,2} - E_{avg}\right) \\ \left(E^{SPG}_{1,3} - E_{avg}\right) \\ \vdots \\ \left(E^{SPG}_{N_{rdt},T} - E_{avg}\right) \end{bmatrix} \tag{S30}$$

N_{atoms} is the number of atoms in the material's unit cell. As a result, $\vec{Y}^{QM\_forces}$ is a column vector with $\text{length}\left[\vec{Y}^{QM\_forces}\right] = 3(N_{atoms})\left(N_{forces\_geoms}\right)$, and $\vec{Y}^{QM\_energies}$ is a column vector with $\text{length}\left[\vec{Y}^{QM\_energies}\right] = TN_{rdt}$.

The predictor variables also contain two sets of data: one for forces fitting and the other for the rotatable dihedrals fitting. Let M be a matrix containing values of the predictor variables. This leads to the following linear model:

$$Y_i^{pred} = \sum_{j=1}^{p}\left(M_{ij}\beta_j\right) \tag{S31}$$

where i is the observation index and j is the model parameter index. In our case, each model parameter is a force constant for a flexibility term:

$$\beta_j = k_j \tag{S32}$$

The total number of attempted force constants in the model is p. Here, the term 'total number of attempted force constants' refers to the number of flexibility terms (i.e., number of force constants) that were 'attempted' before any of these were zeroed out by the bounds or regularization constraints.

Because the atom-in-material forces for the AIMD-generated geometries depend on all of the force constants while the RTS energies depend only on the rotatable dihedral force constants, it follows that the predictor variables matrix **M** has the form

$$\mathbf{M} = \begin{bmatrix} \mathbf{M1}\left(1:\text{f\_rows},1:\left(N_{AFCs} - N_{rd\_AFCs}\right)\right) & \mathbf{M1}\left(1:\text{f\_rows},\left(1+N_{AFCs} - N_{rd\_AFCs}\right):N_{AFCs}\right) \\ 0 & \mathbf{M2} \end{bmatrix} \tag{S33}$$

It directly follows that

$$\text{size}[\mathbf{M1}] = \left(\text{f\_rows}, N_{AFCs}\right) \tag{S34}$$

$$\text{size}[\mathbf{M2}] = \left(TN_{rdt}, N_{rd\_AFCs}\right) \tag{S35}$$

N_{AFCs} is the total number of attempted force constants. N_{rd_AFCs} is the number of rotatable dihedral attempted force constants.

To define the $\mathbf{M1}$, we need to start from the following relation:

$$\vec{F}_A^{bonded} = -\vec{\nabla}_A U_{cluster}^{bonded,new}\left[\left\{\vec{R}_B\right\}\right] = -\sum_{j=1}^{p} k_j \vec{\nabla}_A G_j\left[\left\{\vec{R}_B\right\},\left\{\alpha_h^{eq}\right\}\right] \quad \text{(S36)}$$

Comparing eqn (S31) and (S32) to (S36) reveals that

$$M1_{(3N_{atoms}(\mu-1)+3(\gamma-1)+\xi),j} = -\vec{\nabla}_\gamma G_j\left[\left\{\vec{R}_B^\mu\right\},\left\{\alpha_h^{eq}\right\}\right] \quad \text{(S37)}$$

where μ is the geometry number. ξ = 1, 2, 3 for x, y, z force components of atom γ, respectively. $\left\{\alpha_h^{eq}\right\}$ are the equilibrium values of the internal coordinates. The derivatives in eqn (S37) can be computed either numerically (using finite difference approximation) or analytically. In this article, we used finite difference approximation with 0.0001 Å displacement of an atom along x, y, or z to compute these derivatives:

$$\frac{\partial G_j}{\partial R_\gamma^\xi} = \frac{G_j\left[\left\{\vec{R}_{B\neq\gamma}^\mu, \vec{R}_\gamma^\mu + (\Delta d)\hat{x}_\gamma^\xi\right\},\left\{\alpha_h^{eq}\right\}\right] - G_j\left[\left\{\vec{R}_{B\neq\gamma}^\mu, \vec{R}_\gamma^\mu\right\},\left\{\alpha_h^{eq}\right\}\right]}{\Delta d} \quad \text{(S38)}$$

where j is the interaction index; μ is the geometry index; γ is the atom index; ξ = 1, 2, 3 for x, y, z components, respectively; Δd is the displacement distance; $\hat{x}_\gamma^\xi$ is a unit vector.

For the predictor variables in rotatable dihedrals fitting, as discussed in Sections 2.2 and 7.1, we can have up to seven active modes for each rotatable dihedral. We first determine which dihedral modes are active for each rotatable dihedral type using the method described in Section 7.1. Then let $G_{\mu,i,\vartheta,m_\varsigma}^{SPG}$ be the k-free flexibility term for the ϑth dihedral instance's ς^{th} smart-selected torsion mode $m_\varsigma \in \{1,2,...7\}$ of the ith rotatable dihedral type of the μth geometry in the rotatable dihedrals training dataset. We sum these over all instances belonging to the same rotatable dihedral type to get

$$G_{\mu,i,m_\varsigma}^{SPG} = \sum_\vartheta G_{\mu,i,\vartheta,m_\varsigma}^{SPG} \quad \text{(S39)}$$

For a rigid torsion scan along an instance of a particular rotatable dihedral type, we still have to compute $G_{\mu,i,\vartheta,m_\varsigma}^{SPG}$ for every instance of every rotatable dihedral type, because the dihedral displacements of different instances and/or different types are not necessarily independent of each other. Next, we average over all of the rigid torsion scan geometries:

$$G_{i,m_\varsigma}^{avg} = \frac{1}{TN_{rdt}}\sum_{\mu=1}^{TN_{rdt}} G_{\mu,i,m_\varsigma}^{SPG} \quad \text{(S40)}$$

This allows us to construct a zero-intercept model by defining the matrix $\mathbf{M2}$ as follows:

$$\mathbf{M2} = \begin{bmatrix} \left(G_{1,1,m_1}^{SPG} - G_{1,m_1}^{avg}\right) & \left(G_{1,1,m_2}^{SPG} - G_{1,m_2}^{avg}\right) & \cdots & \left(G_{1,N_{rdt},m_{last}}^{SPG} - G_{N_{rdt},m_{last}}^{avg}\right) \\ \left(G_{2,1,m_1}^{SPG} - G_{1,m_1}^{avg}\right) & & & \\ \vdots & \ddots & \ddots & \vdots \\ \left(G_{TN_{rdt},1,m_1}^{SPG} - G_{1,m_1}^{avg}\right) & \cdots & \cdots & \left(G_{TN_{rdt},N_{rdt},m_{last}}^{SPG} - G_{N_{rdt},m_{last}}^{avg}\right) \end{bmatrix} \quad \text{(S41)}$$

(*Note*: In the software code, the columns for the dihedral potential modes (and associated dihedral torsion force constants) could be presented in a different order than that shown in eqn (S41). However, regardless of the particular ordering, the set of equations solved would be functionally equivalent.)

S4. Python function to compute lambda best

```
def finding_lambda_best(lambda_sequence, R2_calculated_sequence, number_of_parameters_sequence, Natoms):
        temp = 0
        lambda_a = lambda_sequence[-1]
        R_sq_a = R2_calculated_sequence[-1]
        n_params_a = number_of_parameters_sequence[-1]
        lambda_temp = lambda_a

        for i in range((len(lambda_sequence)-2),-1,-1):
            lambda_b = lambda_sequence[i]
            R_sq_b = R2_calculated_sequence[i]
            n_params_b = number_of_parameters_sequence[i]
            if n_params_b < n_params_a:
                temp = ((3*Natoms)/(1-R_sq_b))*((R_sq_a - R_sq_b)/(n_params_a - n_params_b))
                if temp > 0.5:
                    lambda_temp = lambda_a
                    n_parameters_temp = n_params_a
                    break
                else:
                    lambda_a = lambda_b
                    R_sq_a = R_sq_b
                    n_params_a = n_params_b

        lambda_best = lambda_temp
        n_parameters_best  = n_parameters_temp

        return [lambda_best, n_parameters_best]
```

Figure S2: Python function to compute lambda_best.

S5. Test of training datasets not including AIMD data

For one MOF without rotatable dihedrals and one MOF with rotatable dihedrals, we tested the impact of omitting AIMD data from the training dataset. In this case, the training dataset was comprised of only finite-displaced ‘Hessian’ geometries, the optimized geometry, and single-point displaced dihedral geometries (if any rotatable dihedrals were present in the MOF). The validation datasets were comprised of the optimized geometry plus AIMD data from runs independent of AIMD data (if any) used in the training dataset. For each MOF, the same validation dataset was used irrespective of the training dataset used. As shown in Table S1, including AIMD data in the training dataset resulted in a better flexibility model, because it significantly increased the R-squared validation and significantly lowered the RMSE validation.

Table S1: Effects of excluding AIMD data from the training dataset. Values are listed with using individual [average] equilibrium values of internal coordinates. These calculations were performed after dihedral pruning with no bond-bond cross terms.

	R-squared validation	RMSE (eV/Å) validation
PEYPIU with AIMD in training	0.9317 [0.9231]	0.1779 [0.1887]
PEYPIU without AIMD in training	0.8951 [0.8536]	0.2204 [0.2603]
PEGBEK01 with AIMD in training	0.9464 [0.9394]	0.1486 [0.1581]
PEGBEK01 without AIMD in training	0.9323 [0.9181]	0.1670 [0.1838]

S6. Test of torsion scans for all instances of a rotatable dihedral type

Among the MOFs from quadrants 3 and 4 (i.e., having at least one rotatable dihedral type), we used a random number generator to randomly select one MOF. Then we used a random number generator to randomly select one of this MOF's rotatable dihedral types that had more than one instance. As it happened, this process randomly selected a rotatable dihedral type with six instances in the MOF BEPMEQ. Then we quantum-mechanically-computed torsion scan curves for every instance of this rotatable dihedral type. As shown in Figure S3, all of these torsion scan curves were identical. More generally, if two instances of the same type have different signs for ϕ_{eq} , then one would have torsion scan curves for these two instances that are mirror images of each other; since this case is automatically handled by Manz's torsion model potentials, it does not require generating separate torsion scan curves for these two instances. This validates the method of generating a torsion scan curve for one instance of each rotatable dihedral type.

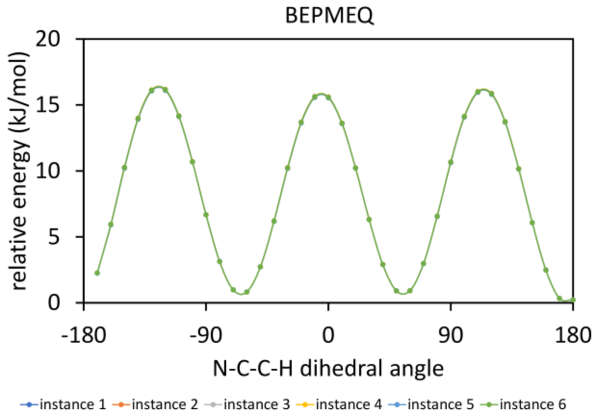

Figure S3: Torsion scan curves for six instances of the same rotatable dihedral type in the MOF BEPMEQ. All of the curves are directly on top of each other.

S7. Histograms showing the number of instances per type

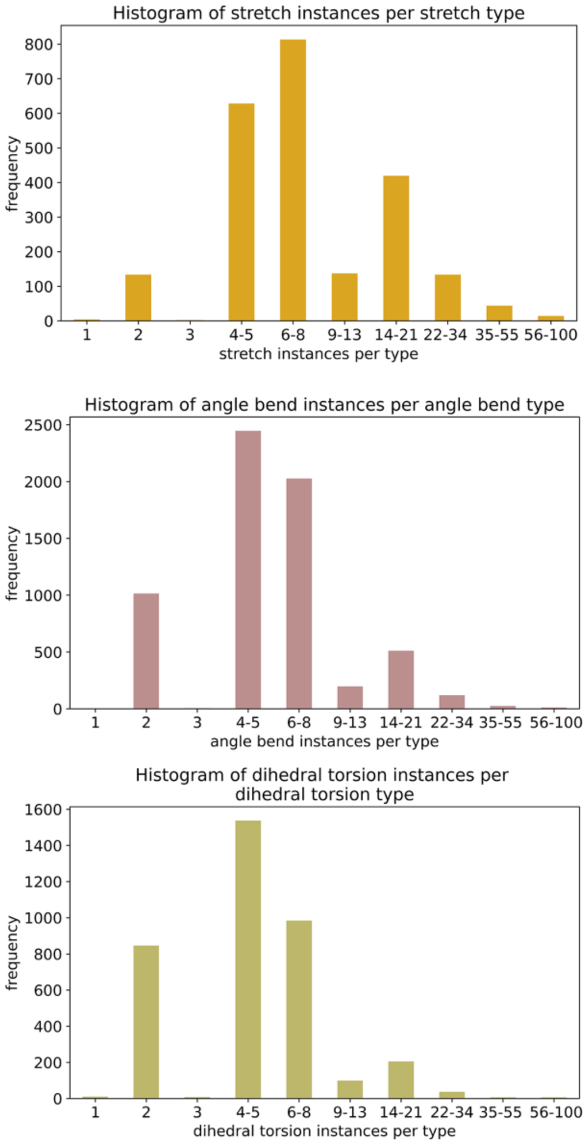

Figure S4: Histograms showing the number of stretch instances per stretch type (top panel), the number of angle bend instances per angle bend type (middle panel), and the number of dihedral torsion instances per dihedral torsion type (bottom panel). The data includes all MOFs in quadrants 1, 2, 3, and 4.

S8. Additional LASSO results for MOFs without rotatable dihedrals

All results in this section are for calculations following dihedral pruning. In Figure S5, we present a histogram illustrating the difference between the R-squared value for $\lambda \to 0$ compared to $\lambda = \lambda_{best}$, on forces fitting within the training dataset of MOFs in quadrants 1 and 2. This R-squared difference peaked within the range 0 to 0.0001. Hence, the effect on R-squared was almost negligible. Notably, the subsequent range, spanning from 0.0001 to 0.001, gave the second-highest bar in the plot.

Figure S6 contains raincloud plots showing the distribution of R-squared and RMSE (eV/Å) values for forces in the training and validation datasets for MOFs in quadrants 1 and 2 using λ_{best}. As shown in these raincloud plots, R-squared > 0.81 for each MOF in these quadrants. Such high R-squared values indicate strong agreement between the model-predicted and quantum-mechanically-computed forces. Even when using new geometries (i.e., validation dataset) that were not part of the model's training, the model accurately reproduced the ab-initio forces.

Figure S7 shows a stacked histogram of the R-squared difference when bond-bond cross terms are included in the potential model versus when they are not. The plotted data is for MOFs in quadrants 1 and 2 after dihedral pruning and using λ_{best}. When using either average equilibrium values or individual equilibrium values, the distribution peaked at a R-squared difference of 0.015–0.02. This shows that including bond-bond cross terms slightly improves the flexibility model's accuracy; however, this improvement in accuracy comes with some increased computational costs due to the added bond-bond cross terms.

Figure S8 shows the number of force constants eliminated by the LASSO method for $\lambda \to 0$ and the additional number that were eliminated when increasing λ to λ_{best}. The results plotted in this figure included bond-bond cross terms. Results without bond-bond cross terms were already plotted in Section 8.6.1 of the main text. Some of the k's eliminated for $\lambda \to 0$ may have been eliminated by the non-negative bounds placed on stretches, bends, single-mode torsions, and linear-dihedral torsions, while others may have been eliminated due to linear dependencies between the flexibility terms. The k's eliminated when increasing λ to λ_{best} also correspond to unimportant flexibility terms that contribute negligibly to the model's accuracy. Examining Figure S8, the percentage of k's eliminated was relatively small but nonzero.

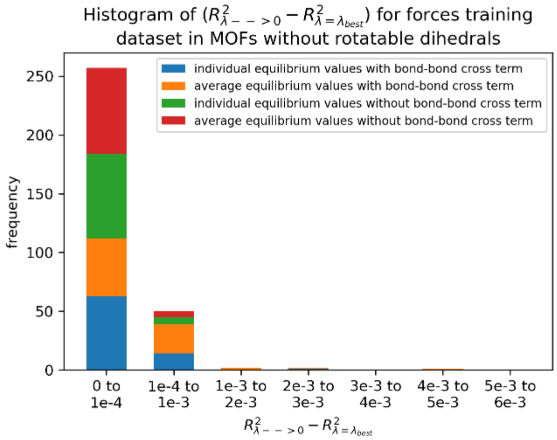

Figure S5: Histogram of difference between R-squared for λ → 0 and R-squared for λ = λ_{best} for forces training dataset in MOFs belonging to quadrants 1 and 2.

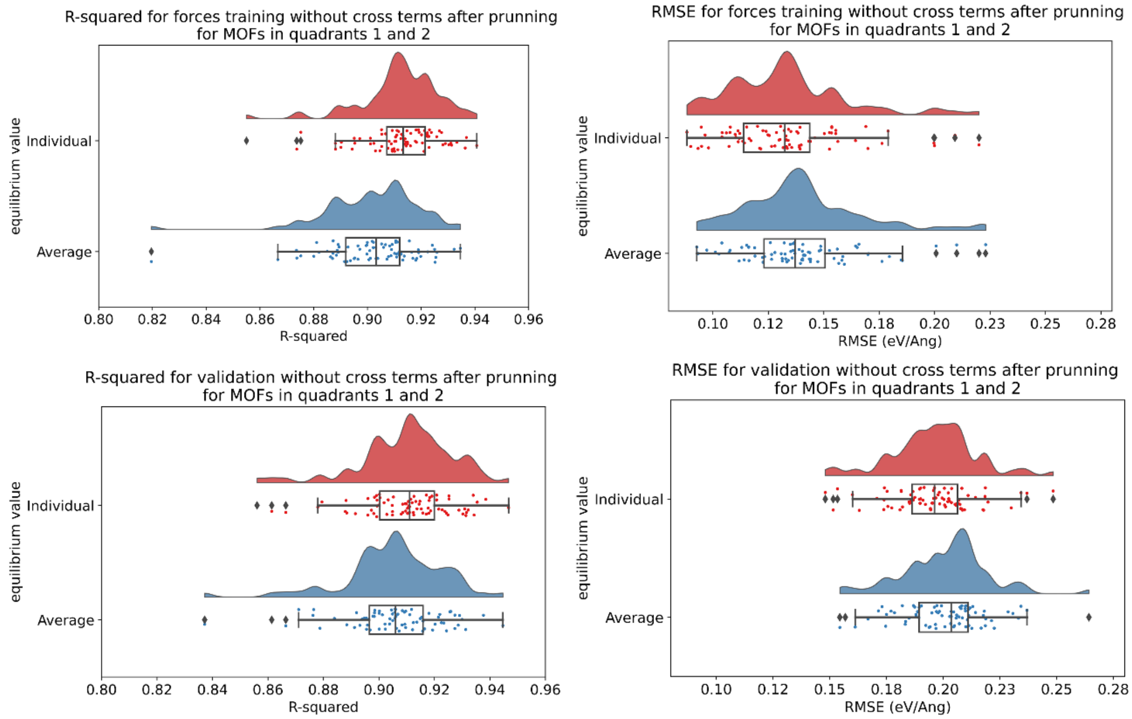

Figure S6: Raincloud plots of R-squared (left panels) and RMSE (right panels) for forces training datasets (top panels) and validation datasets (bottom panels) for MOFs in quadrants 1 and 2 without cross terms and after pruning. The red distributions represent the values for individual equilibrium values, while the blue distributions represent the values for average equilibrium values.

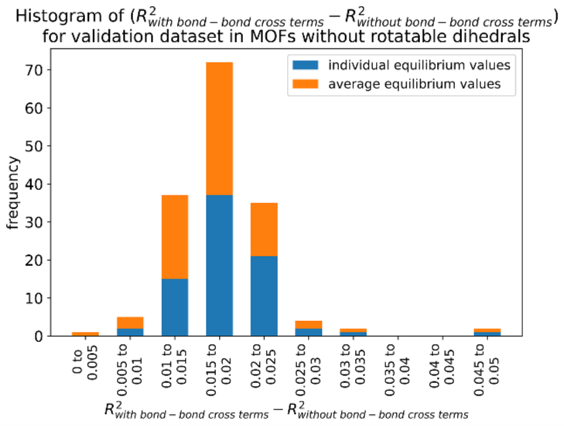

Figure S7: Histogram of difference between R-squared with bond-bond cross terms and R-squared without bond-bond cross terms for MOFs without rotatable dihedrals.

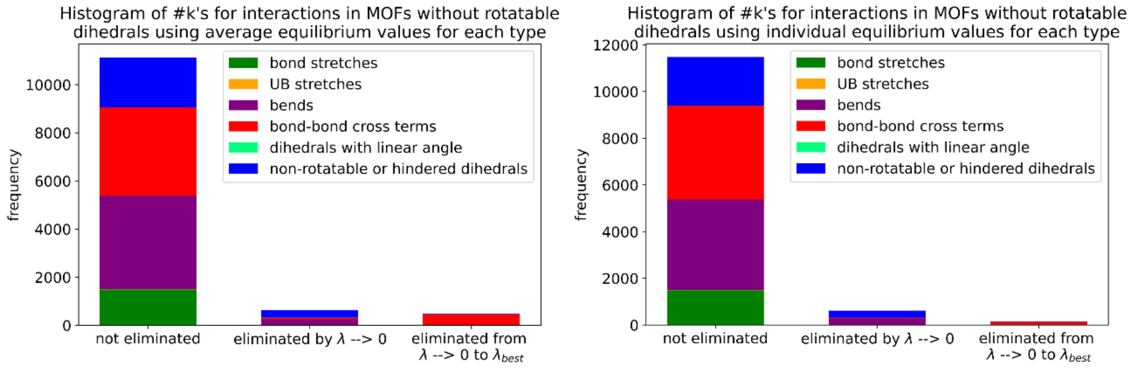

Figure S8: Histograms of force constants eliminated by the bounds or regularization constraints in the LASSO method applied to MOFs in quadrants 1 and 2 for flexibility models including bond-bond cross terms. Data shown in the left (right) panel used average (individual) equilibrium values.

S.9 Images of MOF crystal structures

In the below images, the gold lines indicate the unit cell boundary within the displayed 2×2×2 supercell. The three panels display views along each of the three lattice vectors.

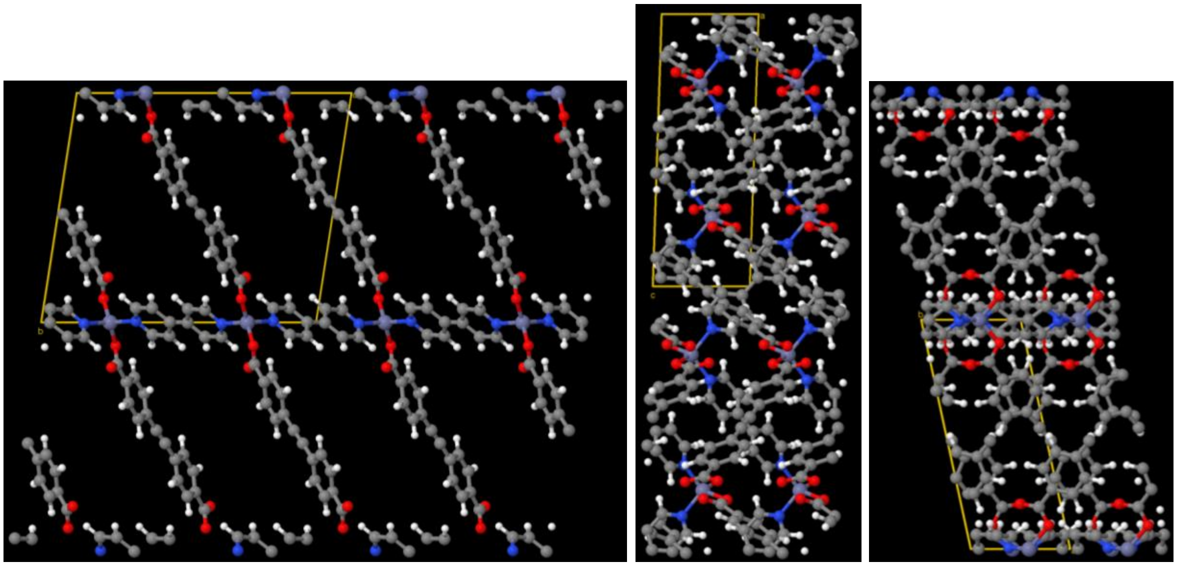

Figure S9: AFITEP contains the chemical elements Zn, H, C, N, and O.

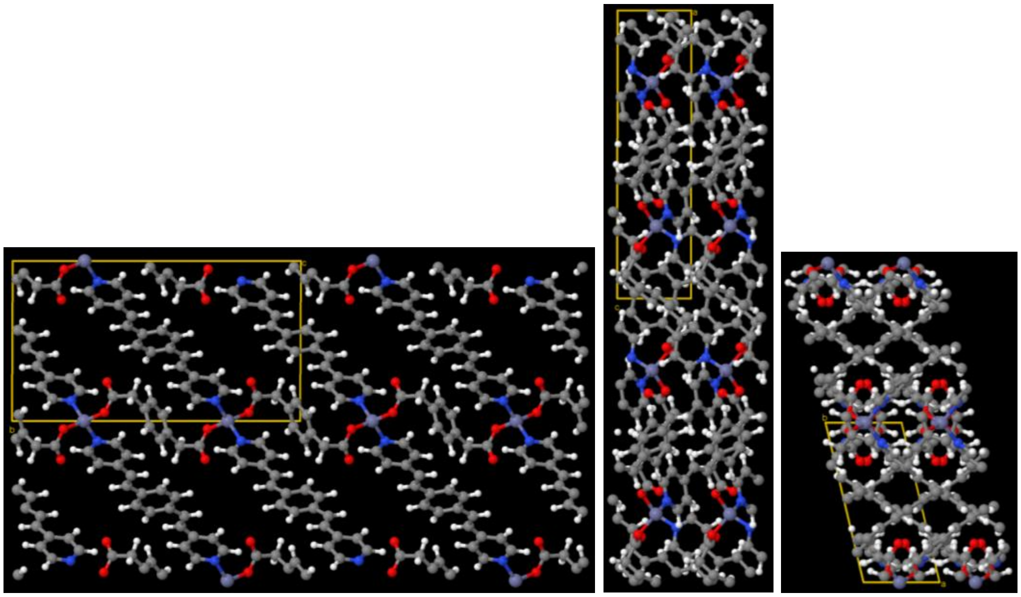

Figure S10: AMOYOR contains the chemical elements Zn, H, C, N, and O.

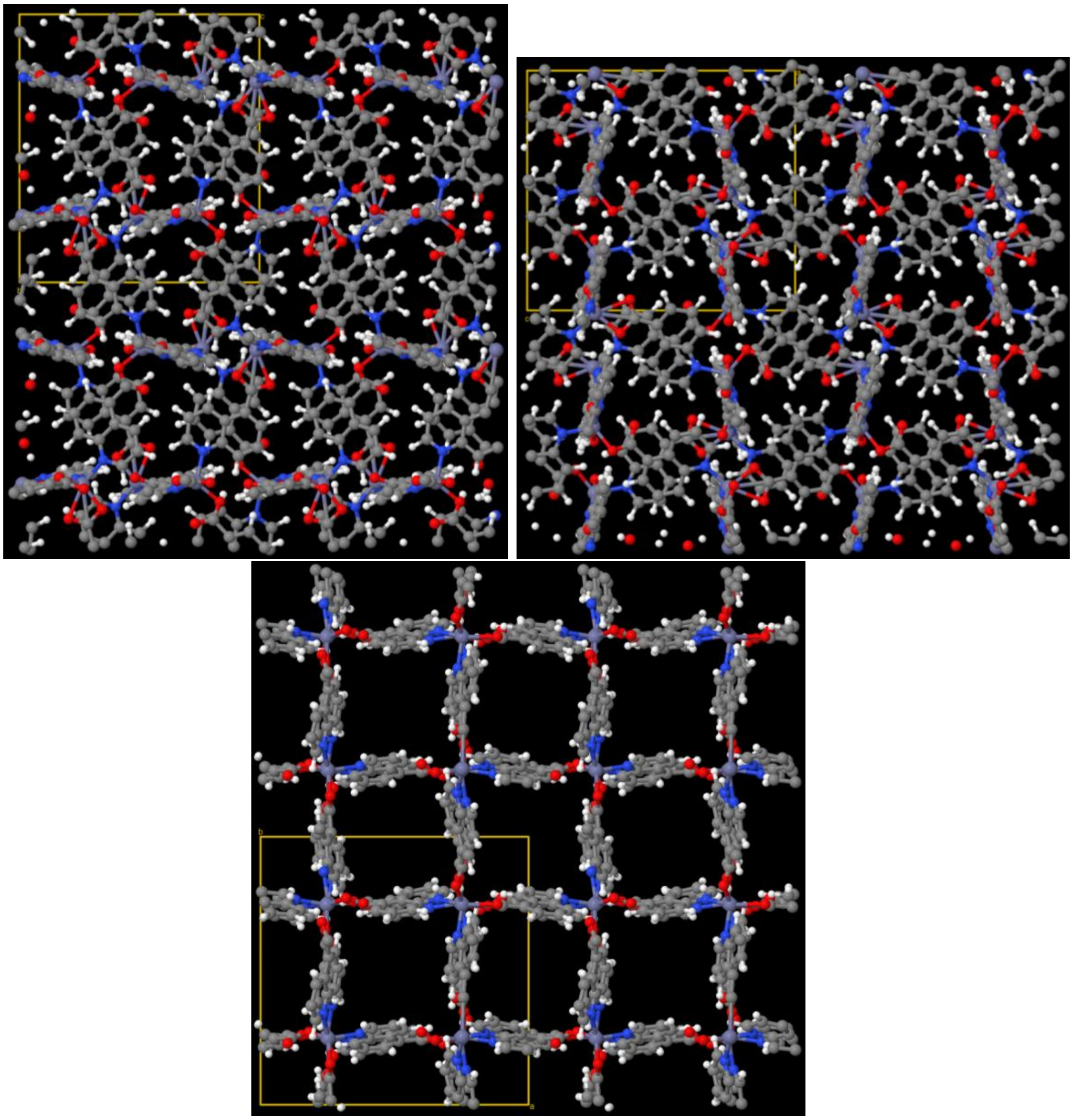

Figure S11: ARAKAH contains the chemical elements O, C, H, Zn, and N.

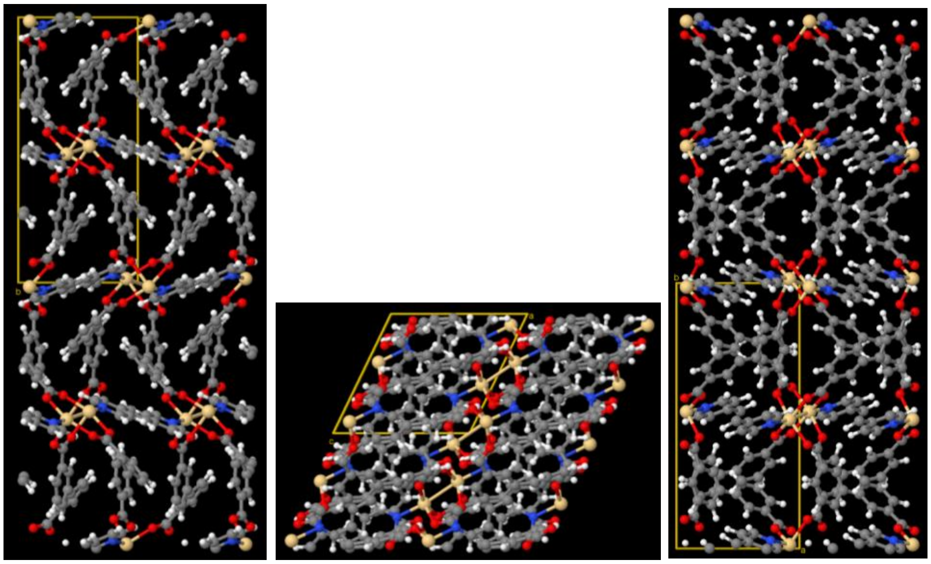

Figure S12: ARIBOS contains the chemical elements Cd, O, C, H, and N.

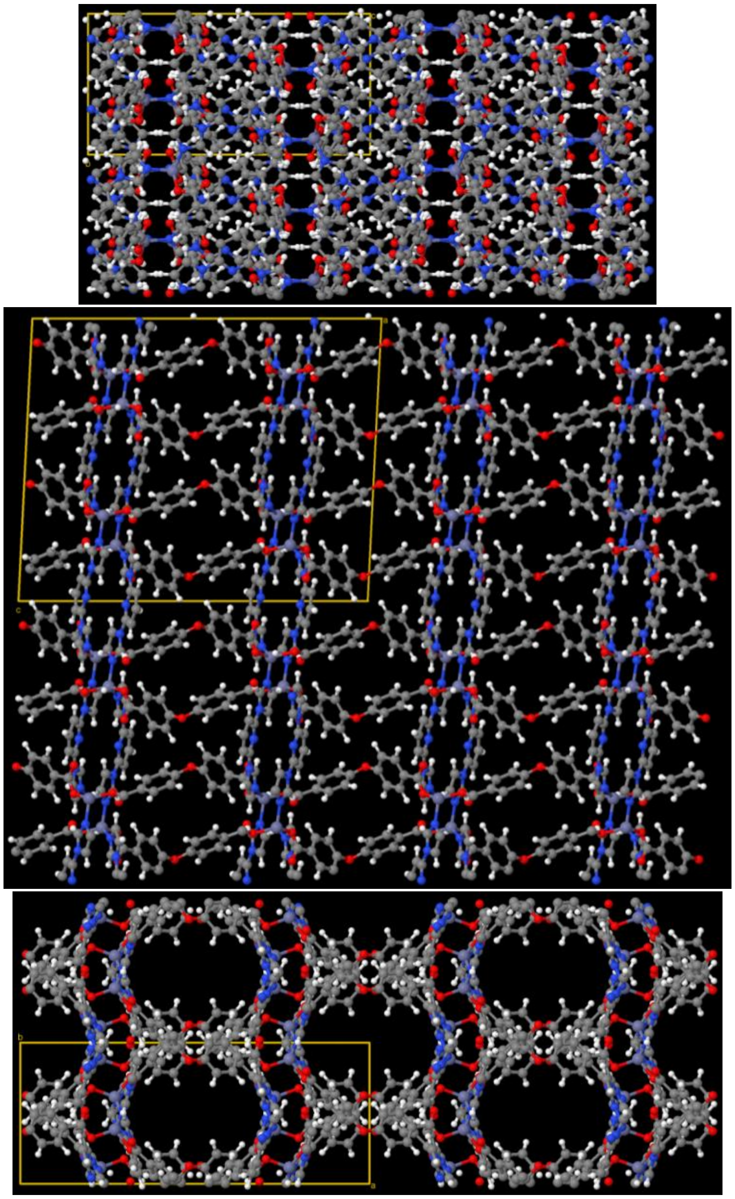

Figure S13: ATOBIW contains the chemical elements C, H, O, Zn, and N.

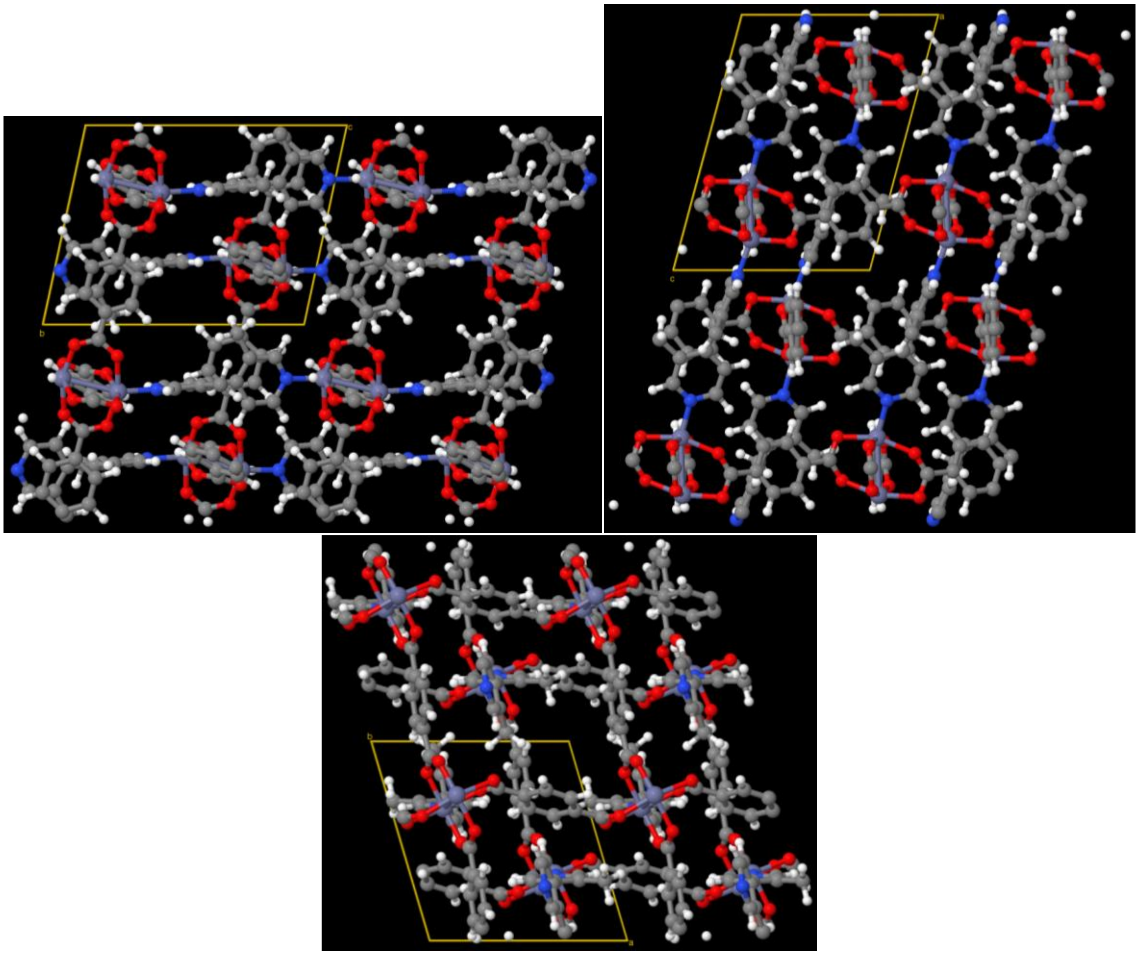

Figure S14: ATOXEN contains the chemical elements C, H, O, Zn, and N.

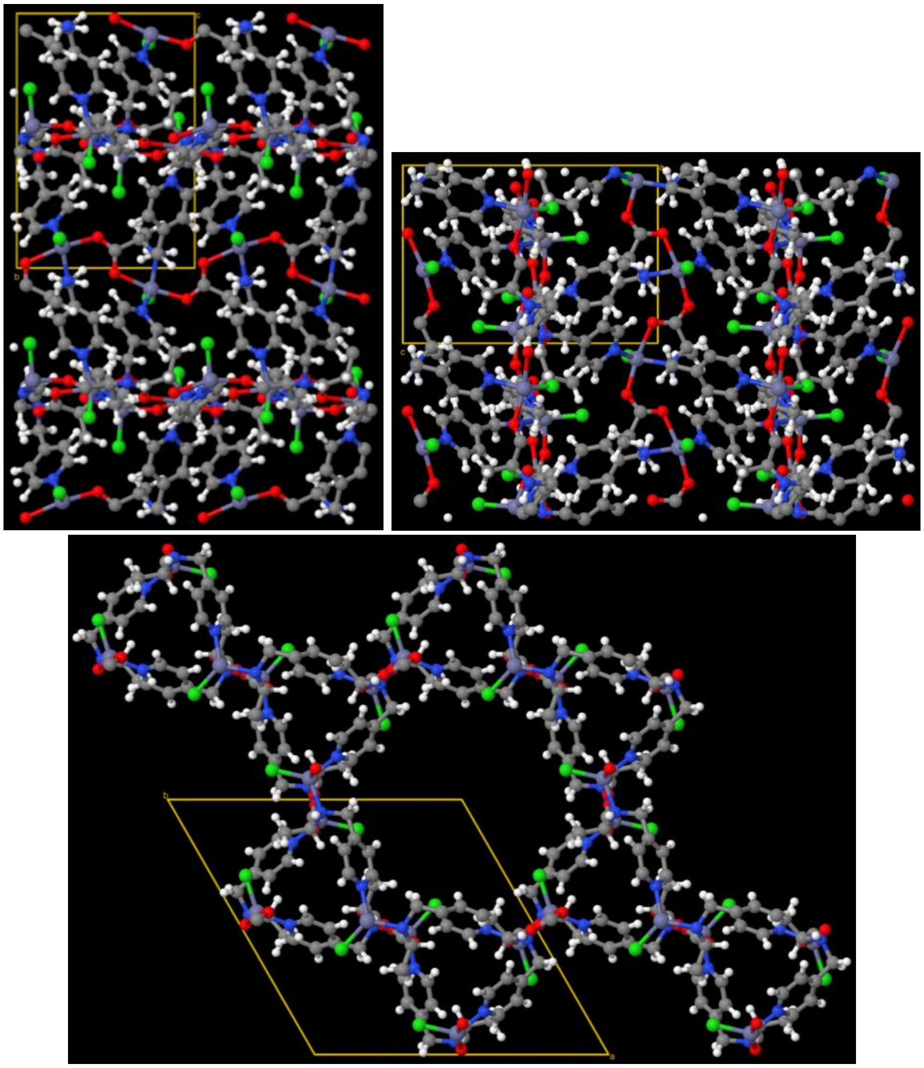

Figure S15: BEPMEQ contains the chemical elements C, H, N, Zn, O, and Cl.

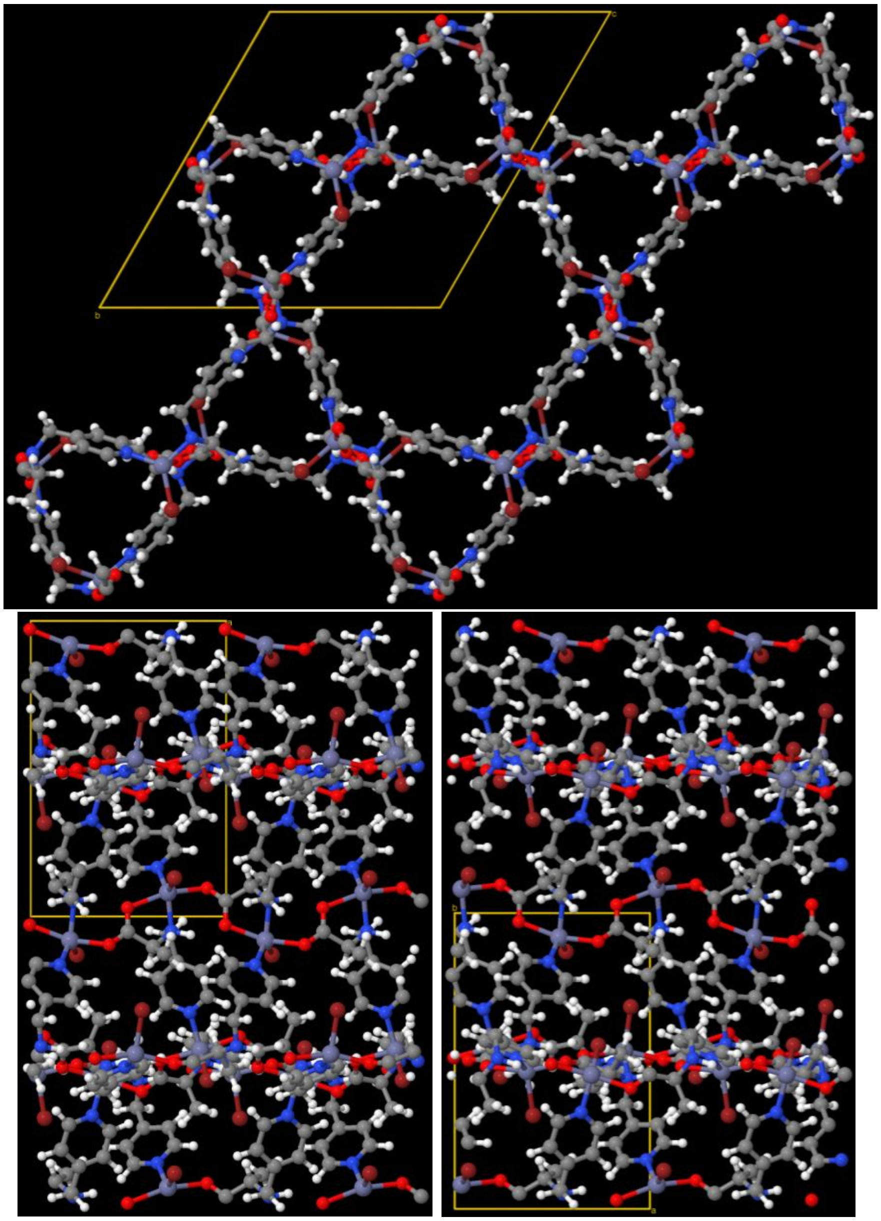

Figure S16: BEPNUH contains the chemical elements Zn, H, C, Br, N, and O.

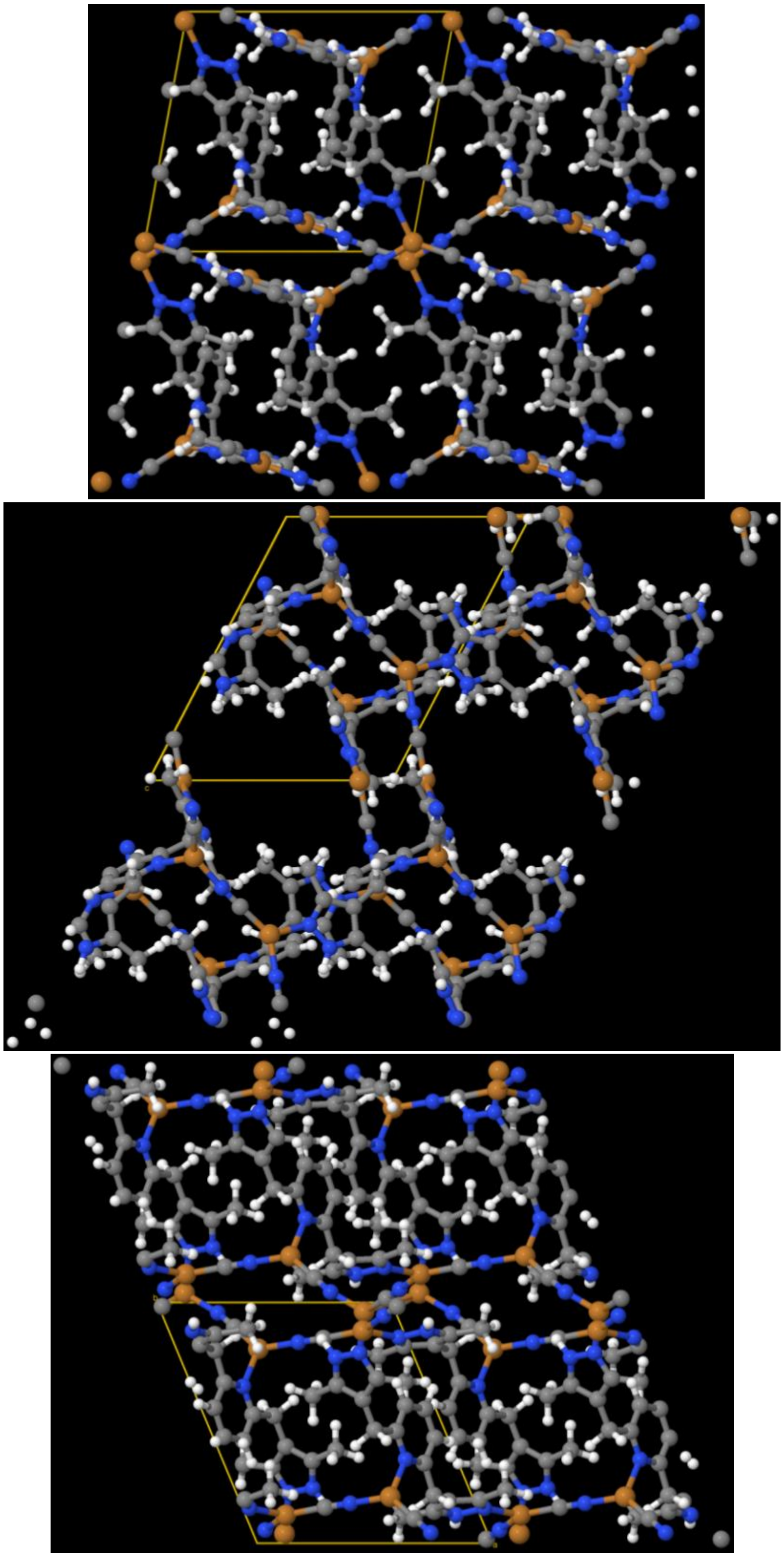

Figure S17: BEPVID contains the chemical elements Cu, H, C, and N.

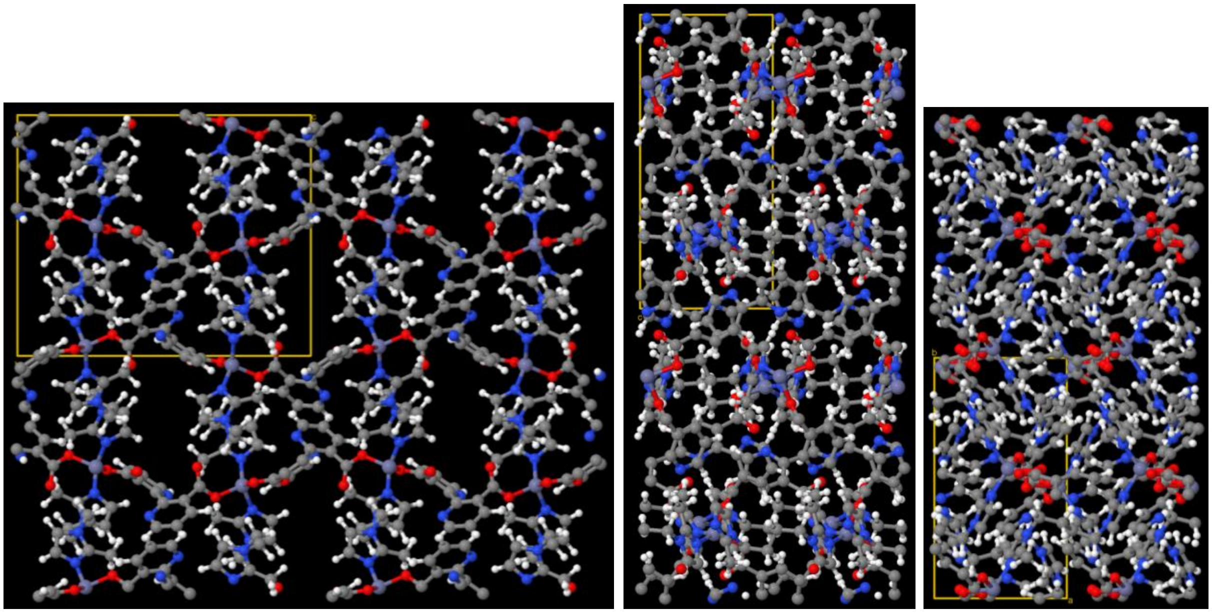

Figure S18: BIBBUL contains the chemical elements Zn, H, C, N, and O.

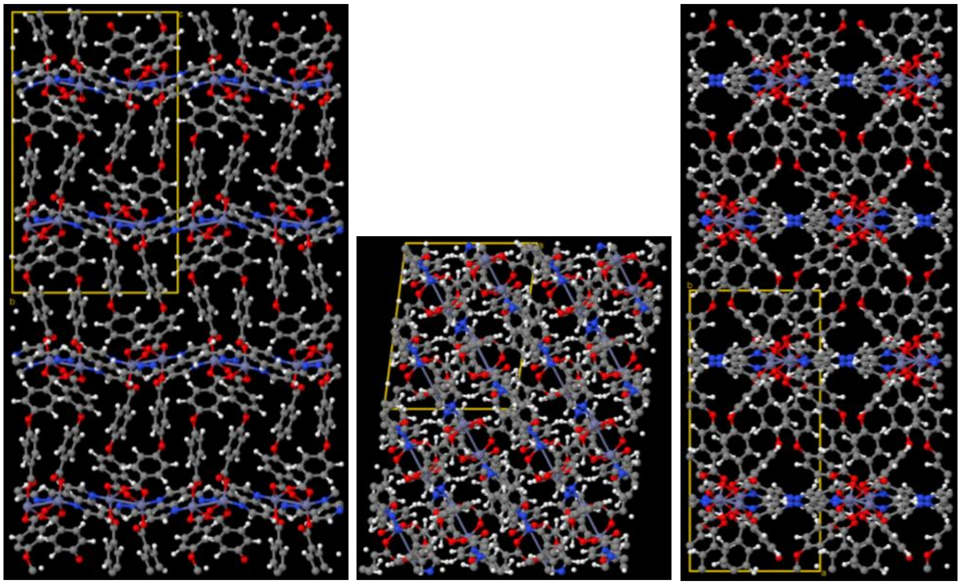

Figure S19: BOMCOX contains the chemical elements Zn, O, N, C, and H.

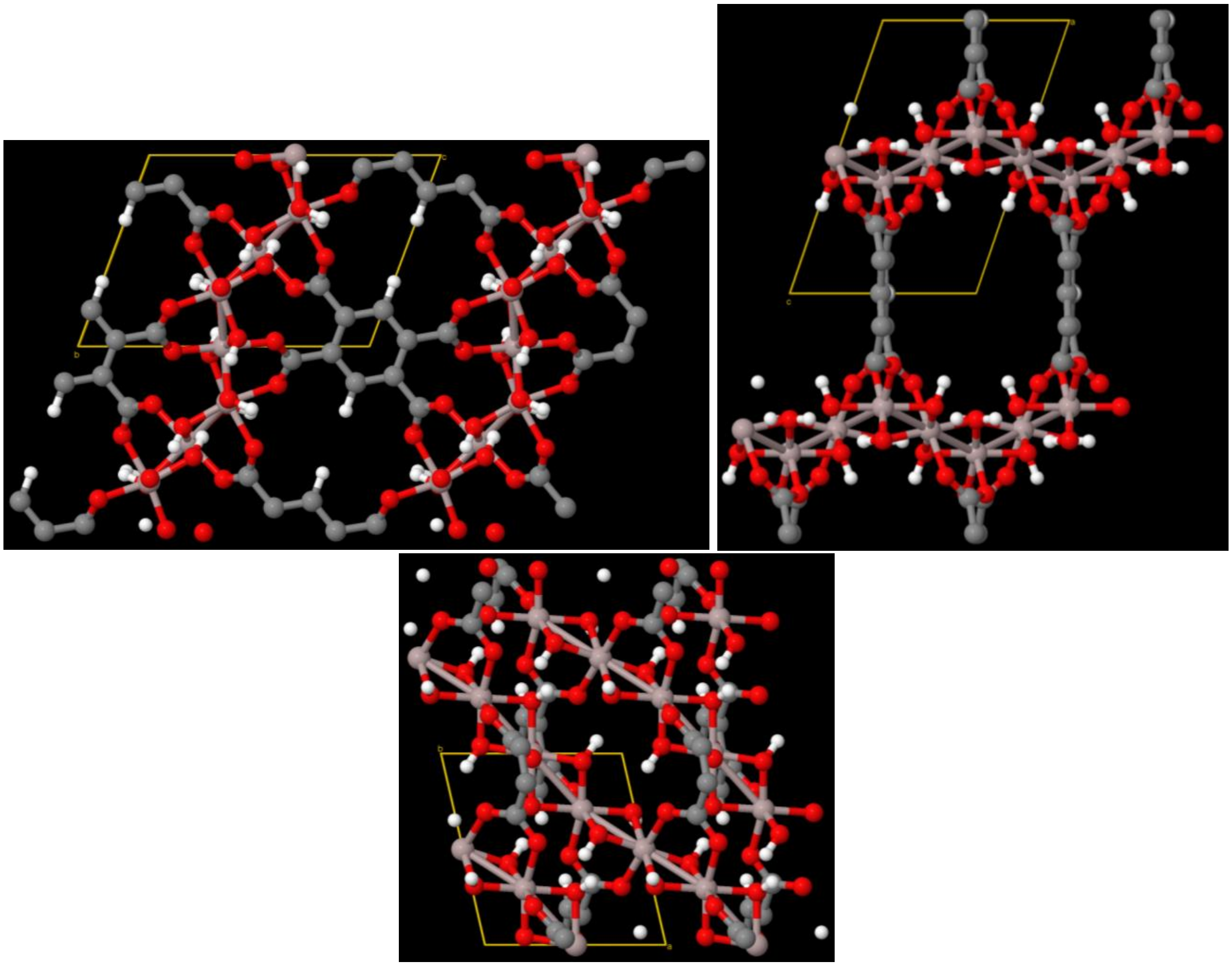

Figure S20: BUSQIQ contains the chemical elements Al, H, C, and O.

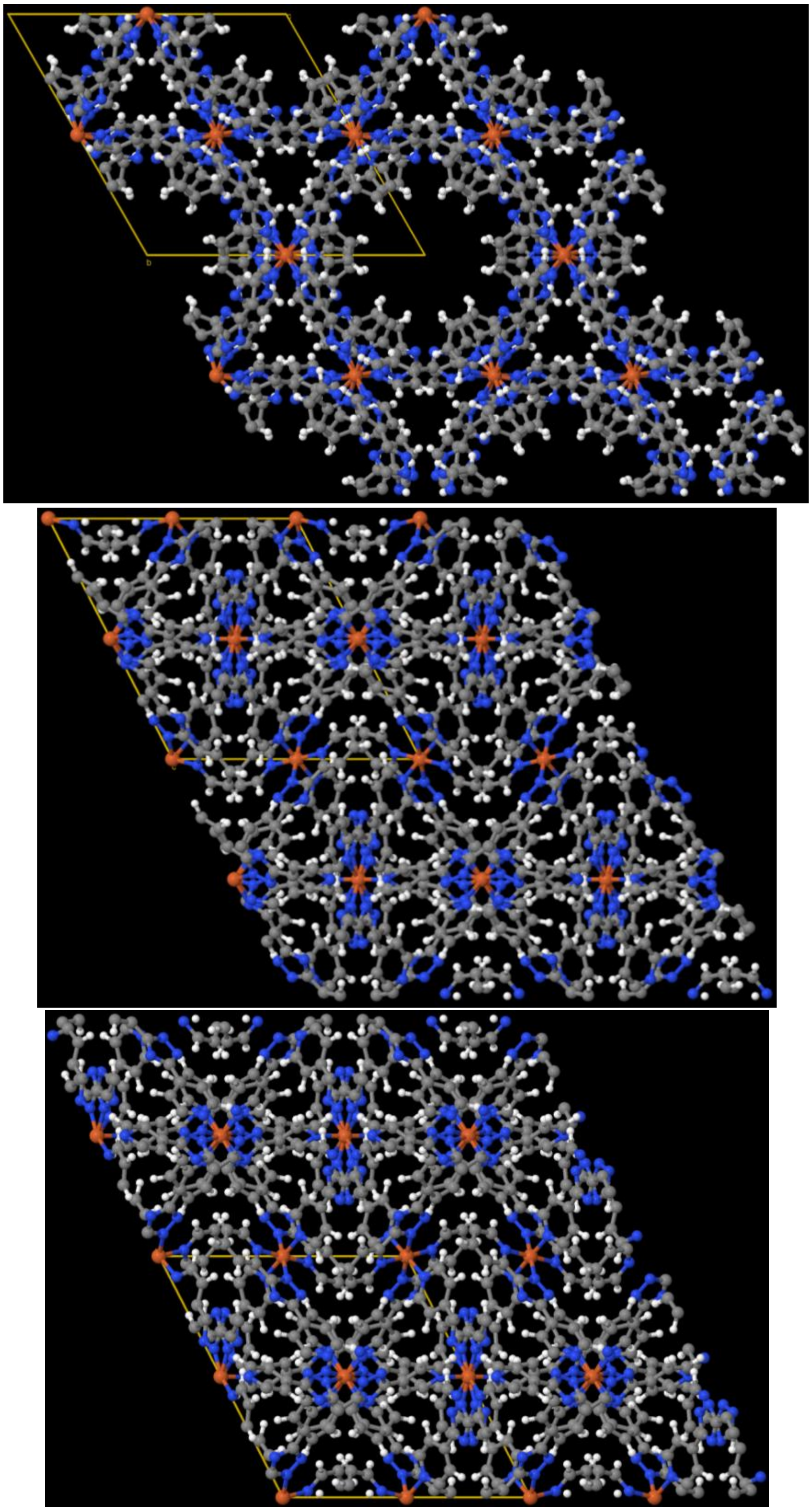

Figure S21: BUVWOF03 contains the chemical elements Fe, H, C, and N.

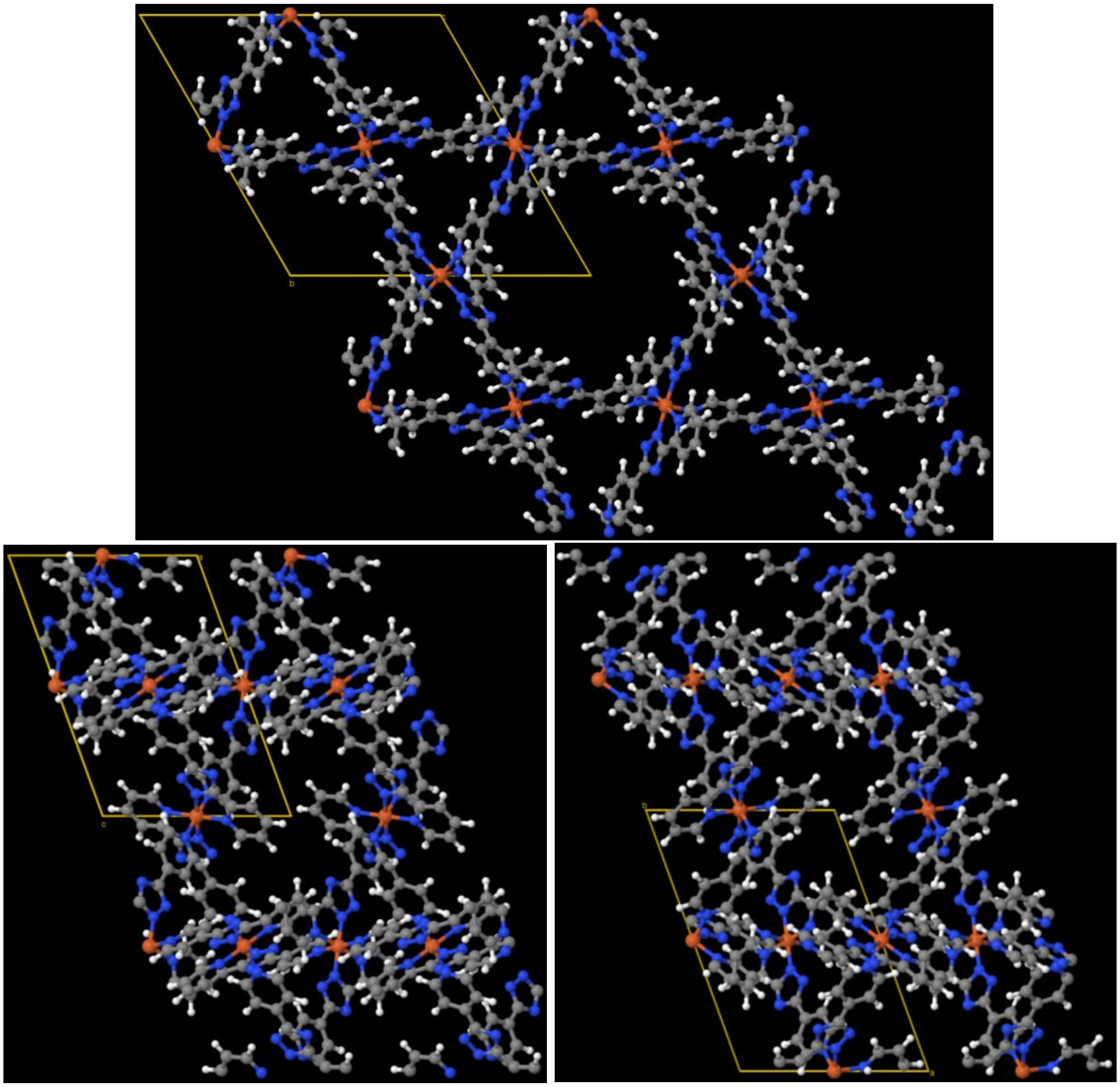

Figure S22: BUVYIB contains the chemical elements Fe, H, C, and N.

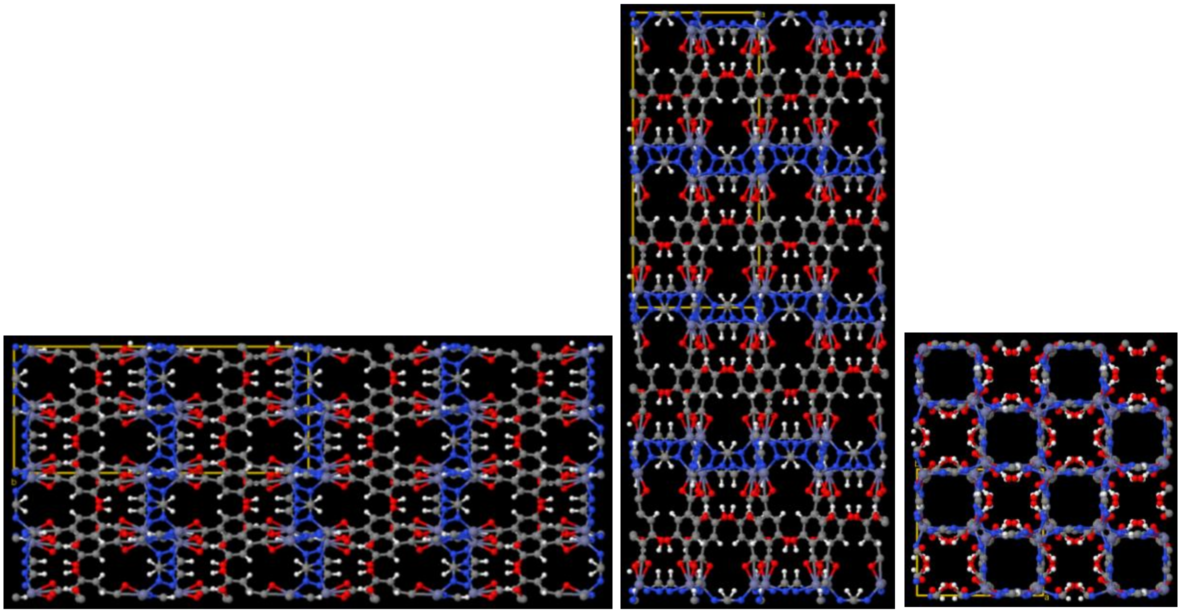

Figure S23: BUYDEG contains the chemical elements C, H, N, O, and Zn.

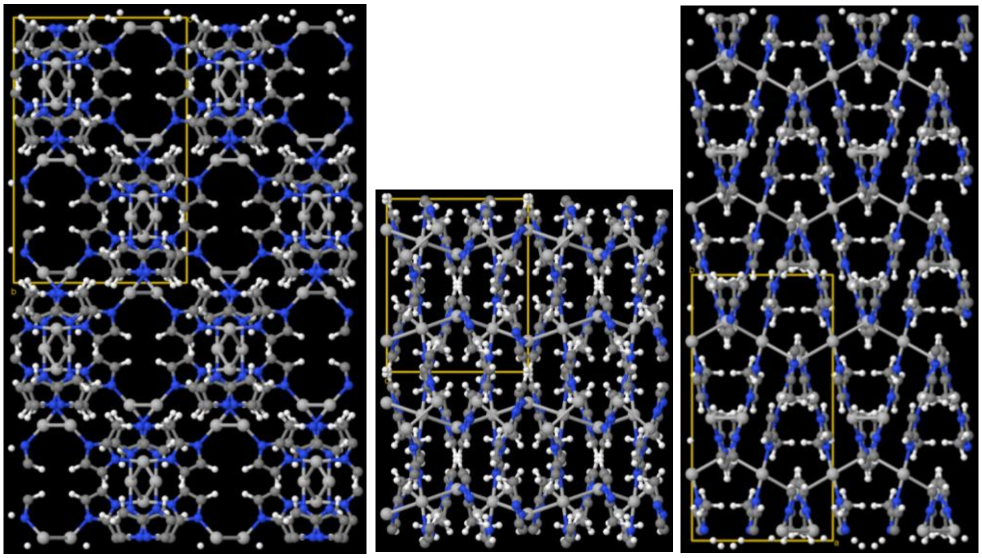

Figure S24: CEGDUO contains the chemical elements Ag, H, C, and N.

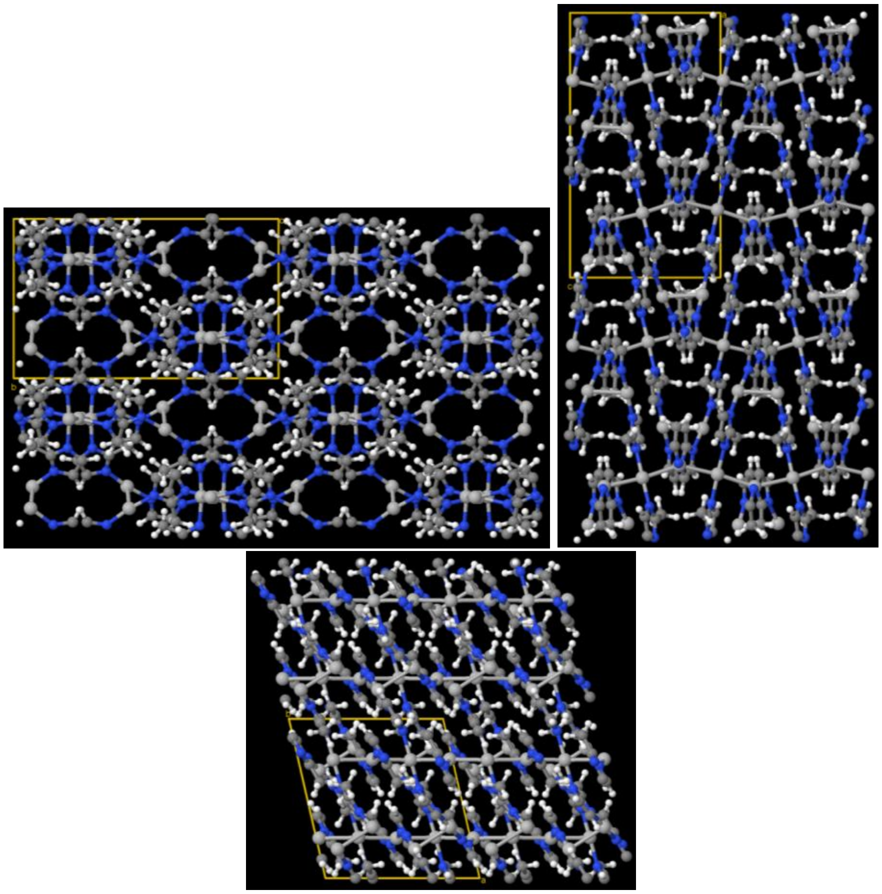

Figure S25: CEGFAW contains the chemical elements Ag, H, C, and N.

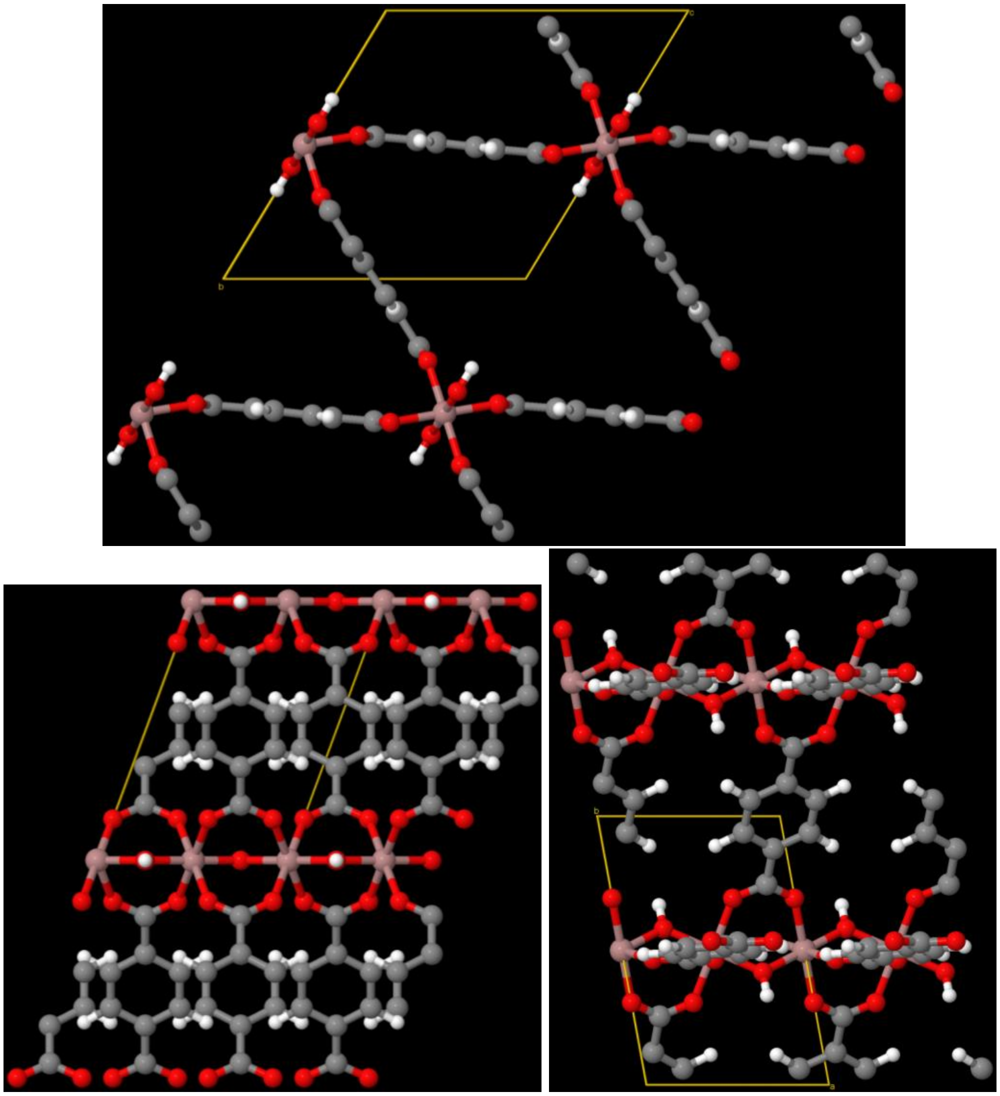

Figure S26: COMDOY contains the chemical elements Ga, H, C, and O.

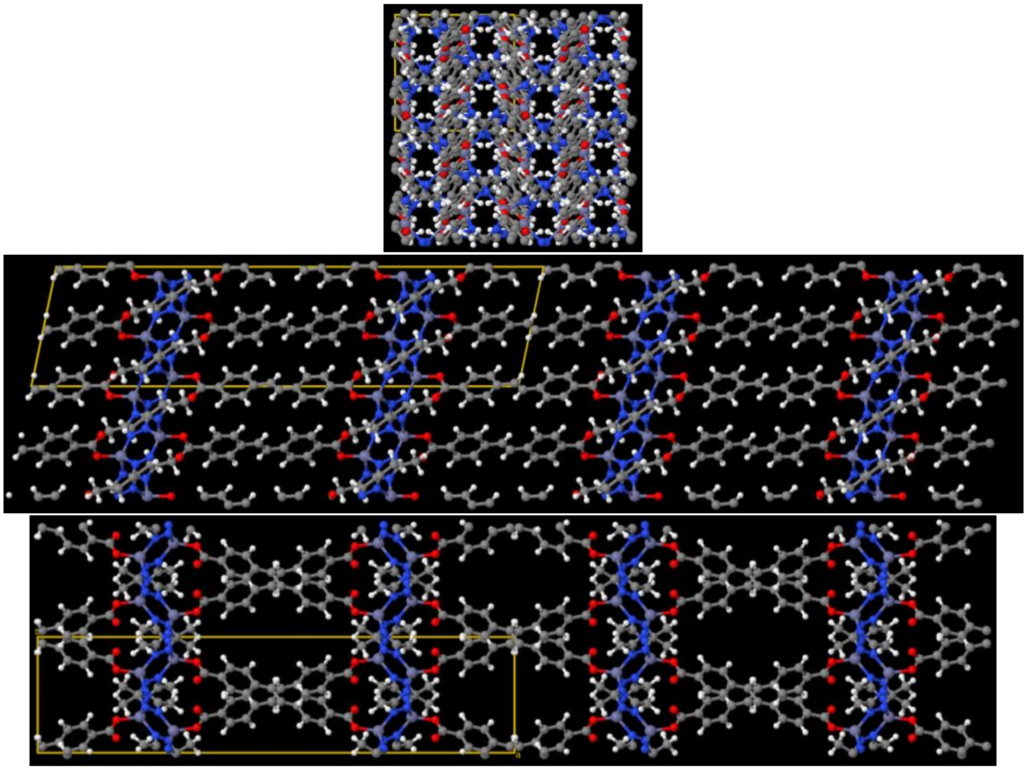

Figure S27: CUQRIR contains the chemical elements Zn, N, O, C, and H.

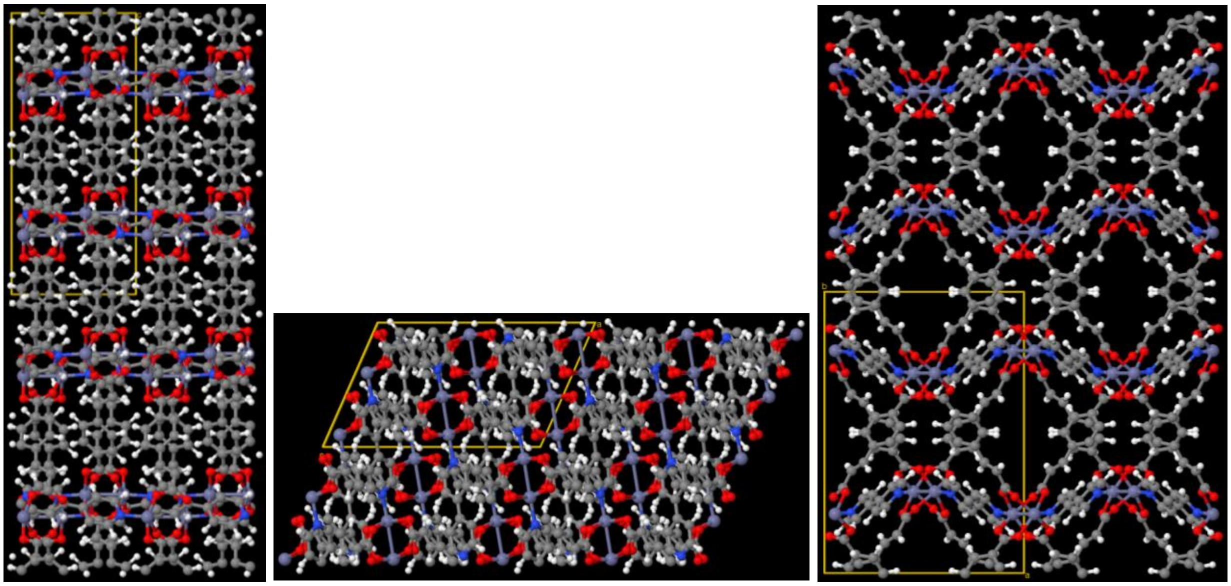

Figure S28: DIXJIF contains the chemical elements Zn, O, C, H, and N.

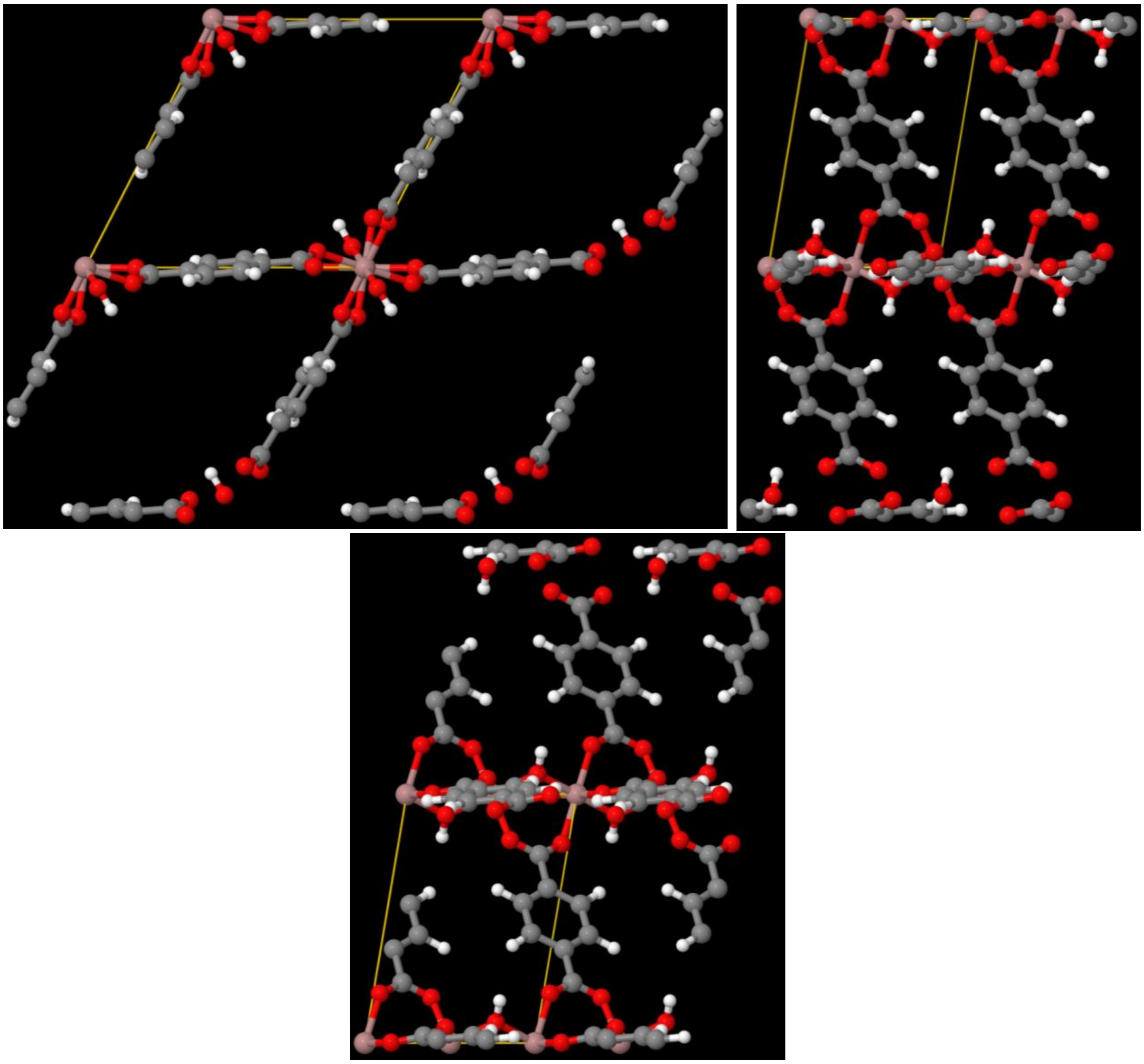

Figure S29: DONNEA contains the chemical elements Ga, H, C, and O.

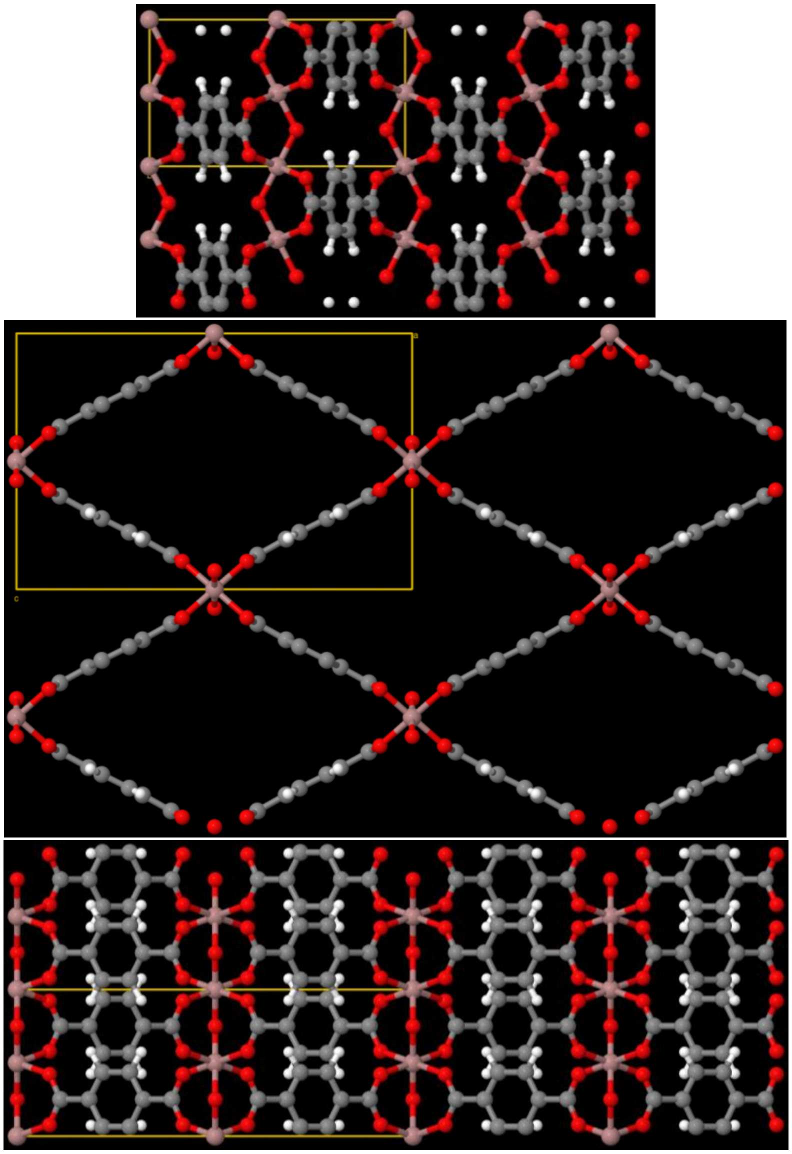

Figure S30: DONNIE contains the chemical elements O, C, H, and Ga.

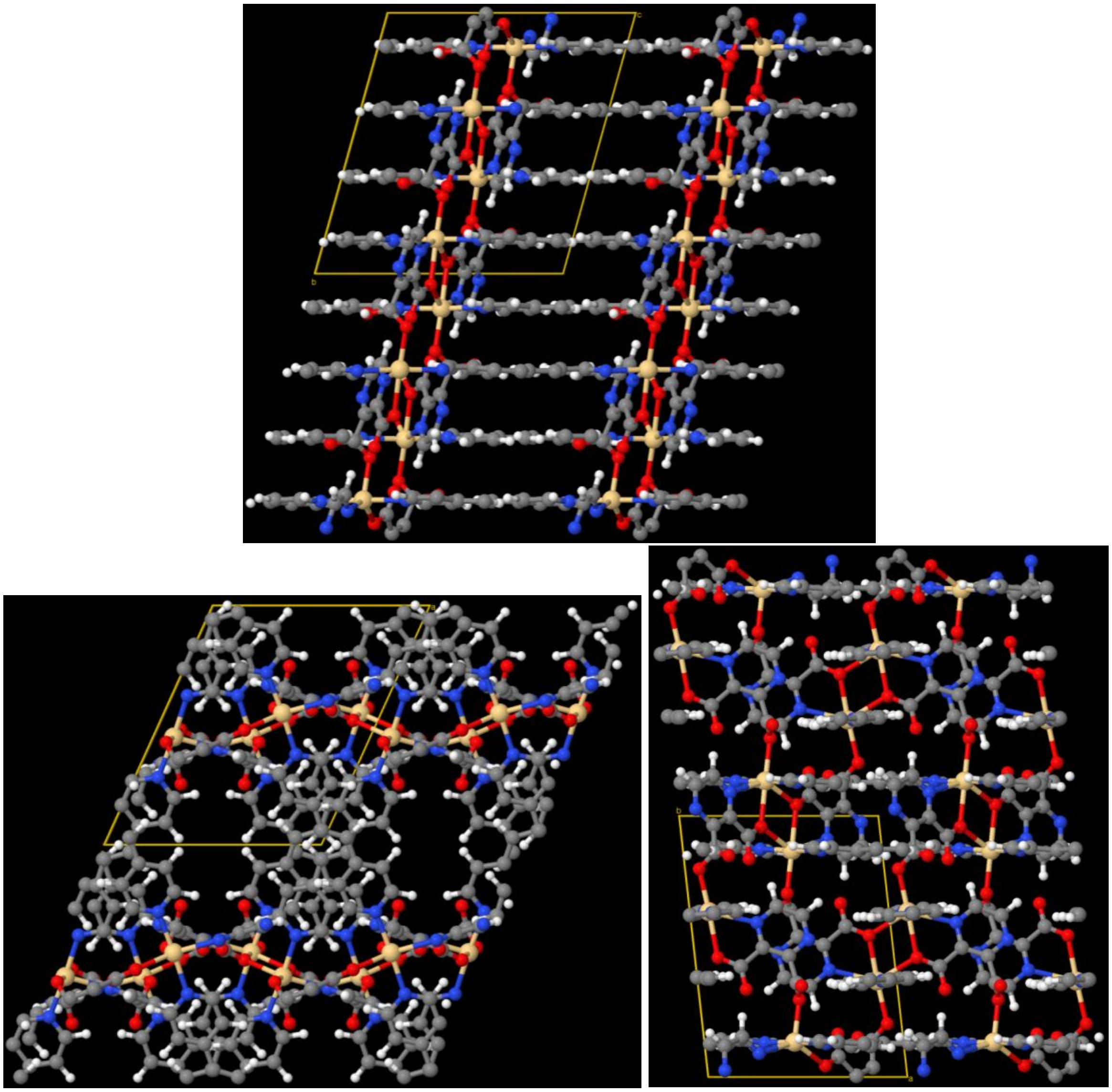

Figure S31: EBEMII contains the chemical elements Cd, H, C, N, and O.

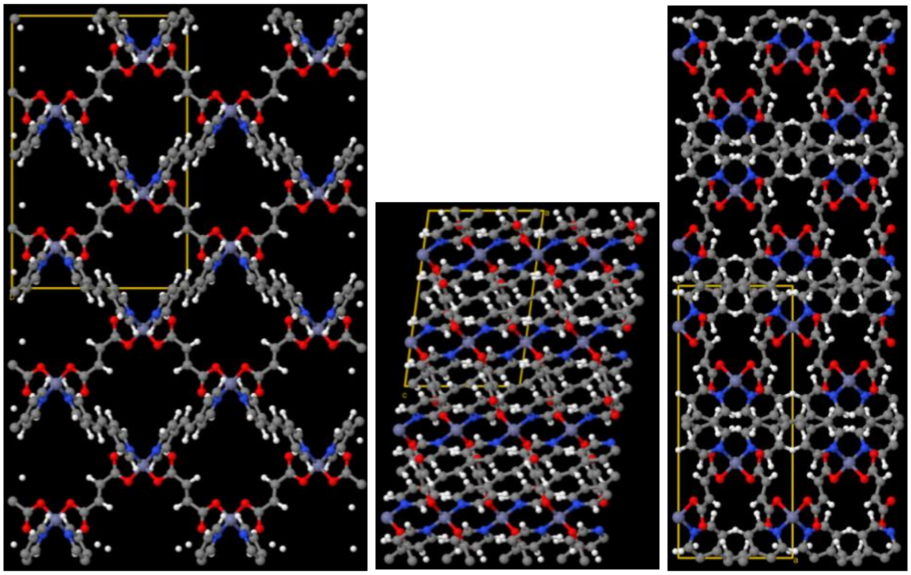

Figure S32: EBOBIJ contains the chemical elements O, N, C, H, and Zn.

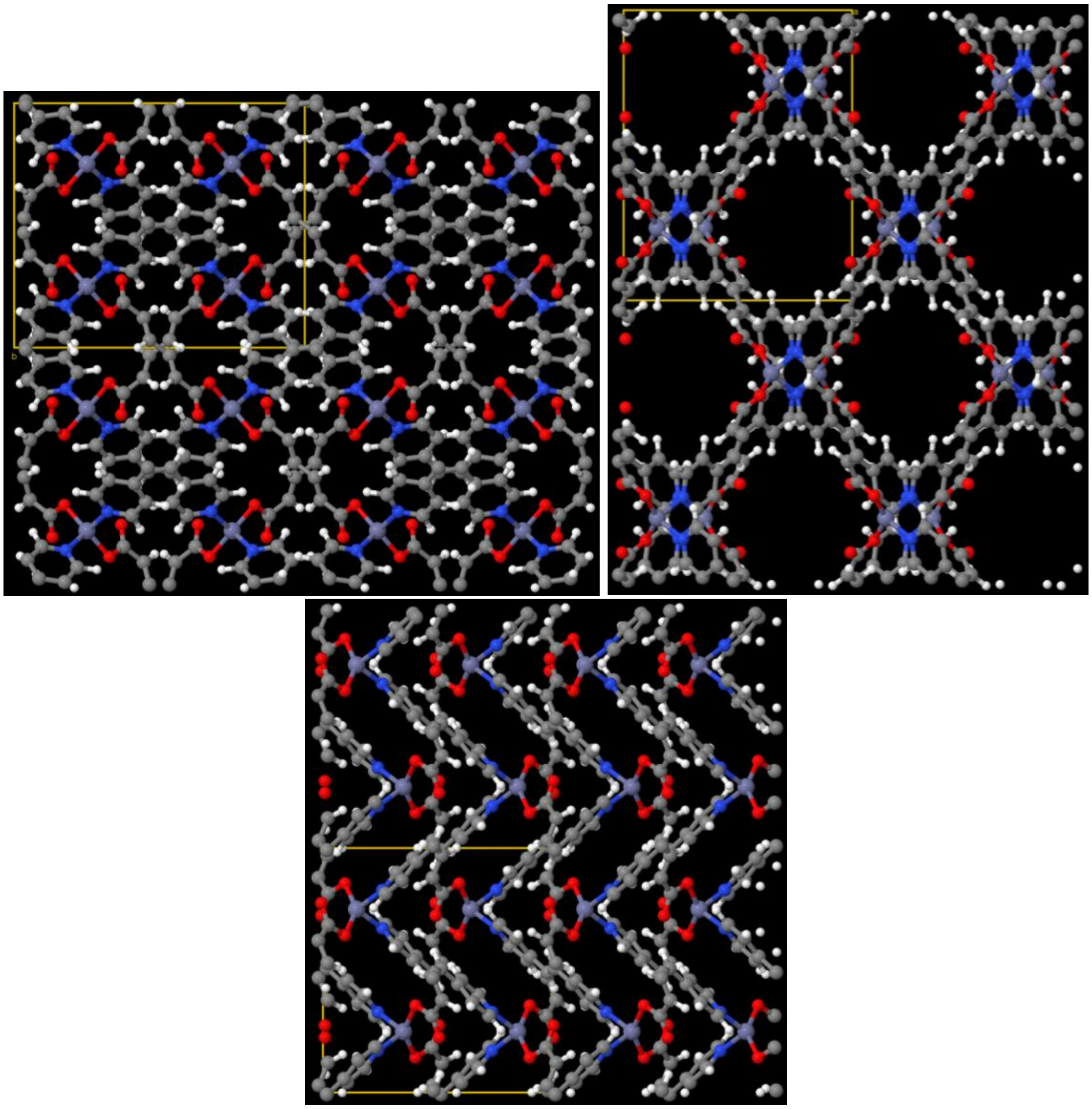

Figure S33: EBOBUV contains the chemical elements C, H, O, Zn, and N.

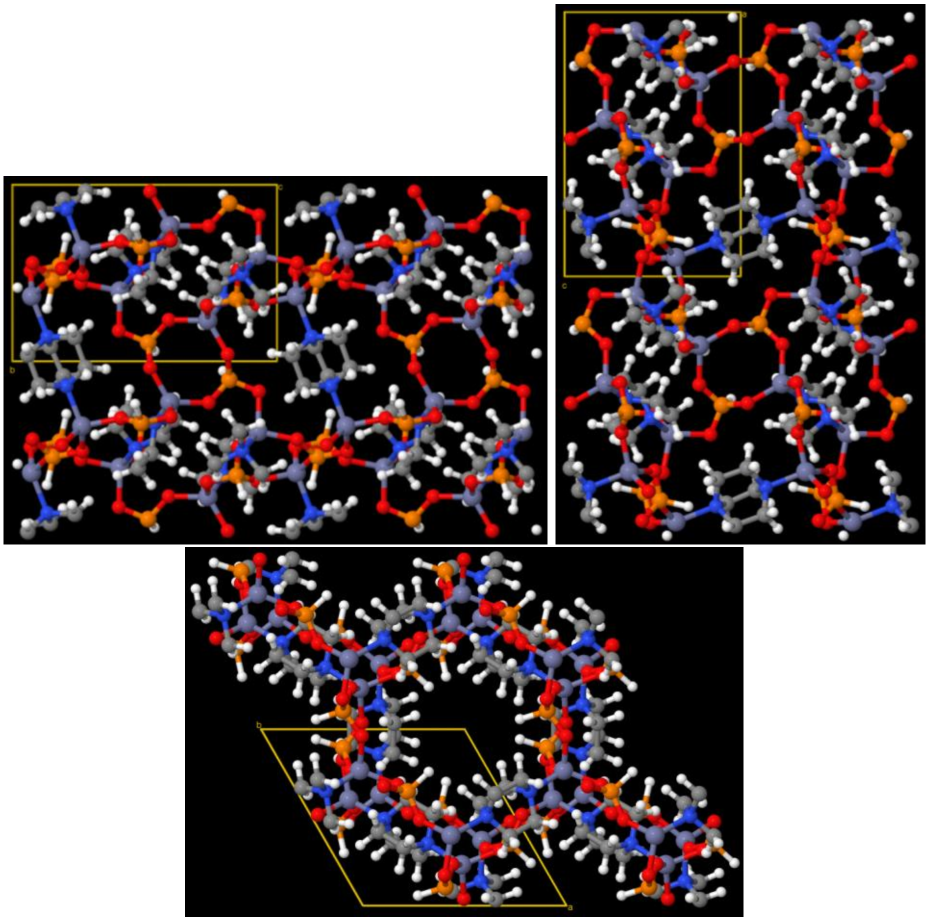

Figure S34: EKEHUZ contains the chemical elements Zn, P, H, O, N, and C.

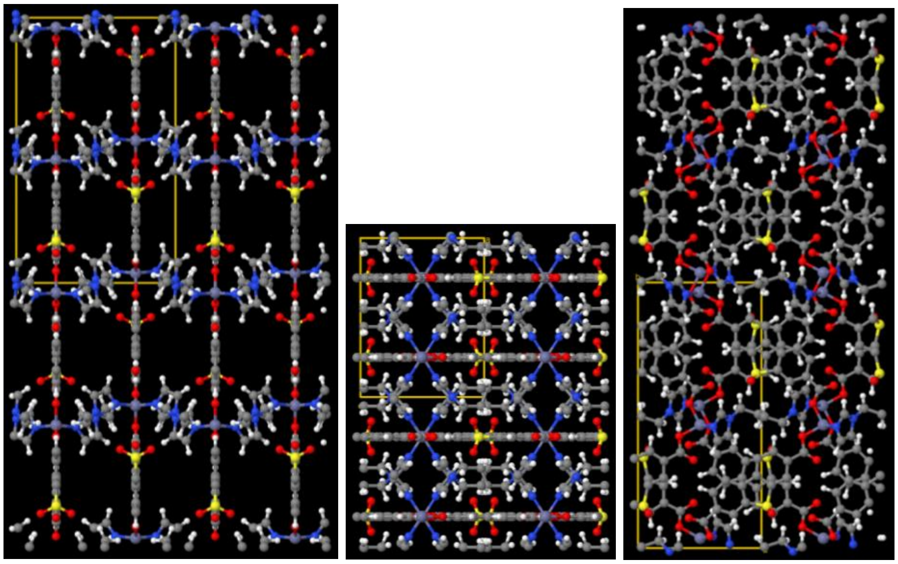

Figure S35: EMIVAY contains the chemical elements Zn, H, N, C, S, and O.

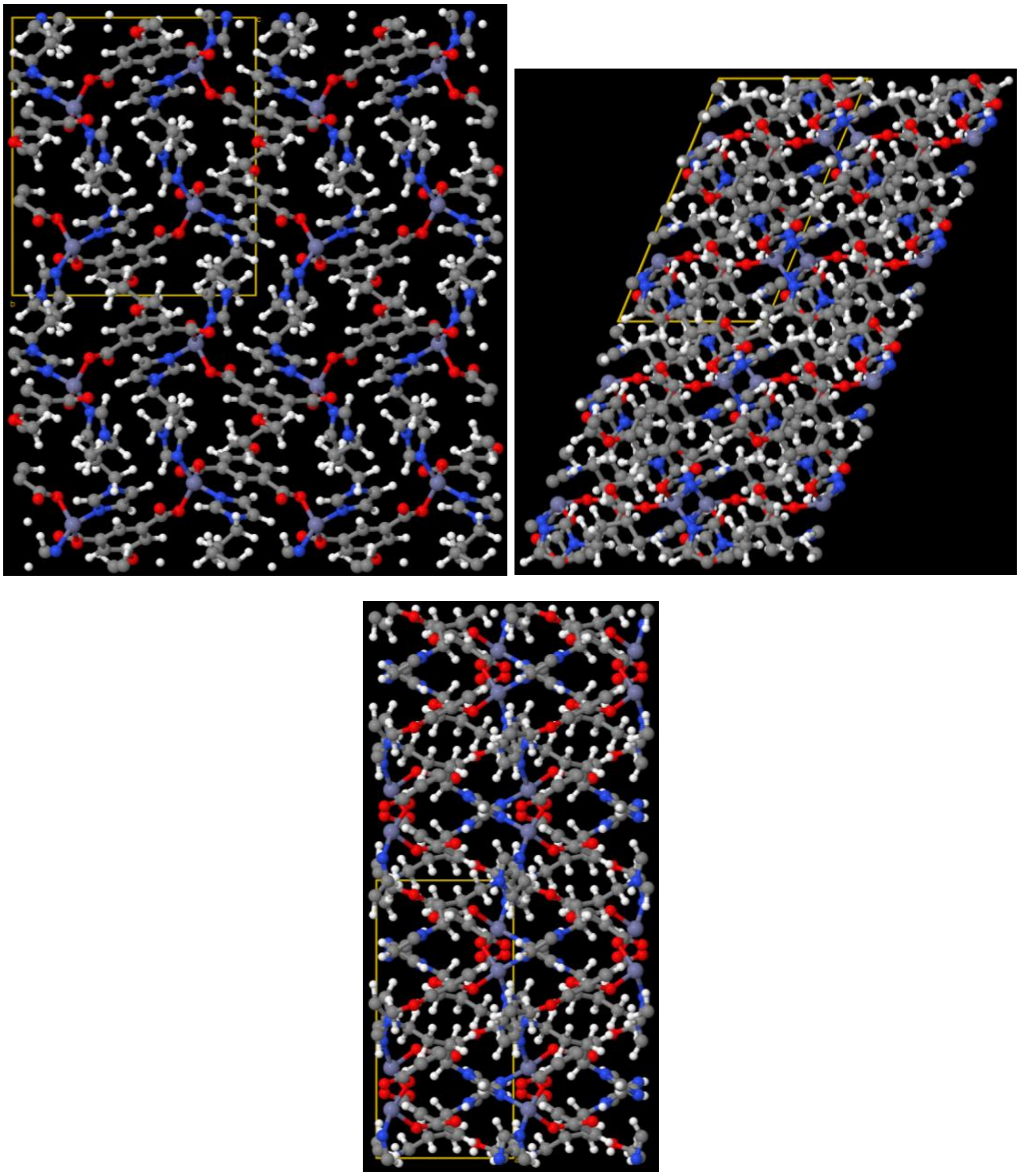

Figure S36: EMOFIX contains the chemical elements Zn, O, N, C, and H.

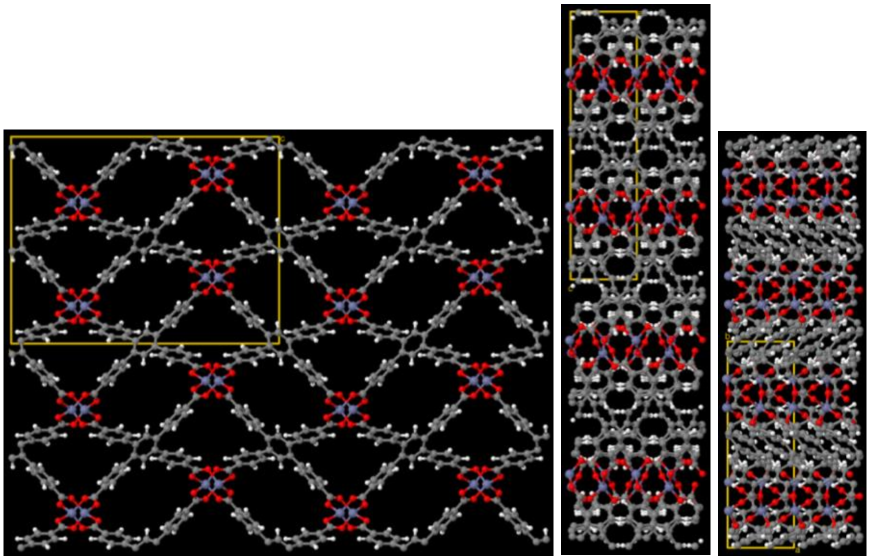

Figure S37: ESEDAJ contains the chemical elements C, H, O, and Zn.

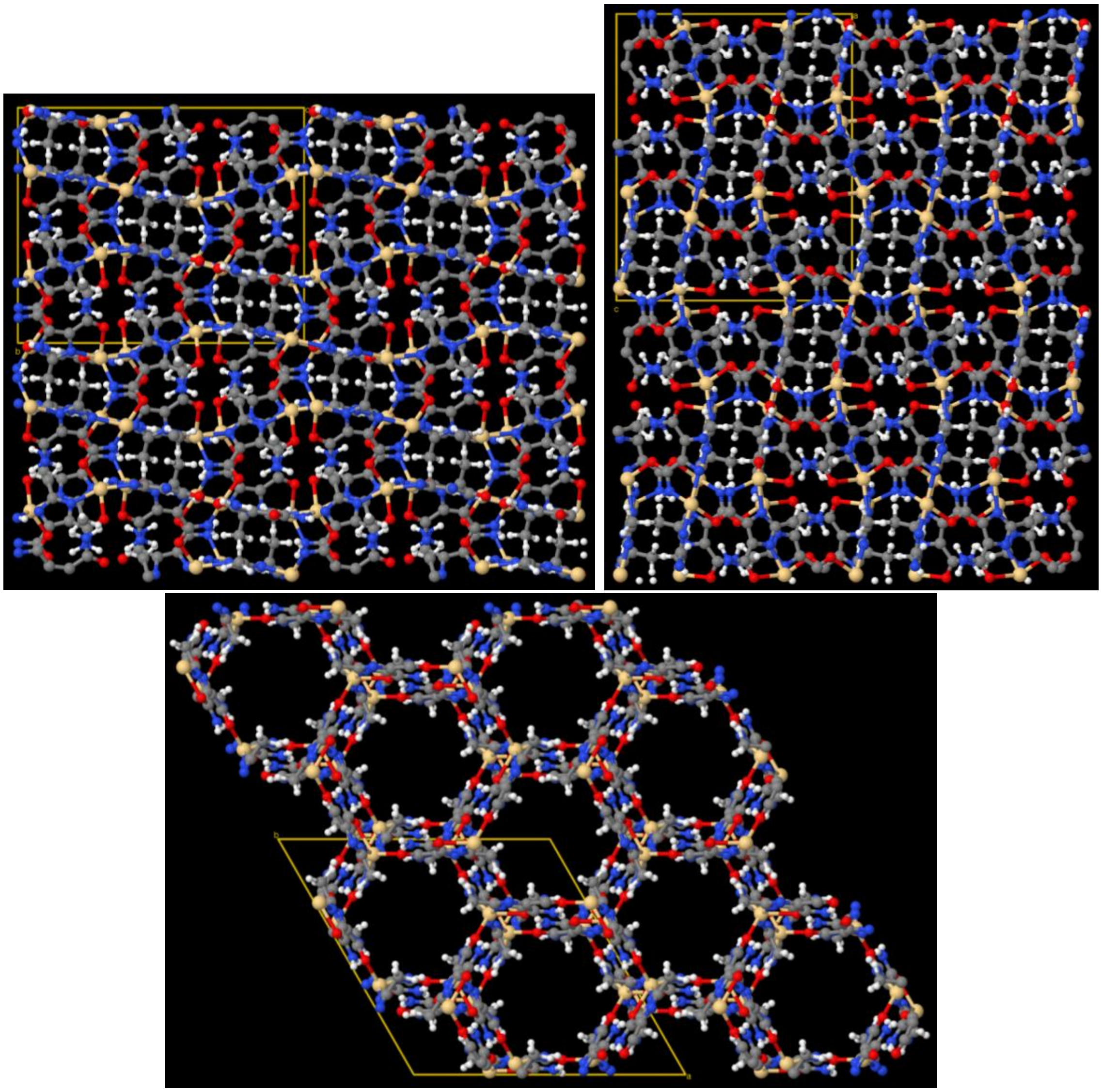

Figure S38: ESIFIX contains the chemical elements C, H, Cd, N, and O.

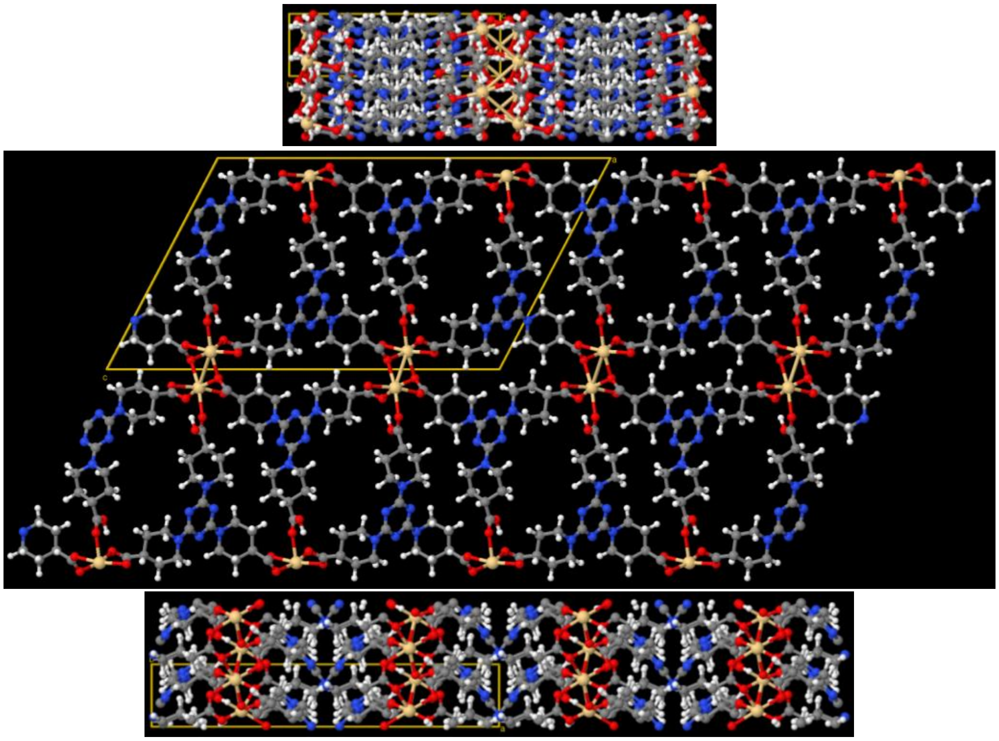

Figure S39: EWUGEK contains the chemical elements C, H, N, O, and Cd.

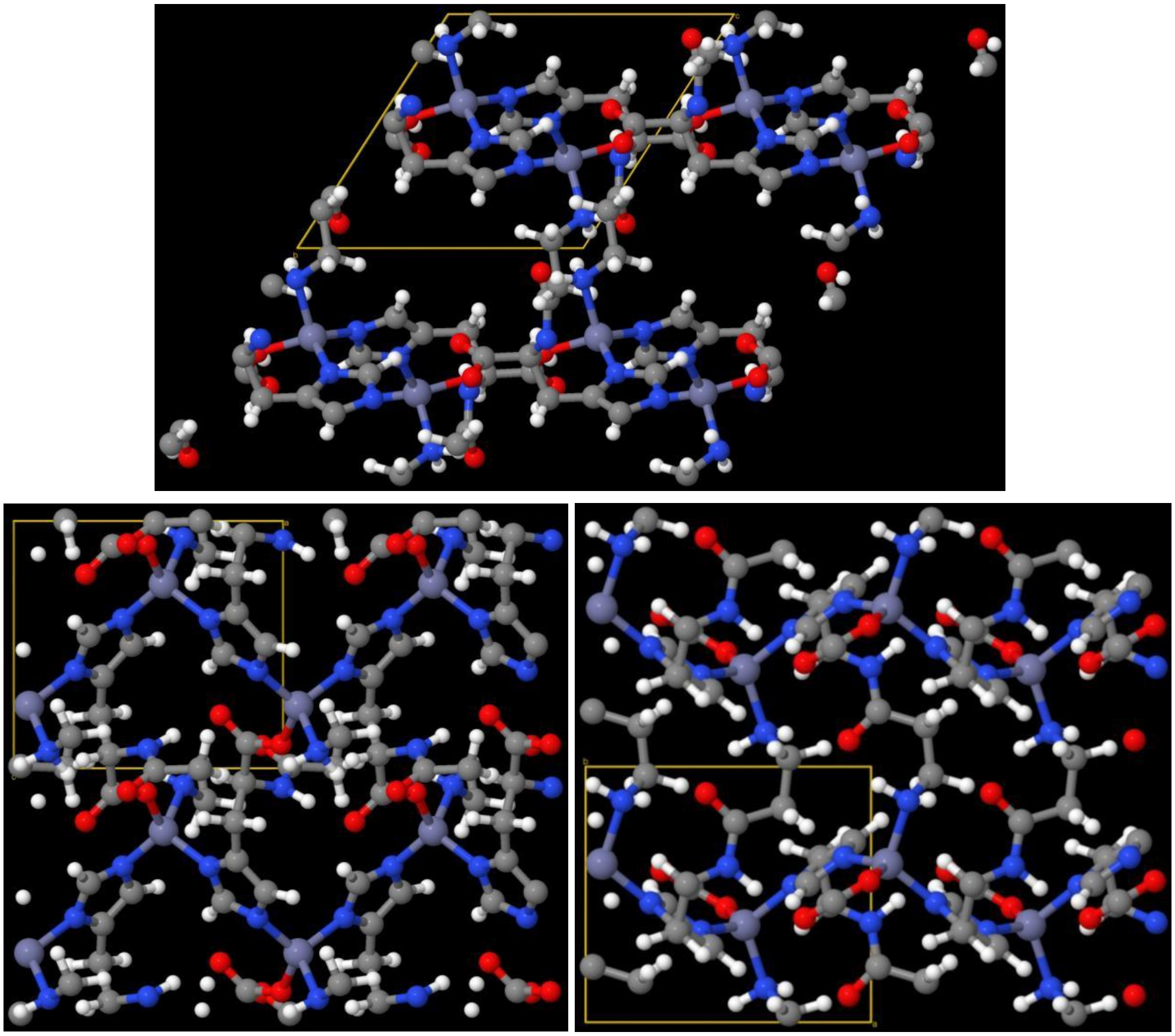

Figure S40: FIPWOS contains the chemical elements Zn, H, C, N, and O.

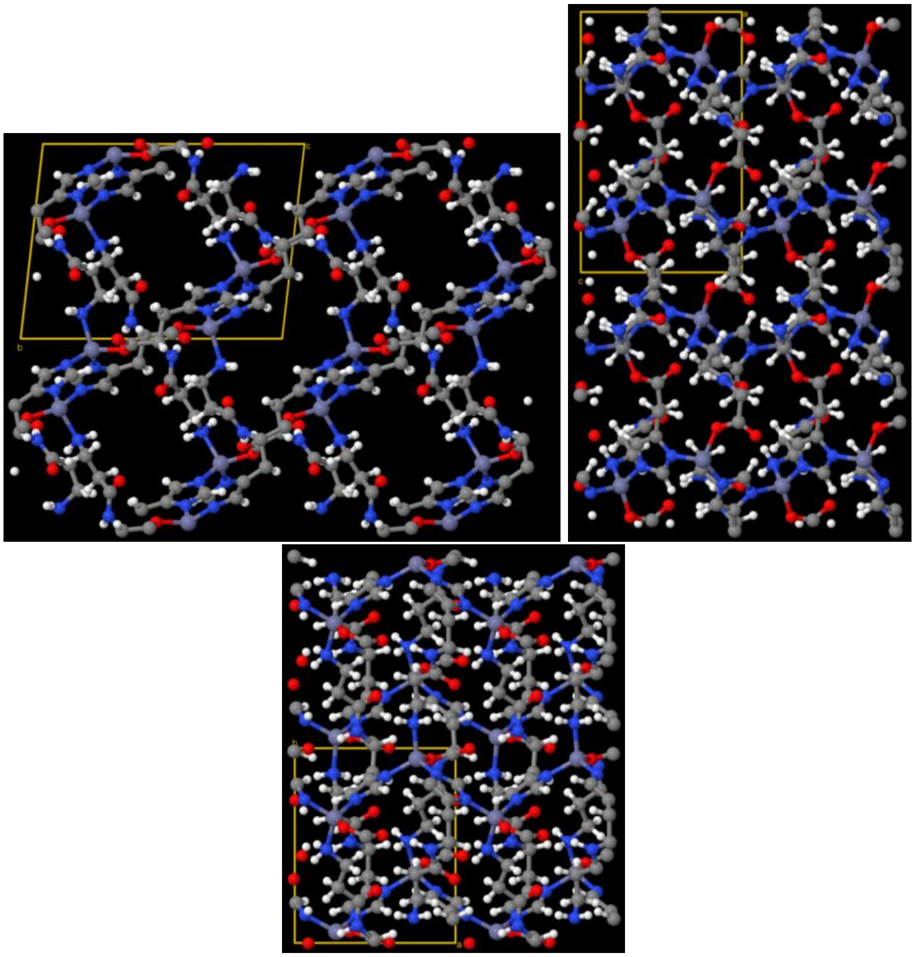

Figure S41: FIPXEJ contains the chemical elements Zn, H, C, N, and O.

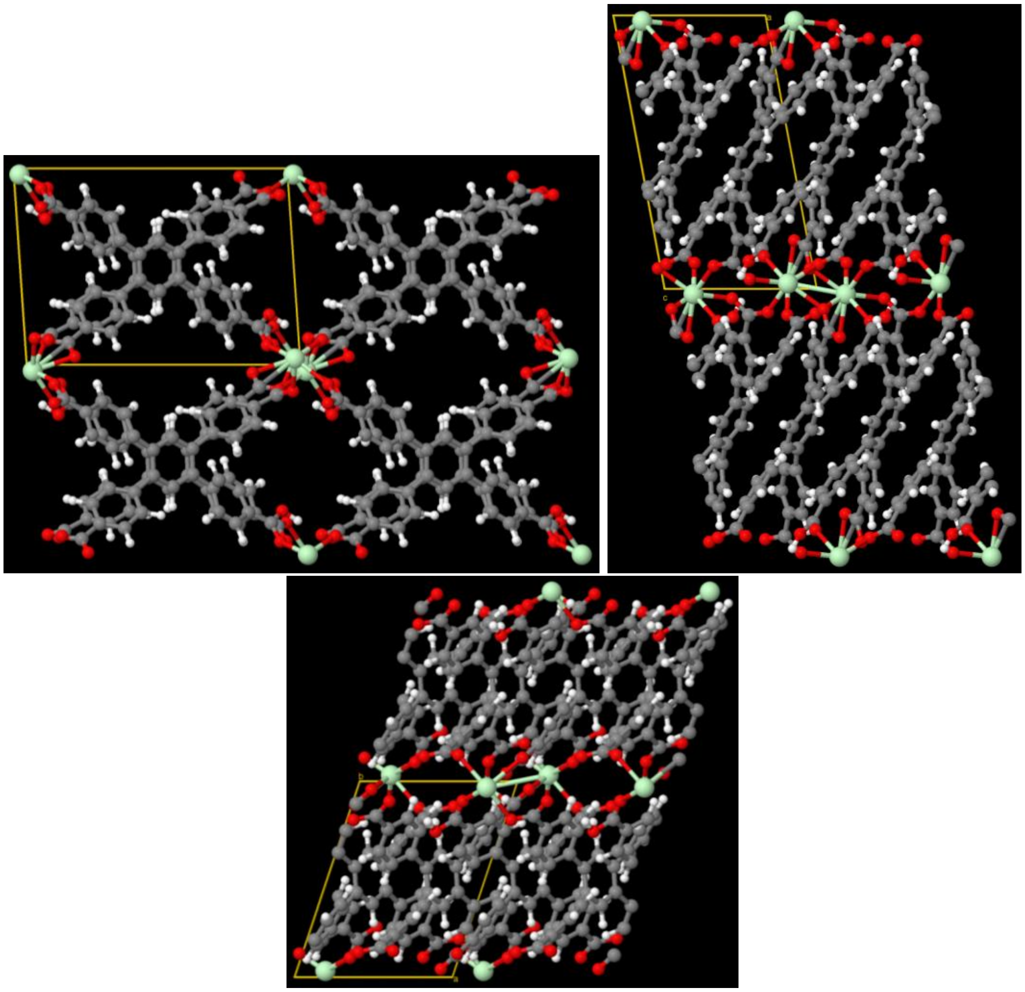

Figure S42: FOCYUT contains the chemical elements Nd, O, H, and C.

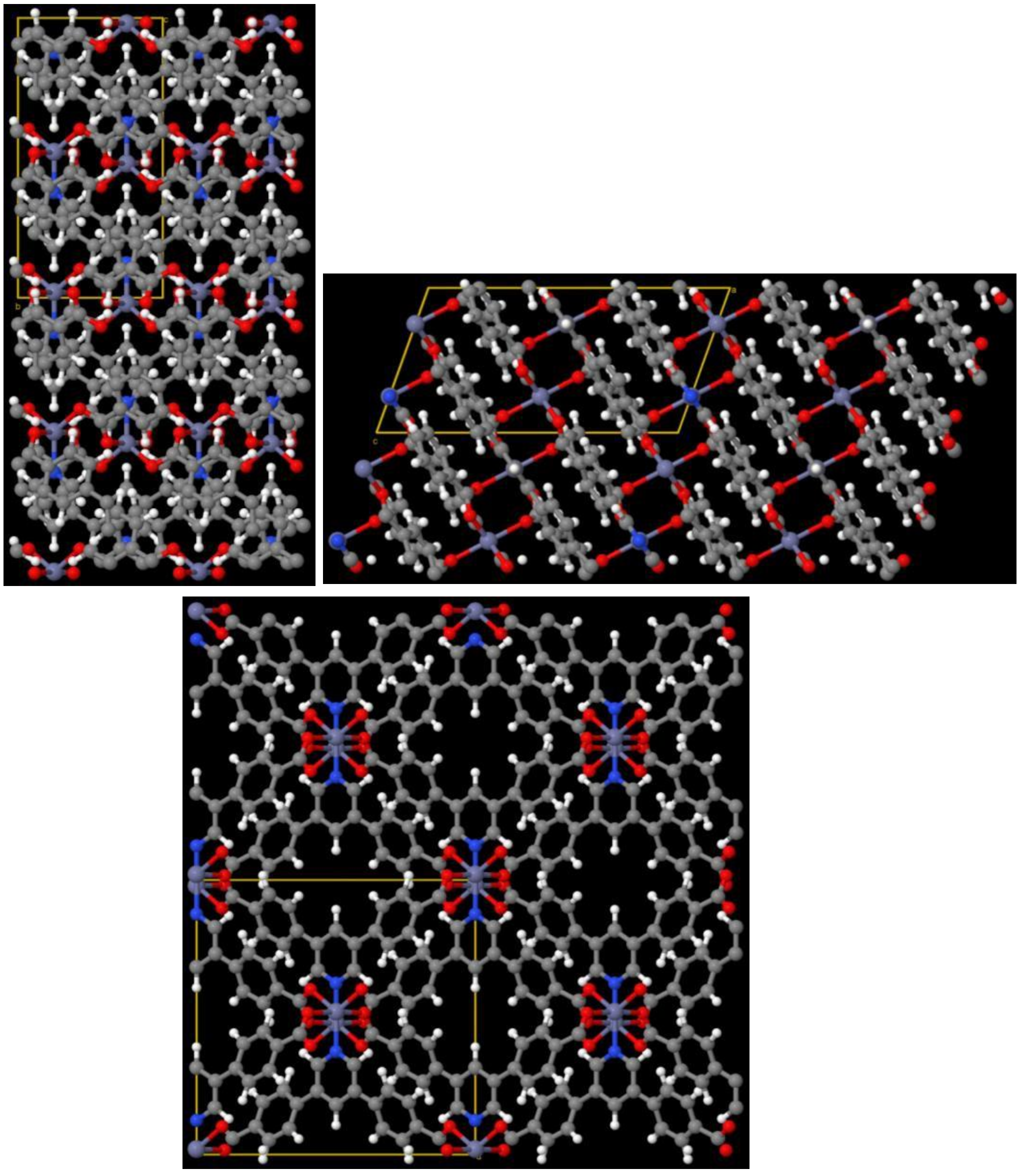

Figure S43: FUBNOH contains the chemical elements C, H, O, Zn, and N.

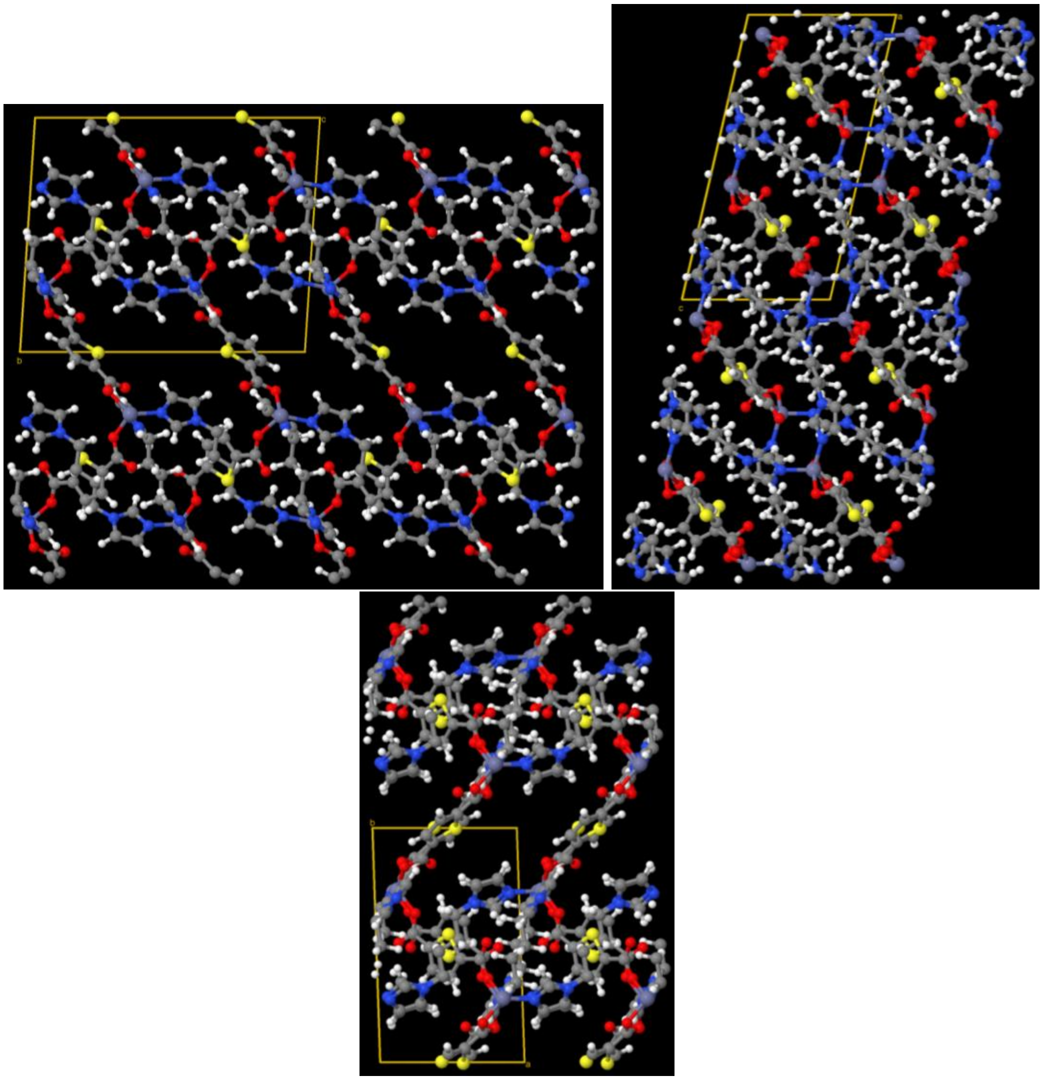

Figure S44: FURFOP contains the chemical elements Zn, C, H, O, N, and S.

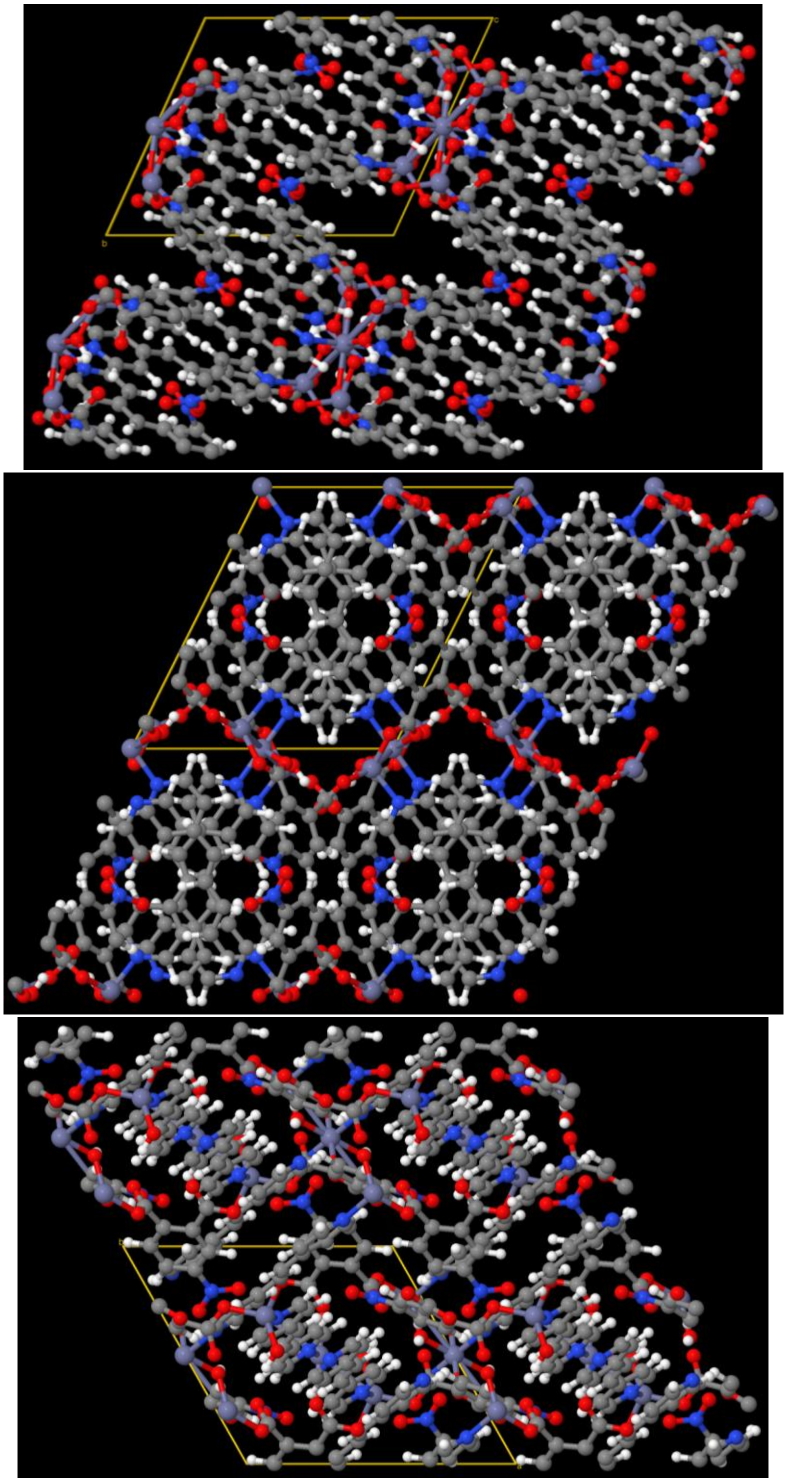

Figure S45: GIRYIR contains the chemical elements Zn, H, C, N, and O.

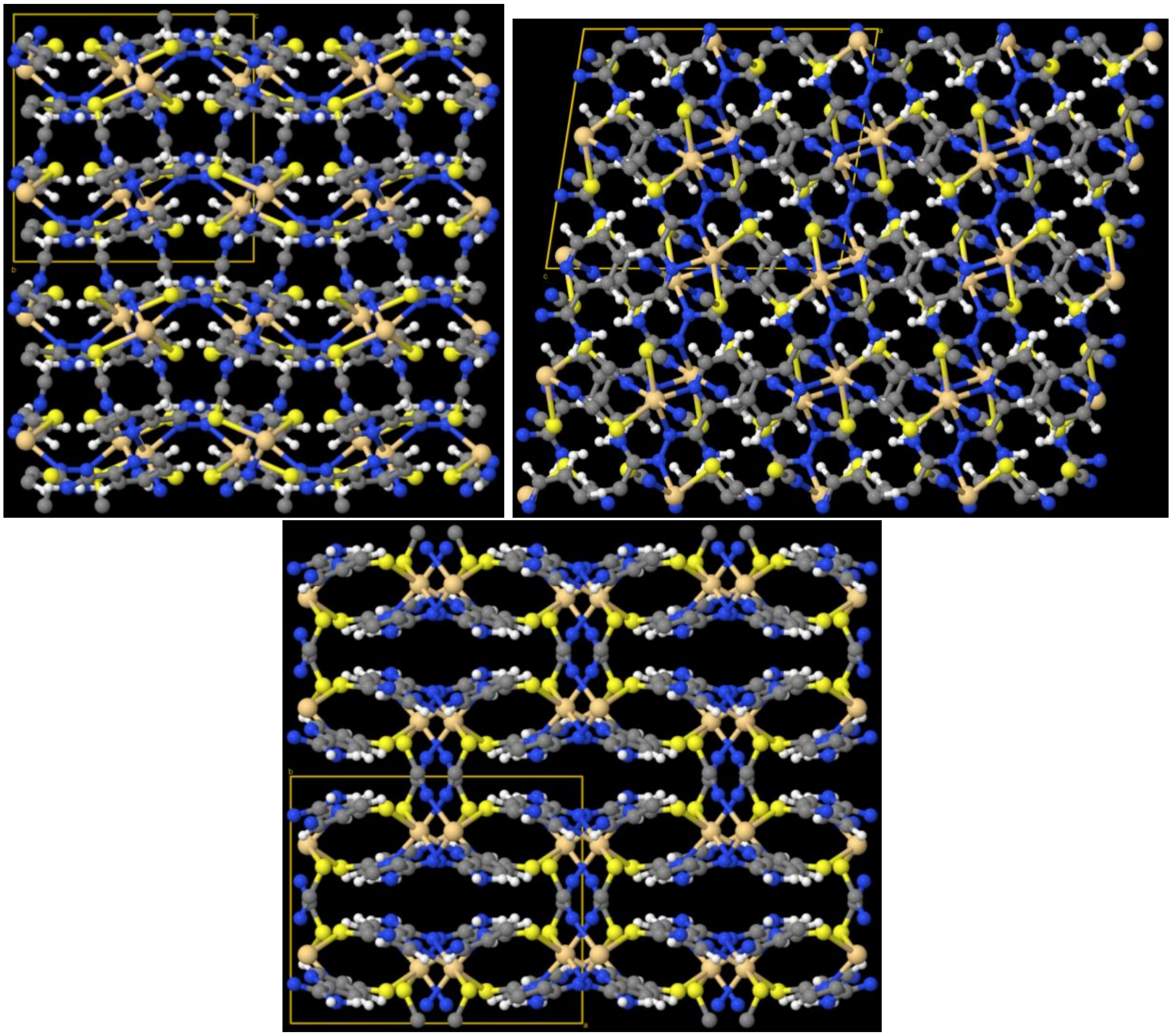

Figure S46: GIWMOP contains the chemical elements Cd, C, H, N, and S.

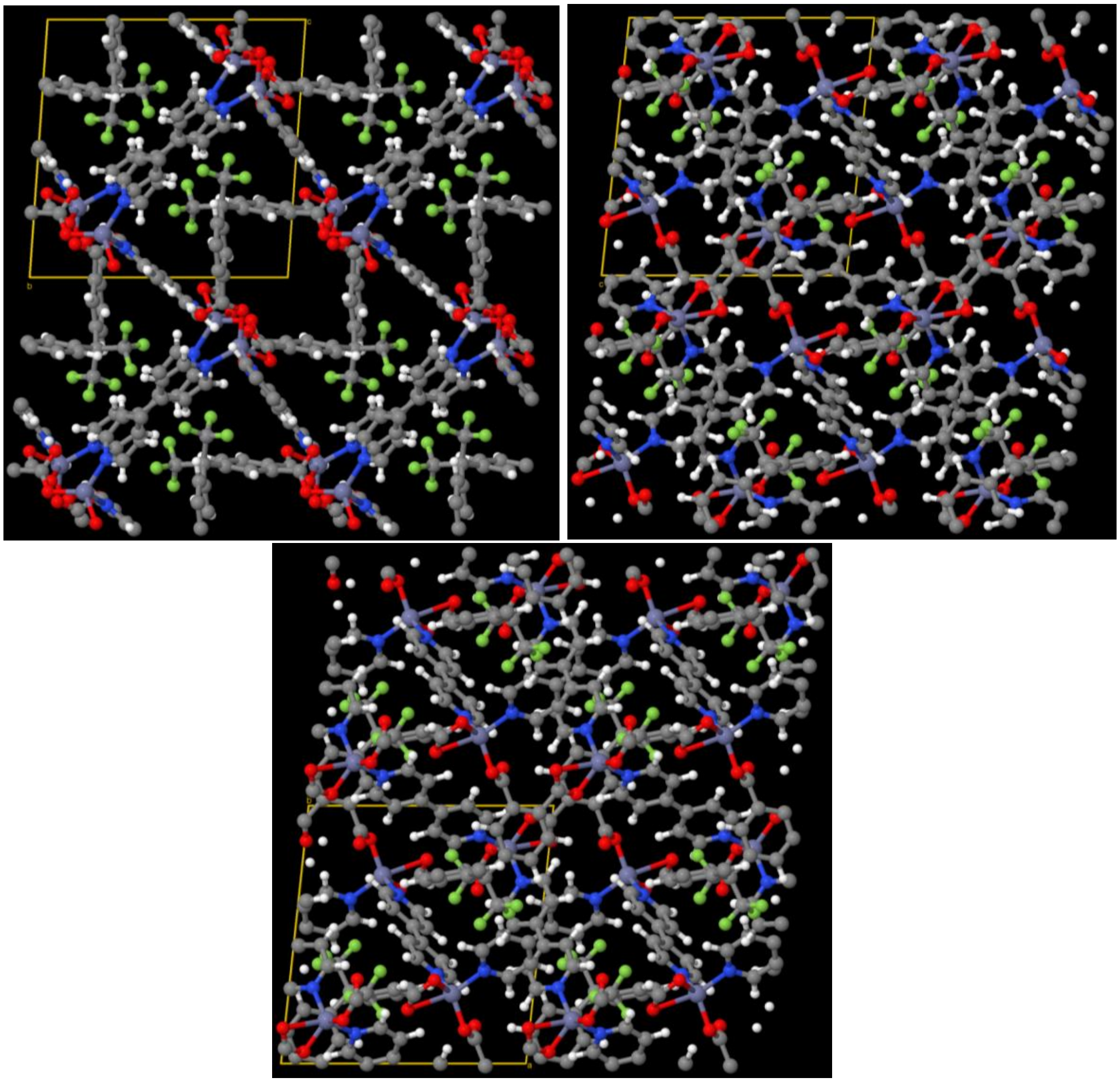

Figure S47: GULPIN contains the chemical elements Zn, H, C, N, O, and F.

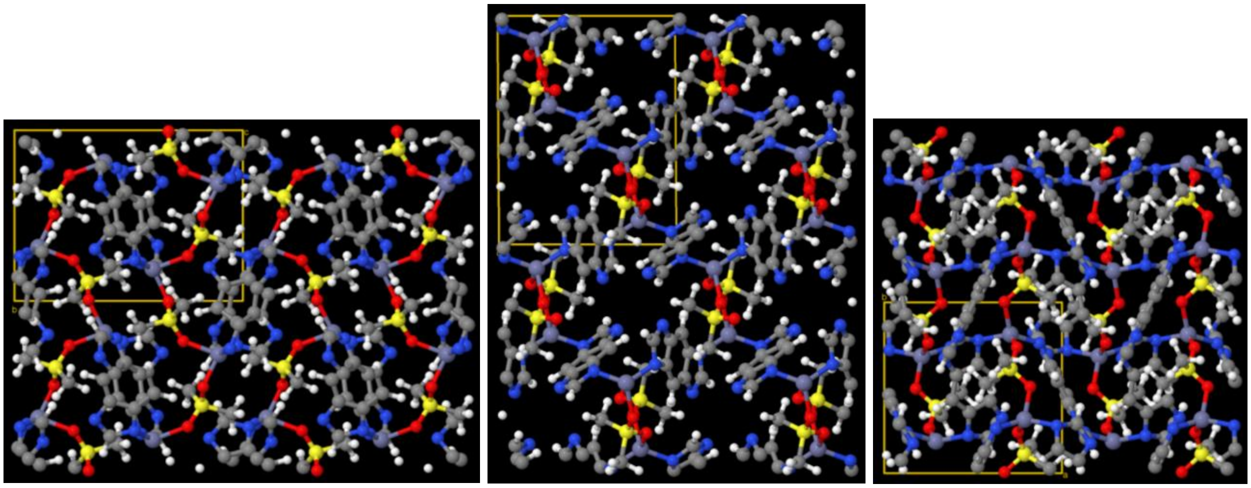

Figure S48: GUMDEZ contains the chemical elements Zn, S, C, H, N, and O.

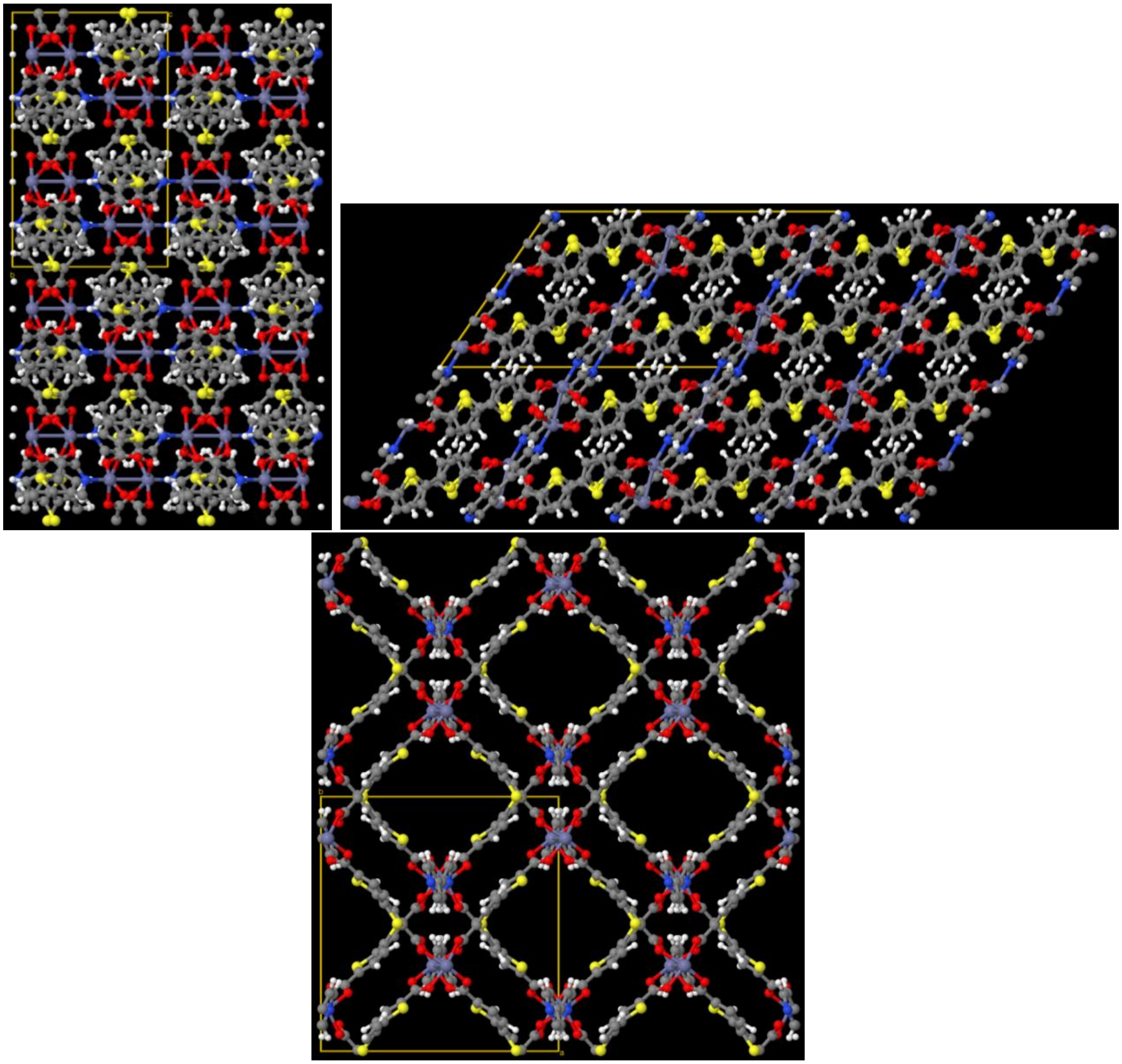

Figure S49: GUYLOC contains the chemical elements Zn, O, C, H, N, and S.

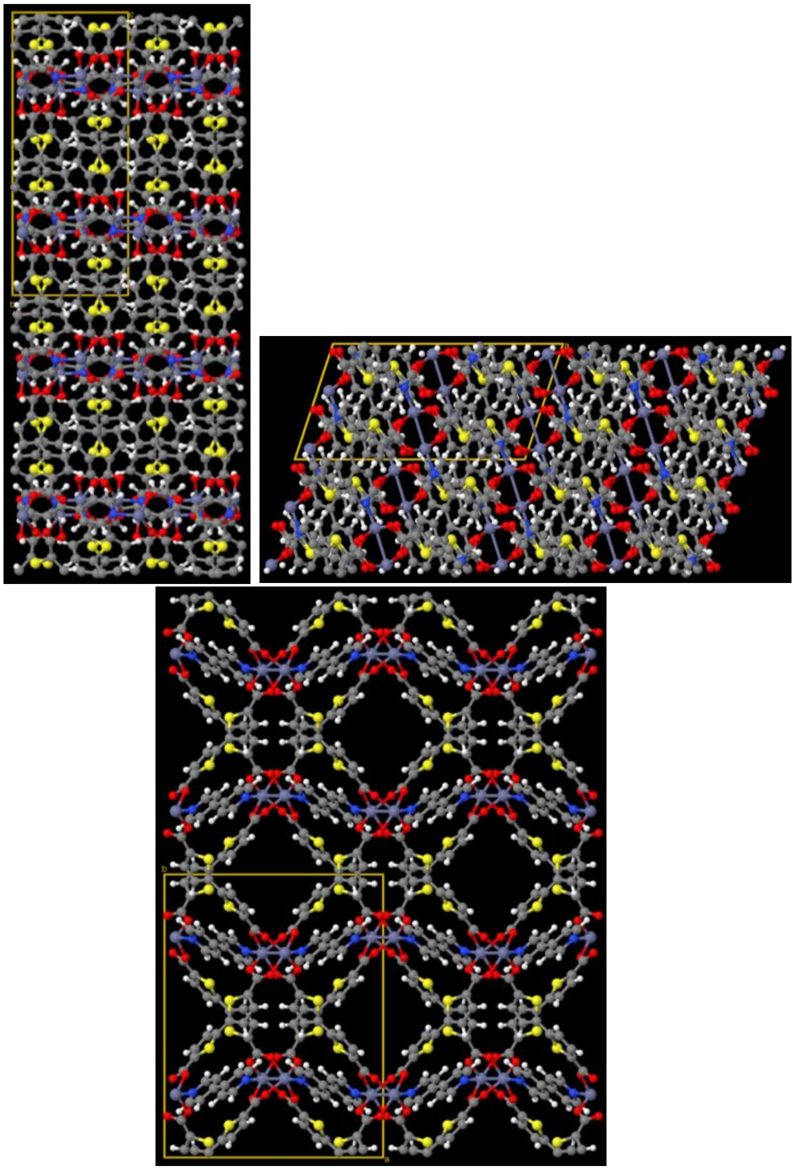

Figure S50: GUYLUI contains the chemical elements O, C, S, H, Zn, and N.

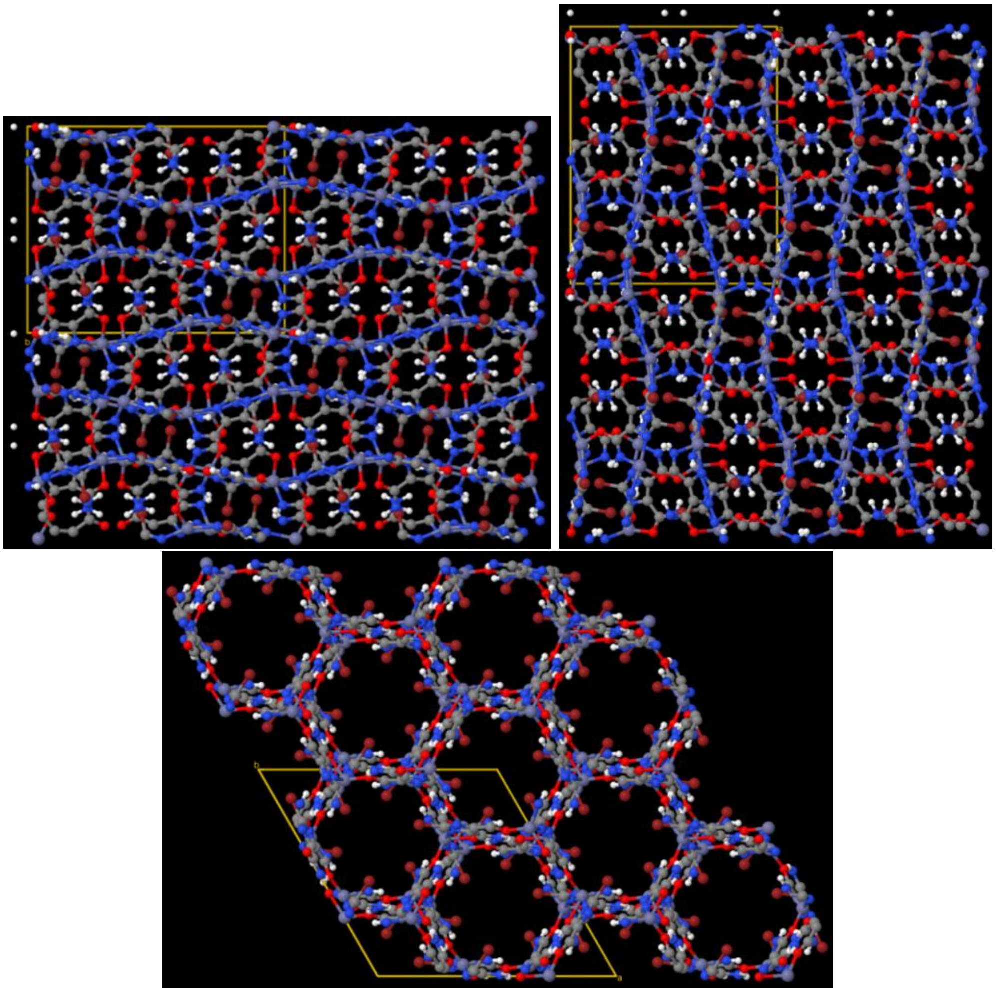

Figure S51: HEBZAR contains the chemical elements C, Br, N, Zn, H, and O.

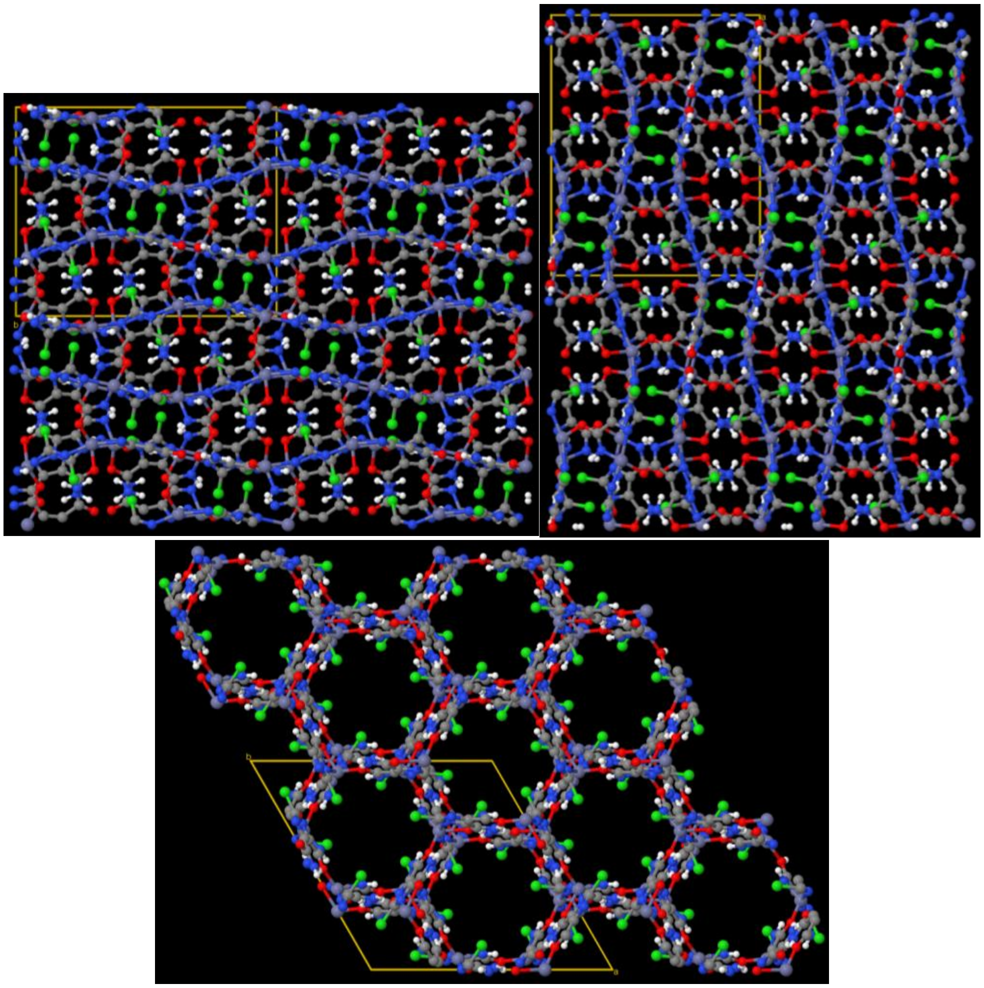

Figure S52: HEBZEV contains the chemical elements C, Cl, N, Zn, H, and O.

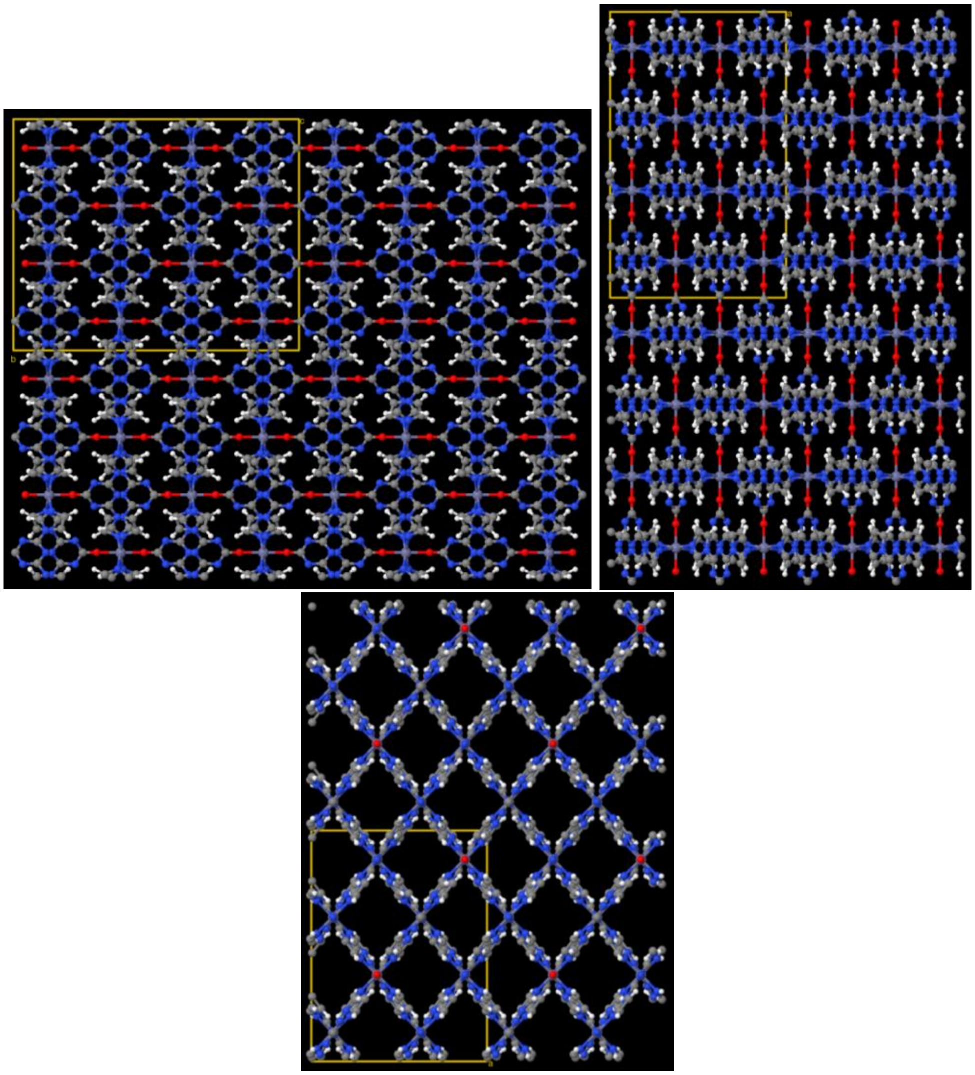

Figure S53: HECQUB contains the chemical elements N, C, H, Zn, and O.

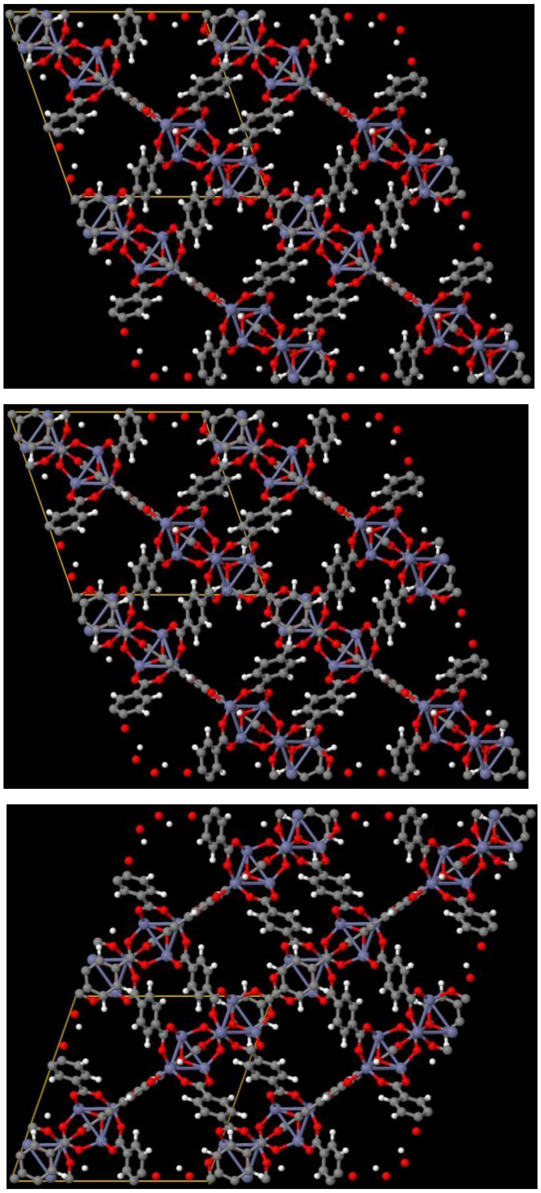

Figure S54: HIFTOG02 contains the chemical elements Zn, H, C, and O.

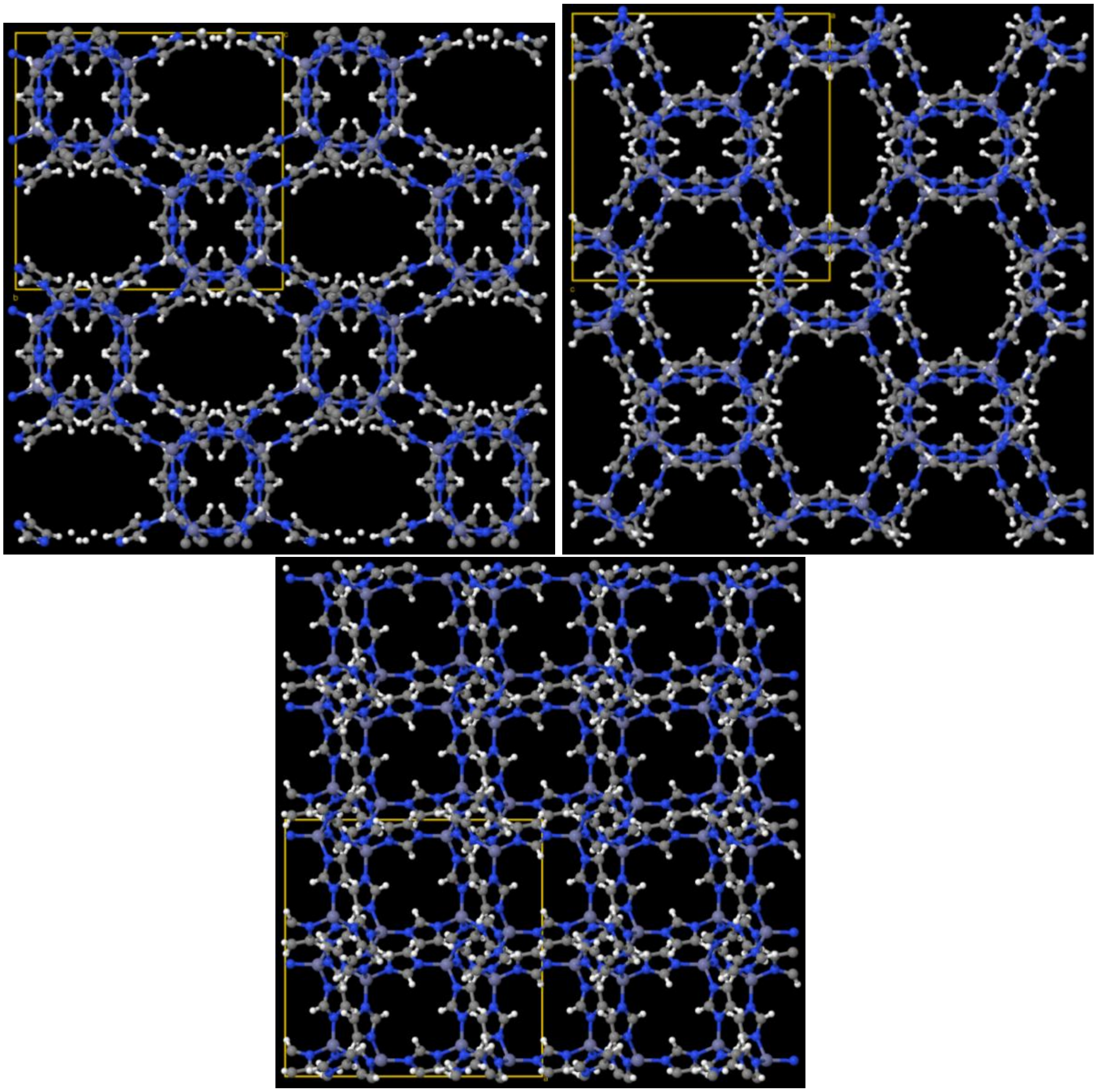

Figure S55: HIFVUO contains the chemical elements Zn, N, C, and H.

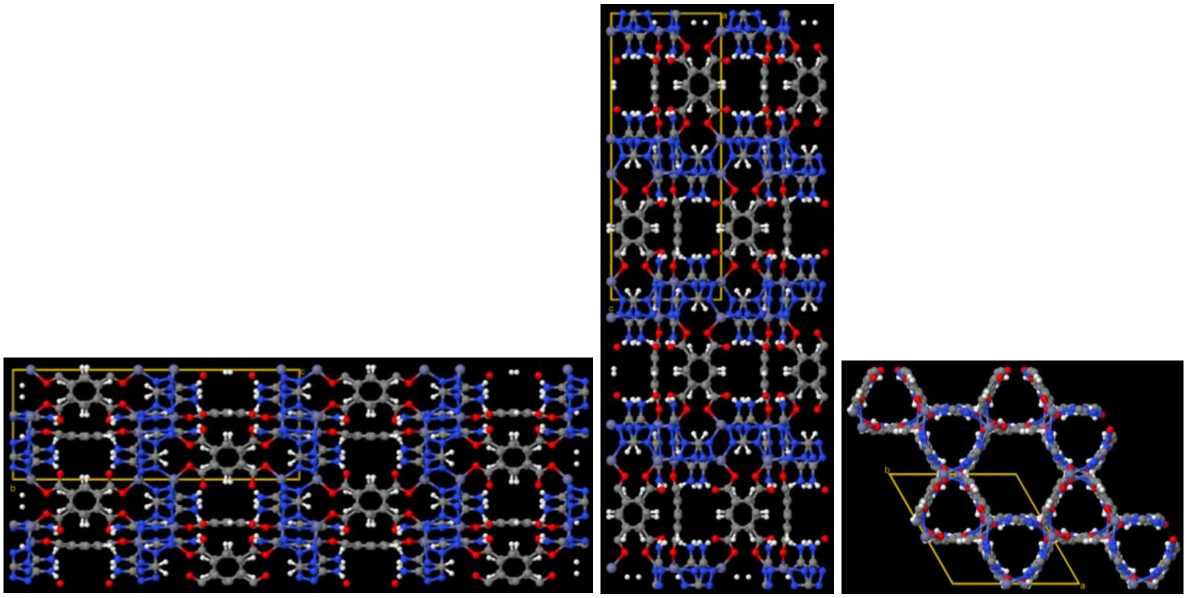

Figure S56: HIHJUF contains the chemical elements O, N, C, H, and Zn.

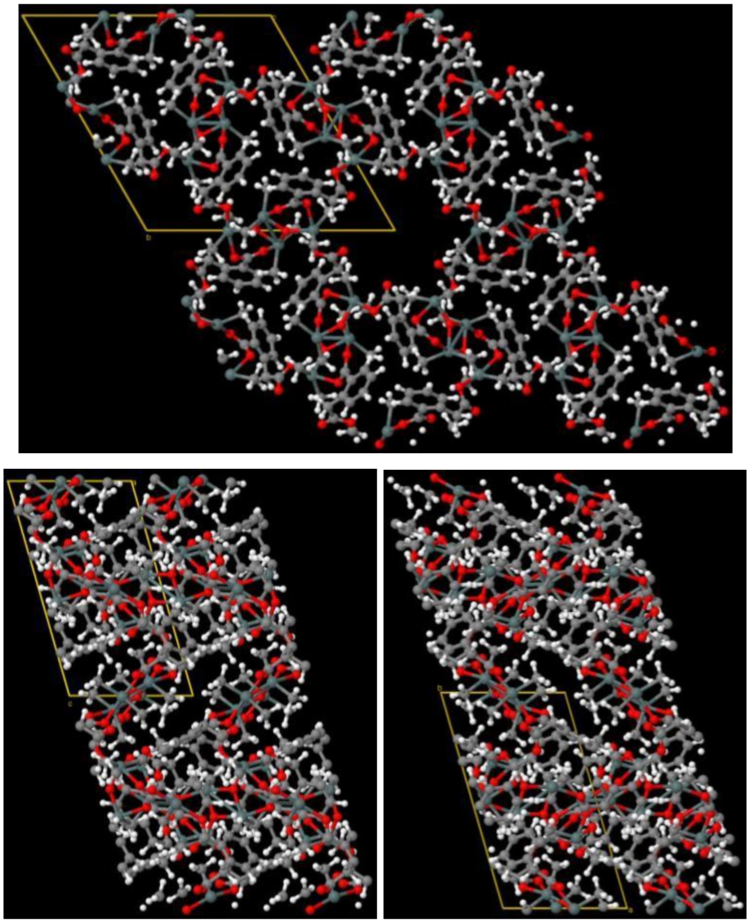

Figure S57: HOXGEH contains the chemical elements Sn, H, C, and O.

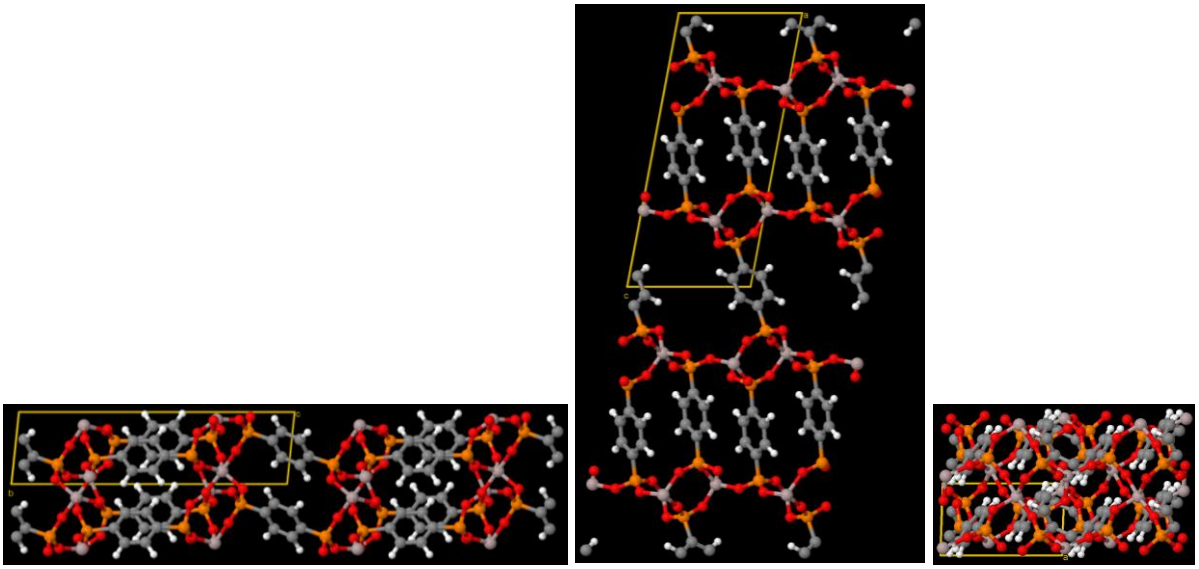

Figure S58: HOZDOR contains the chemical elements Al, P, O, C, and H.

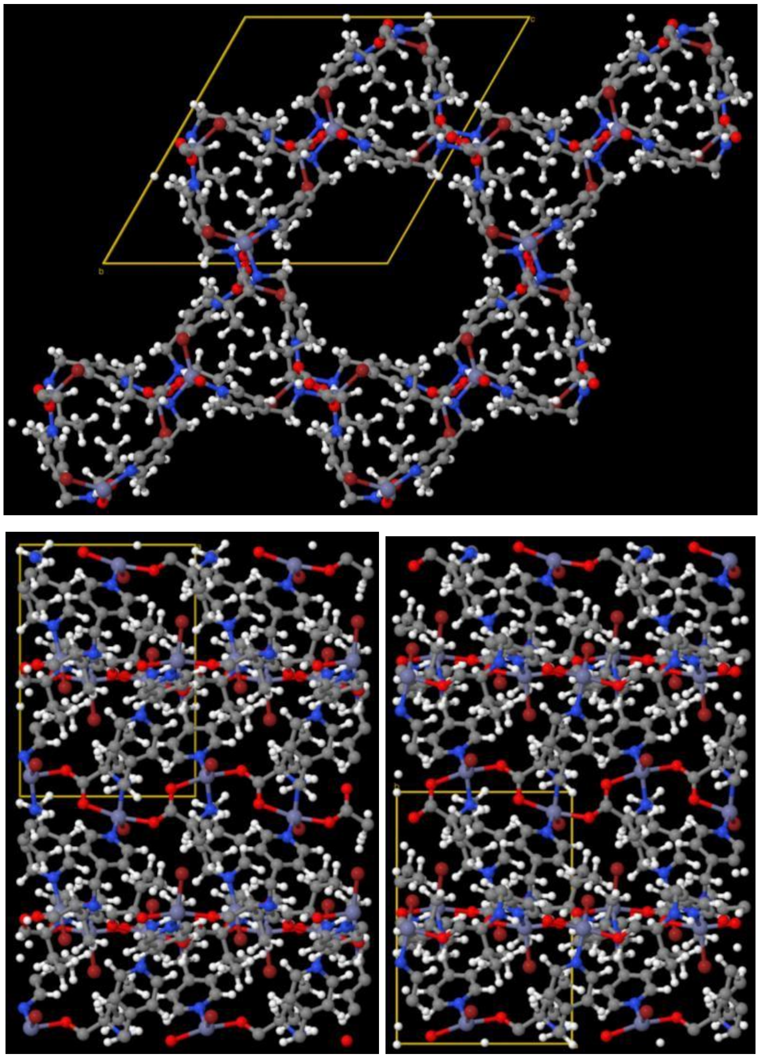

Figure S59: IBICED contains the chemical elements Zn, H, C, Br, N, and O.

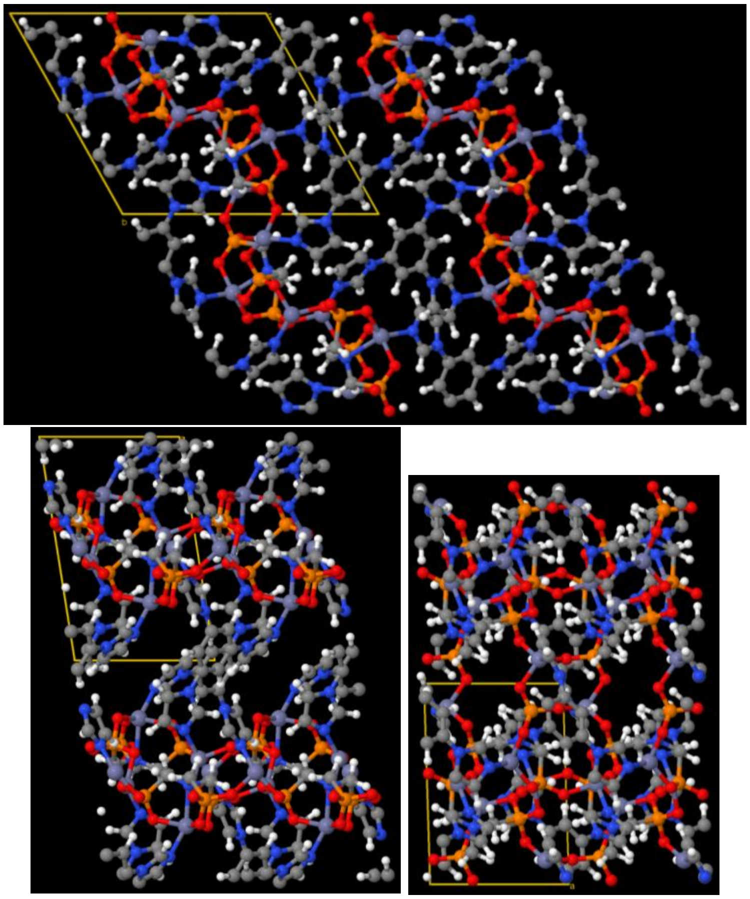

Figure S60: IBOCIO contains the chemical elements H, C, O, N, Zn, and P.

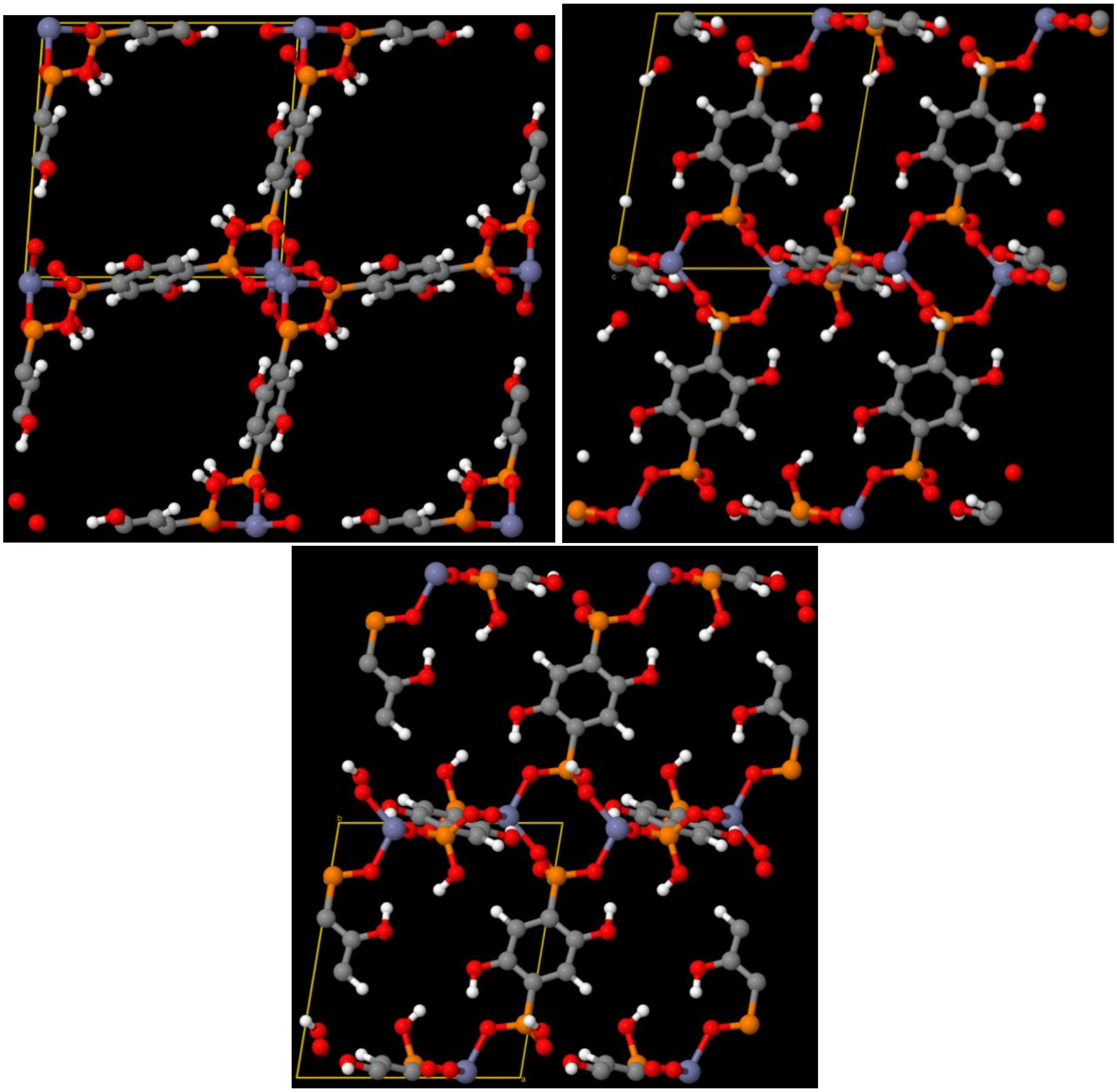

Figure S61: JIVFUQ contains the chemical elements Zn, P, H, C, and O.

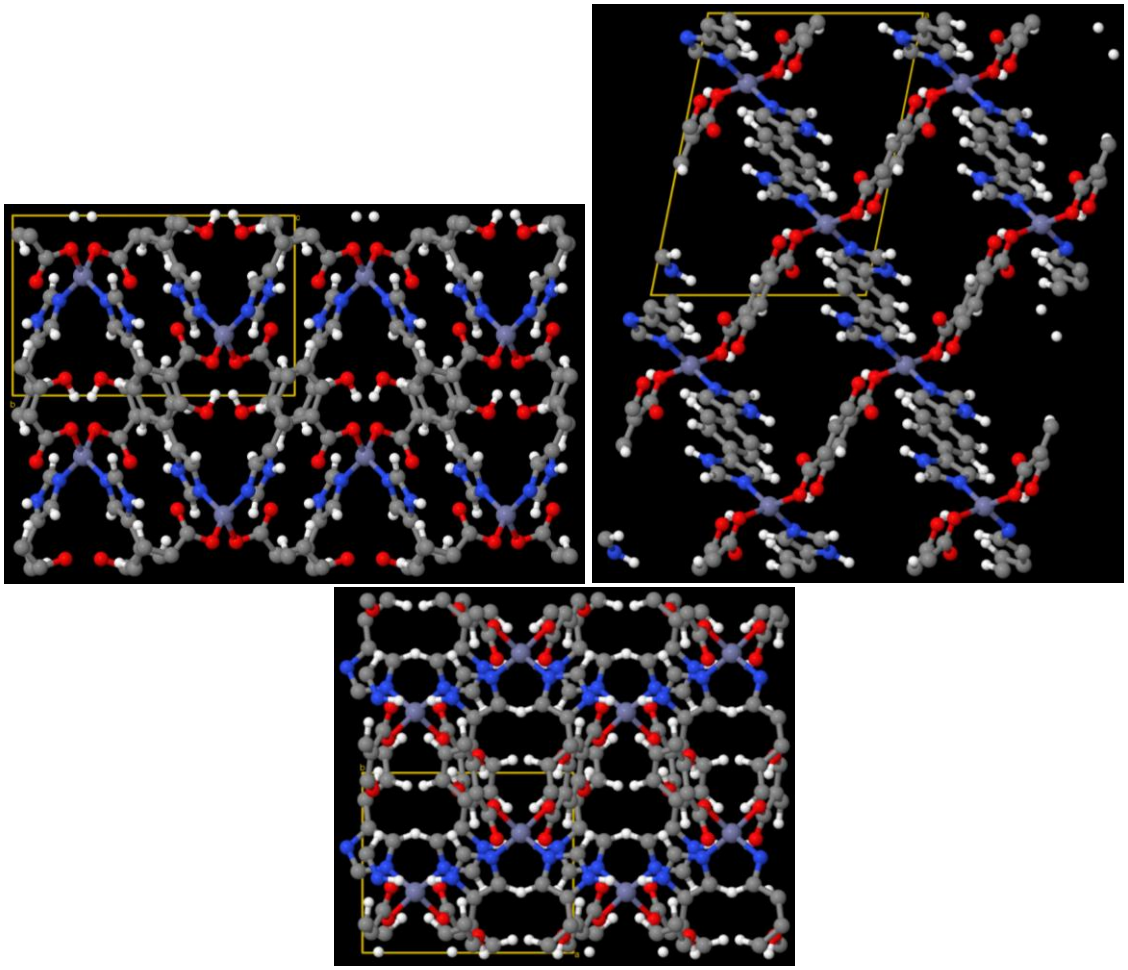

Figure S62: KACZUM contains the chemical elements O, C, H, Zn, and N.

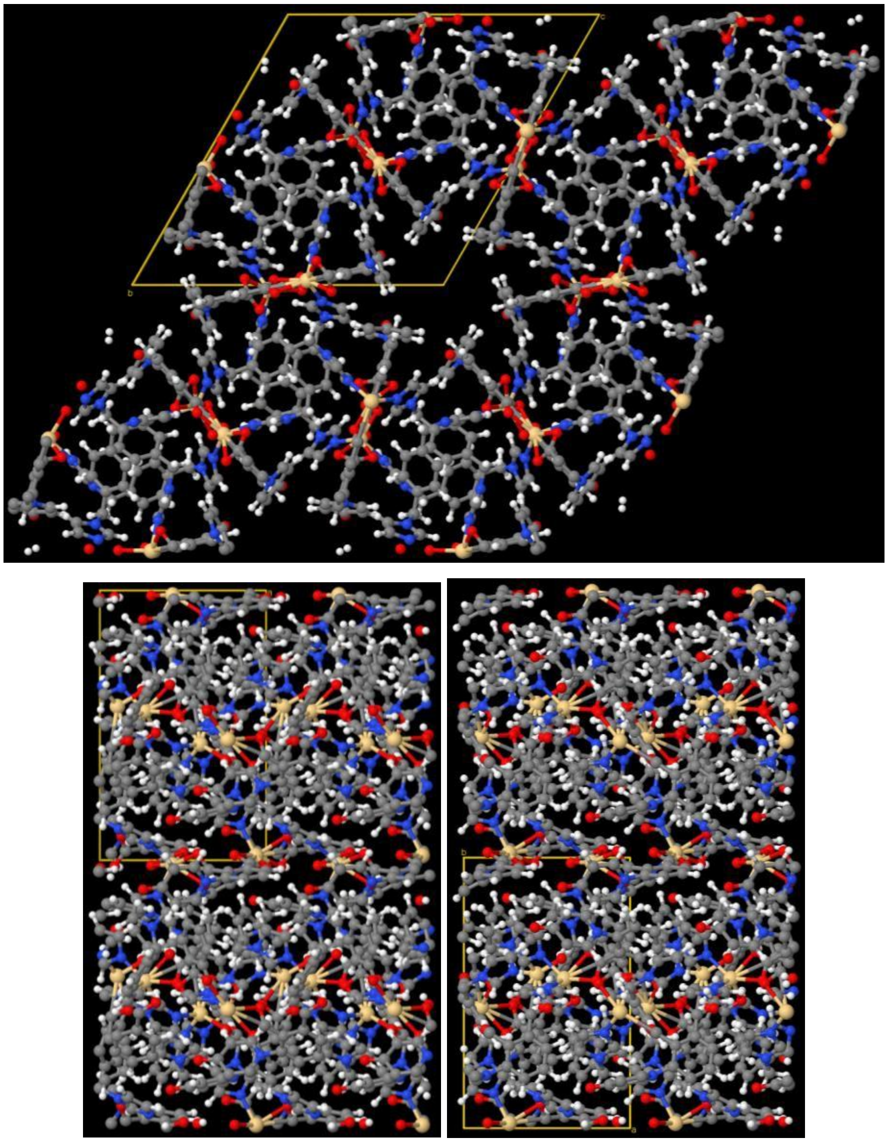

Figure S63: KATDAM contains the chemical elements Cd, H, C, N, and O.

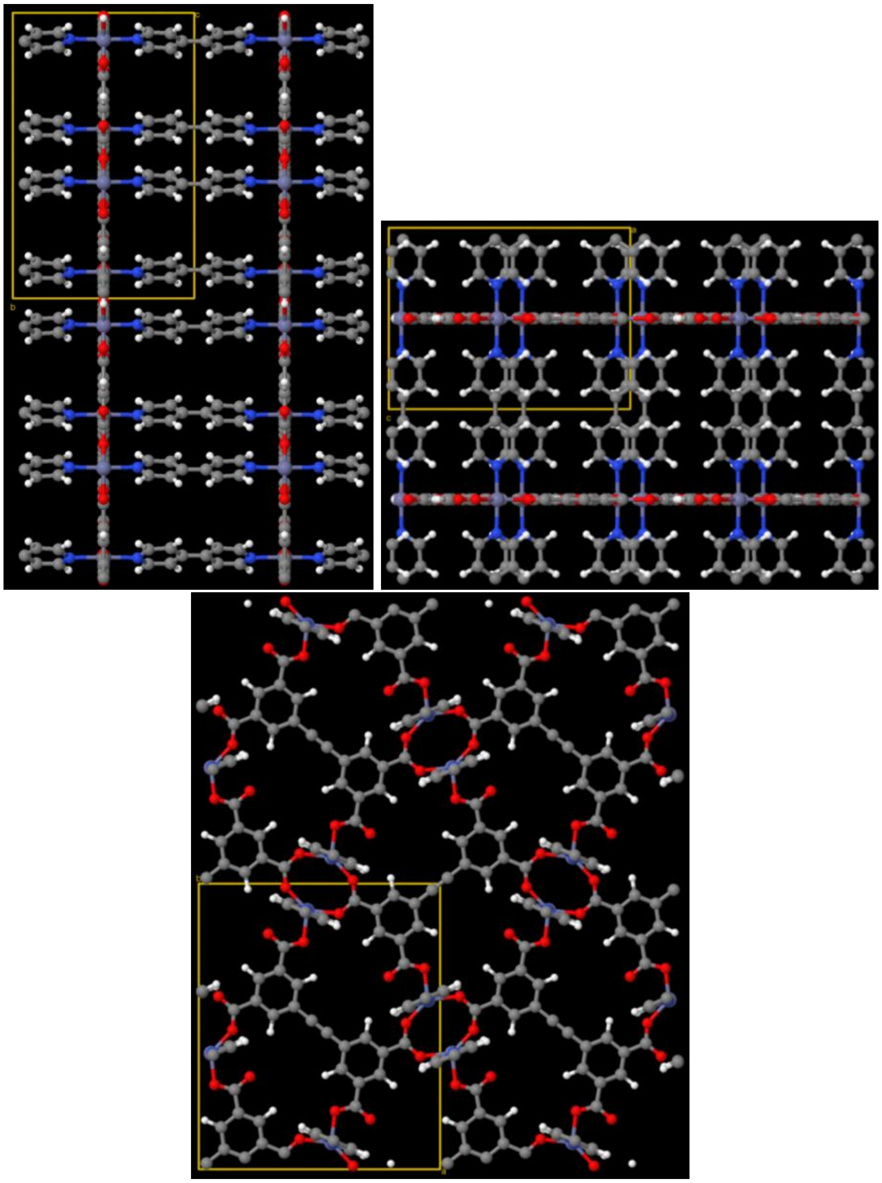

Figure S64: KEWZOD contains the chemical elements O, C, H, Zn, and N.

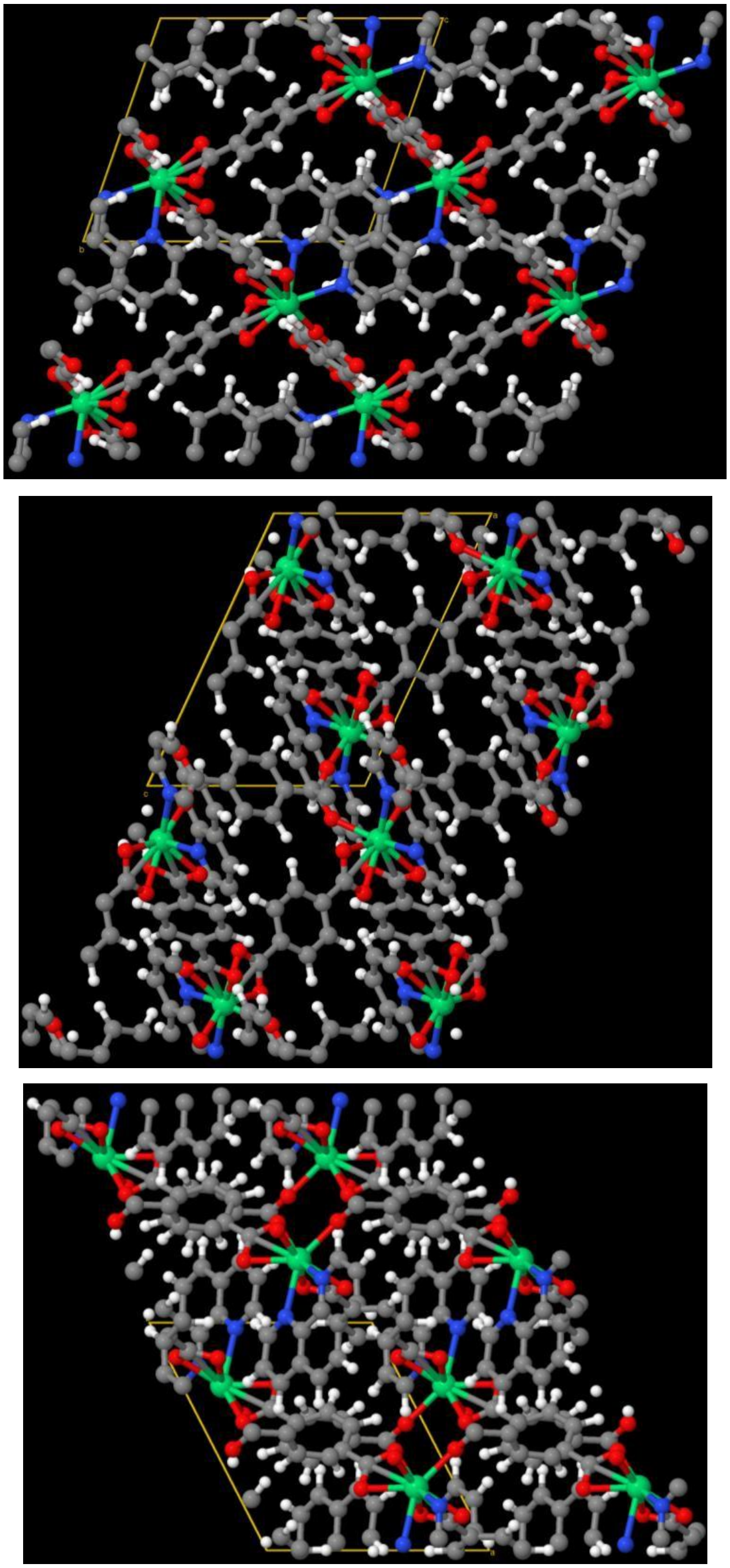

Figure S65: KINDIW contains the chemical elements Er, H, C, N, and O.

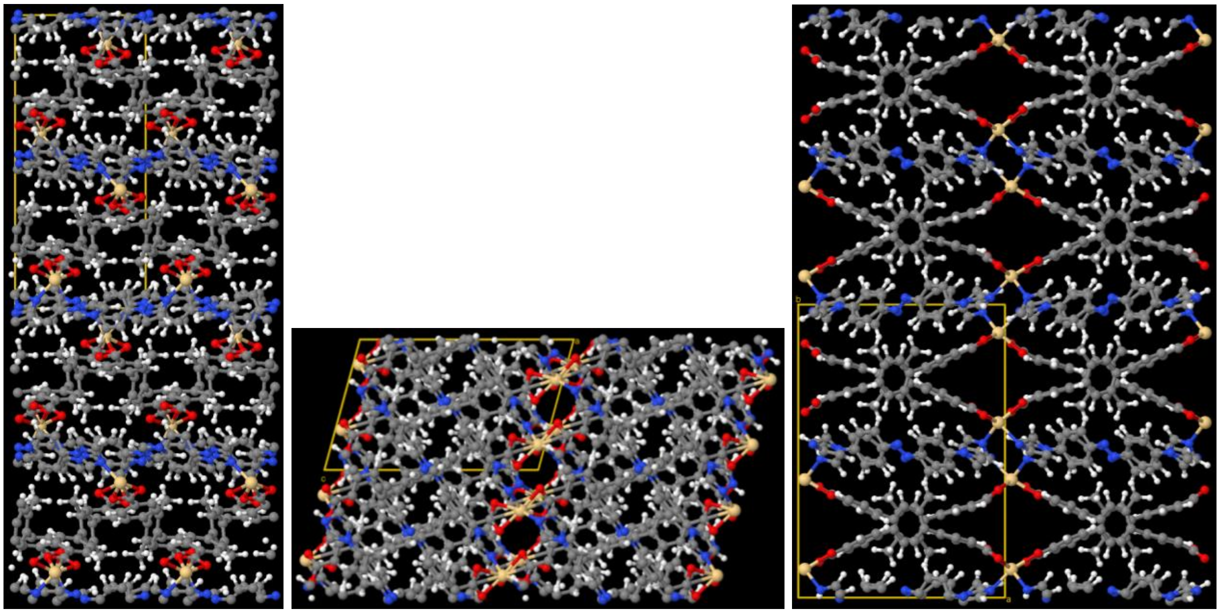

Figure S66: KINJUO contains the chemical elements C, H, N, Cd, and O.

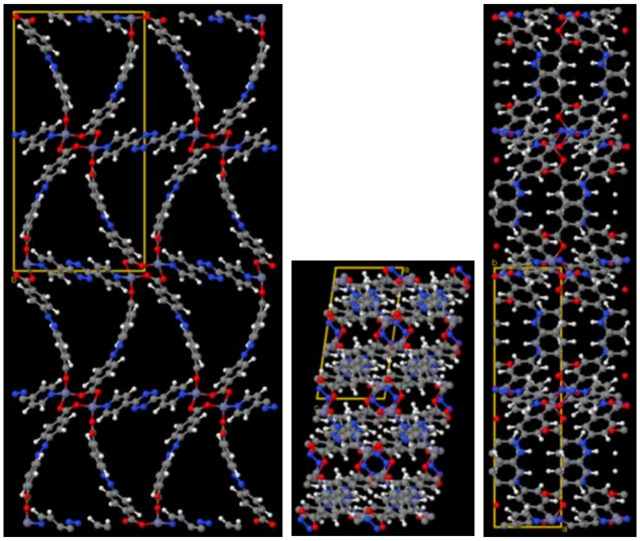

Figure S67: KURSOH contains the chemical elements Zn, O, C, H, and N.

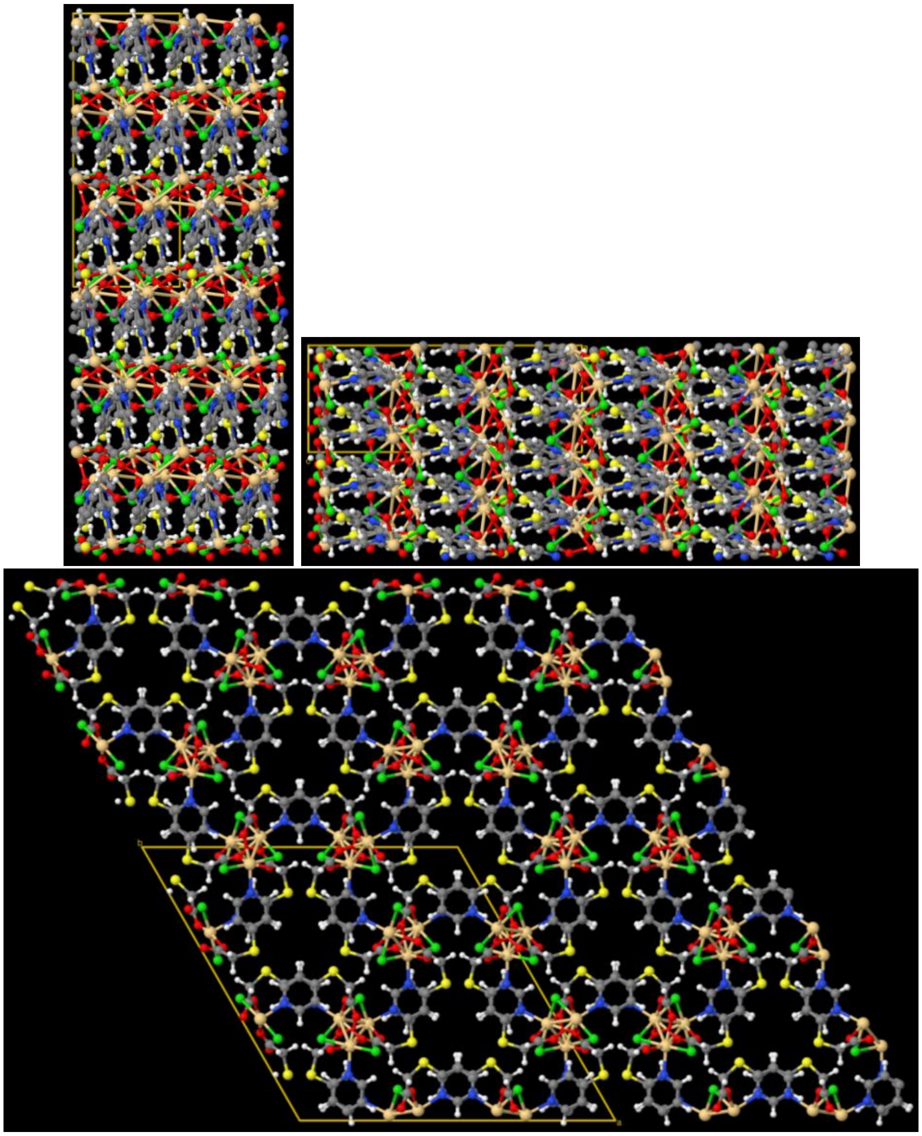

Figure S68: LALYOO contains the chemical elements Cd, Cl, O, S, N, C, and H.

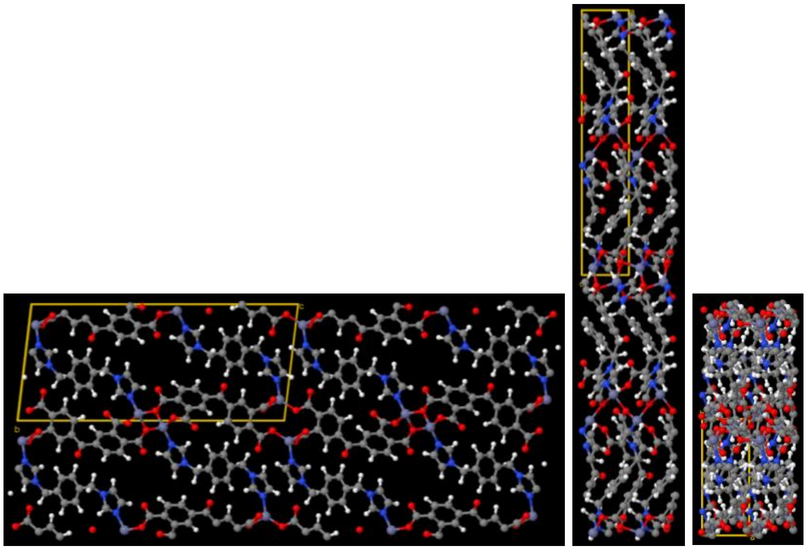

Figure S69: LIWXIZ contains the chemical elements Zn, H, C, N, and O.

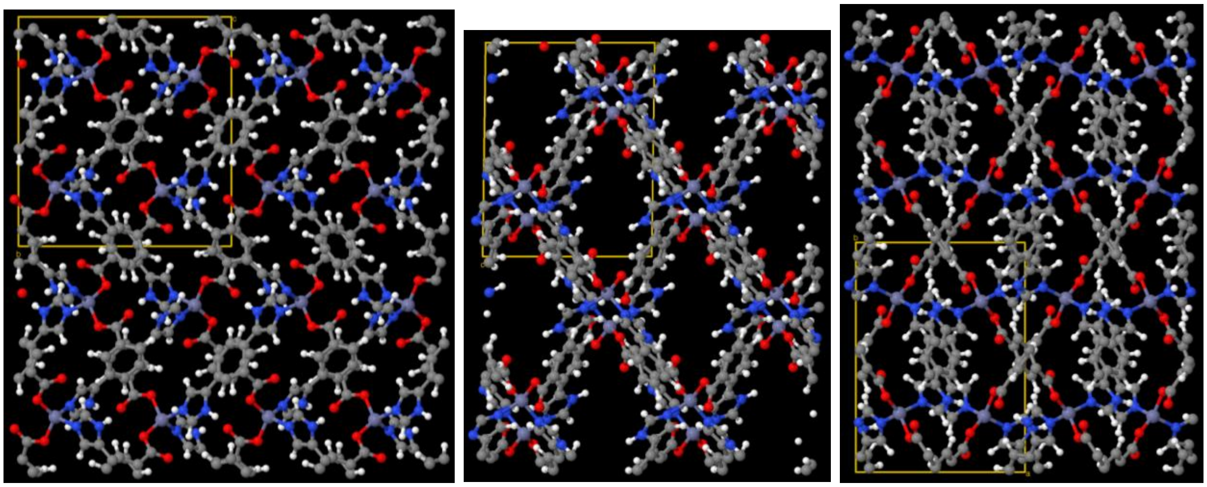

Figure S70: LODPOL contains the chemical elements N, H, C, O, and Zn.

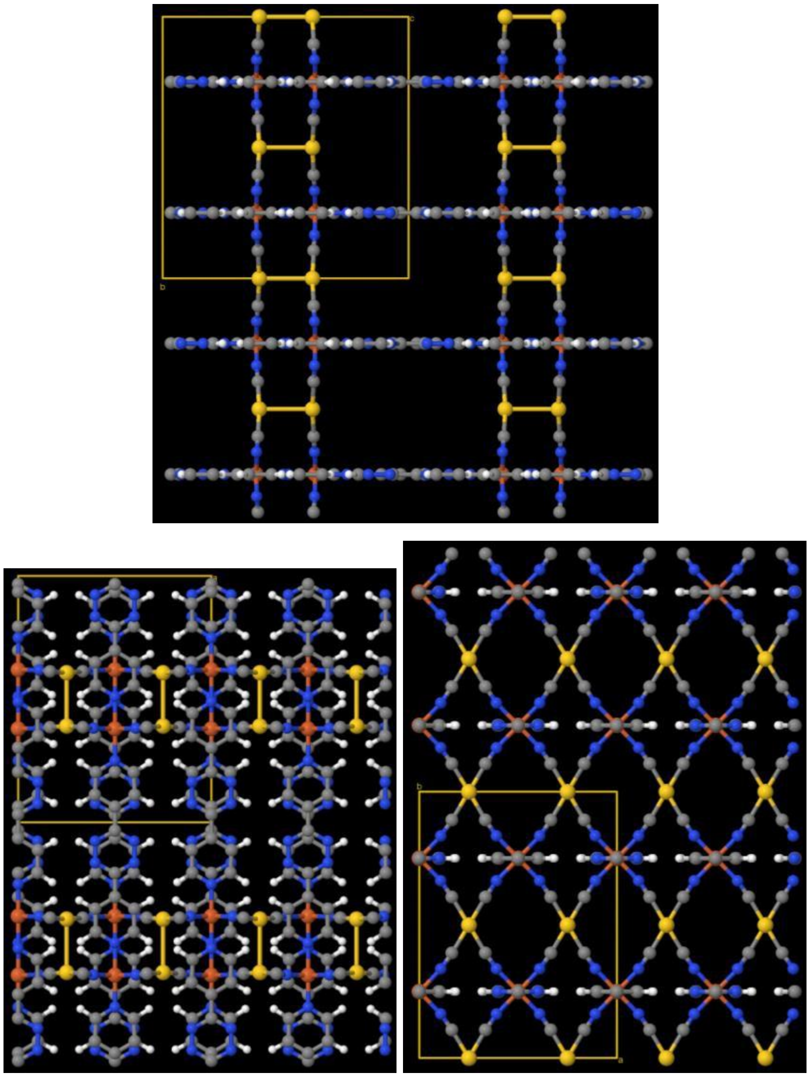

Figure S71: LOJLAZ contains the chemical elements N, C, Au, Fe, and H.

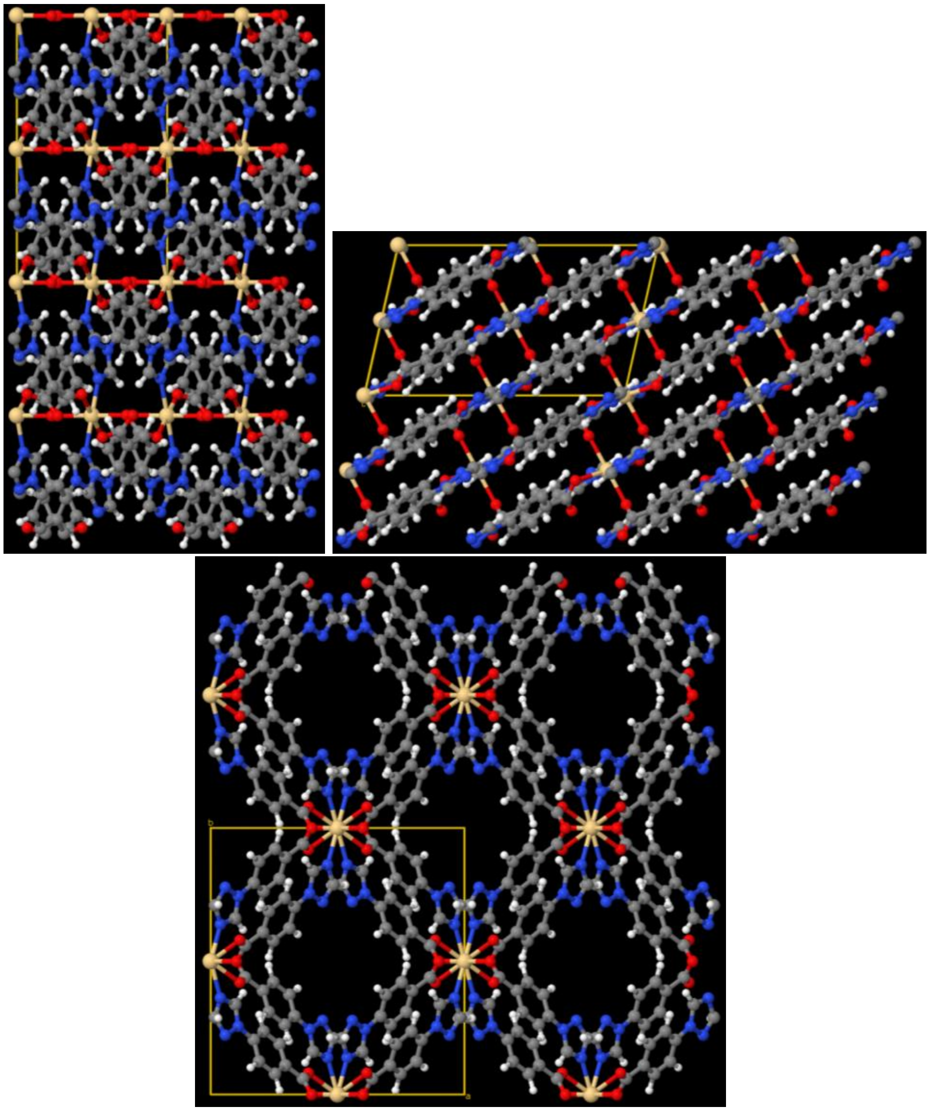

Figure S72: LUFCAS contains the chemical elements O, N, C, H, and Cd.

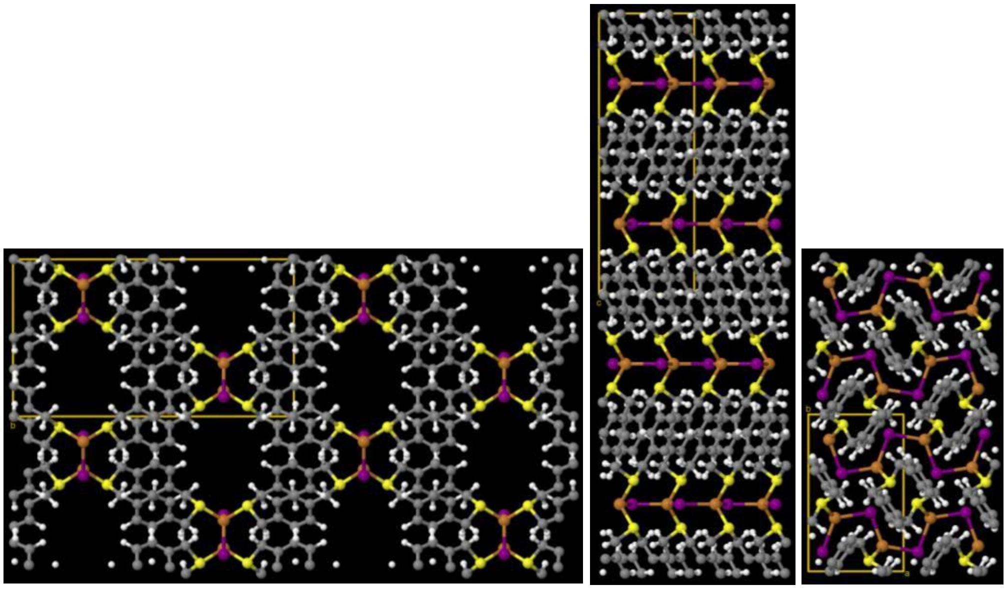

Figure S73: MADVUJ contains the chemical elements Cu, H, C, S, and I.

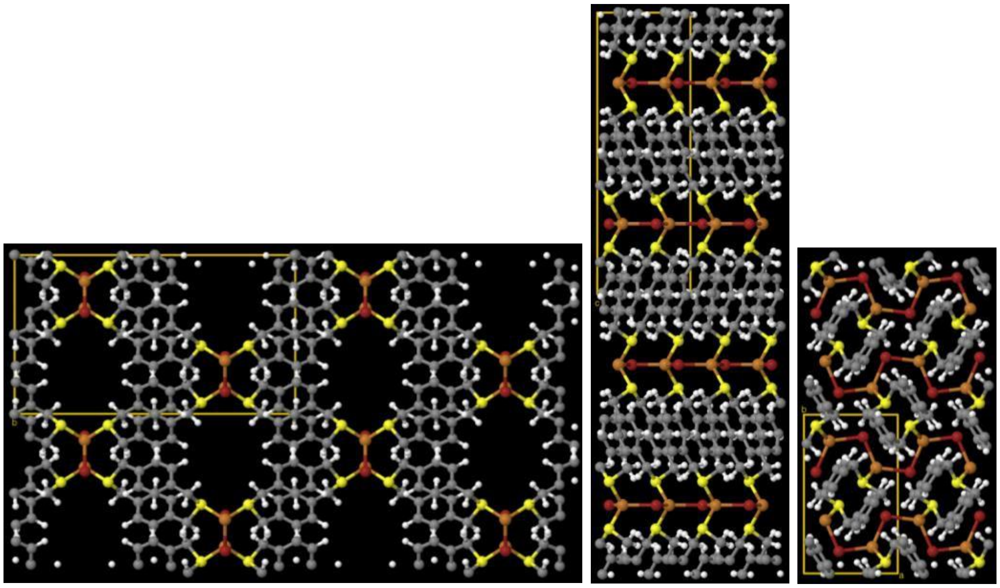

Figure S74: MADWAQ contains the chemical elements Cu, H, C, S, and Br.

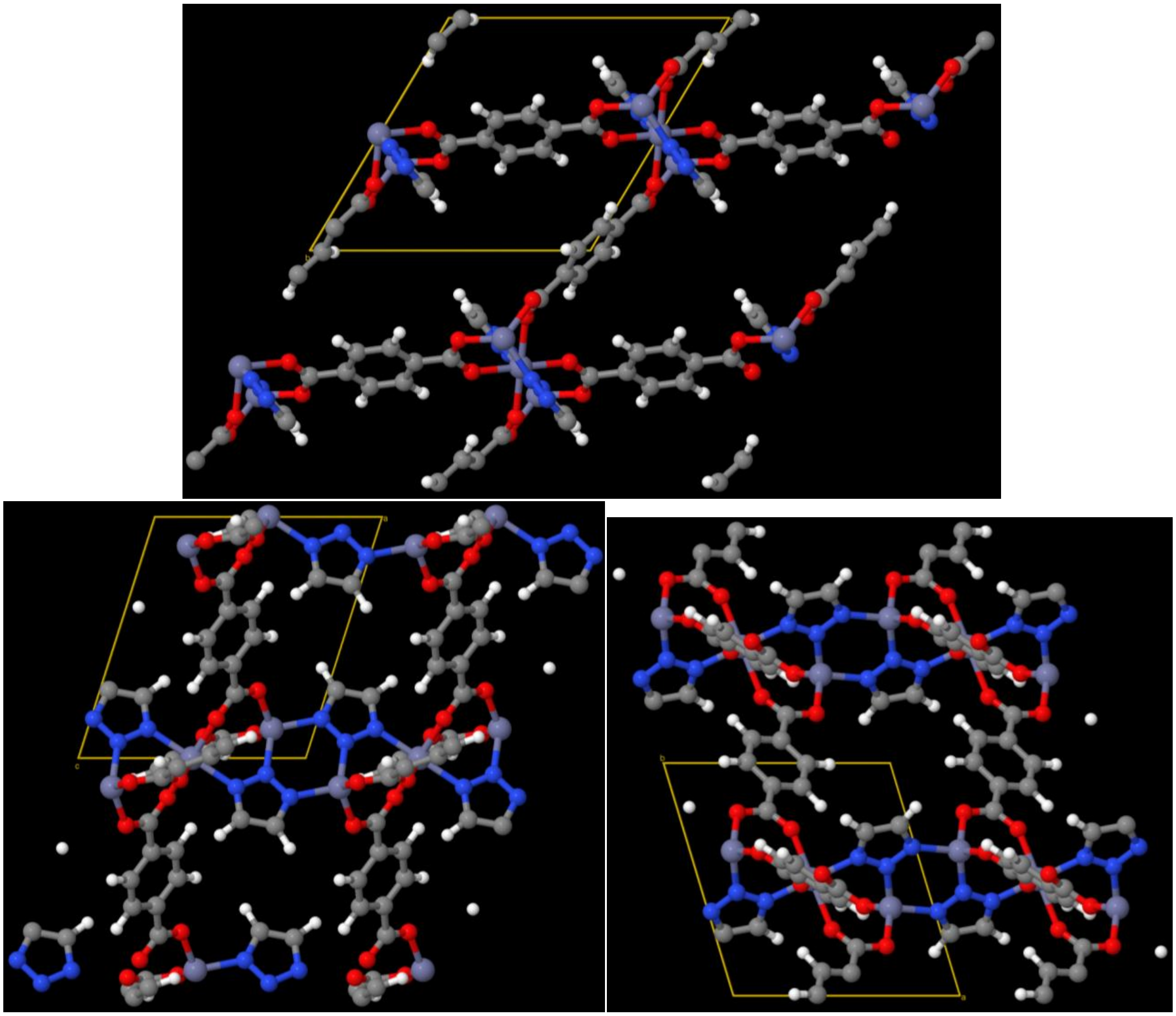

Figure S75: MAZSUD contains the chemical elements Zn, H, C, N, and O.

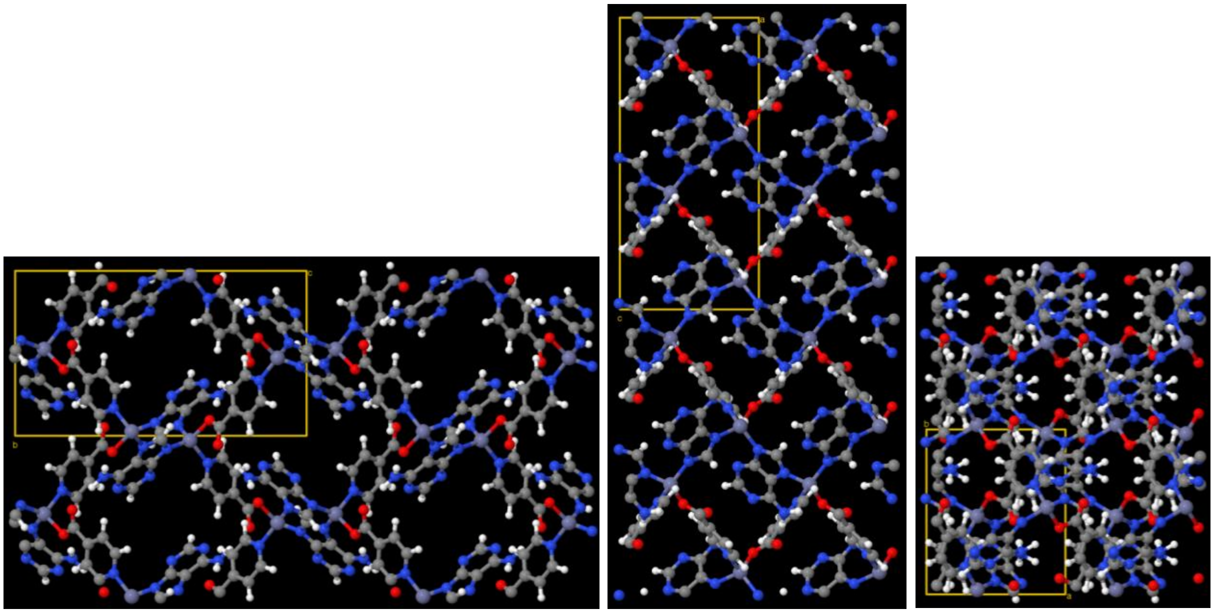

Figure S76: MEJQID contains the chemical elements Zn, H, C, N, and O.

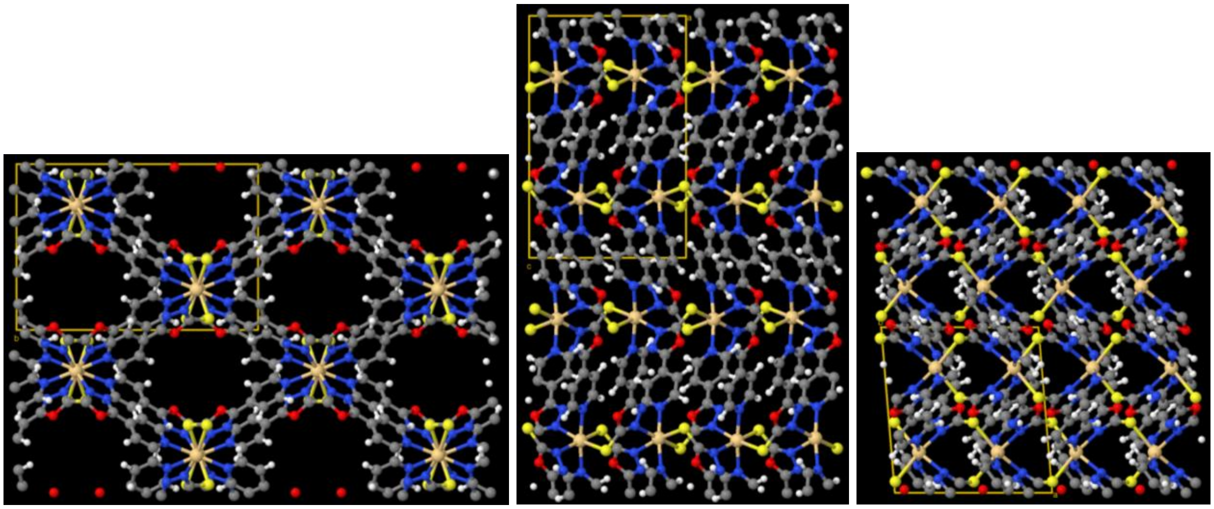

Figure S77: MEKDUC contains the chemical elements Cd, H, C, S, N, and O.

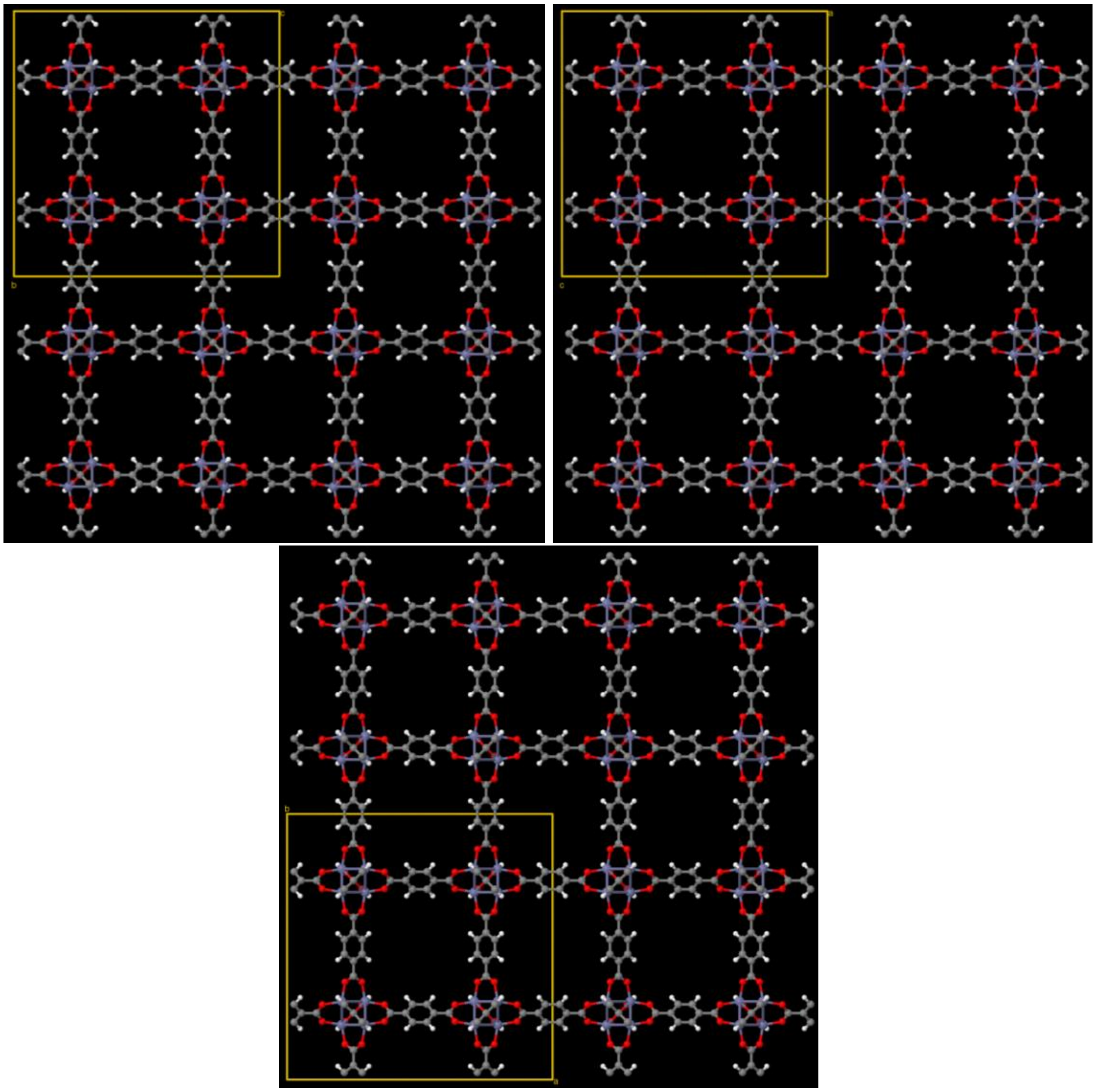

Figure S78: MIBQAR01 contains the chemical elements Zn, O, C, and H.

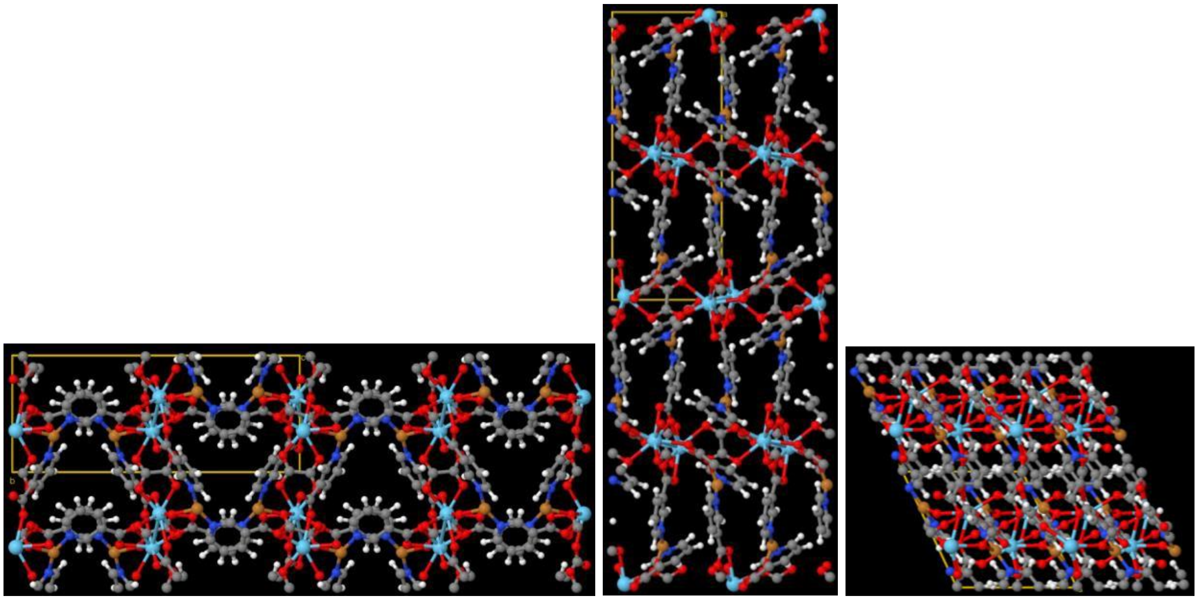

Figure S79: MIZKOW contains the chemical elements La, Cu, H, C, N, and O.

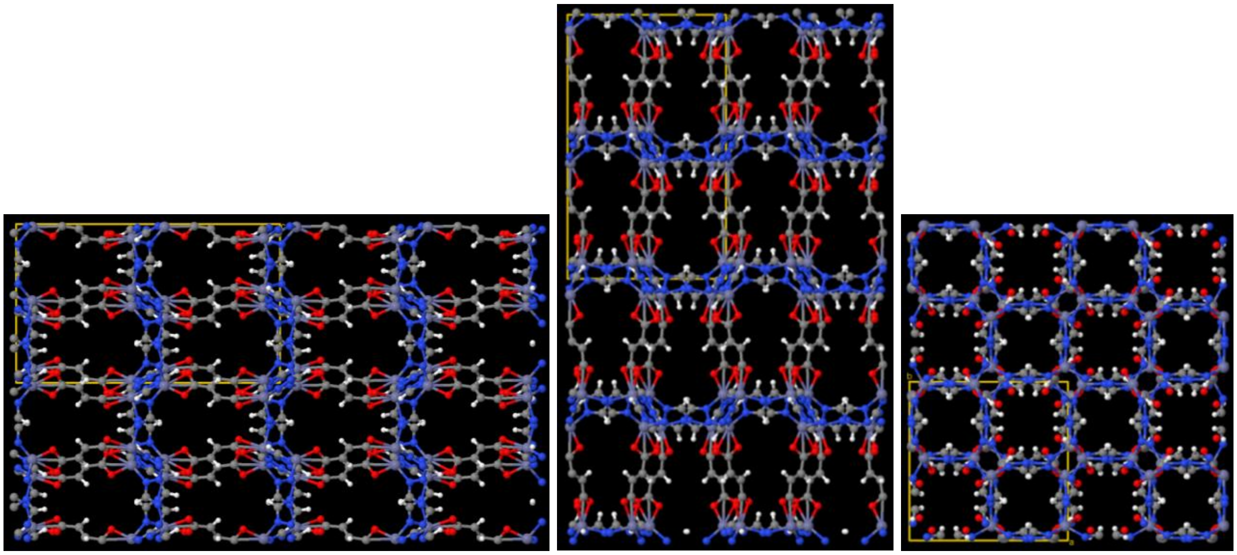

Figure S80: MOPFOO contains the chemical elements O, H, N, C, and Zn.

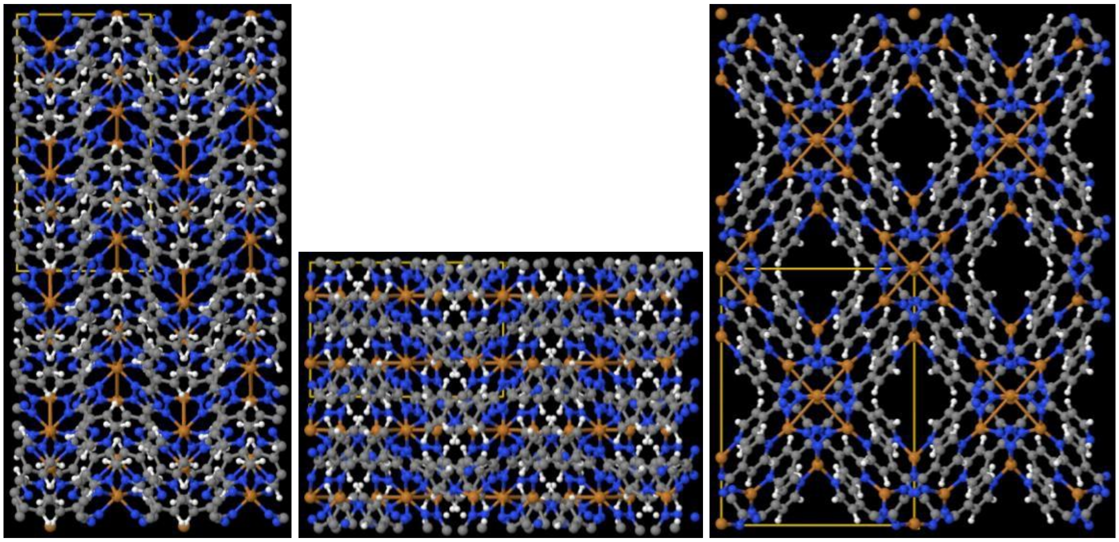

Figure S81: MUGQAI contains the chemical elements Cu, N, C, and H.

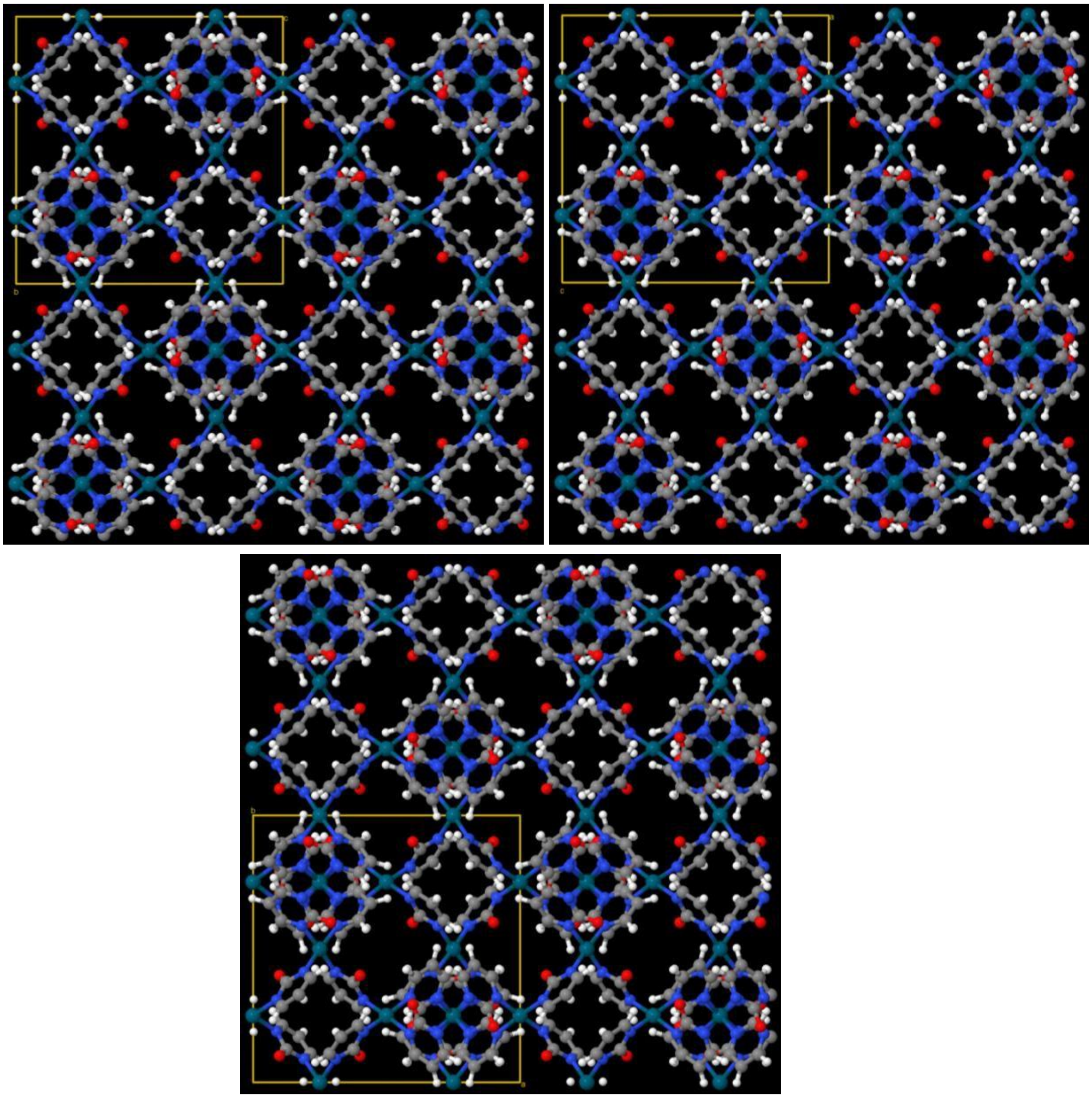

Figure S82: NEKREB contains the chemical elements N, C, H, Pd, and O.

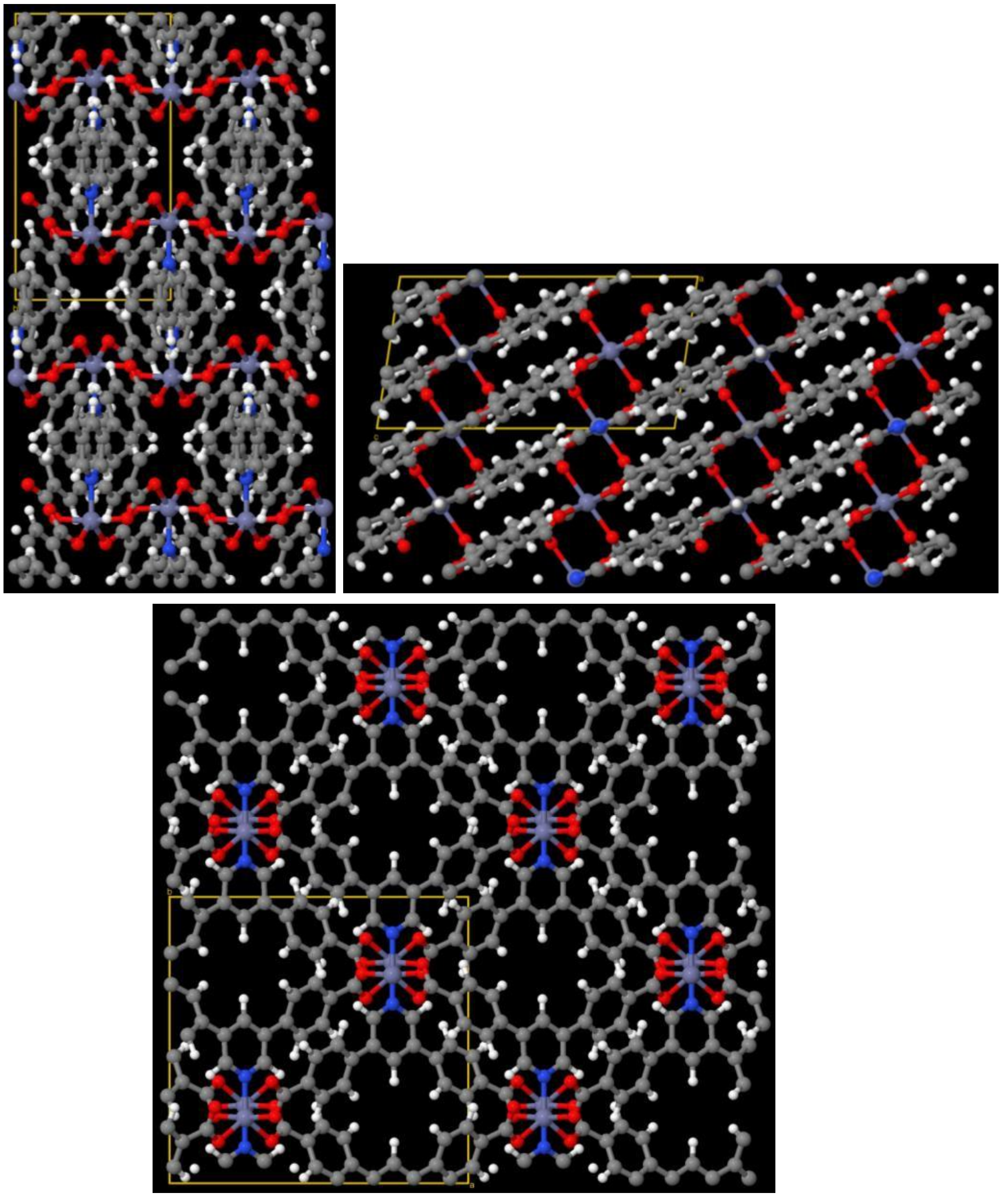

Figure S83: NOMQAJ01 contains the chemical elements C, H, N, O, and Zn.

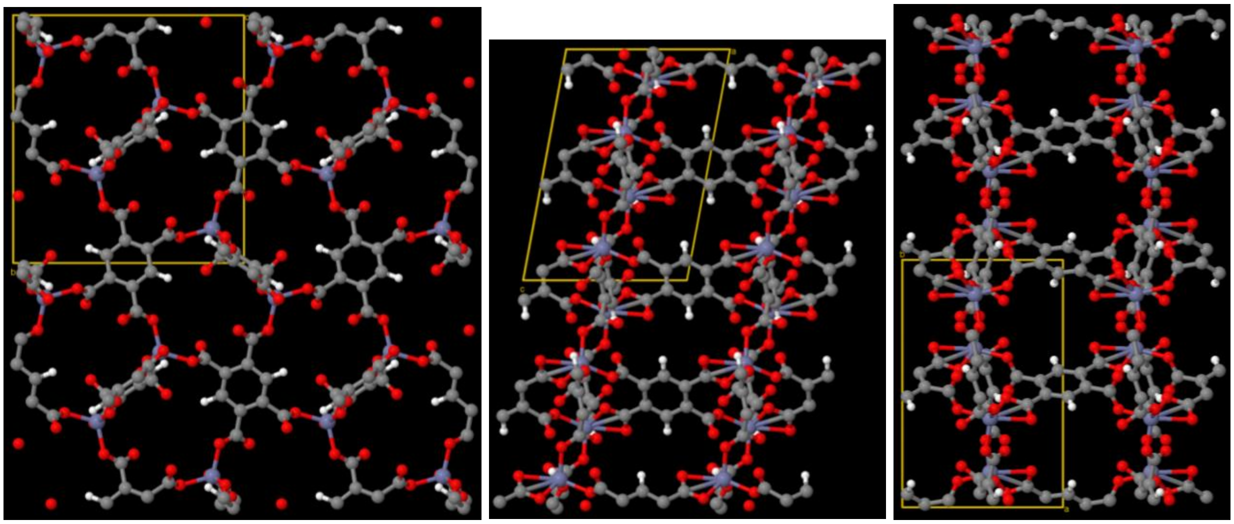

Figure S84: OGIBUD contains the chemical elements Zn, O, C, and H.

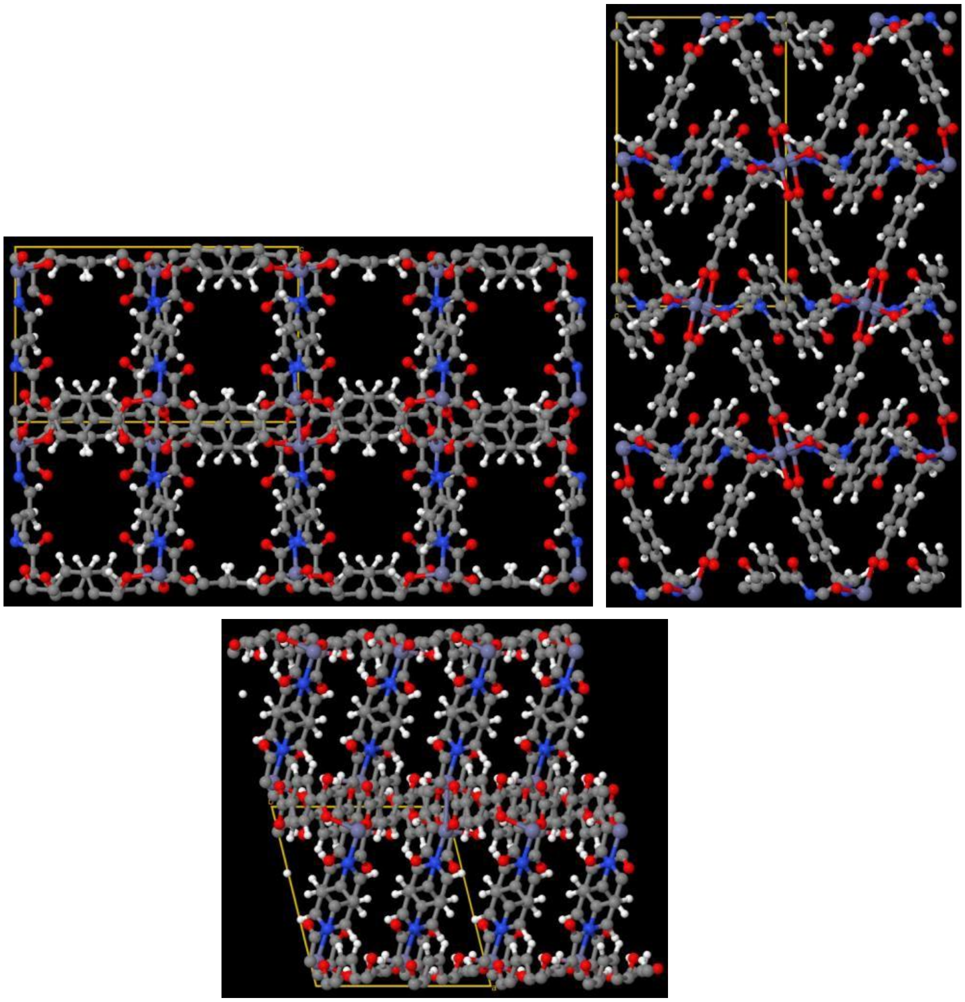

Figure S85: OJIWIO contains the chemical elements Zn, H, C, N, and O.

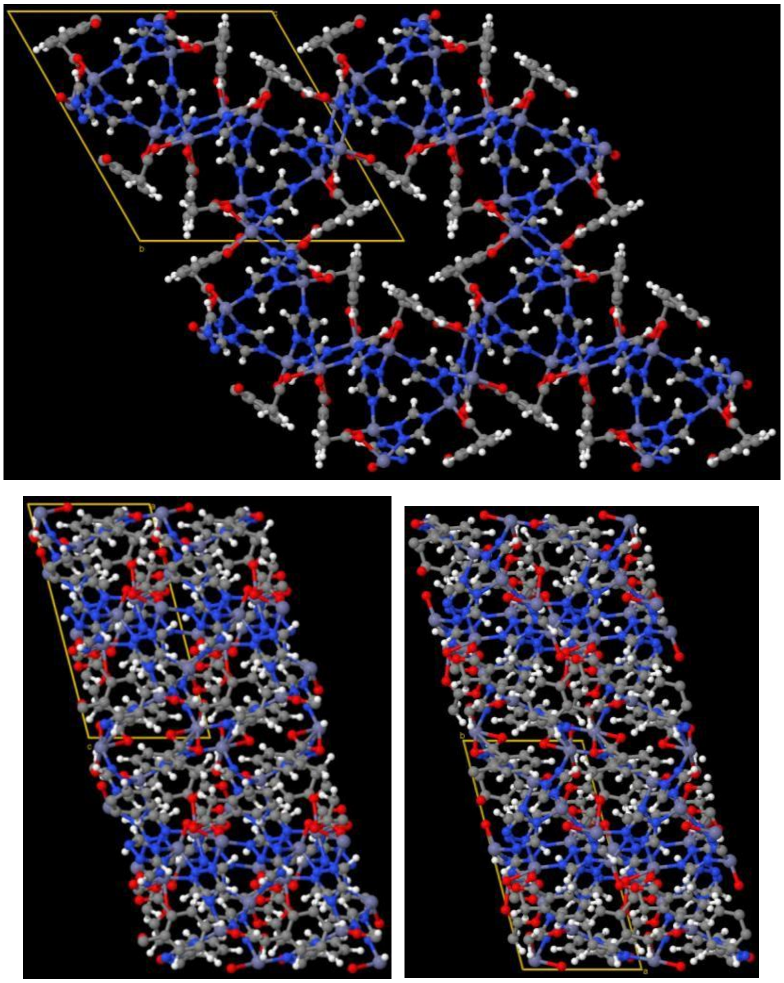

Figure S86: OPOBIF contains the chemical elements Zn, H, C, N, and O.

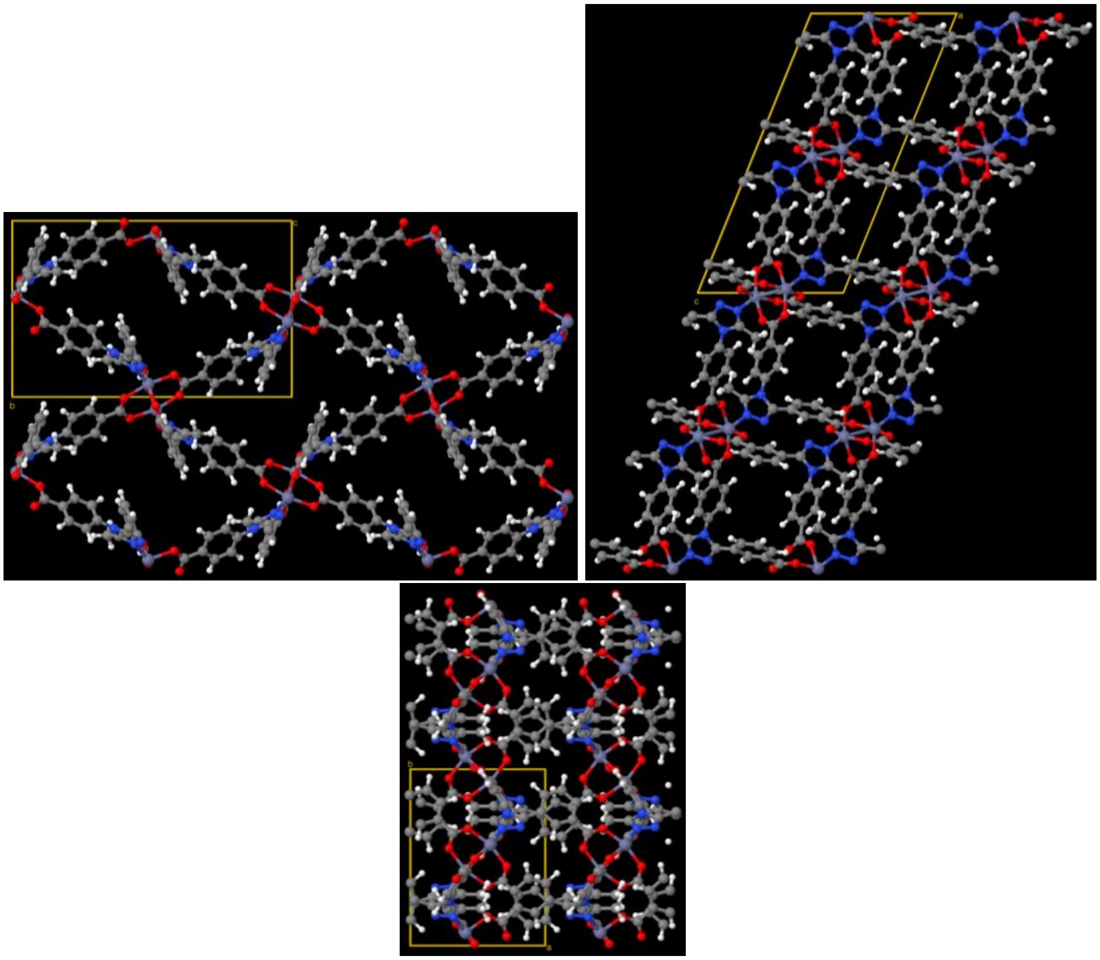

Figure S87: OTOZOO contains the chemical elements Zn, C, H, O, and N.

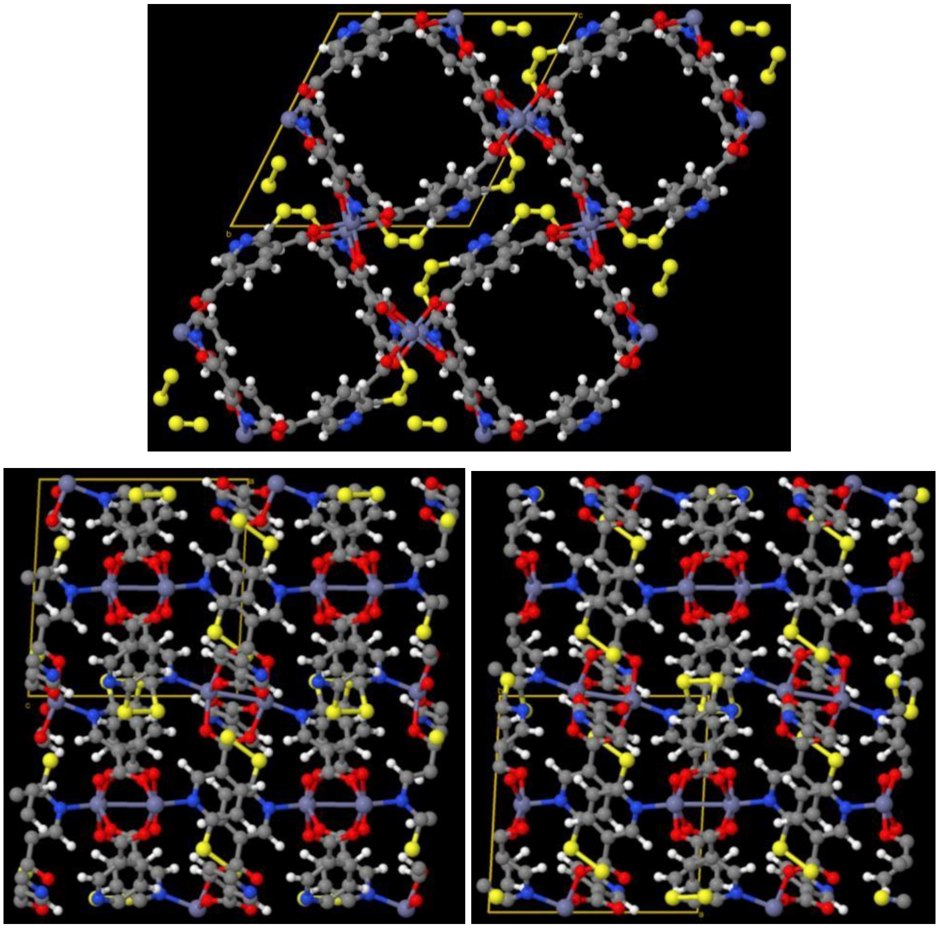

Figure S88: OVICUS contains the chemical elements Zn, H, C, S, N, and O.

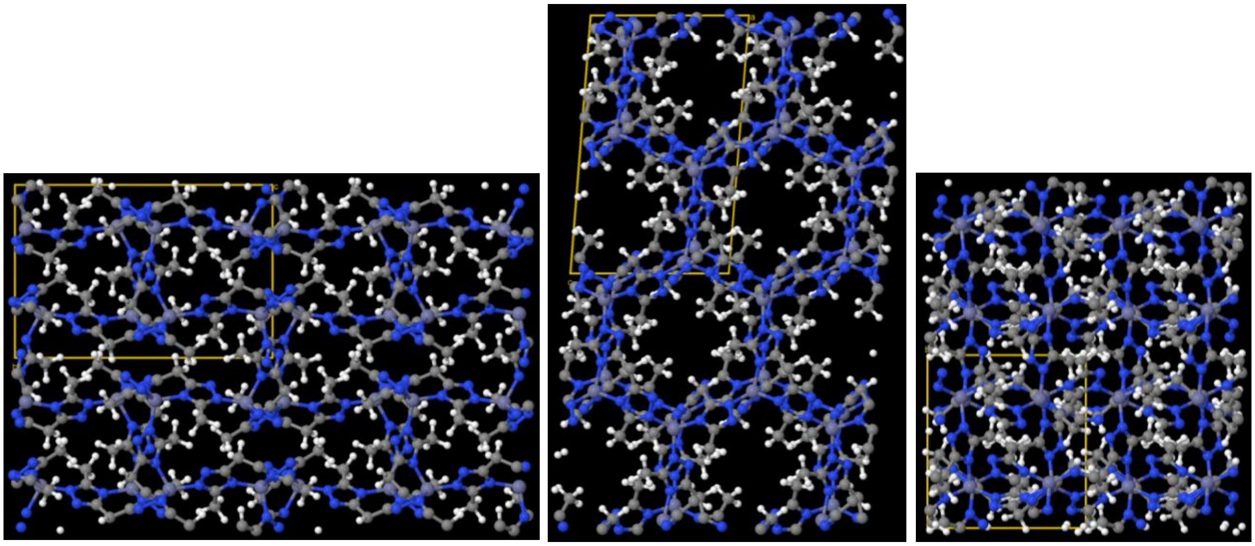

Figure S89: PEGBEK01 contains the chemical elements N, H, Zn, and C.

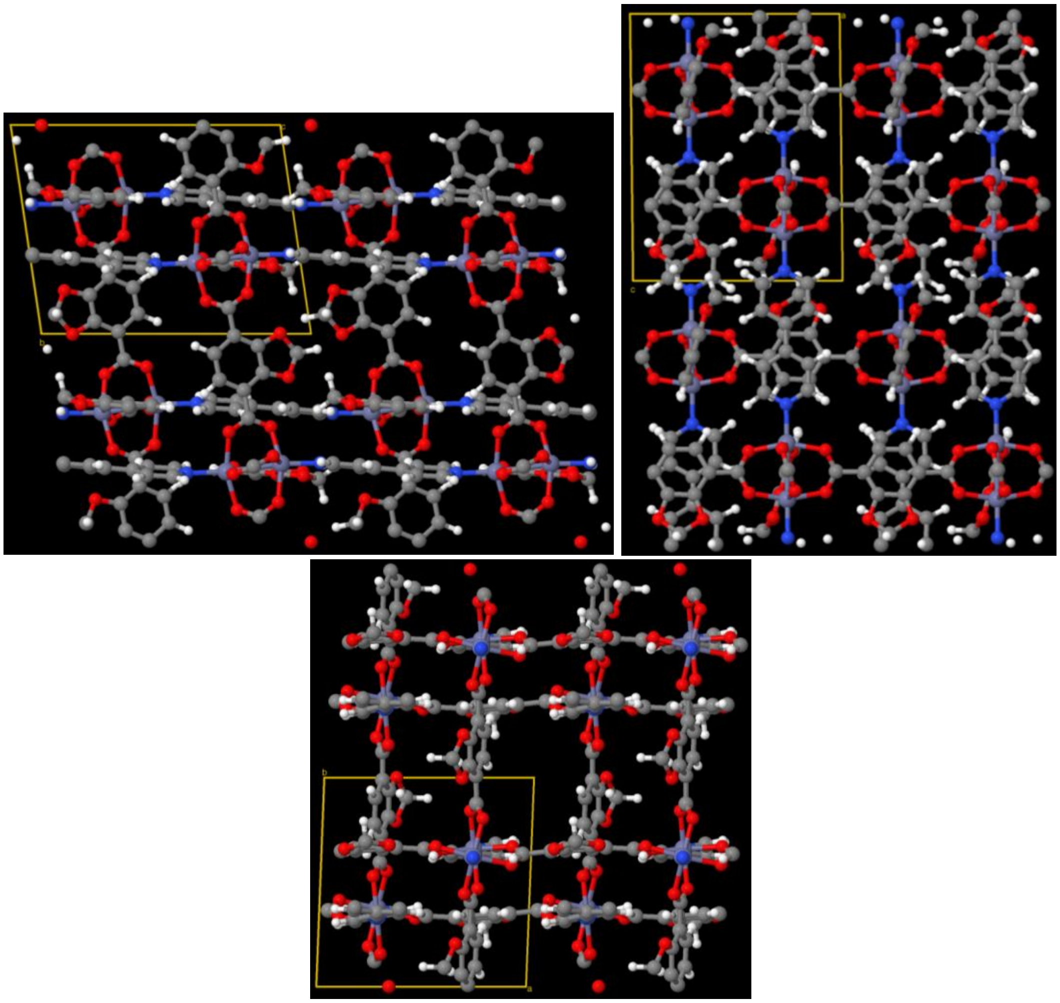

Figure S90: PEYPIU contains the chemical elements Zn, H, C, N, and O.

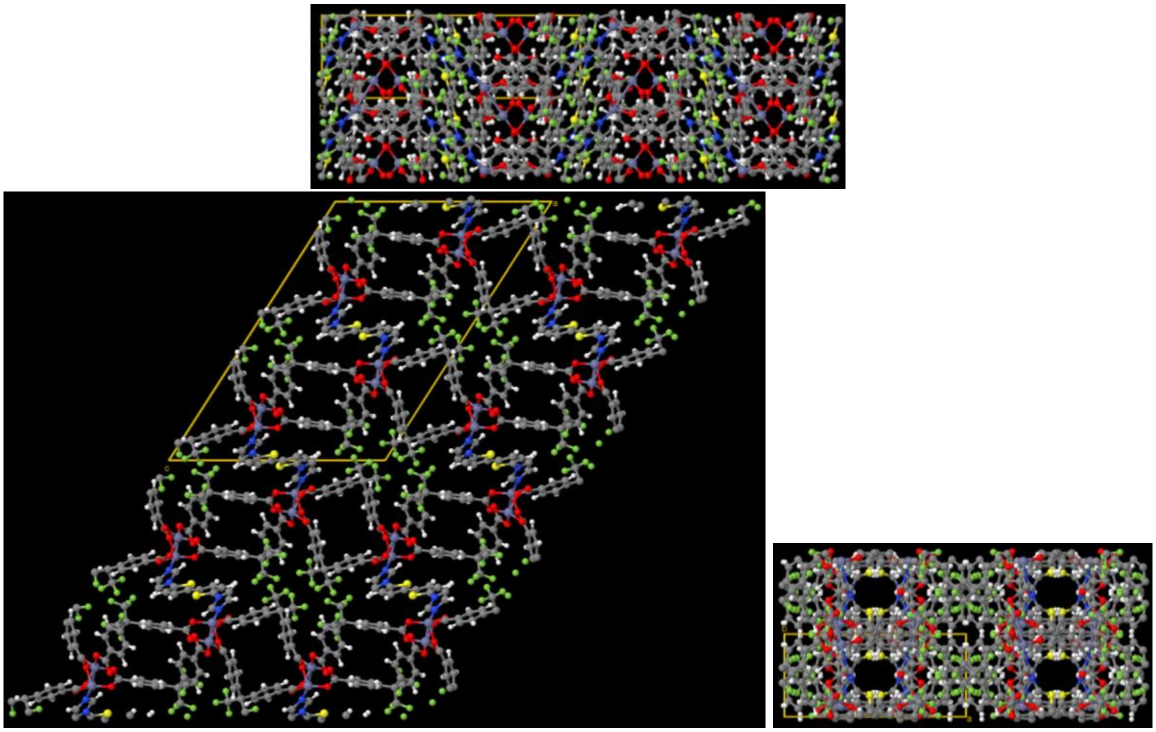

Figure S91: PITMOW contains the chemical elements F, C, H, O, Zn, N, and S.

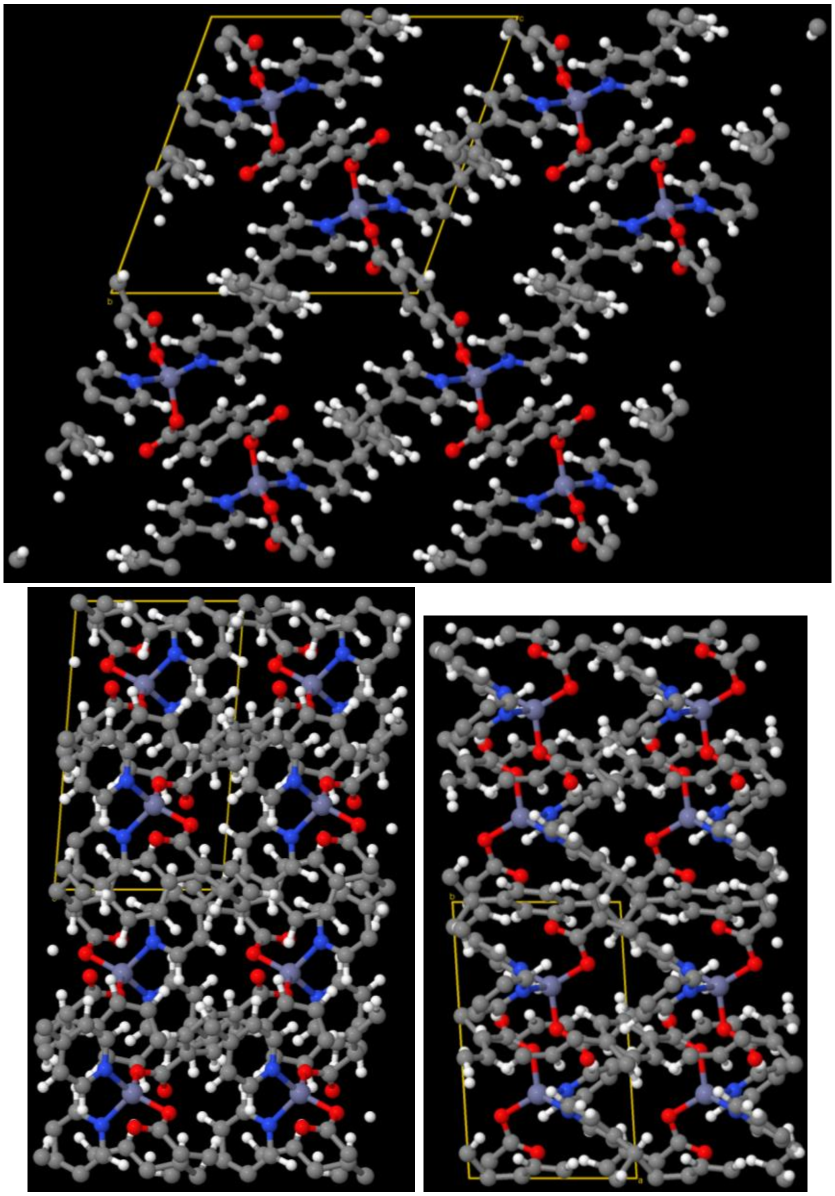

Figure S92: PIXHUB contains the chemical elements O, C, Zn, H, and N.

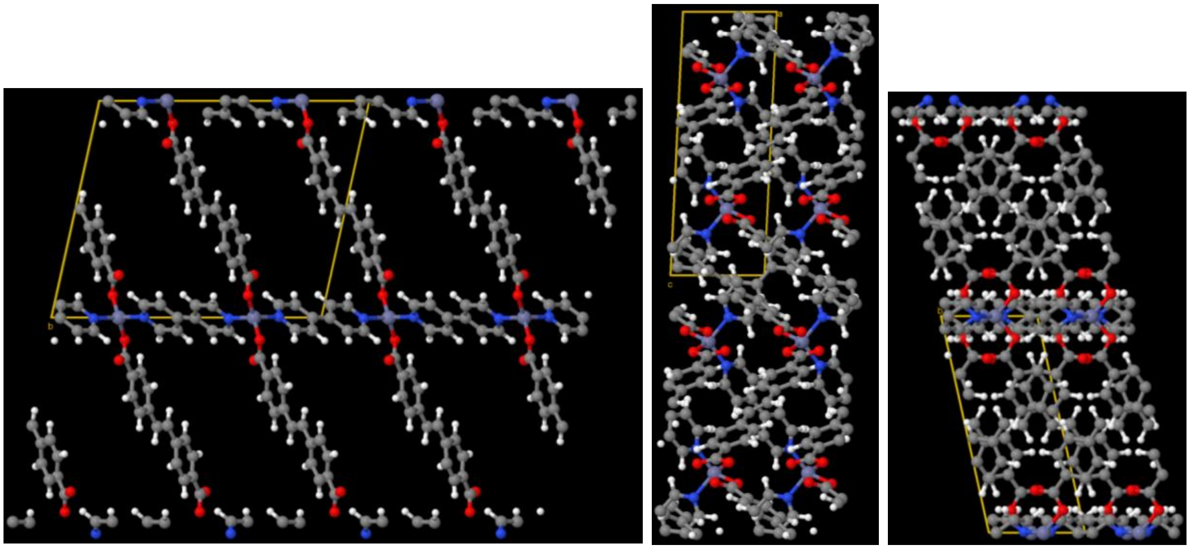

Figure S93: PORVUO contains the chemical elements Zn, H, C, N, and O.

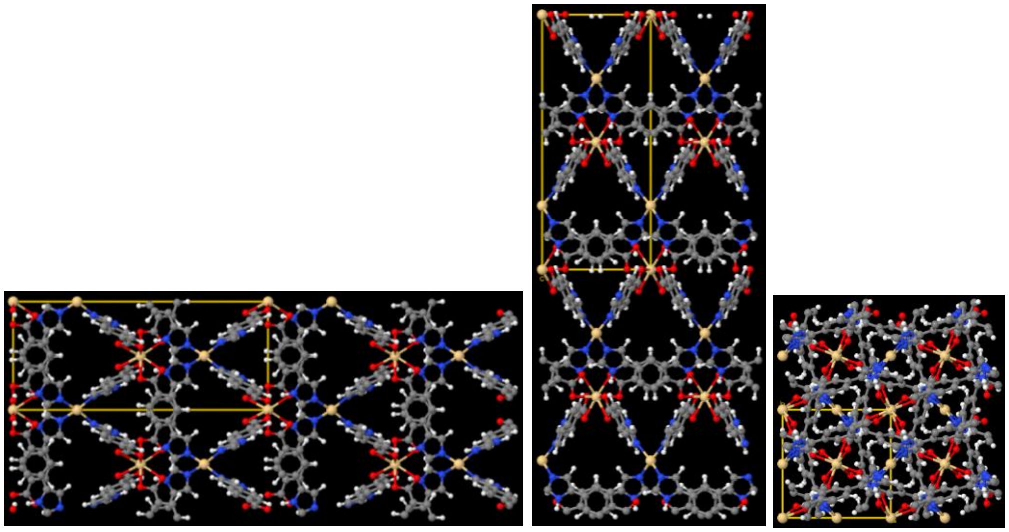

Figure S94: POSFOU contains the chemical elements Cd, H, C, N, and O.

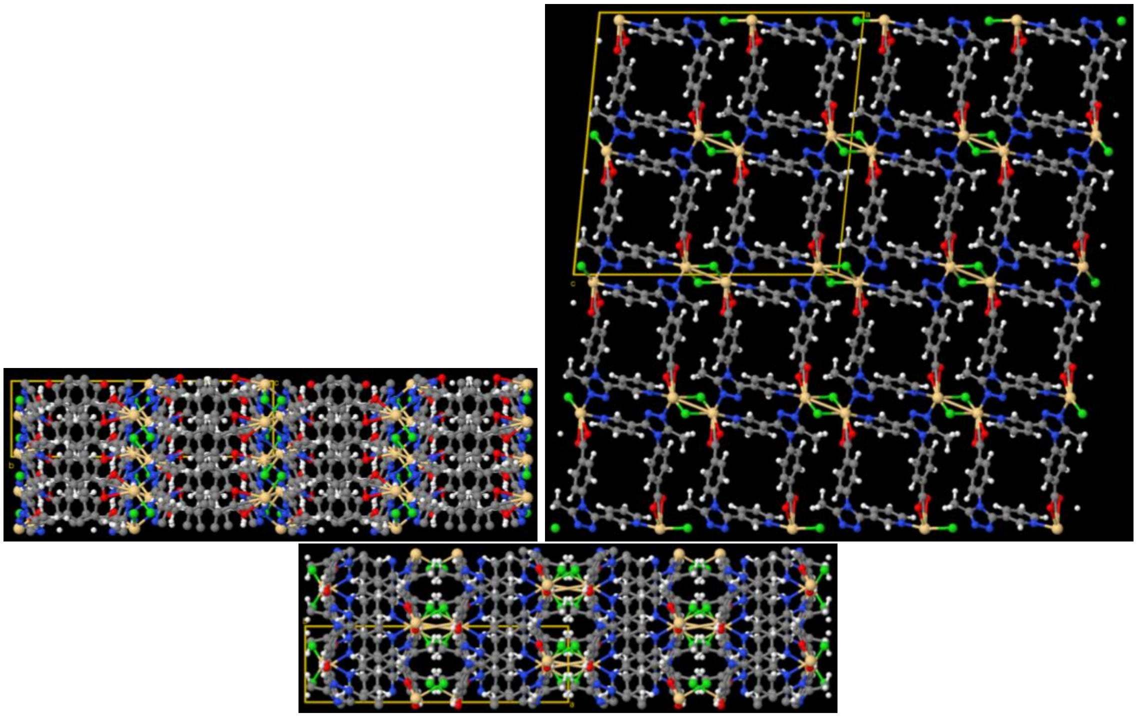

Figure S95: PUFVUJ contains the chemical elements C, H, Cd, Cl, N, and O.

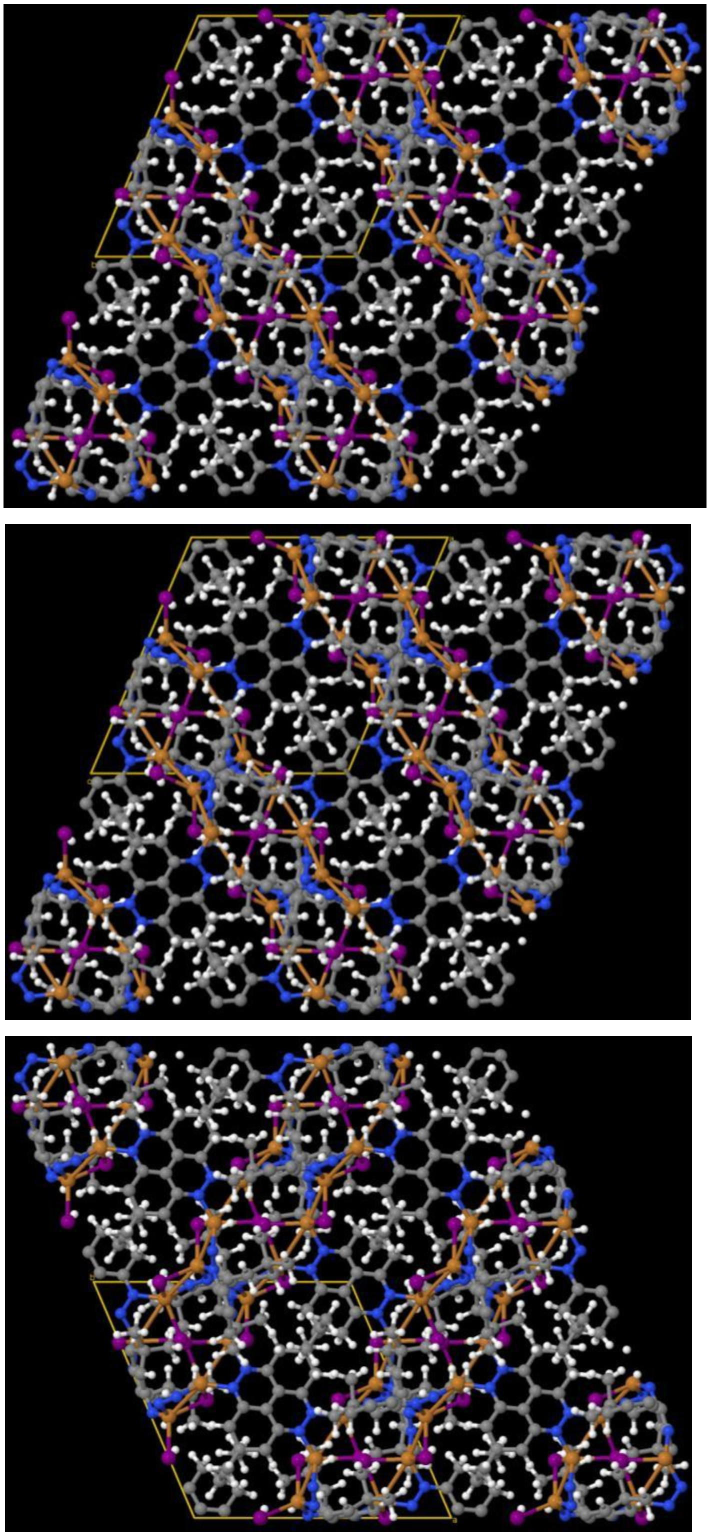

Figure S96: QESYOD contains the chemical elements Cu, H, C, I, and N.

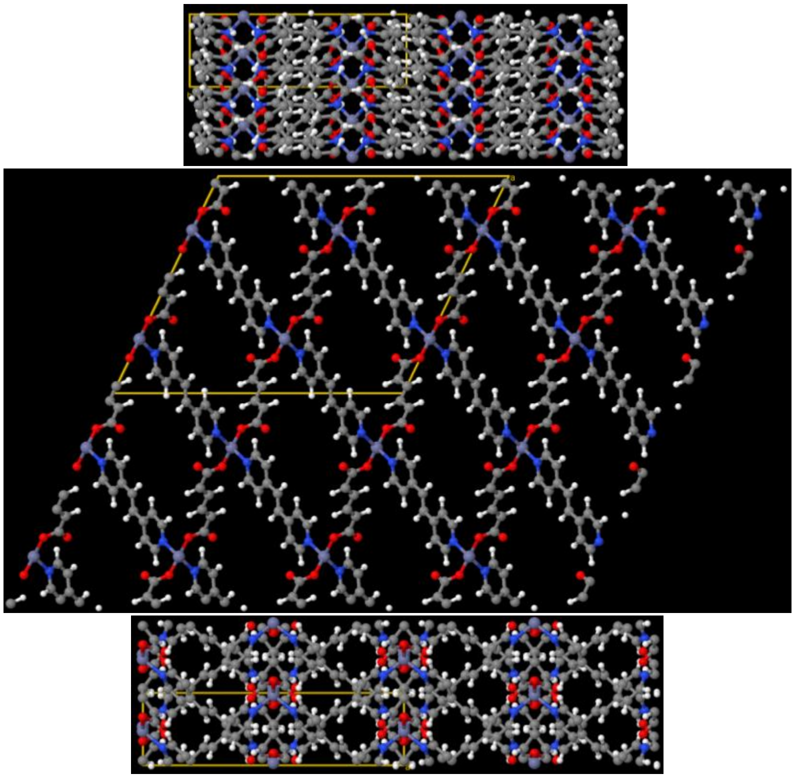

Figure S97: QIVYUR contains the chemical elements C, H, O, N, and Zn.

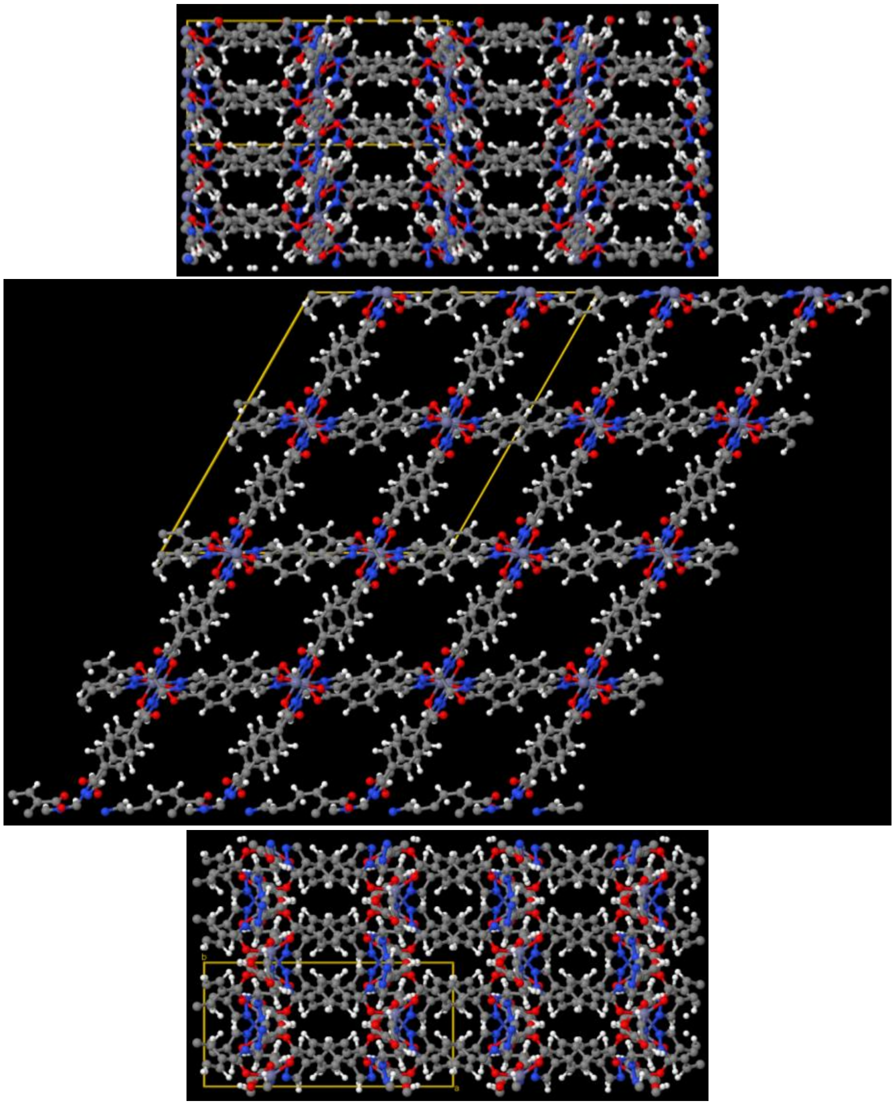

Figure S98: QIWCUW contains the chemical elements O, H, Zn, N, and C.

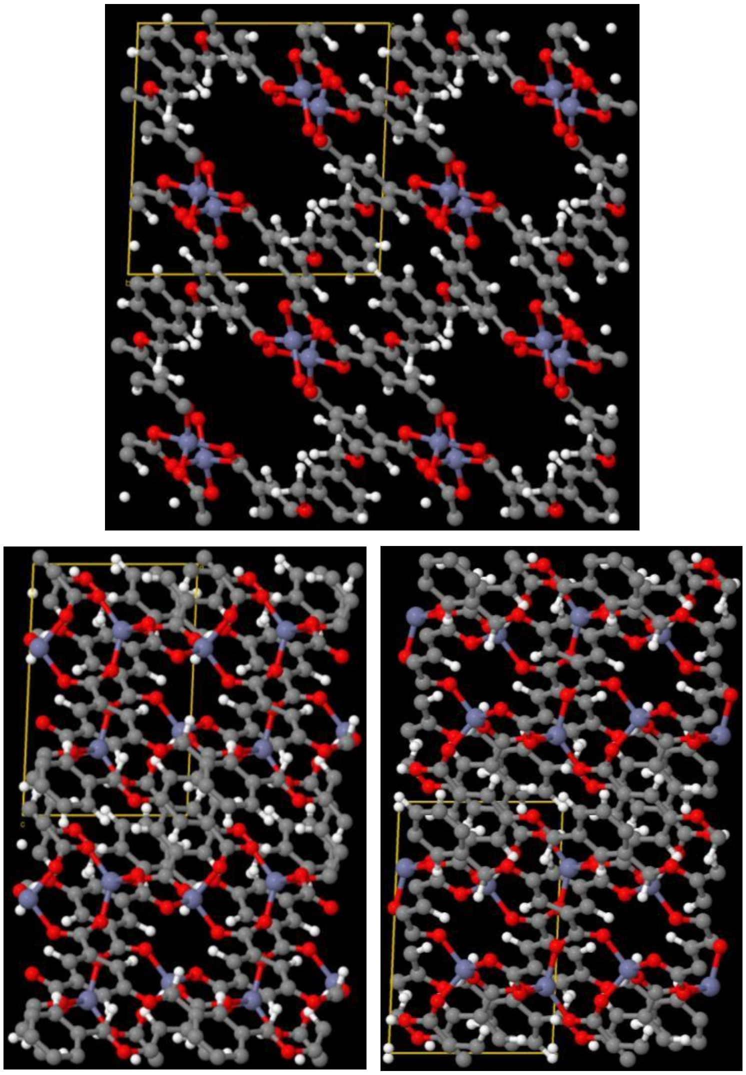

Figure S99: QOKCID contains the chemical elements Zn, H, C, and O.

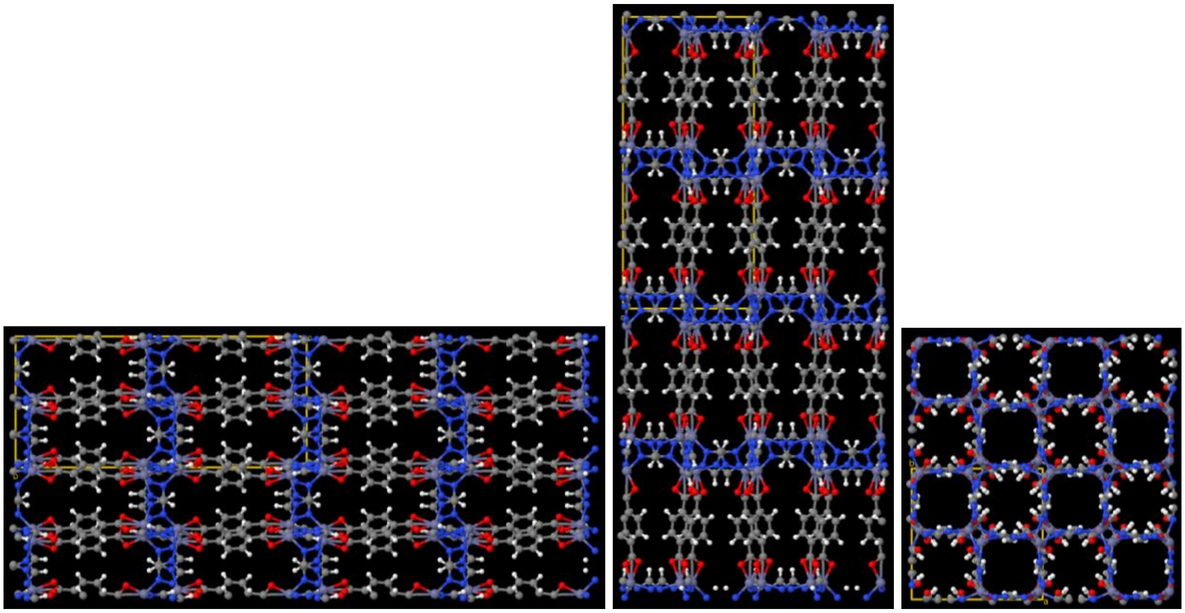

Figure S100: QOTBAE contains the chemical elements N, C, H, Zn, and O.

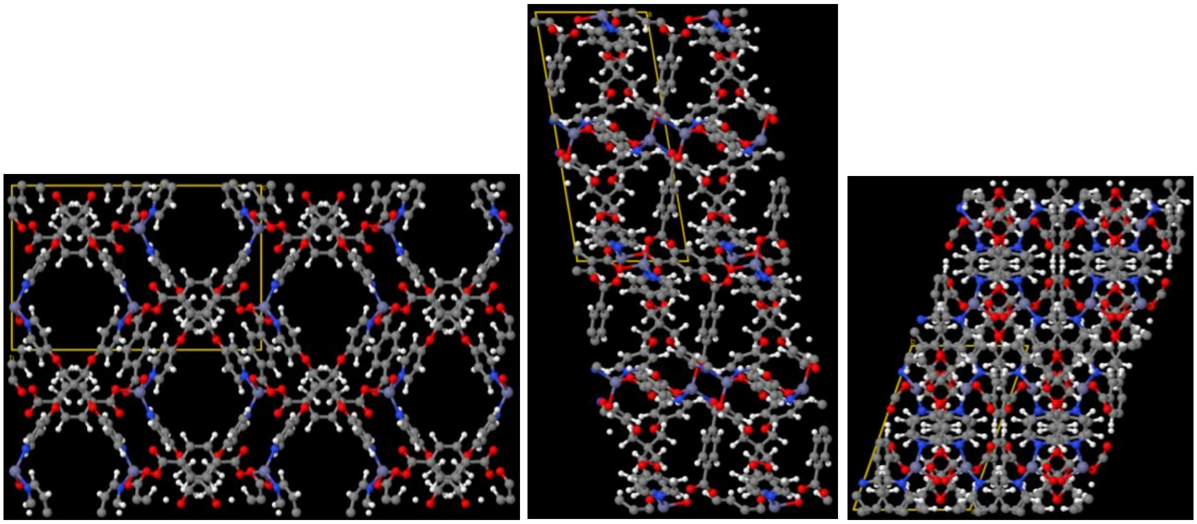

Figure S101: QUPHUF contains the chemical elements Zn, H, C, N, and O.

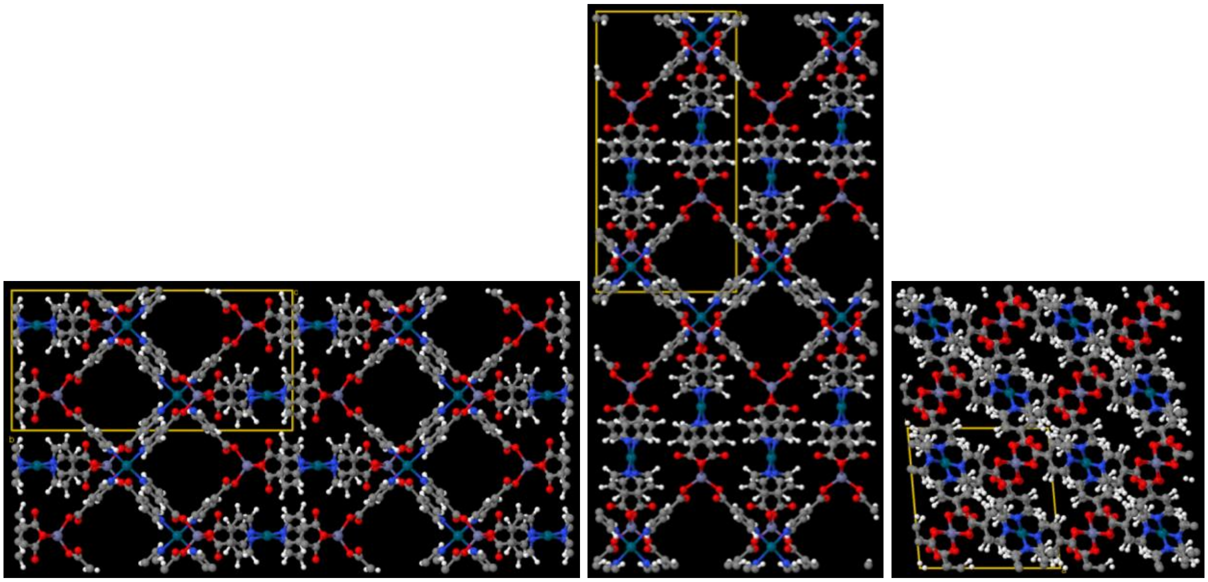

Figure S102: QURSEC contains the chemical elements Zn, H, Pd, C, N, and O.

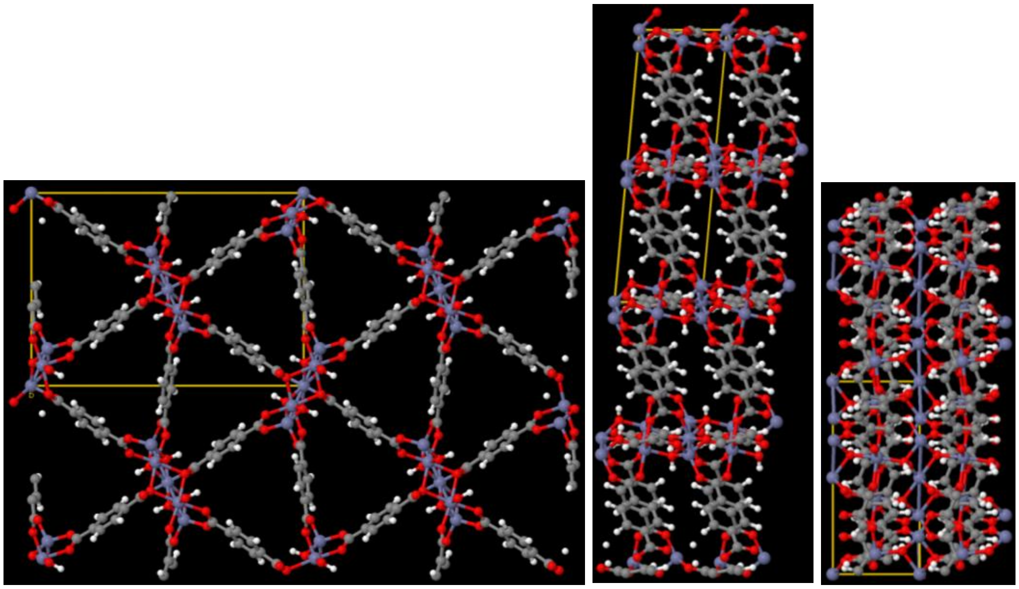

Figure S103: REDROI contains the chemical elements Zn, H, C, and O.

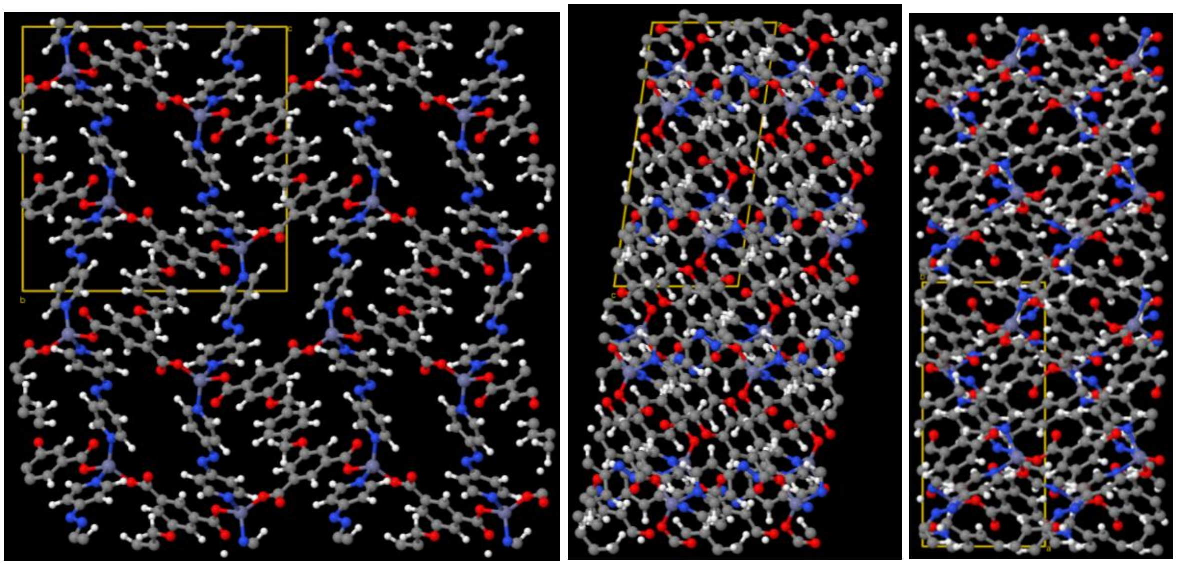

Figure S104: REYBAA contains the chemical elements Zn, H, C, N, and O.

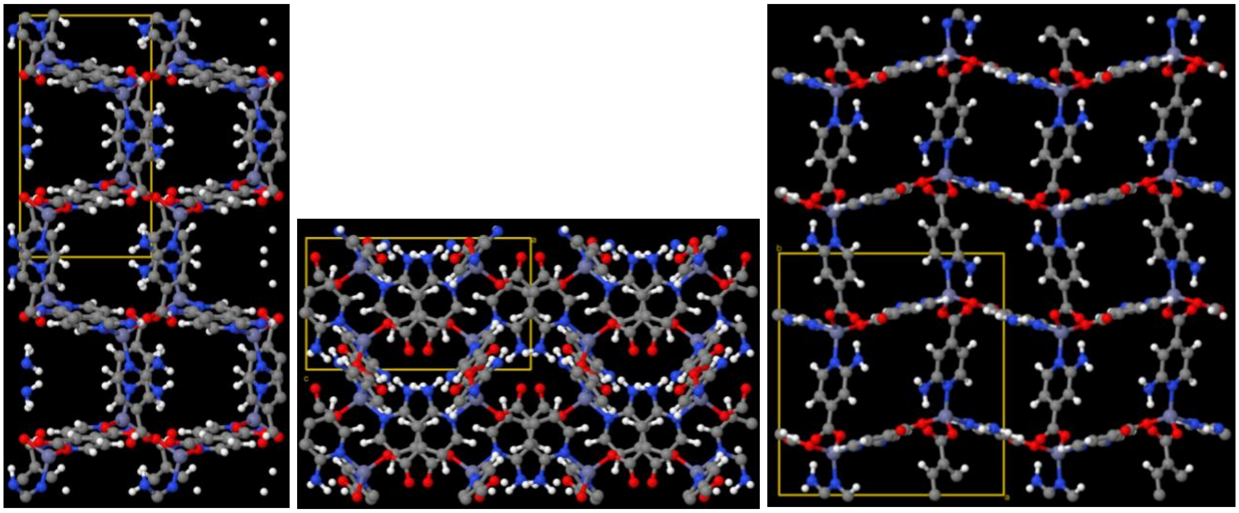

Figure S105: SARBOE contains the chemical elements Zn, O, C, H, and N.

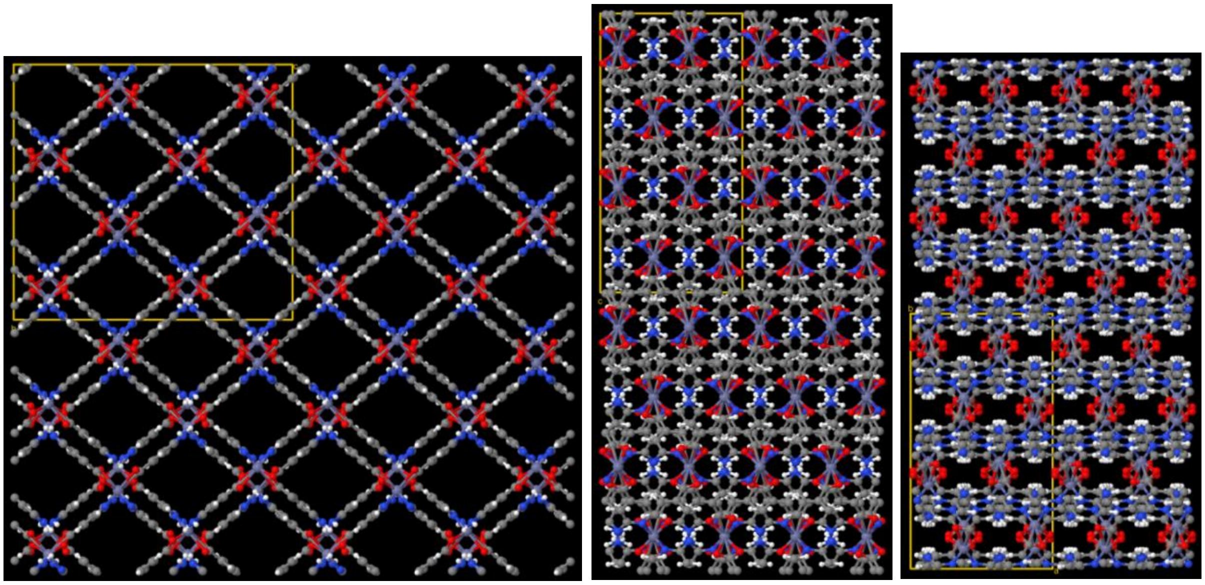

Figure S106: SARBUK contains the chemical elements Zn, C, H, N, and O.

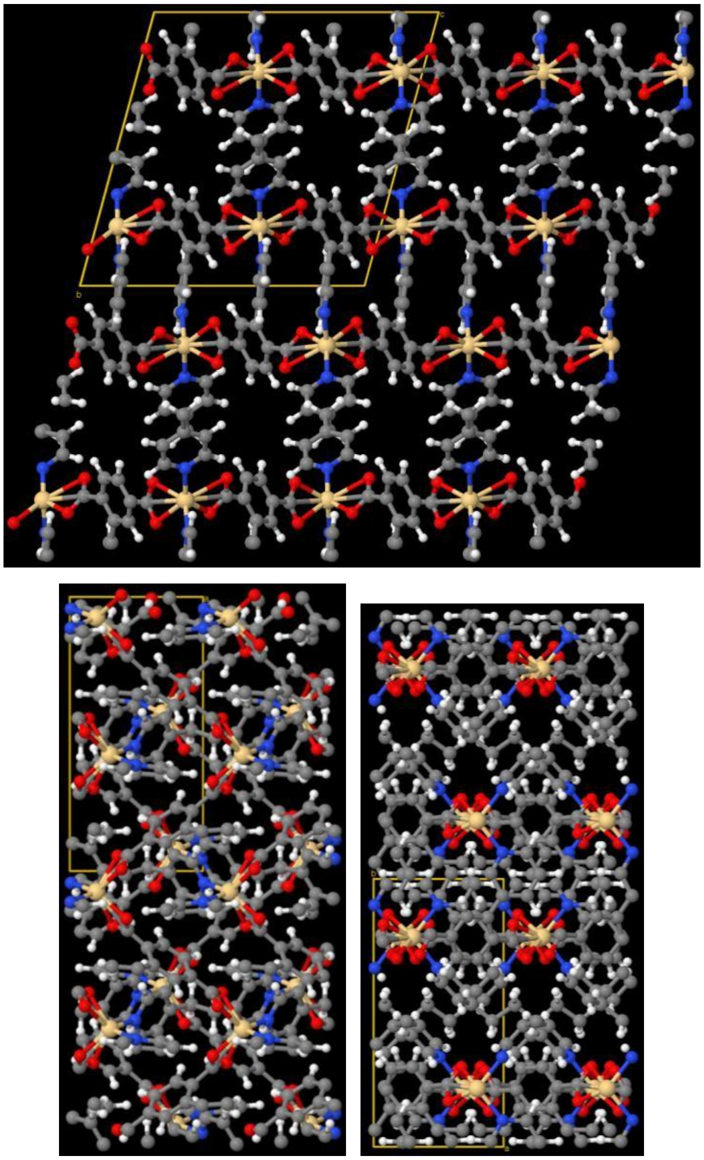

Figure S107: SOCLAZ contains the chemical elements Cd, H, C, N, and O.

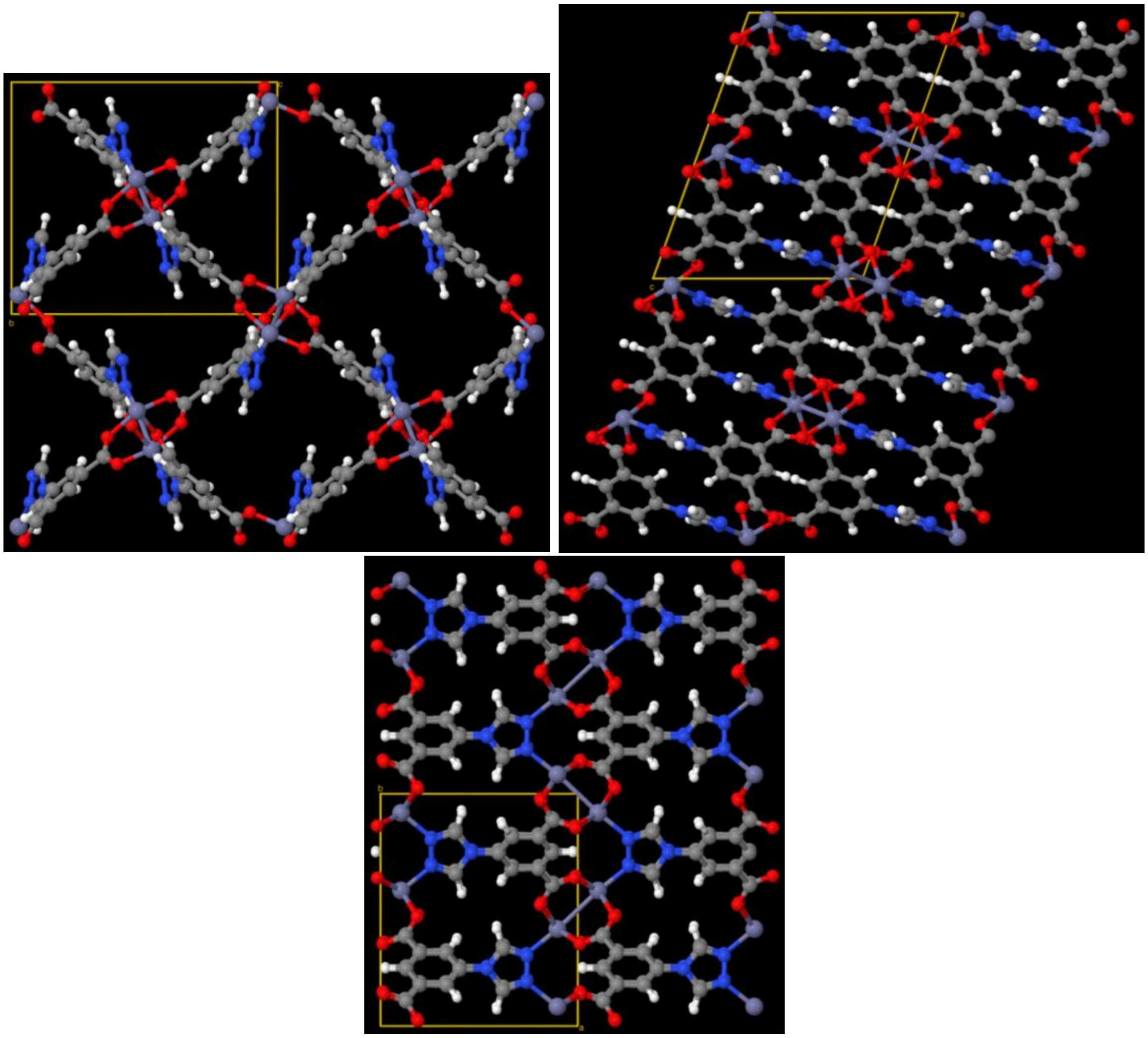

Figure S108: SOWCOY01 contains the chemical elements Zn, O, N, C, and H.

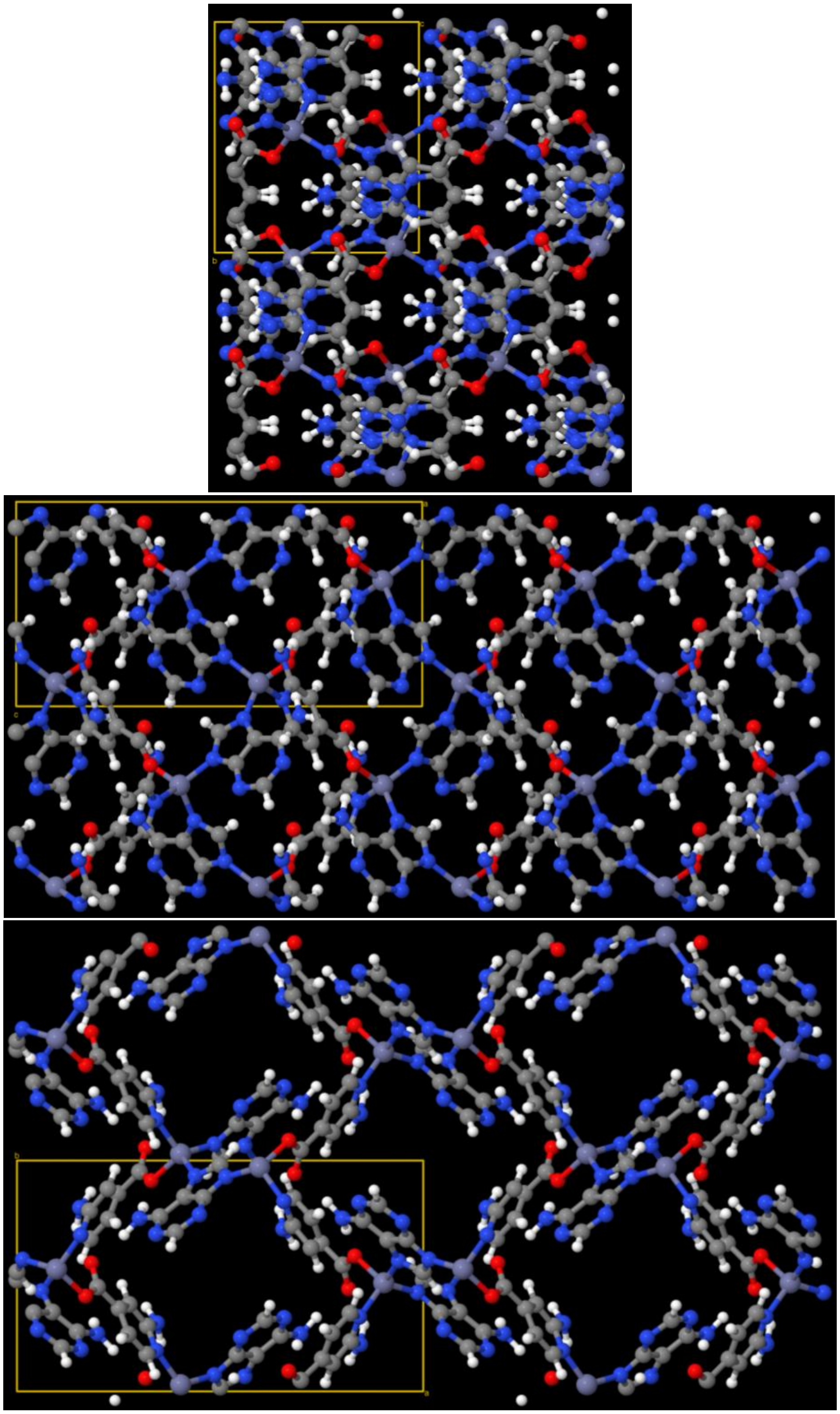

Figure S109: TEPGUS contains the chemical elements O, Zn, C, H, and N.

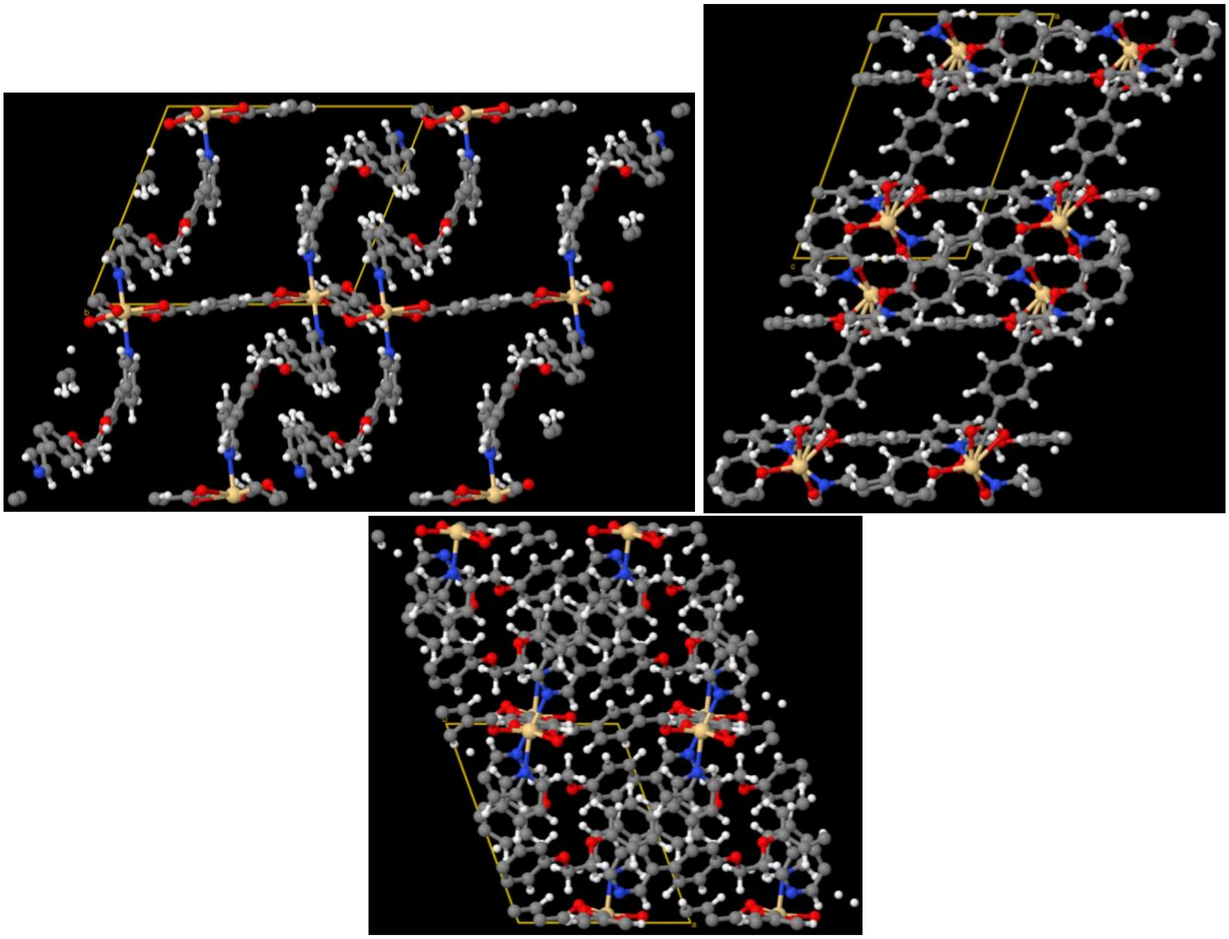

Figure S110: TUBLIN contains the chemical elements Cd, H, C, N, and O.

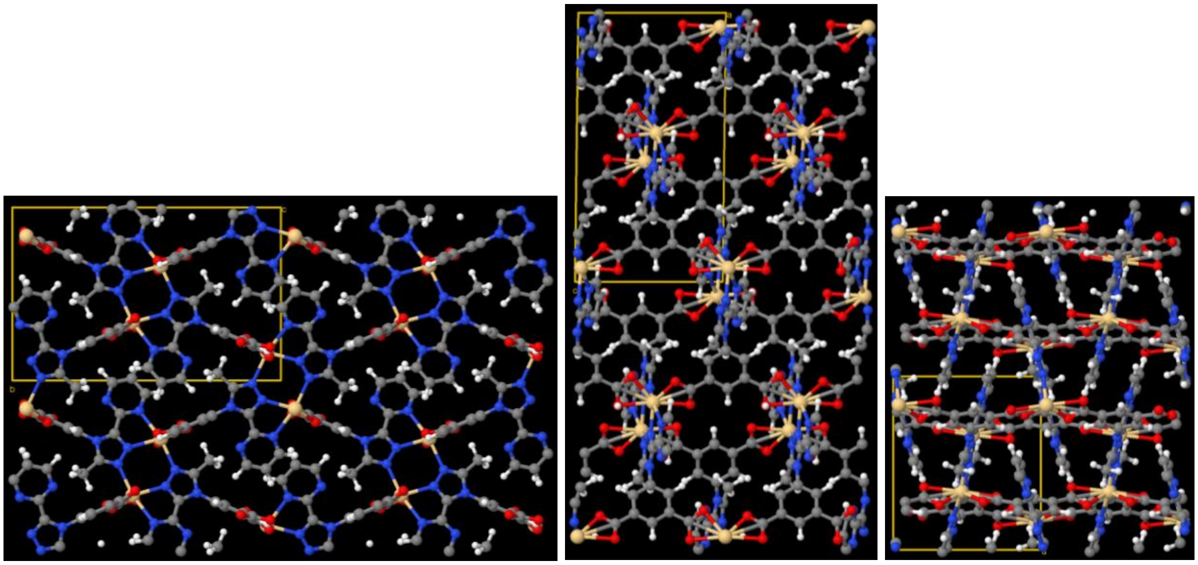

Figure S111: TUMDEM contains the chemical elements C, H, N, O, and Cd.

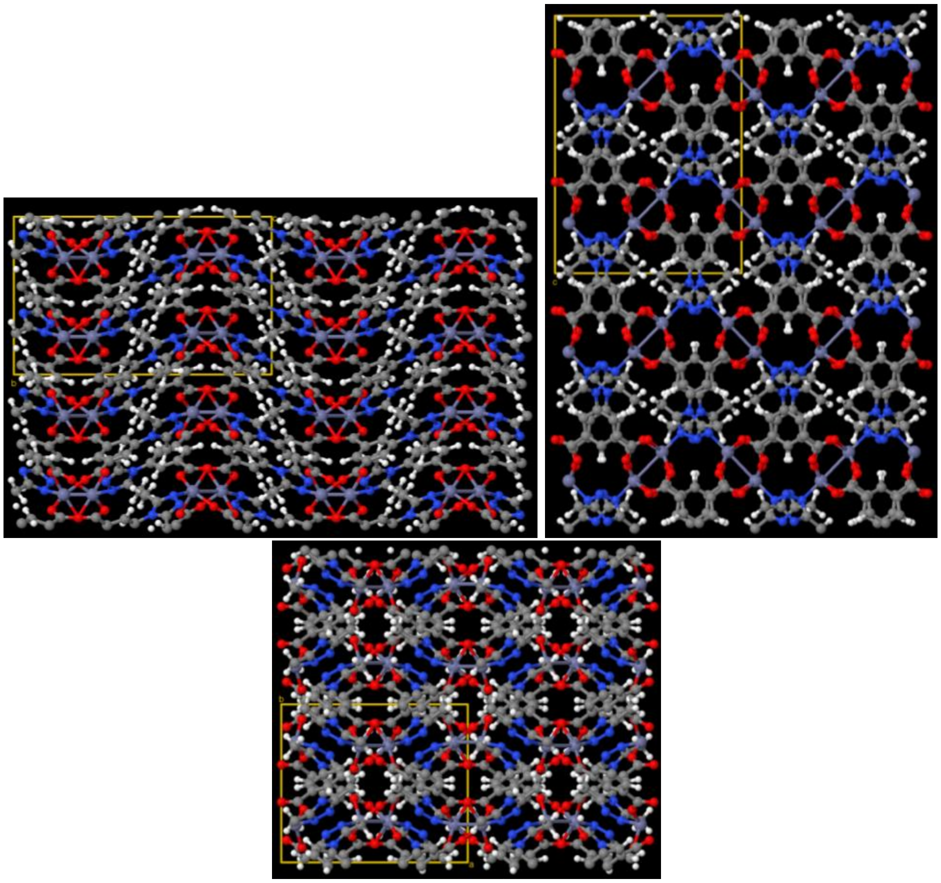

Figure S112: UKALEZ contains the chemical elements C, H, N, O, and Zn.

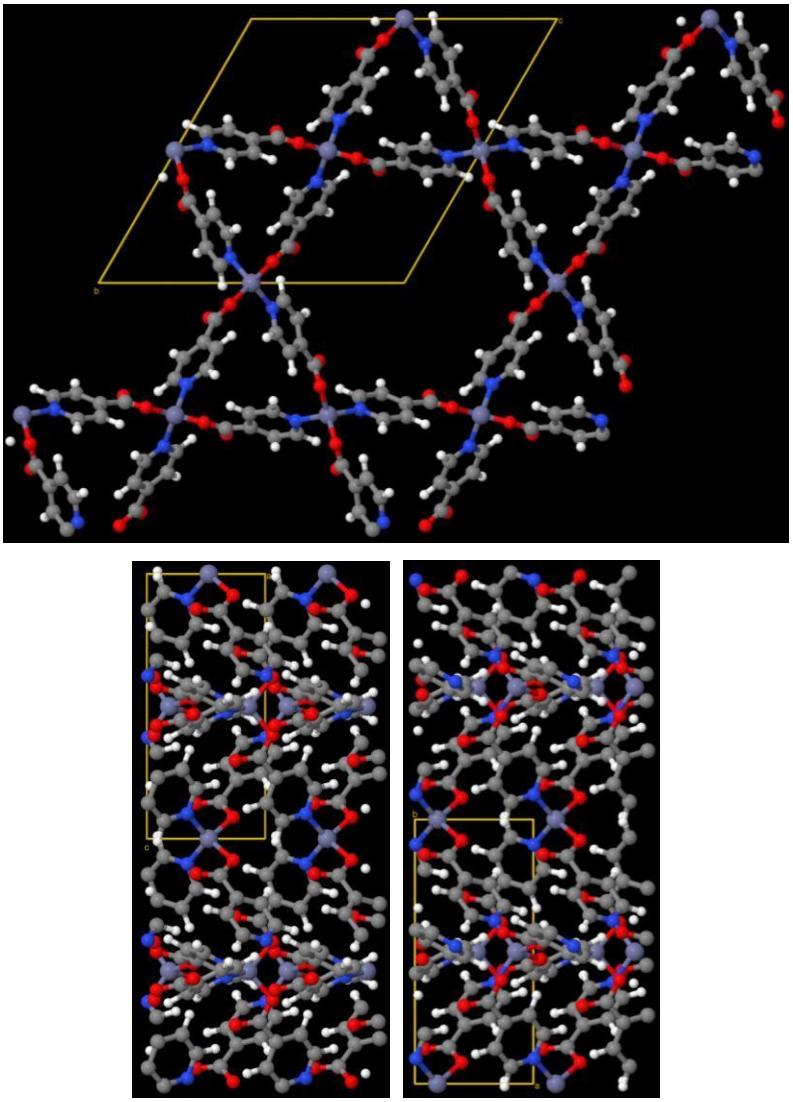

Figure S113: VACFUB01 contains the chemical elements Zn, H, C, N, and O.

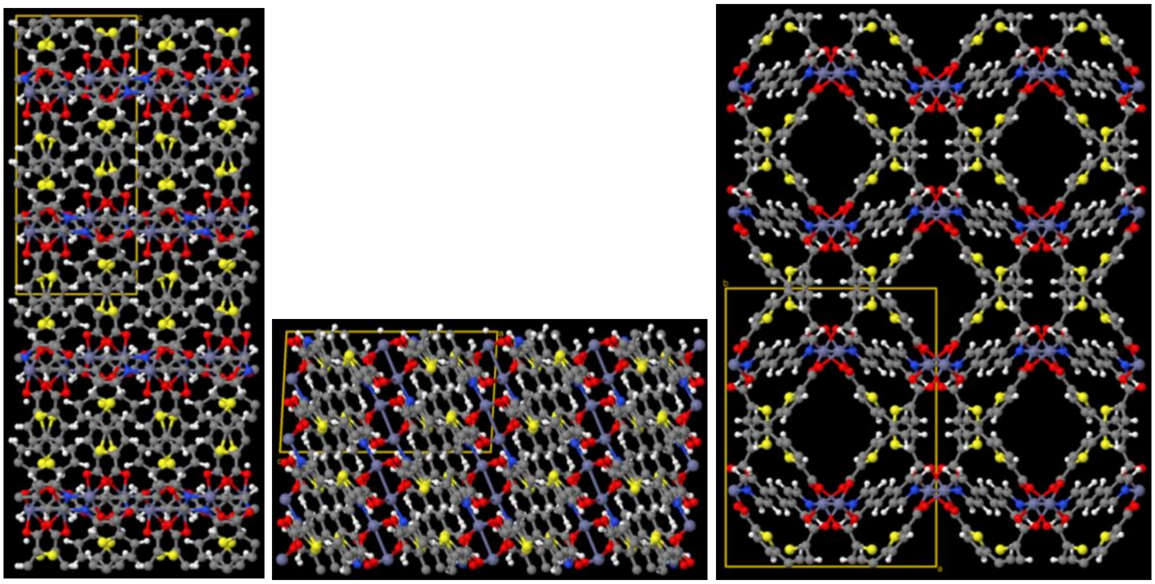

Figure S114: WECJAR contains the chemical elements Zn, C, H, N, O, and S.

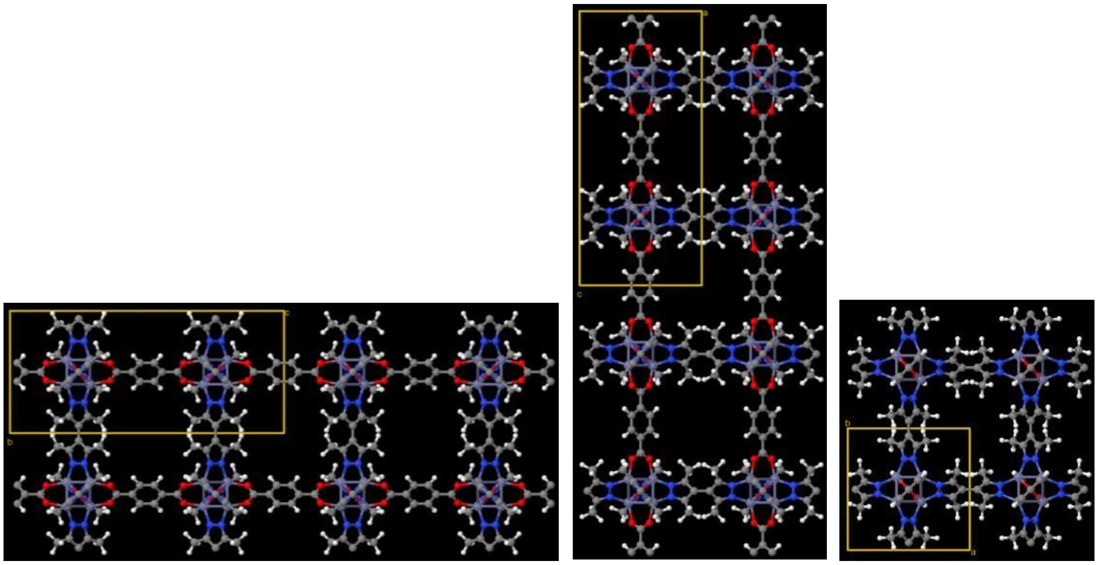

Figure S115: WIYFAM contains the chemical elements Zn, H, C, N, and O.

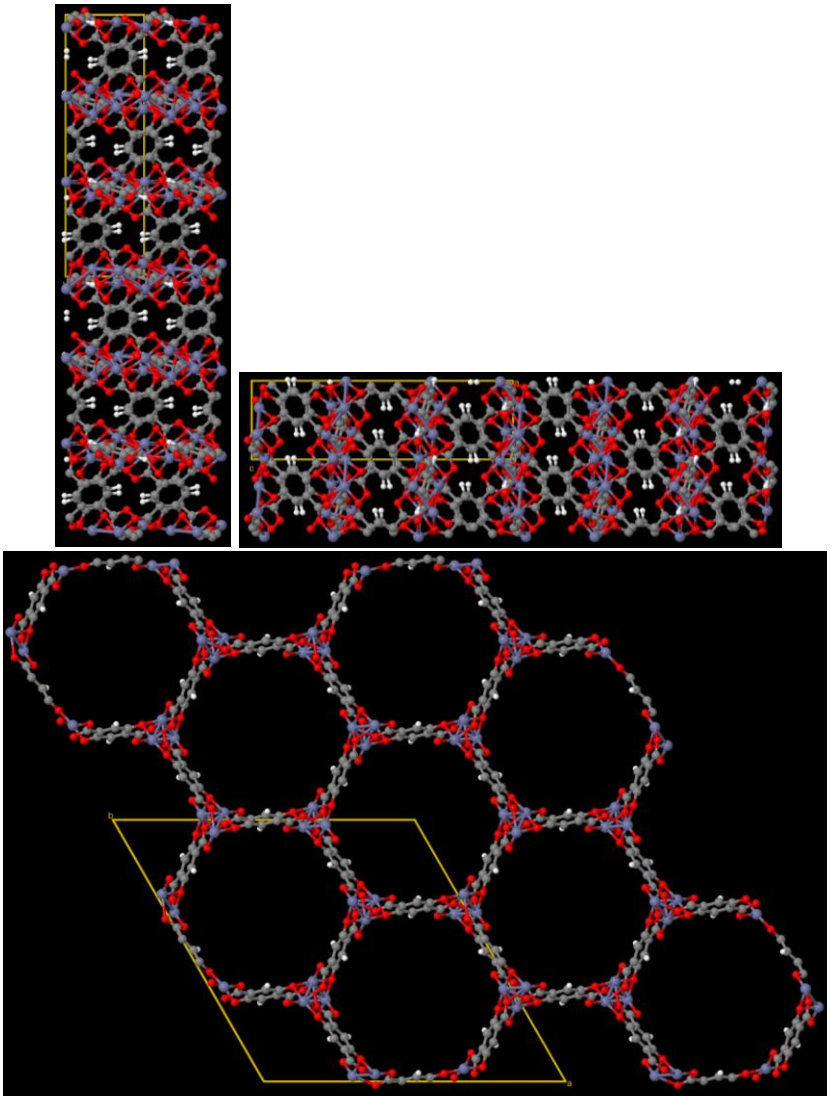

Figure S116: WOBHIF01 contains the chemical elements O, Zn, C, and H.

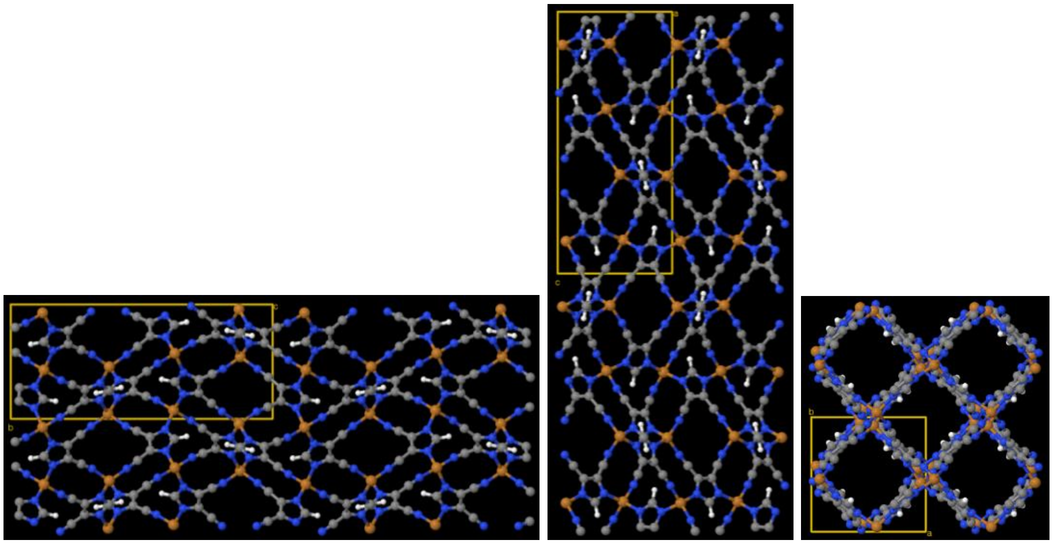

Figure S117: XAHROQ contains the chemical elements Cu, N, C, and H.

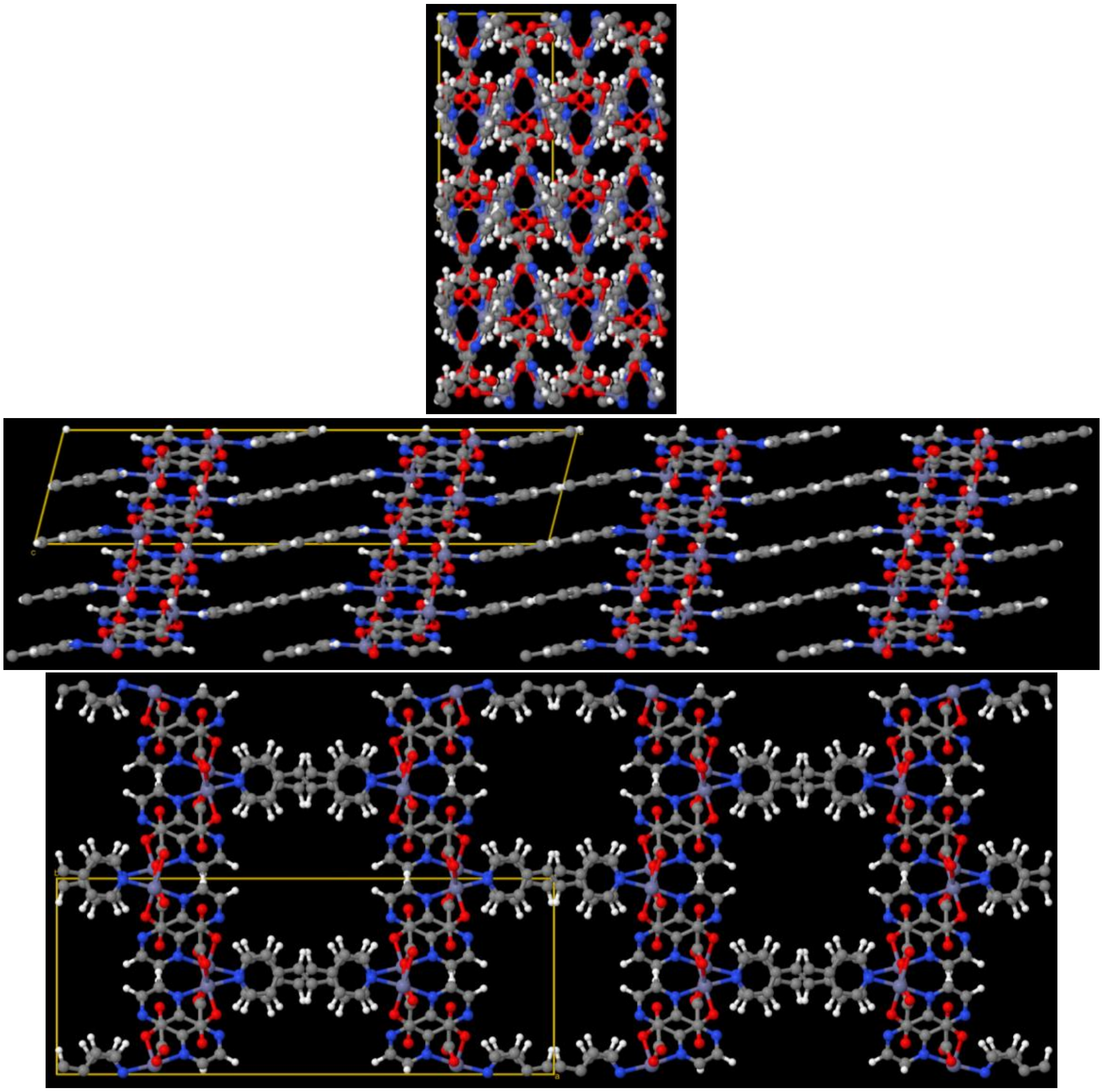

Figure S118: XARCEB contains the chemical elements H, Zn, C, N, and O.

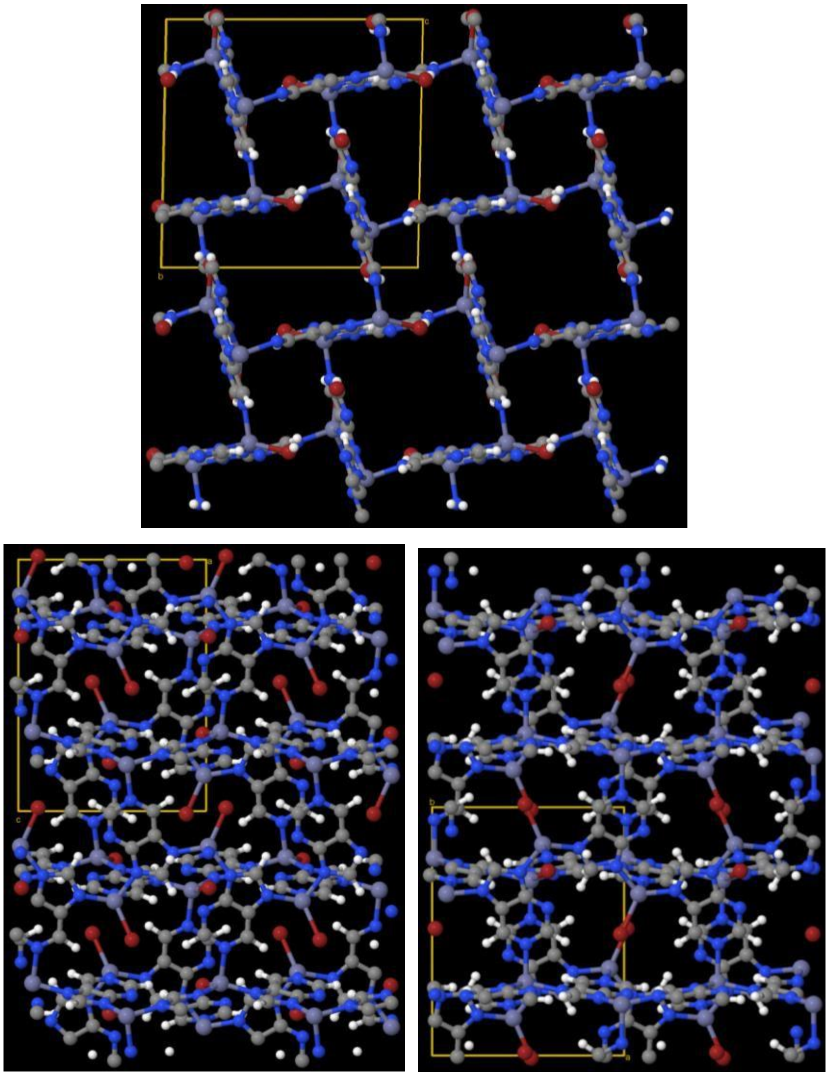

Figure S119: XEDPIH contains the chemical elements Zn, H, C, Br, and N.

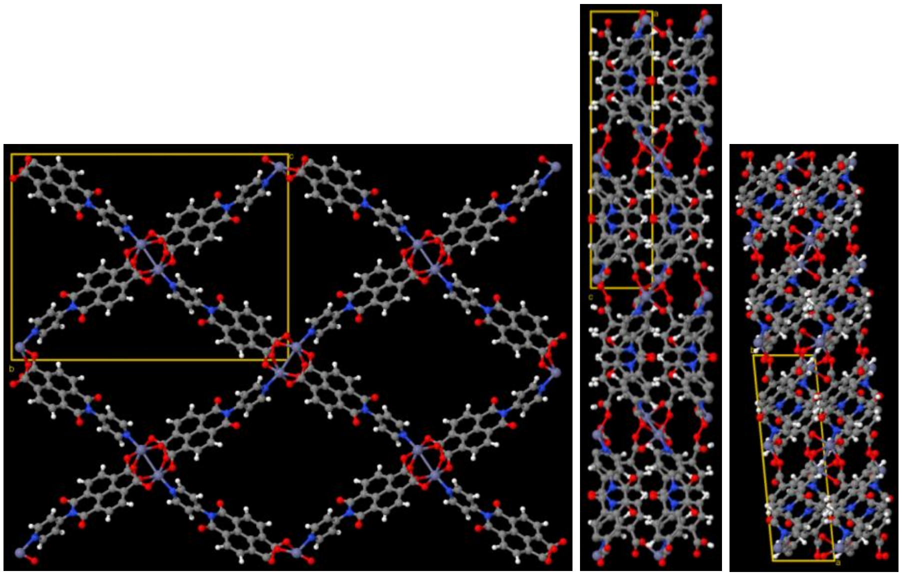

Figure S120: XEXMEU contains the chemical elements Zn, H, C, N, and O.

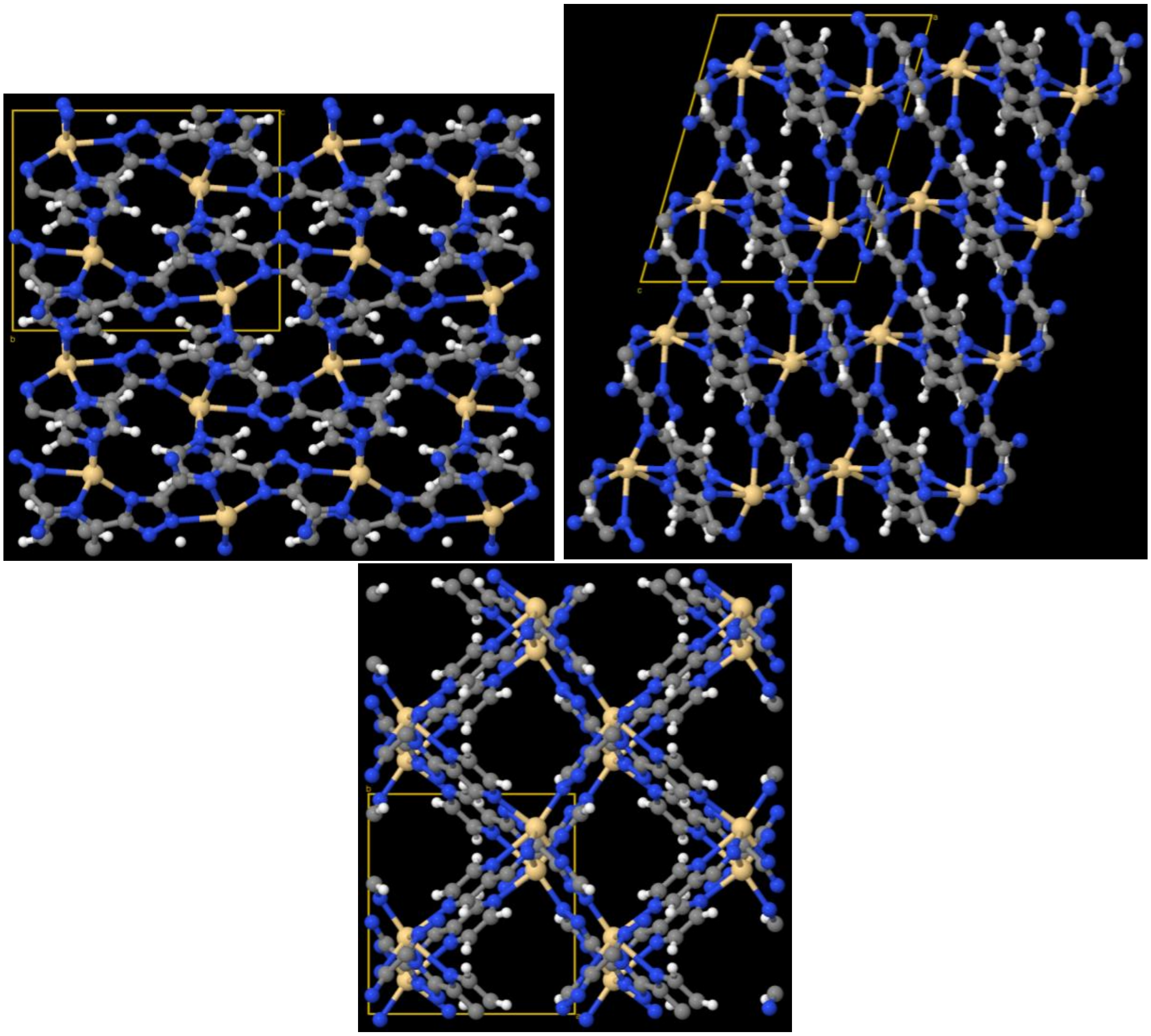

Figure S121: XORGUI contains the chemical elements Cd, N, C, and H.

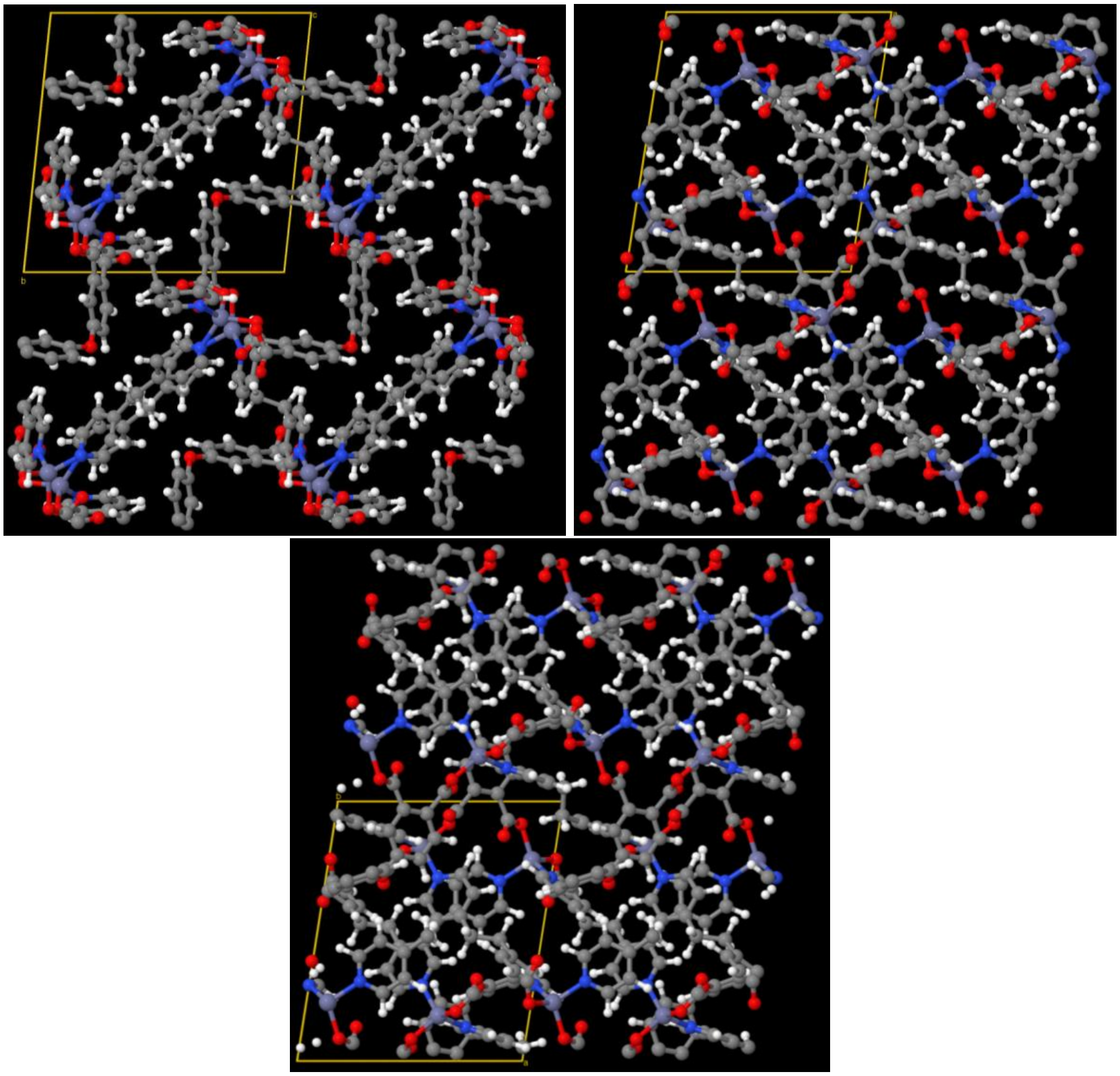

Figure S122: YARKEJ contains the chemical elements Zn, H, C, N, and O.

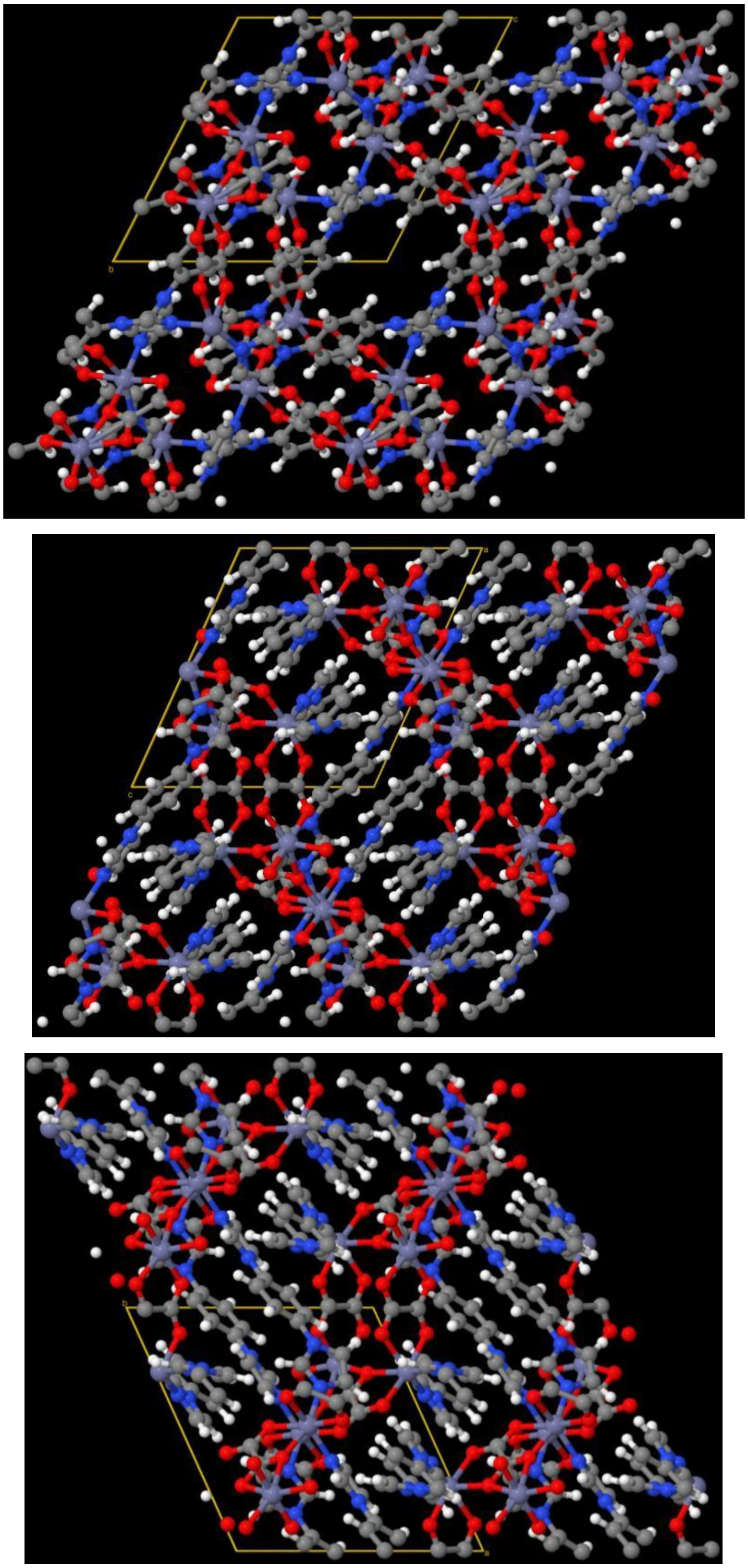

Figure S123: YARSIV contains the chemical elements Zn, H, C, N, and O.

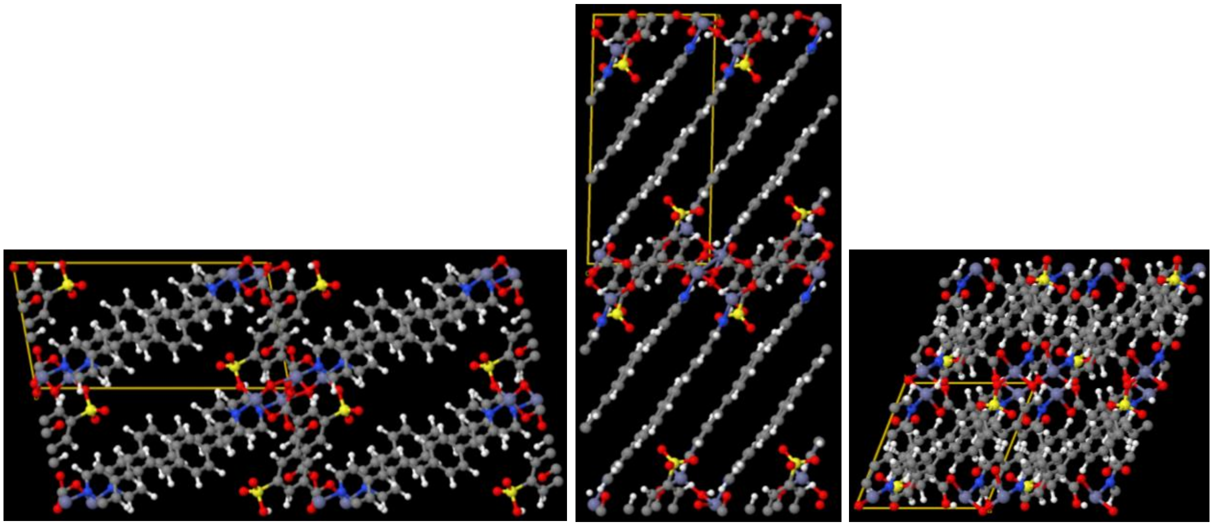

Figure S124: YUSGID contains the chemical elements Zn, H, C, S, N, and O.

Reference

S1. (a) T. A. Manz, A formally exact theory to construct nonreactive forcefields using linear regression to optimize bonded parameters, *RSC Adv.*, 2024, **14**, 33345-33383, doi:10.1039/d4ra01861c. (b) T. A. Manz, Dihedral-torsion model potentials that include angle-damping factors, *RSC Adv.*, 2025, **15**, 7257-7306, doi:10.1039/d4ra08960j.
